# Effects of control interventions on *Clostridium difficile* infection in England: an observational study

**DOI:** 10.1016/S1473-3099(16)30514-X

**Published:** 2017-04

**Authors:** Kate E Dingle, Xavier Didelot, T Phuong Quan, David W Eyre, Nicole Stoesser, Tanya Golubchik, Rosalind M Harding, Daniel J Wilson, David Griffiths, Alison Vaughan, John M Finney, David H Wyllie, Sarah J Oakley, Warren N Fawley, Jane Freeman, Kirsti Morris, Jessica Martin, Philip Howard, Sherwood Gorbach, Ellie J C Goldstein, Diane M Citron, Susan Hopkins, Russell Hope, Alan P Johnson, Mark H Wilcox, Timothy E A Peto, A Sarah Walker, Derrick W Crook, Modernising Medical Microbiology Informatics Group, Carlos Del Ojo Elias, Charles Crichton, Vasiliki Kostiou, Adam Giess, Jim Davies

**Affiliations:** a Nuffield Department of Clinical Medicine, Oxford University, Oxford, UK; b National Institute for Health Research (NIHR) Oxford Biomedical Research Centre, John Radcliffe Hospital, Oxford, UK; c NIHR Health Protection Research Unit in Healthcare Associated Infection and Antimicrobial Resistance at University of Oxford in partnership with Public Health England, Oxford, UK; d Department of Infectious Disease Epidemiology, and NIHR Health Protection Research Unit in Healthcare Associated Infection and Antimicrobial Resistance at Imperial College London in partnership with Public Health England, Imperial College, London, London, UK; e Department of Zoology, Oxford University, Oxford, UK; f Wellcome Trust Centre for Human Genetics, University of Oxford, Oxford, UK; g Public Health England Academic Collaborating Centre, Oxford, UK; h Microbiology Department, Oxford University Hospitals NHS Trust, Oxford, UK; i Leeds Teaching Hospitals and University of Leeds, Department of Microbiology, Leeds General Infirmary, Leeds, UK; j Leeds Teaching Hospitals NHS Trust, Leeds, UK; k Cubist Pharmaceuticals, Lexington, MA, USA; l Tufts University School of Medicine, Boston, MA, USA; m R M Alden Research Laboratory, Culver City, CA USA; n Healthcare-Associated Infection, Antimicrobial Resistance and Stewardship and Healthcare-Associated Infections Programme, Public Health England, London, UK; o Royal Free London NHS Foundation Trust and Public Health England, London, UK; p Department of Healthcare-Associated Infections and Antimicrobial Resistance, Centre for Infectious Disease Surveillance and Control, National Infection Service, Public Health England, London, UK

## Abstract

**Background:**

The control of *Clostridium difficile* infections is an international clinical challenge. The incidence of *C difficile* in England declined by roughly 80% after 2006, following the implementation of national control policies; we tested two hypotheses to investigate their role in this decline. First, if *C difficile* infection declines in England were driven by reductions in use of particular antibiotics, then incidence of *C difficile* infections caused by resistant isolates should decline faster than that caused by susceptible isolates across multiple genotypes. Second, if *C difficile* infection declines were driven by improvements in hospital infection control, then transmitted (secondary) cases should decline regardless of susceptibility.

**Methods:**

Regional (Oxfordshire and Leeds, UK) and national data for the incidence of *C difficile* infections and antimicrobial prescribing data (1998–2014) were combined with whole genome sequences from 4045 national and international *C difficile* isolates. Genotype (multilocus sequence type) and fluoroquinolone susceptibility were determined from whole genome sequences. The incidence of *C difficile* infections caused by fluoroquinolone-resistant and fluoroquinolone-susceptible isolates was estimated with negative-binomial regression, overall and per genotype. Selection and transmission were investigated with phylogenetic analyses.

**Findings:**

National fluoroquinolone and cephalosporin prescribing correlated highly with incidence of *C difficile* infections (cross-correlations >0·88), by contrast with total antibiotic prescribing (cross-correlations <0·59). Regionally, *C difficile* decline was driven by elimination of fluoroquinolone-resistant isolates (approximately 67% of Oxfordshire infections in September, 2006, falling to approximately 3% in February, 2013; annual incidence rate ratio 0·52, 95% CI 0·48–0·56 *vs* fluoroquinolone-susceptible isolates: 1·02, 0·97–1·08). *C difficile* infections caused by fluoroquinolone-resistant isolates declined in four distinct genotypes (p<0·01). The regions of phylogenies containing fluoroquinolone-resistant isolates were short-branched and geographically structured, consistent with selection and rapid transmission. The importance of fluoroquinolone restriction over infection control was shown by significant declines in inferred secondary (transmitted) cases caused by fluoroquinolone-resistant isolates with or without hospital contact (p<0·0001) versus no change in either group of cases caused by fluoroquinolone-susceptible isolates (p>0·2).

**Interpretation:**

Restricting fluoroquinolone prescribing appears to explain the decline in incidence of *C difficile* infections, above other measures, in Oxfordshire and Leeds, England. Antimicrobial stewardship should be a central component of *C difficile* infection control programmes.

**Funding:**

UK Clinical Research Collaboration (Medical Research Council, Wellcome Trust, National Institute for Health Research); NIHR Oxford Biomedical Research Centre; NIHR Health Protection Research Unit on Healthcare Associated Infection and Antimicrobial Resistance (Oxford University in partnership with Public Health England [PHE]), and on Modelling Methodology (Imperial College, London in partnership with PHE); and the Health Innovation Challenge Fund.

## Introduction

*Clostridium difficile* infection is a major clinical challenge worldwide.^1^, ^2^ At least three antimicrobial classes are deemed to be high-risk *C difficile* infection triggers,^3^ including most cephalosporins, to which *C difficile* is inherently resistant,^4^ and clindamycin, to which genotypes causing early outbreaks were resistant.^5^, ^6^, ^7^ Global dispersion of hypervirulent NAP1/PCR-ribotype-027 *C difficile* revealed an association between fluoroquinolone resistance and epidemic spread.^8^, ^9^ Accordingly, clindamycin or fluoroquinolone use has been restricted, and combined with other measures aiming to control localised *C difficile* infection outbreaks.^7^, ^10^, ^11^

Most cases of *C difficile* infection are temporally associated with health care,^2^ reflecting a combination of health-care-associated acquisition, and health-care-related triggers including antibiotics. Three UK studies using highly discriminatory whole genome sequences,^12^, ^13^, ^14^ and a US study using alternative high-resolution typing,^15^ found as few as a third of *C difficile* infections involved recent acquisition from an active case, leaving the source for two-thirds of infections unexplained.

**Research in context**

**Evidence before this study**

We searched PubMed with the terms “Clostridium difficile” AND “sequencing” for articles published in English on or before Feb 1, 2016. We prioritised articles including whole genome sequences. We also reviewed the references of papers identified by this strategy. In previous studies, whole-genome sequencing of *C difficile* was done to investigate its transmission and evolution. We identified no studies in which whole genome sequence based phylogenies were used to investigate the specific role of fluoroquinolone susceptibility or selection in the changing molecular epidemiology or incidence of *C difficile* infection. England is almost unique in experiencing a marked, recent decline in the incidence of health-care-associated *C difficile* infections. Previous reports showed the decline of one epidemic genotype (PCR-ribotype 027), whereas other genotypes appeared to persist. These changes followed the implementation of a multifaceted national *C difficile* infection control policy in 2007. However, the relative contributions made by the different interventions that were introduced simultaneously is unknown.

**Added value of this study**

This study is the first to investigate the contribution of specific public health interventions to the marked national decline in *C difficile* infection. Our novel approach involved the integrated analysis of multiple, large, concurrent datasets concerning incidence of *C difficile* infection, antimicrobial prescribing, and, crucially, the whole genome sequences of more than 4000 human *C difficile* isolates. Our key finding was that the post-interventions decline in *C difficile* infections reflected the disappearance of fluoroquinolone-resistant isolates (predominantly from four genetically distinct genotypes), whereas the incidence of *C difficile* infections caused by fluoroquinolone-susceptible isolates (of many different genotypes) remained unchanged. Whole genome sequence-based phylogenetic analyses of the entire *C difficile* population, with one phylogeny constructed for each genotype, identified shorter, geographically clustered branches, specific to the fluoroquinolone-resistant regions. This finding is consistent with rapid nosocomial transmission preceding the disappearance of fluoroquinolone-resistant isolates. Among the susceptible isolates, the numbers that were closely genetically related (and by inference transmitted, either directly or indirectly) did not change over time. This lack of change was despite the implementation of comprehensive infection prevention and control measures, which would have targeted fluoroquinolone-resistant and susceptible *C difficile* equally. These data suggest that it was the restriction of fluoroquinolone prescribing, above other interventions (including cephalosporin restriction and infection control precautions), that appears to explain the decline in incidence of *C difficile* infections.

**Implications of all the available evidence**

The restriction of fluoroquinolone prescribing should be a cornerstone in the control of epidemic *C difficile* infections in the UK and worldwide.

By comparison with other countries,^1^, ^2^ the incidence of *C difficile* infection in England decreased markedly over the past decade,^16^ after the introduction of national *C difficile* infection prevention and management policies from June, 2007.^17^, ^18^ These included recommendations to avoid clindamycin and cephalosporins, minimised use of fluoroquinolone, carbapenem and aminopenicillin, and improved infection prevention and control activities (appendix).^17^ We investigated the effect of these interventions on *C difficile* evolution, selection, and transmission, to inform future *C difficile* infection control policies for this global challenge.

## Methods

### Study design

This observational study tested two hypotheses. First, if *C difficile* infection declines in England were driven by reductions in use of particular antibiotics, then incidence of *C difficile* infection caused by resistant isolates should decline faster than that caused by susceptible isolates across several genotypes (defined by multilocus sequence type). Second, if decreases in *C difficile* infection were driven by improvements in hospital infection control, then transmitted (secondary) cases should decline regardless of susceptibility.

To confirm that national policies^17^, ^18^ affected antibiotic prescribing and *C difficile* infection incidence, we first compared national antimicrobial prescribing data for hospitals and the community (obtained respectively from IMS Health [Danbury, CT, USA] and the Health & Social Care Information Centre [appendix]) with national incidence of *C difficile* infection (ie, infections per English population per year, using data from Public Health England).

The primary study dataset comprised whole genome sequences from clinical *C difficile* isolates cultured from consecutive toxin enzyme immunoassay (EIA)-positive stool samples from symptomatic, unique patients submitted to the Oxford University Hospitals NHS Trust between Sept 12, 2006, and Aug 19, 2013 (n=2021; appendix). A further 261 isolates between Sept 1, 2006, and Feb 26, 2013, where only the sequence type was available were also included. The hospital did all *C difficile* testing in Oxfordshire, serving general practices, community hospitals, and other providers, so incidence is per Oxfordshire population (approximately 600 000) per year. This culture-positive *C difficile* infection incidence was compared with Oxfordshire's nationally submitted EIA-positive incidence (incorporating changes in mandatory reporting requirements in 2008) to confirm representativeness of whole genome sequences. The latter was compared with English incidence of *C difficile* infection to assess generalisability.

Generalisability of Oxfordshire data was also assessed with similar information from Leeds Teaching Hospitals NHS Trust, UK. This comprised whole genome sequences for consecutive clinical, toxin-positive (cytotoxin assay) isolates from symptomatic patients (Aug 2, 2010, to May 1, 2013; n=1020; appendix), Leeds regional incidence of *C difficile* infection data (nationally submitted) and ribotype prevalence, and antibiotic prescribing data.

Additional genetic context was provided by further regional and international *C difficile* whole genome sequences (May 9, 2006, to July 12, 2013) of isolates from toxin-EIA-negative clinical samples of symptomatic Oxfordshire patients (n=395), toxin-positive samples representing two clinical trials of fidaxomicin in North America and Europe (n=803),^19^, ^20^ and from healthy Oxfordshire infants (non-clinical; n=200; appendix).

### Genome sequences and multilocus sequence type identification

Genomes were sequenced using Illumina technology. Velvet de novo assemblies and reference-based assemblies were generated, the latter mapped to *C difficile* 630 (GenBank AM180355.1; reads submitted to National Center for Biotechnology Information, BioProjectID PRJNA304087; appendix). The sequences of loci defining *C difficile* sequence types were identified and extracted with BIGSdb;^21^ sequence types were assigned with the *C difficile* PubMLST database. The notation ST1(027) indicates, for example, sequence-type-1 (PCR-ribotype-027).

### Whole genome sequence-derived fluoroquinolone susceptibility

Isolates were designated fluoroquinolone-susceptible or fluoroquinolone-resistant based on specific non-synonymous substitutions within the quinolone resistance-determining region of *gyrA/B* genes^22^, ^23^ extracted from whole genome sequences.^21^ *gyrA* C(245)T[T(82)I] and *gyrB* G(1276)A [D(426)N] confer high-level fluoroquinolone resistance in *C difficile* and other species.^16^, ^17^ Susceptibility predictions were validated phenotypically for 387 fidaxomicin trial isolates^19^, ^20^ (n=191 Canada, n=196 USA), with agar dilution (moxifloxacin minimum inhibitory concentration; appendix).

### Statistical analysis

We made univariable comparisons between English antimicrobial prescribing and incidence of *C difficile* infection with bivariate cross-correlations (appendix). Genotype (sequence type)-specific incidence rates for *C difficile* infection caused by toxin EIA-positive, culture-positive isolates were calculated with negative binomial regression accounting for missing data by probability weights (appendix). For genotypes with more than 10% fluoroquinolone-resistant isolates, rates were calculated separately for fluoroquinolone-susceptible and fluoroquinolone-resistant isolates. These data were available for isolates from April 2008 to March 2011. Rates were also calculated separately for infections that could plausibly have arisen from secondary spread (transmission) inferred by close genetic relationships to previous infections (two or fewer single nucleotide variants from the original case),^12^ and also separately for fluoroquinolone-susceptible and fluoroquinolone-resistant isolates. Phylogenetic trees were constructed for each sequence type (or several closely related sequence types), with maximum likelihood, then corrected for recombination using ClonalFrameML (version 1.0–6).^24^ Trees were time-scaled and made directly comparable post-1990 (appendix). In each tree, the evolutionary distinctiveness (ED) score of each genome was calculated;^25^ low ED scores indicate closely related genomes, whereas high scores indicate their relative absence (appendix).

### Role of the funding source

The study sponsor had no role in study design, data collection, data analysis, data interpretation, or writing of the report. The corresponding author had full access to all study data and had final responsibility for the decision to submit for publication.

## Results

**Figure 1:**
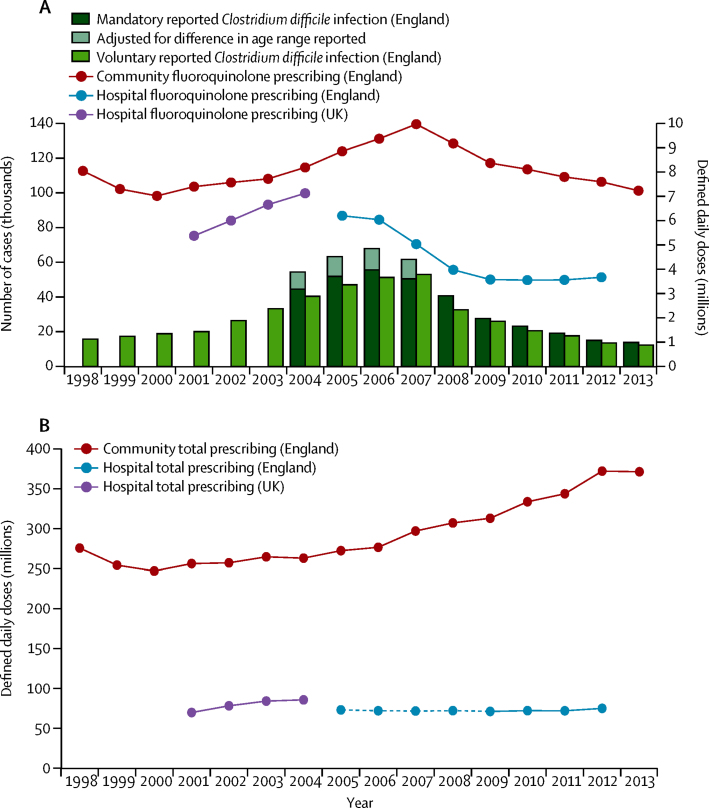
National incidence of *Clostridium difficile* infections and fluoroquinolone prescribing (A) and national antibiotic prescribing overall (B) (A) Mandatory incidence of *C difficile* infections corresponds to all infections reported for individuals older than 2 years (from 2004 to 2007, infections were only reported for individuals older than 65 years, and are upweighted to provide similar estimates in individuals older than 2 years; appendix). Since mandatory reporting was only introduced in 2004, we have also included voluntary-reported *C difficile* infections to give an indication of trends before that date. (B) Dotted lines are estimates (appendix).

Incidence of *C difficile* infection in England increased from 1998 to 2006 (p<0·0001) then declined rapidly over the years that followed to 2013 (p<0·0001; figure 1A). *C difficile* infection declines occurred while total antibiotic prescribing was increasing (by 4·4% per year in the community [p<0·0001, 2006–13], but only 0·5% per year in hospitals [p=0·053, 2006–12]; figure 1B). Between 2005 and 2012 (when data were complete for England), the cross-correlations (CCs) between English incidence of *C difficile* infection and total English antibiotic prescribing were −0·57 (95% CI −0·67 to −0·41) for hospital and community, −0·59 (−0·68 to −0·44) for community, and 0·29 (−0·19 to 0·60) for hospital prescribing (optimum CC using a 1-year lag; appendix). During the same period, the strongest univariable associations between English incidence of *C difficile* infection and individual antimicrobials were with cephalosporins (CC=0·97, 95% CI 0·82–0·98 for hospital and community; 0·94, 0·68–0·97 for community; and 0·97, 0·81–0·99 for hopital prescribing; optimum 0-year lag) and fluoroquinolones (CC=1·00, 0·84–1·00 for hospital and community; 0·88, 0·48–0·95 for community; and 0·93, 0·66–0·97 for hospital prescribing; optimum 0-year lag; appendix), although hospital fluoroquinolone prescribing began to decline slightly earlier than community prescribing (p<0·0001 from 2005 to 2009 *vs* in the community p<0·0001 from 2007 to 2012; figure 1A). Other antibiotics were more weakly associated (appendix).

**Figure 2:**
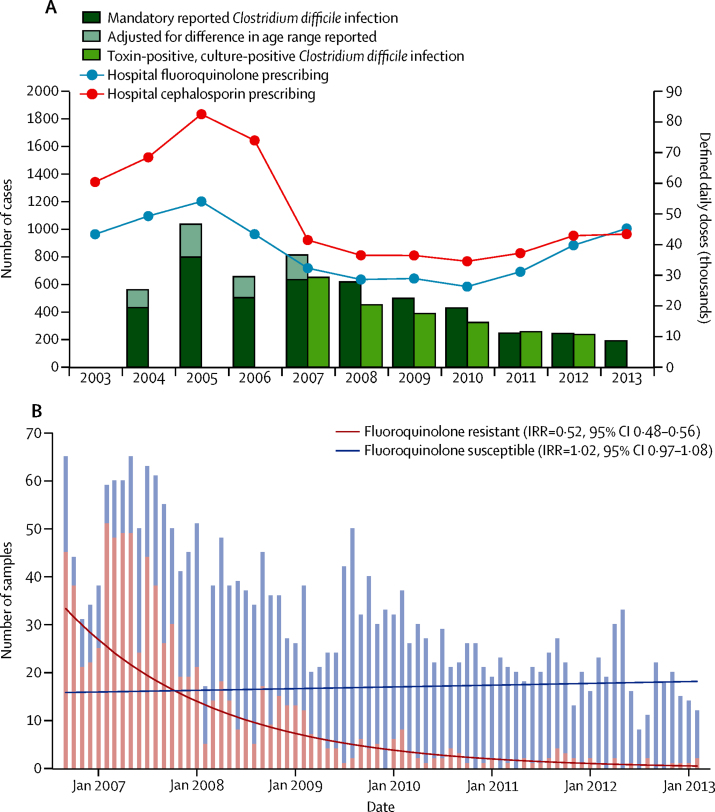
Incidence of *Clostridium difficile* infections together with fluoroquinolone and cephalosporin prescribing for Oxfordshire (A) and incidence of *C difficile* infections by fluoroquinolone susceptibility for Oxfordshire (B) (A) Mandatory incidence of *C difficile* infections corresponds to all cases reported for individuals older than 2 years (from 2004 to 2007, cases were only reported for individuals older than 65 years, and are upweighted to provide similar estimates in individuals older than 2 years; appendix). Only toxin-positive culture-positive samples were used in the genotype-specific and phylogenetic analyses. (B) *C difficile* is inherently resistant to most cephalosporins.^4^ IRR=annual incidence rate ratio.

Similar to English incidence of *C difficile* infection, Oxfordshire rates also decreased from 2007 (when isolate-level fluoroquinolone-susceptibility could be determined; p<0·0001; figure 2A). Fluoroquinolone prescribing in Oxfordshire hospitals declined from a peak in 2005 until 2010 (p<0·0001), when use began to increase again (p<0·0001 from 2010 to 2013). Hospital cephalosporin and fluoroquinolone prescribing were also positively associated with incidence of *C difficile* infection (CC=0·73, 0·15 to 0·86, and 0·62, −0·09 to 0·81; appendix), but associations were estimated much less precisely given the much smaller population (approximately 1% of England). Positive associations were also observed between *C difficile* infection decline and decline in extended spectrum penicillins (0·84, 0·24 to 0·90) and beta-lactamase-resistant penicillins (0·67, −0·04 to 0·81; appendix). Community prescribing data were not available.

Paired fluoroquinolone susceptibility phenotype and *gyrA*/*B* DNA sequences were assessed for 387 isolates from the two clinical trials of fidaxomicin in North America and Europe,^19^, ^20^ representing 53 sequence types. Phenotype and whole genome sequences were 98·7% concordant (appendix; sensitivity 97·8%, specificity 99·5%); only one of 185 isolates predicted as resistant by whole genome sequences^22^, ^23^ lacked an elevated minimum inhibitory concentration (MIC). Conversely, only four of 202 isolates lacking resistance-associated substitutions^22^, ^23^ had raised MICs (16 mg/L). *gyrA*/*B* sequence therefore reliably predicts the fluoroquinolone resistance phenotype.

**Figure 3:**
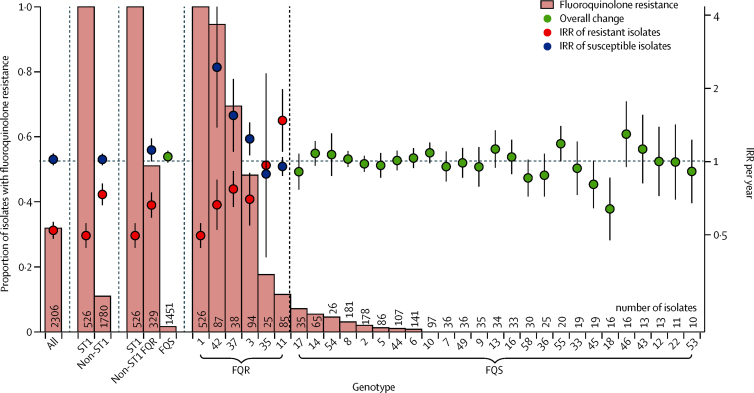
Oxfordshire *Clostridium difficile* IRR by fluoroquinolone resistance and genotype For genotypes with more than 10% resistant isolates (denoted FQR), rates were calculated separately for *C difficile* infections caused by fluoroquinolone-susceptible and resistant isolates. To show that the difference in IRR for resistant and susceptible isolates is not driven solely by the decline in ST1(027), rates were also calculated for all non-ST1(027) genotypes together, as well as for all genotypes with more than 10% resistant isolates (excluding ST1(027)) and for all genotypes with 10% or less resistant isolates (FQS). Heterogeneity between IRRs in *C difficile* infections caused by fluoroquinolone-resistant versus fluoroquinolone-susceptible isolates: all p<0·0001, non-ST1 p<0·0001, non-ST1 FQR p<0·0001, ST42 p<0·0001, ST37 p=0·00015, ST3 p=0·00070, ST35 p=0·92, ST11 p=0·0053. The dotted vertical lines separate out the different ways the isolates were divided for the different analyses. The horizontal dotted line represents an IRR of 1. IRR=annual incidence rate ratio.

**Figure 4:**
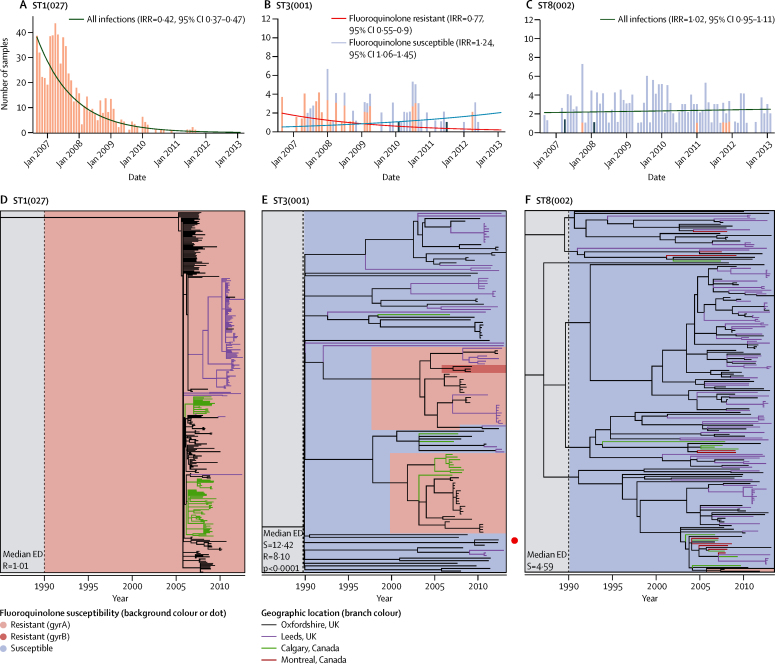
Contrasting incidence of *Clostridium difficile* infections (Oxfordshire) and whole-genome sequence phylogenies representing the fluoroquinolone-resistant genotype ST1(027), the mixed resistant and susceptible genotype ST3(001), and the almost entirely fluoroquinolone-susceptible genotype ST8(002) (A) Incidence of *C difficile* infections by fluoroquinolone susceptibility for genotype ST1(027) in Oxfordshire. Red bars indicate fluoroquinolone-resistant isolates, blue bars indicate fluoroquinolone-susceptible isolates, grey bars indicate resistance not determined. (B) Incidence of *C difficile* infections by fluoroquinolone susceptibility for genotype ST3(001) in Oxfordshire. (C) Incidence of *C difficile* infections by fluoroquinolone susceptibility for genotype ST8(002) in Oxfordshire. (D) Time-scaled phylogeny for ST1(027) generated with ClonalFrameML.^24^ Every third Oxfordshire isolate (by date) is shown. Phylogenies were scaled to be directly similar post-1990; the grey shaded regions before 1990 represent the regions of the phylogenies that should not be compared because they are not scaled identically. Background colour indicates fluoroquinolone susceptibility; branch colour indicates geographic location. (E) Time-scaled phylogeny for the mixed fluoroquinolone resistant or susceptible genotype, ST3(001), generated using ClonalFrameML.^24^ Two fluoroquinolone-resistant areas of the phylogeny are indicated by red shading within the blue susceptible region. Rapid clonal expansion after resistance emergence is supported by significantly lower ED scores for resistant versus susceptible areas. (F) Time-scaled phylogeny for ST8(002) generated using ClonalFrameML.^24^ Every second Oxfordshire isolate (by date) is shown. Two fluoroquinolone-resistant isolates are indicated at the bottom of the panel. IRR=annual incidence rate ratio. ED=evolutionary distinctiveness.^25^ R=fluoroquinolone resistant. S=fluoroquinolone susceptible.

The decrease in Oxfordshire *C difficile* infections was solely due to a decline in *C difficile* infection caused by fluoroquinolone-resistant isolates, estimated at approximately 67% of all Oxfordshire *C difficile* infections in September, 2006, falling to approximately 3% by February, 2013 (annual incidence rate ratio [aIRR] 0·52, 95% CI 0·48–0·56, p<0·0001; figure 2B). Most (62%) fluoroquinolone-resistant isolates were from genotype ST1(027), but the decline persisted even when excluding ST1(027) and pooling the remaining fluoroquinolone-resistant isolates (aIRR 0·73, 0·66–0·81, p<0·0001 for all non-ST1; 0·66, 0·59–0·75, p<0·0001 for all non-ST1 with >10% resistant isolates; figure 3, appendix). Considering genotypes containing more than 10% resistant isolates separately, *C difficile* infection caused by fluoroquinolone-resistant isolates declined significantly for four genotypes from three distinct chromosomal backgrounds:^26^ clade 1 ST42(106) (p=0·00076), ST3(001) (p=0·0054); clade 2 ST1(027) (p<0·0001) and clade 4 ST37(017) (p=0·0027; figure 3, figure 4A, B, appendix).

The incidence of *C difficile* infection caused by fluoroquinolone-susceptible isolates remained unchanged (aIRR 1·02, 95% CI 0·97–1·08, p=0·45; figure 2B; heterogeneity p<0·0001 *vs* fluoroquinolone-resistant), and actually increased in three of the five genotypes with more than 10% but less than 99% resistant isolates (figure 3, figure 4B, appendix). More limited data for Leeds, representing a geographically independent region, were broadly similar (aIRR0·55, 0·49–0·61, p<0·0001 pooling predominantly fluoroquinolone-resistant ribotypes versus 1·03, 1·01–1·05, p=0·0031 pooling fluoroquinolone-susceptible ribotypes; appendix), as were national ribotyping data,^27^ supporting generalisability.

19 phylogenies were constructed representing the 22 most common *C difficile* genotypes in Oxfordshire and Leeds (figure 4D–F, appendix). The phylogeny of each genotype containing more than 10% fluoroquinolone-resistant isolates (figure 4D, E, appendix) showed rapid, geographically structured clonal expansions associated with resistance. This observation was reproduced internationally in parts of the phylogenies representing Calgary, Canada (figure 4D, E) and in isolates from three cities in northern Italy: Modena, Turin, and Arsizio (appendix). We recorded significantly lower ED scores for resistant versus susceptible areas of phylogenies containing both fluoroquinolone-resistant and fluoroquinolone-susceptible isolates (eg, ST3 p<0·0001, figure 4E; ST37 p<0·0001, appendix). By contrast, the phylogenies of genotypes consisting primarily of susceptible isolates (figure 4F, appendix) were geographically unstructured and had longer branches. This was also seen internationally in susceptible isolates from Calgary and Montreal, Canada (figure 4E, appendix). In fluoroquinolone-susceptible genotypes, the ED scores (and, by inference, transmission) did not differ significantly between Oxfordshire and Leeds clinical isolates (p>0·1; appendix).

Additional phylogenies for three prevalent fluoroquinolone-susceptible genotypes revealed similar branch lengths irrespective of sampling region size (appendix). Oxfordshire phylogenies (appendix), containing genomes from toxin EIA-positive and EIA-negative samples, plus genomes from healthy, asymptomatic, community infants, showed a lack of structure by source, even within a single region. ED scores were generally lower for clinical toxin EIA-positive genomes than for infant and EIA-negative genomes, especially in ST8(002) (p=0·0033) and ST2(014/020) (p=0·0014; appendix), consistent with greater transmission in the former.

**Figure 5:**
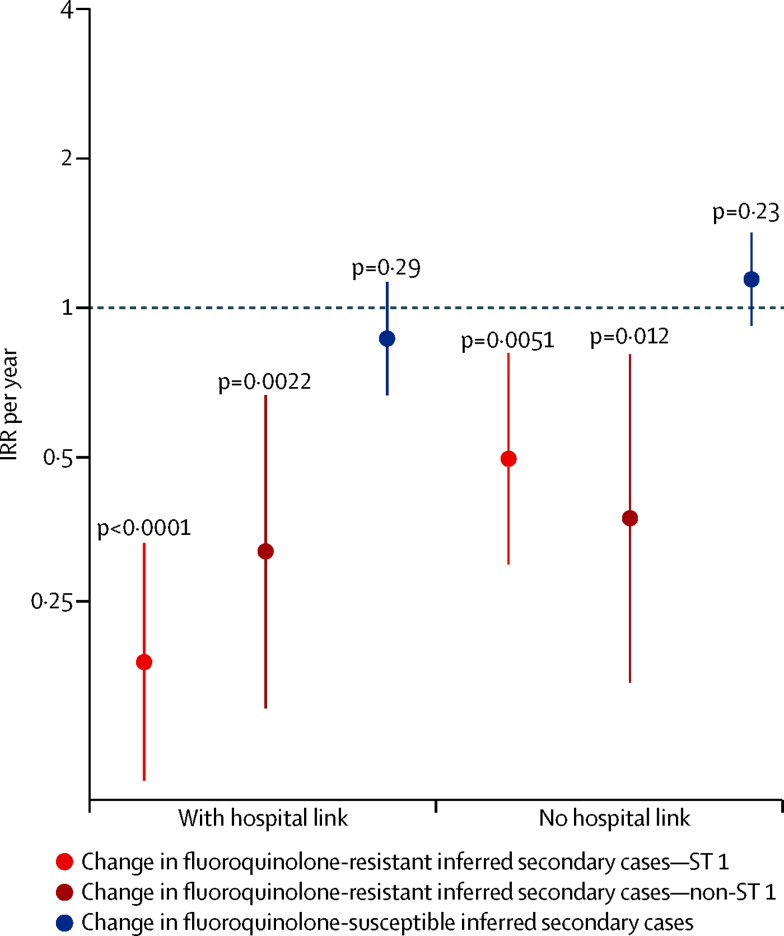
Incidence trends of inferred secondary *Clostridium difficile* cases in Oxfordshire from April 2008 to March 2011 Inferred secondary cases are those caused by *C difficile* isolates that are genetically closely related (≤two single nucleotide variants) to isolates recovered from a previous case, and therefore potentially transmitted. Incidence trends were calculated separately for inferred secondary cases caused by fluoroquinolone-resistant ST1(027), fluoroquinolone-resistant non-ST1(027), and fluoroquinolone-susceptible isolates, stratified by with versus without hospital-based contact. Horizontal dotted line shows an IRR per year of 1 (ie, no change over time) against which the 95% CI bars are compared to determine statistical significance of any change. The p values are a test of the IRR against the null hypothesis of no change over time (IRR=1). IRR=annual incidence rate ratio.

Fluoroquinolone restriction and multiple enhanced infection control measures were introduced simultaneously in England in 2007.^17^ Therefore, we investigated the hypothesis that infection control, not antimicrobial stewardship, reduced incidence of C *difficile* infection by reducing transmission (eg, that fluoroquinolone-resistant isolates were simply more prevalent in hospitals where infection control efforts were concentrated). Secondary spread (transmission) was inferred when subsequent infections had closely genetically related isolates. We estimated the Oxfordshire incidence of inferred secondary cases separately for fluoroquinolone-resistance versus fluoroquinolone-susceptibility, and also for infections where hospital-based contact occurred between primary and secondary cases.^12^ There was strong evidence for declines in secondary *C difficile* infections caused by fluoroquinolone-resistant isolates, both with hospital contact with a previous case (aIRR 0·21, 95% CI 0·13–0·34, p<0·0001) and without (0·45, 0·29–0·71, p<0·0001; figure 5). Declines occurred in secondary cases caused by fluoroquinolone-resistant isolates of ST1(027) and non-ST1(027) genotypes (p≤0·012, appendix). By contrast, there was no evidence of declines in secondary cases caused by fluoroquinolone-susceptible isolates, either with hospital contact with a previous infection (0·87, 0·67–1·13, p=0·29) or without (1·14, 0·92–1·42, p=0·23), supporting the importance of fluoroquinolone restriction over infection control interventions.

## Discussion

Our analysis of multiple whole genome sequence datasets shows that reductions in the incidence of *C difficile* infections caused by fluoroquinolone-resistant isolates (of multiple genotypes) plausibly has driven the decline in *C difficile* infections in Oxfordshire and Leeds, England, from 2007. Declines occurred alongside significant reductions in fluoroquinolone use in hospitals and the community. Extensive whole genome sequence phylogenies show that acquisition of fluoroquinolone resistance preceded the emergence of multiple, prevalent genotypes (figure 4, appendix); after fluoroquinolone prescribing was controlled, incidence declines were specific to *C difficile* infections caused by fluoroquinolone-resistant isolates of these same genotypes (figure 3, figure 4, appendix). By contrast, the incidence of *C difficile* infections from multiple fluoroquinolone-susceptible genotypes remained constant (figure 3, figure 4C, appendix), unaffected by changes in fluoroquinolone use or other national policy measures, such as restricted cephalosporin prescribing and enhanced infection control interventions, irrespective of genotype (figure 5, appendix).^17^ Crucially, there was no evidence of a decline in plausibly nosocomially transmitted secondary cases caused by fluoroquinolone-susceptible *C difficile*, which would be expected if improved infection control had made a major contribution to *C difficile* infection declines, whereas secondary cases caused by fluoroquinolone-resistant *C difficile* decreased markedly (figure 5, appendix).

The phylogenetically estimated date of fluoroquinolone resistance emergence preceded the clinical emergence of several problematic *C difficile* genotypes of different phylogenetic clades:^26^ ST1(027),^9^ ST42(106), ST3(001), and ST37(017) (figure 4, appendix).^28^, ^29^ The recent emergence of fluoroquinolone-resistant ST17(018) in Italy (appendix) also followed high fluoroquinolone use.^30^ Our greater sampling density^9^ revealed short-branched, geographically structured phylogenies of fluoroquinolone-resistant *C difficile* consistent with rapid spread within hospitals, and occasional transmission between them (figure 4D–F, appendix). Inclusion of international isolates allowed us to show generalisability of our findings outside of the UK. Although fluoroquinolone-susceptible, limited ST8(002) and ST2(014/020) transmission plausibly occurred, as indicated by small, short-branched clusters, and lower ED scores for clinical-toxin EIA-positive isolates than for infant/EIA-negative isolates (appendix). However, the absence of large-scale geographic structure in the long-branched phylogenies of all fluoroquinolone-susceptible genotypes (appendix) suggests that most were introduced independently into the clinical environment from alternative potential reservoirs.^31^, ^32^ Fluoroquinolone-susceptible *C difficile* might therefore represent a population lacking large-scale adaptation to antimicrobial selection pressures of clinical environments.

The decline in incidence of *C difficile* infection after national restriction of high-risk antimicrobials is consistent with previously successful small-scale interventions restricting high-risk antimicrobials as part of control packages.^7^, ^10^, ^11^ However, our study showed conclusively that Oxfordshire declines of *C difficile* infection were due to the parallel disappearance of fluoroquinolone-resistant isolates of multiple genotypes (figure 2, figure 3), suggesting that any selective advantage specific to resistant isolates might be lost when the antimicrobial is withdrawn. In England, additional antimicrobials were also targeted for restriction.^17^ However, only cephalosporin use also fell (figure 2A, appendix). Because all *C difficile* is inherently resistant to most cephalosporins,^4^ their restriction cannot explain the fluoroquinolone-susceptibility-specific declines in incidence observed. Similarly, if an ST1(027)-specific factor had led to its decline, there would be no reason for *C difficile* infection caused by fluoroquinolone-resistant isolates of several other genotypes—ST42(106), ST3(001), and ST37(017)—in two other *C difficile* clades (1 and 3)^26^ to decline concurrently (figure 3, figure 5). Although univariate cross-correlations between decline of *C difficile* infection and hospital-prescribed extended-spectrum penicillins (mostly amoxicillin alone) and beta-lactamase resistant penicillins (mostly flucloxacillin alone) were stronger than for fluoroquinolones in Oxfordshire, the use of many antibiotics in these groups actually rose because they were instead used in combinations, such as co-amoxiclav. Penicillins generally have a lesser risk of provoking *C difficile* infections than other classes of antibiotics,^8^, ^33^ and when taking community prescribing into account, (which forms a larger proportion of overall antimicrobial use than hospital prescribing) the correlation between these penicillin groups and incidence of *C difficile* infection in England disappears. Unfortunately, community prescribing data were not available for Oxfordshire for comparison. Finally, the much smaller population size meant that these univariate cross-correlations were estimated imprecisely compared with those for England as a whole. Our study therefore clarifies the issue of whether fluoroquinolone or cephalosporin restriction alone or in combination is key to *C difficile* infection control.^34^, ^35^, ^36^ However, changes in dominant genotypes over time have been reported in a single centre in the absence of antimicrobial restriction policies.^37^ ST1(027) outbreak control has also been achieved when total antimicrobial (not only fluoroquinolone) use was reduced,^38^ although this change could still predominantly reflect the effect of fluoroquinolones.

Similar to cephalosporin restriction, enhanced infection control measures^17^ such as isolation, contact precautions, and enhanced environmental cleaning do not target specific *C difficile* genotypes and should therefore reduce numbers of symptomatic patients infected with transmitted strains, irrespective of fluoroquinolone susceptibility. Analysis of closely related *C difficile* genomes from different patients (ie, representing possible transmissions^12^ potentially preventable by infection control measures) clearly showed that incidence only fell for secondary cases caused by fluoroquinolone-resistant *C difficile*, irrespective of hospital contact with a previous closely genetically related case, with no change in secondary cases caused by fluoroquinolone-susceptible isolates (figure 5, appendix). This finding is consistent with previous work^38^ finding no change in incidence of *C difficile* infection after infection control procedures were strengthened. This finding supports the greater importance of fluoroquinolone restriction in both hospitals and the community over enhanced infection control in recent reductions in English incidence of *C difficile* infection.

Antimicrobial stewardship targeted all patients in hospitals and the community,^17^ so clinically adapted resistant *C difficile* might conceivably have been eliminated from asymptomatic carriers and cases. If fluoroquinolone-resistant *C difficile* persisted in carriers, outbreak conditions should have returned rapidly once fluoroquinolone prescribing increased. This did not occur even after post-2010 increases in hospital fluoroquinolone prescribing in Oxford and Leeds (figure 2A, appendix). However, whereas before 2007 fluoroquinolones were prescribed widely, including in elderly people, increases after 2010 do not necessarily equate to increased exposure of patients with high risk of *C difficile* infection. Instead, these increases might reflect new, specific indications such as neutropenic prophylaxis (see appendix for Leeds; equivalent data not available in Oxford), consistent with observations that fluoroquinolone use is not a risk factor under non-outbreak conditions.^39^ The lack of national rise in fluoroquinolone-resistant *C difficile* infections also supports their almost complete eradication from both symptomatic patients and asymptomatic carriers in England, consistent with regional (Oxfordshire) findings that by late 2011, fluoroquinolone-resistant isolates of the commonest incidence genotype (ST1(027)) had disappeared from asymptomatic colonisation as well as infection.^31^

The genotypes ST1(027), ST42(106), ST3(001), and ST37(017), accounting for most fluoroquinolone-resistant isolates, represent three divergent *C difficile* clades,^26^ each with a genetically distinct, toxin-encoding pathogenicity locus.^26^ These genotypes could therefore differ in virulence or transmissibility due to varying gene content. ST1(027), for example, is almost four times likelier than other genotypes to cause symptomatic infection^40^ (although this could reflect its fluoroquinolone-resistant phenotype in settings with high fluoroquinolone prescribing). It seems unlikely that other gene content should be completely confounded with fluoroquinolone resistance, particularly within the large clade 1^26^ (containing ST42(106), ST3(001), and Italian ST17(018)). However, even if additional virulence factors are associated with ST1(027), the overall diversity of outbreak-associated genetic backgrounds in which fluoroquinolone resistance is found suggests that this phenotype alone might be sufficient to confer outbreak potential.

A few sporadic fluoroquinolone-resistant isolates were identified in otherwise susceptible genotypes (appendix), suggesting that chance, combined with regional antibiotic prescribing policies, could trigger localised spread. ST11(078) was unusual, in that fluoroquinolone resistance occurred in 24 (13%) of 182 isolates, distributed throughout the phylogeny (appendix). ST11(078) can be transmitted zoonotically,^32^ and the unstructured pattern of fluoroquinolone resistance within this phylogeny could reflect the sporadic emergence of resistance either during agricultural fluoroquinolone use, or after human colonisation and antibiotic exposure.

The main study limitation was being primarily based in one, albeit large (population of approximately 600 000 people) region, where 7 years of individual-isolate whole genome sequences enabled us to predict fluoroquinolone susceptibility. Whole genome sequence data from Leeds were available for less than 3 years, precluding a similar analysis to figure 2 in another region. Different datasets from different sources were used for incidence of *C difficile* infections and antibiotic use because no one dataset was collected consistently across the entire period from a single source. Comparisons of incidence of *C difficile* infections and antibiotic use are ecological, and therefore prone to unmeasured confounding. English hospital-level antibiotic data are not available before 2013 (only subsequently),^41^ so we were unable to investigate associations between fluoroquinolone use and *C difficile* infections across Trusts in a broader ecological analysis. However, our key characteristics, fluoroquinolone susceptibility and genotype, were unknown when the *C difficile* infections occurred and were not part of the inclusion or exclusion criteria. Therefore, the phylogenetic analyses are representative of the genotypes circulating in the locations studied when sampled.

In summary, fluoroquinolone resistance occurs in several genetically divergent *C difficile* genotypes.^26^ The contrasting phylogenies of fluoroquinolone-resistant and fluoroquinolone-susceptible *C difficile* probably reflect increased potential for health-care-associated selection and epidemic spread of fluoroquinolone-resistant bacteria. Thus, the *C difficile* genotypes causing infections at any given time and location, and the relative importance of different transmission routes (nosocomial person-to-person versus multiple introductions) might be a direct consequence of antimicrobial prescribing policies. The multifaceted approach to *C difficile* infection control adopted by England successfully curtailed transmission. Whole genome sequence data suggest that fluoroquinolone restriction plausibly played the most important part in this success. Appropriate antimicrobial stewardship therefore is, and will likely remain, central to the control of *C difficile* infections.
