# Supplementary material for: Effects of control interventions on Clostridium difficile infection in England: an observational study
Source: Lancet Infect Dis. 2017 Apr;17(4):411–21. doi: 10.1016/S1473-3099(16)30514-X (PMC5368411; doi:10.1016/S1473-3099(16)30514-X)
Supplement: Supplementary appendix [file mmc1.pdf]

THE LANCET
Infectious Diseases

Supplementary appendix

This appendix formed part of the original submission and has been peer reviewed. We post it as supplied by the authors.

Supplement to: Dingle K E, Didelot X, Quan T P, et al. Effects of control interventions on Clostridium difficile infection in England: an observational study. *Lancet Infect Dis* 2017; published online Jan 24. http://dx.doi.org/10.1016/S1473-3099(16)30514-X.

Web Extra Material

Elimination of healthcare associated fluoroquinolone-resistant, but not fluoroquinolone-susceptible *Clostridium difficile*: an observational study

Kate E. Dingle, Xavier Didelot, T. Phuong Quan, David W. Eyre, Nicole Stoesser, Tanya Golubchik, Rosalind M. Harding, Daniel J. Wilson, David Griffiths, Alison Vaughan, John M. Finney, David H. Wyllie, Sarah J. Oakley, Modernising Medical Microbiology Informatics Group, Warren N. Fawley, Jane Freeman, Kirsti Morris, Jessica Martin, Philip Howard, Sherwood Gorbach, Ellie J.C. Goldstein, Diane M. Citron, Susan Hopkins, Russell Hope, Alan P. Johnson, Mark H. Wilcox, Timothy E.A. Peto, A. Sarah Walker, and Derrick W. Crook.

Contents

Supplementary Methods **Pg. 2-5**
References **Pg. 6**
Figure S1 A and B. Resistance to moxifloxacin in *C. difficile* can be accurately predicted by the presence of the non-synonymous mutations: *gyrA* C(245)T [T(82)I], or *gyrB* G(1276)A, [D(426)N]. **Pg. 7**
Figure S2 A to F. National hospital and community antibiotic prescribing for England and the UK. **Pg. 8-9**
Figure S3 A to C. Prevalence of *C. difficile* genotypes (as defined by multilocus sequence type) in Oxfordshire and Leeds. **Pg. 10-13**
Figure S4. Incidence of CDI and prescribing of fluoroquinolones and cephalosporins at Leeds teaching hospitals. **Pg. 14**
Figure S5 A to D. Phylogenetic analysis of each genotype. **Pg. 15-18**
Figure S6 A and B. Phylogenies of three prevalent fluoroquinolone susceptible genotypes are similar whether sampled (inter)nationally or regionally. **Pg. 19**
Figure S7. Quinolone prescribing in Leeds, UK by specialty. **Pg. 20**
Figure S8. The incidence of CDI in Oxfordshire for inferred secondary cases **Pg. 21**
Figure S9. Incidence of CDI and hospital prescribing of extended spectrum and beta-lactamase resistant penicillins in Oxfordshire. **Pg. 22**
Table S1. Isolate Collections. **Pg. 23-171**
Table S2. Cross-correlation of antibiotic class with CDI incidence in England, Oxfordshire and Leeds. **Pg. 172**

Supplementary Methods

Changes in Antibiotic Prescribing Policy[1]
A directive aiming to control nosocomial CDIs was introduced in June 2007. All National Health Service Hospital Trusts were required to have antibiotic guidelines restricting the use of broad-spectrum antibiotics. This aimed to reduce the use of fluoroquinolones and extended-spectrum cephalosporins, avoiding empirical or definitive overuse, and ensuring their prescription only when indicated by the clinical condition of the patient or the results of microbial investigation. Antibiotic guidelines were required to limit iv regimens to 48 hours; to provide guidance on iv–oral switch, with automatic stop dates facilitating 48-hour iv and 5-day oral regimens; and to limit most surgical prophylaxis to a single dose. Regular audit of compliance was required.[1]

CDI Incidence Data
In order to characterise overall reductions in CDI, and assess representativeness of Oxfordshire and the Leeds region (from where whole genome sequence (WGS) data was derived, see below) for England in general, national (England) and regional (Oxfordshire and Leeds) CDI surveillance data were obtained from Public Health England. However, from the 1990s to date, CDI surveillance data had been collected in several different ways. From the early 1990s, data on cases were collected on a voluntary basis. Mandatory reporting of cases aged 65 years and older in England was introduced in 2004 and this was extended to include cases aged 2 years and over in 2007.

To account for the fact that mandatory reporting between 2004-2007 did not include cases under 65 years, for this study (Figures 1 and 2) CDI cases for those aged 2 years and over in 2004-2007 were estimated by multiplying the reported over-65 cases in this period by the inverse proportion of over-65 cases out of over-2 cases in 2008 for each region (ie estimated using probability sampling weights).

All incidence data are frequency counts, excluding repeat isolations within 28 days of an index test. They include cases both from the community and hospitals, and therefore there is no single relevant denominator; rather the frequency counts can be considered per relevant population (relatively invariant in terms of total numbers over the study period). All trends in CDI incidence data were estimated and tested using negative binomial regression. We used the estimated probability sampling weights above to adjust the mandatory reporting cases from 2004-2007 that did not include cases under 65 years, and estimated the number of cases that would have been mandatorily reported before 2004 by weighting the observed voluntary reported cases according to the ratio of voluntary: age-adjusted mandatorily reported cases in 2004.

The reports used to obtain the incidence data were as follows:

Mandatory reporting: "*C. difficile* infections: quarterly counts by acute trust and CCG and financial year counts and rates by acute trust and CCG, up to financial year 2013 to 2014", published by Public Health England, available from:
https://www.gov.uk/government/statistics/clostridium-difficile-infection-annual-data "Archived Quarterly counts of *Clostridium difficile* in patients aged 65 years and over (January 2004 – December 2007)", published by Public Health England, available from:
http://webarchive.nationalarchives.gov.uk/20140629102627/http://www.hpa.org.uk/webc/HPAwebFile/HPAweb_C/1239695844315

Over-65s data for 2008 were downloaded from the Healthcare Associated Infections Data Capture System on 18th August 2015.

Voluntary reporting: "Voluntary surveillance of *Clostridium difficile*, England, Wales and Northern Ireland: 2013", published by Public Health England, available from
http://webarchive.nationalarchives.gov.uk/20140629102627/http://www.hpa.org.uk/Topics/InfectiousDiseases/InfectionsAZ/ClostridiumDifficile/EpidemiologicalData/VoluntarySurveillance/.

Antimicrobial Prescribing Data
In order to confirm that the introduction of national policies affected antimicrobial prescribing, national antimicrobial prescribing data were obtained from IMS Health (for hospitals; 2001-2014) and HSCIC (for community) (Health & Social Care Information Centre), and converted to WHO defined daily doses

(http://www.whocc.no/atc_ddd_methodology/purpose_of_the_atc_ddd_system/). Hospital usage covered inpatients, outpatients, genitourinary medicine clinics and other prescribing to ambulatory patients; data were available only for the whole of the UK for calendar years 2001-2004 (not available separately for England alone), and separately for England for calendar years 2005-2012. Data were only available yearly, not by smaller time units. Between 2005-2008, data were only available for 42 common antimicrobials, so overall prescribing in this time period was estimated using the proportion of total antibiotic use represented by these antimicrobials in 2009-2012 (ie estimated using probability sampling weights). Community usage covered all prescriptions dispensed in the community, and data were available for calendar years 1998-2013 for England.

Trends in antibiotic prescribing data were estimated and tested using negative binomial regression. In order to estimate a simple univariable association between antimicrobial prescribing and mandatory reported CDI, analogous to a Spearman rho for two continuous factors, we calculated a bivariate cross-correlation, i.e. the correlation between one series at time t and another series at time t - k as a function of the time t and lag k. Because of differences in the time periods in which (yearly) antibiotic prescribing data were available from different sources (see above), we included only the 8 years from 2005-2012. For each class of antibiotics, and all antibiotics combined, we considered hospital use, community use and hospital plus community use, each with a time lag of 0 (ie same year), -1 and +1, (where +1 means antibiotic use in previous year against CDI in current year), and reported the highest cross-correlation.

Regional antimicrobial prescribing data, for Oxford (Oxford University Hospitals NHS Trust) and Leeds (Leeds Teaching Hospitals NHS Trust), also in terms of defined daily doses, were obtained from the respective Trusts, and covered total hospital Trust usage for both Oxford (2003-2013) and Leeds (2008-2014). Regional community prescribing data were not available. Cross-correlations between hospital antimicrobial prescribing and mandatory reported CDI were calculated as above, except that for the more limited Leeds data, only a time lag of 0 was possible. Fluoroquinolones included ciprofloxacin, levofloxacin, moxifloxacin, norfloxacin, ofloxacin and nalidixic acid.

There are no closed form formulae to calculate 95% confidence intervals for cross-correlations. In order to provide estimates of uncertainty we used a simulation based approach. For each time-series (prescribing for each antibiotic/class, *C. difficile* incidence) we simulated new observations for each calendar year from the observed value (taken as the mean of a negative binomial distribution) with the overdispersion parameter based on the negative binomial regression used to estimate trends over calendar time for significance testing. We then calculated the cross-correlation between the pair of simulated time-series, and repeated this 3000 times. The 95% CI reported in Table S2 are the 2.5th and 97.5th percentiles. Where two observed time-series are very highly correlated (ie cross-correlation close to 1), it is possible for the 97.5th percentile to lie below the observed cross-correlation because any error introduced into the simulation weakens the association between the two time-series. For these small number of simulations (with observed cross-correlation >0.988) we report the upper 95% confidence limit as the maximum of the 97.5th percentile and the observed cross-correlation.

Isolate Collections

The four isolate collections from which the isolates studied were drawn are listed in their entirety in Supplementary Table 1 and described in more detail below in sections (i) to (iv). The isolates used to construct phylogenies are shaded in cream within Supplementary Table 1. *C. difficile* isolate culture and genotyping by MLST[2] and PCR-ribotyping were performed as described previously.[3-5] The notation ST1(027) was adopted to indicate, for example, Sequence Type 1 (PCR-ribotype 027). Not all isolates underwent PCR-ribotyping; PCR-ribotypes known to correspond to STs were as previously described.[3]

(i) Clinical Isolates from Oxfordshire and the Leeds region

Clinical isolates from symptomatic patients were cultured from *C. difficile*-positive stool samples identified by enzyme immunoassay (EIA); (initially the Meridian Premier Toxins A&B Enzyme Immunoassay [Meridian Bioscience Europe, Milan, Italy], until 1 April 2012 and subsequently the TechLab Tox A/B II assay [TechLab Inc, Blacksburg, VA, USA]), at the Clinical Microbiology Laboratory, Oxford University Hospitals NHS Trust, Oxford, or by cytotoxin testing at the Leeds Teaching Hospitals NHS Trust, Leeds. Oxfordshire clinical isolates from unique patients (n = 2,021) were first cultured between September 12 2006, and August 19 2013, and Leeds clinical isolates (n = 1,020) between August 2 2010, and May 1 2013. During this time, the number of EIA-positive or cytotoxin positive samples was almost identical to the mandatory reported cases in Oxfordshire and Leeds respectively; however, the number that were culture-confirmed was slightly smaller as expected.

Of note, before 2012, practice in Oxfordshire was to send three stool samples for testing due to concerns about the sensitivity of the assay. Further, proportions of highly genetically similar CDI cases were similar in Oxfordshire and Leeds, despite different assays being used.[6,7]

(ii) Oxfordshire clinical EIA-negative isolates
A total of 395 Oxfordshire clinical isolates were cultured from EIA-negative, but GDH-positive (by the Premier *C. difficile* GDH EIA, Meridian Bioscience Europe, Milan, Italy) stool samples between 3 December 2010 and April 5 2013 (a subset were described previously[8]). Of these, 121 were ST2 (n=28), ST6 (n=26), ST7 (n=37), ST8 (n=28) and ST37 (n=2); these were included in phylogenetic analyses.

(iii) Oxfordshire Infant (Non-Clinical) isolates
A total of 200 isolates were cultured from the stools of Oxfordshire infants between October 30 2008 and July 12 2013 (a subset were described previously[9]). Of these, 80 representing the genotypes ST2 (n=35), ST6 (n=15), ST8 (n=9) and ST37 (n=21) were used in phylogenetic analyses.

(iv) North American and European Clinical Isolates
North American and European isolates (n=803) cultured between 9 May 2006 and 27 November 2009 were available from two pivotal clinical trials of the drug fidaxomicin.[10,11] Isolates from Oxfordshire were excluded to avoid duplication with the Oxfordshire collection described above. Isolates (n=132), representing three locations that were most intensively sampled (Montreal and Calgary, Canada and three cities in Northern Italy) were included in detailed phylogenetic analyses. These were ST1 (n=70, Montreal), ST2 (n=9 Montreal; n=8 Calgary), ST3 (n=12 Calgary), ST8 (n=10 Montreal and n=10 Calgary) and ST17 (n=13, Northern Italy; comprising n=8 Modena, n=3 Arsizio, n= 2 Torino).

Irrespective of their isolation source or the testing methods used, isolates were referred to as non-toxigenic if the toxin-encoding pathogenicity locus (PaLoc) sequences[12] were absent from the genome. Non-toxigenic isolates could be isolated from a toxin positive stool if a mixed infection involving a PaLoc-positive isolate was present or a false positive toxin assay result occurred.

Fluoroquinolone Susceptibility Testing
Fluoroquinolone susceptibility testing (moxifloxacin MIC) was performed on a subset of the North American and European isolates (n=387) (Figure S1) by the agar dilution method. Doubling dilutions of 1-32 mg/L were used as described in the CLSI M11-A8 document[13]. If growth occurred on the 32 mg/ml plate, the MIC was reported as >32 mg/L.

Genome Sequencing
Genomes were sequenced as described previously[5,14] using Illumina sequencing by synthesis technology.[15] Velvet de novo assemblies were made.[16] VelvetOptimiser 2.1.7 (with Velvet 1.0.7–1.0.18) was run to find the optimal Kmer size (k) for each sample and the N50 (length of the smallest contig such that all contigs of that length or less form half of the final assembly), as well as the expected coverage (average kmer coverage of contigs) and coverage cutoff (kmer coverage threshold). Reference-based assemblies used to build the phylogenies were made as described[6] using the *C. difficile* 630 reference genome (GenBank AM180355.1).

Statistical Analysis of *C. difficile* Incidence
National CDI incidence reporting became mandatory in patients aged over 2 years during 2007. Between 2004-2007, mandatory reporting included only those aged over 65, so incidence in over-2s during 2004-2007 was estimated using the proportion of over-65s reported in 2008 (ie estimated using probability sampling weights). In Oxfordshire, samples submitted for *C. difficile* testing could have several different tests performed and hence different results available: GDH (from 1 April 2012), EIA (on only GDH-positive samples after 1 April 2012, previously on all samples), culture (on toxin-positive samples throughout, plus GDH-positive toxin-negative samples from 1 April 2012, and all samples in a diagnostic study[8]) and WGS. Because of recognised limitations in the Premier EIA test leading to a moderate number of toxin-positive samples before April 2012 not being confirmed on culture[8], monthly CDI incidence in Oxfordshire (from September 1 2006 to Feb 26 2013) was calculated from toxin-positive culture-positive samples, using probability weights to adjust for toxin-positive samples not collected for culture (approximately 30% in 2007-2008 due to limited personnel, subsequently <5%). Repeated positives with the same ST within 28 days of an index test were excluded (assuming non-typed toxin-positive isolates within 28 days of a typed isolate were the same ST). Probability weights were also used to adjust for small numbers of de-duplicated culture-positive samples where ST and fluoroquinolone resistance genotype was not available. If there were no known resistance genotypes for a particular ST in a particular month, the resistance genotype was denoted 'unknown'. Incidence rates were calculated using negative binomial

regression with probability weights above. We did not include auto-correlation because previous studies have shown it to be very low (from[17] overall phi=0.029 (95% CI 0.009-0.049); teaching hospitals 0.104 (95% CI 0.048-0.159). Rates were calculated dividing the single outcome (*C. difficile* case) according to a number of different characteristics, analogous to analyses of different causes of death. First we considered incidence of CDI caused by all fluoroquinolone-resistant vs fluoroquinolone-sensitive isolates. As the hypervirulent ST1(027) comprised 526 (62%) of the 855 fluoroquinolone-resistant cases we then considered it separately from fluoroquinolone-resistant vs fluoroquinolone-sensitive non-ST1 isolates. Lastly, we calculated incidence separately by fluoroquinolone genotype for STs with >10% resistance. Rates were also calculated separately for cases that could plausibly have arisen from secondary spread (transmission) inferred by closely genetic relationships to prior cases (≤2 single nucleotide variants (SNVs) from the original case), separately for fluoroquinolone-susceptible and resistant isolates.

Overall rates are presented since models allowing trend changes with either piecewise linear or natural cubic splines did not improve model fit materially. Model fit was assessed using Pearson's chi-squared test for models without overdispersion, and using Andrews' specification test[18] for models with overdispersion. Of 43 trends calculated, 40 (93%) showed satisfactory goodness of fit (p>0.05) compatible with chance. The 3 models with p<0.05 were ST12 (p<0.0001), fluoroquinolone-resistant isolates within ST35 (p<0.0001) and fluoroquinolone-sensitive isolates within All Non-ST1 genotypes with >10% fluoroquinolone-resistance (p=0.032). We compared incidence trends according to the different classifications of the single outcome using stacked regression [19] with robust standard errors.

In Leeds, where the gold-standard reference assay was used and WGS was only available from 2010, incidence was based on mandatory reporting, and fluoroquinolone resistance estimated from the proportion of isolates submitted to CDRN for ribotyping which came from genotypes that are >90% resistant (106/027/001/017). (Leeds samples which were sent for WGS between 2010-2013 confirmed similar rates of resistance genotypically.)

Phylogenetic Analyses

Initial phylogenetic trees were built using the maximum likelihood approach implemented in phyml version 3.1.[20] The trees were then corrected to account for recombination events using ClonalFrameML version 1.20.[21] The nodes of the trees were dated using the previously estimated *C. difficile* evolutionary rate of 1.4 mutation per year per genome with confidence interval 0.6-2.3.[14] The main period of particular interest from 1990 to 2015 was allocated most horizontal space in graphical tree representations by compressing the period pre-1990, making trees directly comparable post-1990. Time prior to 1990 was not shown since dating older nodes using a short-term evolutionary rate is problematic due to the time-dependency of evolutionary rates.[22] Graphical representations of all trees were made using FigTree version 1.4.2, which is available from http://tree.bio.ed.ac.uk/software/figtree/. In each tree, the Evolutionary Distinctiveness (ED) score of each isolate was calculated,[22] equal to the sum, for all branches on the path from the root to the leaf (isolate), of the length of the branch divided by the number of leaves it supports.[23] For a given isolate, a low ED score therefore indicates the presence of close relatives in the tree, whereas a high ED score indicates their relative absence. Outliers included in phylogenies to obtain pre-1990 roots were excluded from ED analysis.

For the two most numerous fluoroquinolone resistant (ST1(027)) and susceptible (ST8(002)) genotypes, Oxfordshire isolates representing every third and second isolate respectively by date were included (Figure 4C, D). The closely related genotypes ST10(015) and ST44(015) and also ST13, ST14 and ST49(129/014) were combined before phylogenies were constructed (Figure S5B). Additional genomes from international cities/regions where ≥8 isolates from an ST were available from fidaxomicin clinical trials[10,11] (suggesting they were reasonably representative) were included.

References

1. *Clostridium difficile* infection: How to deal with the problem. Public Health England and the Department of Health, 2008. http://www.hpa.org.uk/webc/HPAwebFile/HPAweb_C/1232006607827. Accessed February 16th, 2016.
2. Griffiths D, Fawley W, Kachrimanidou M, et al. Multilocus sequence typing of *Clostridium difficile*. *J Clin Microbiol* 2010; **48**: 770–8.
3. Dingle KE, Griffiths D, Didelot X, et al. Clinical *Clostridium difficile*: clonality and pathogenicity locus diversity. *PLoS One* 2011; **6**: e19993.
4. Dingle KE, Didelot X, Ansari MA, et al. Recombinational switching of the *Clostridium difficile* S-layer and a novel glycosylation gene cluster revealed by large-scale whole-genome sequencing. *J Infect Dis* 2013; **207**: 675–86.
5. Dingle KE, Elliott B, Robinson E, et al. Evolutionary history of the *Clostridium difficile* pathogenicity locus. *Genome Biol Evol* 2014; **6**: 36–52.
6. Eyre DW, Cule ML, Wilson DJ, et al. Diverse sources of *C. difficile* infection identified on whole-genome sequencing. *N Engl J Med* 2013; **369**: 1195–205.
7. Martin J, Eyre DW, Fawley WN, Walker AS, Crook DW, Wilcox MH. 2016 *C. difficile* (CD) ribotypes exhibit variable patient-to-patient transmission rates, as determined by whole-genome sequencing (WGS), suggesting differing reservoirs and modes of acquisition. Abstract O557: ECCMID 2016 Amsterdam 9-12 April 2016.
8. Planche TD, Davies KA, Coen PG, et al. Differences in outcome according to *Clostridium difficile* testing method: a prospective multicentre diagnostic validation study of *C. difficile* infection. *Lancet Infect Dis* 2013; **13**: 936–45.
9. Stoesser N, Crook DW, Fung R, et al. Molecular epidemiology of *Clostridium difficile* strains in children compared with that of strains circulating in adults with *Clostridium difficile*-associated infection. *J Clin Microbiol* 2011; **49**: 3994–6.
10. Cornely OA, Crook DW, Esposito R, et al. Fidaxomicin versus vancomycin for infection with *Clostridium difficile* in Europe, Canada, and the USA: a double-blind, non-inferiority, randomised controlled trial. *Lancet Infect Dis* 2012; **12**: 281–9.
11. Louie TJ, Miller MA, Mullane KM, et al. Fidaxomicin versus vancomycin for *Clostridium difficile* infection. *N Engl J Med* 2011; **364**: 422–431.
12. Braun V, Hundsberger T, Leukel P, Sauerborn M, Von EichelStreiber C. Definition of the single integration site of the pathogenicity locus in *Clostridium difficile*. *Gene* 1996; **27**: 29–38.
13. Wayne P.A. CLSI Methods for antimicrobial susceptibility testing of anaerobic bacteria: approved standard-eighth edition. CLSI document M11-A8. *Clinical and Laboratory Standards Institute* 2012.
14. Didelot X, Eyre DW, Cule M, et al. Microevolutionary analysis of *Clostridium difficile* genomes to investigate transmission. *Genome Biol* 2012; **13**: R118.
15. Bentley DR, Balasubramanian S, Swerdlow HP, et al. Accurate whole human genome sequencing using reversible terminator chemistry. *Nature* 2008; **456**: 53–9.
16. Zerbino DR, Birney E. Velvet: algorithms for de-novo short read assembly using de Bruijn graphs. *Genome Res* 2008; **18**: 821–9.
17. van Kleef E, Gasparrini A, Guy R, et al. Nosocomial transmission of *C. difficile* in English hospitals from patients with symptomatic infection. *PLoS One* 2014; **16**: e99860.
18. Cameron AC, and Trivedi PK. Regression Analysis of Count Data. 2nd ed. Cambridge: Cambridge University Press.
19. Lunn M, McNeil D. Applying Cox regression to competing risks. Biometrics 1995; **51**: 524–32.
20. Guindon S, Dufayard J-F, Lefort V, Anisimova M, Hordijk W, Gascuel O. New algorithms and methods to estimate maximum-likelihood phylogenies: assessing the performance of PhyML 3.0. *Syst Biol* 2010; **59**: 307–21.
21. Didelot X, Wilson DJ. ClonalFrameML: Efficient inference of recombination in whole bacterial genomes. *PLoS Comput Biol* 2015; **11**: e1004041.
22. Biek R, Pybus OG, Lloyd-Smith JO, Didelot X. Measurably evolving pathogens in the genomic era. *Trends Ecol Evol* 2015; **30**: 306–13.
23. Isaac NJB, Turvey ST, Collen B, Waterman C, Baillie JEM. Mammals on the EDGE: Conservation priorities based on threat and phylogeny. *PLoS One* 2007; **2**: e296.

Figure S1. Resistance to moxifloxacin in *C. difficile* can be accurately predicted by the presence of the non-synonymous mutations: *gyrA* C(245)T [T(82)I], or *gyrB* G(1276)A, [D(426)N].

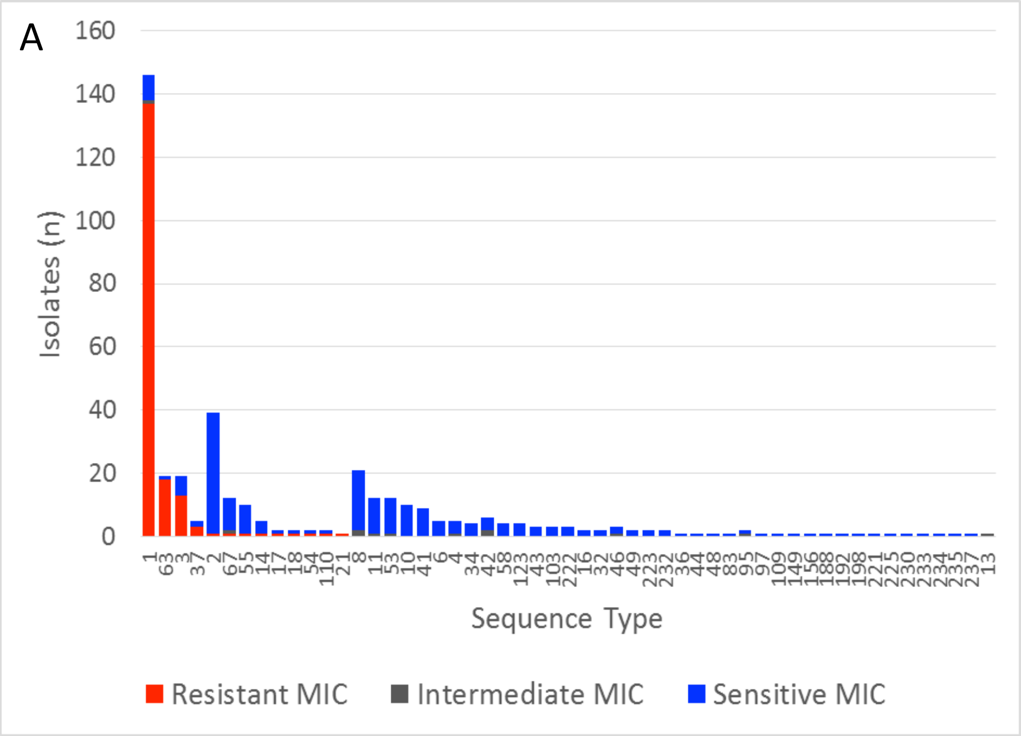

(A) *C. difficile* moxifloxacin susceptibility (MIC) by MLST genotype.
Distribution of 387 isolates undergoing moxifloxacin susceptibility testing, among 53 STs. Stacked bars indicate the proportion each ST phenotypically resistant (MIC ≥8 mg/L), intermediate (MIC 4mg/L) or susceptible (MIC ≤2mg/L).

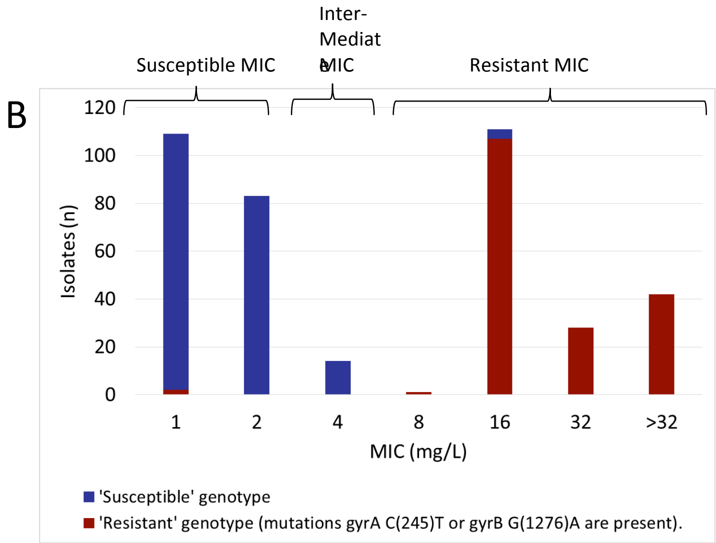

(B) Presence of mutations *gyrA* C(245)T or *gyrB* G(1276)A) confers phenotypic resistance (MIC ≥8 mg/L) to moxifloxacin. Distribution of the 387 isolates, scored as wild type or 'mutant' according to the presence/absence of these mutations, by MIC. There was 98.7% agreement between presence of the above mutations and intermediate or resistant (≥ 4 mg/L) phenotype (sensitivity 97.8%, specificity 99.5%). No grey 'intermediate' is shown because genotype can only be 'resistant' or 'susceptible'; red (resistant) and blue (susceptible) indicate genotype.

Figure S2, parts A to F. National hospital and community antibiotic prescribing for England and the UK.

(A) Cephalosporin prescribing (total and by generations 1 to 3).

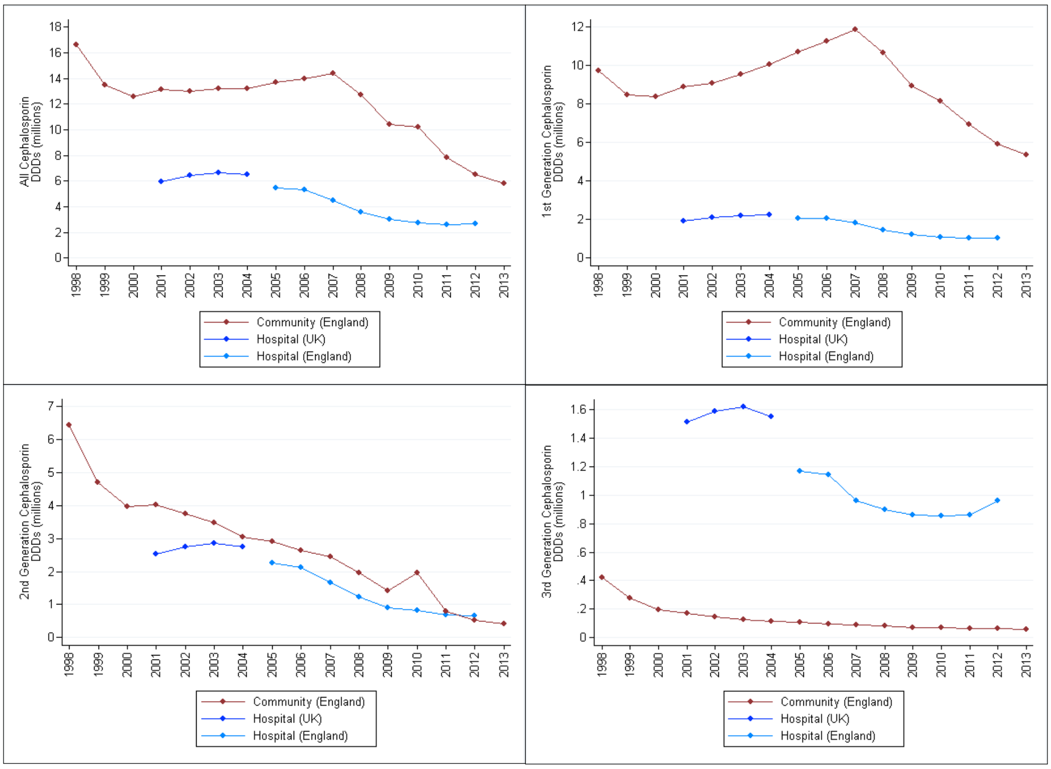

(B) Penicillin-betalactamase combination prescribing (total comprising co-amoxiclav and piperacillin/tazobactam prescribing (shown individually below), plus ticarcillin/clavulanic acid and ampicillin/flucloxacillin (not shown individually).

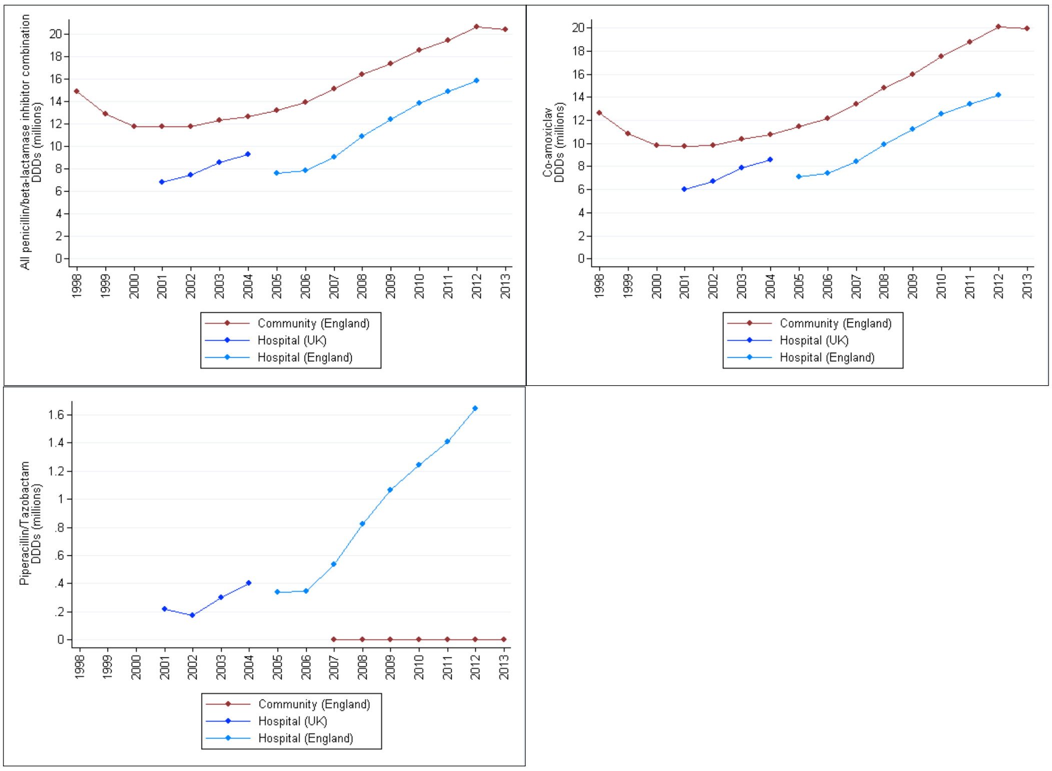

Figure S2, continued. National hospital and community antibiotic prescribing for England and the UK.

(C) Beta-lactamase resistant penicillin prescribing

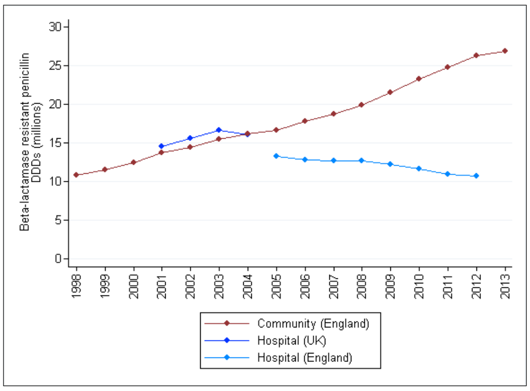

(D) Extended spectrum penicillin prescribing

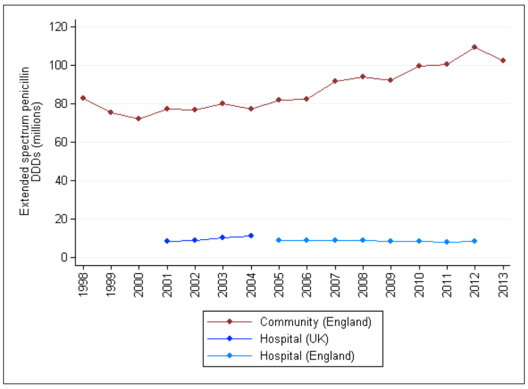

(E) Carbapenem prescribing

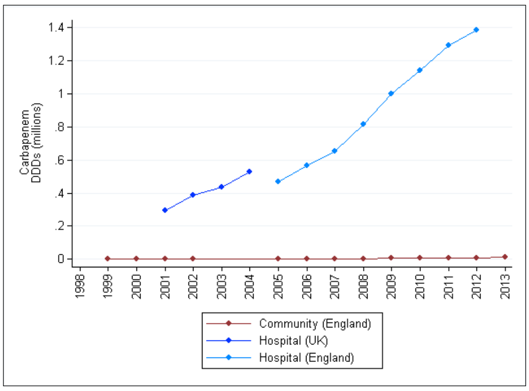

(F) Lincosamide (Clindamycin) prescribing

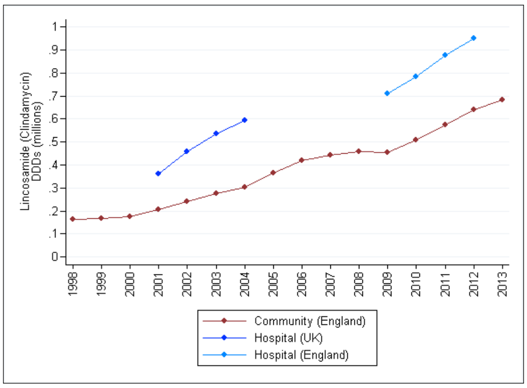

Figure S3. Prevalence of *C. difficile* genotypes (defined by multilocus sequence type, ST) in Oxfordshire and Leeds.

(A) Data used to inform the choice of genotypes for which phylogenies were built representing Oxfordshire and Leeds (Figure S5). ST36 (adjacent to dotted line), with a total of 47 isolates, was chosen as an arbitrary cut off below which phylogenies were not shown due to relatively small numbers of isolates. ST incidence graphs were built for all genotypes containing >10 isolates. The x axis indicates (from top) Sequence Type (ST), then the number of Leeds clinical isolates and Oxford clinical isolates belonging to each ST.

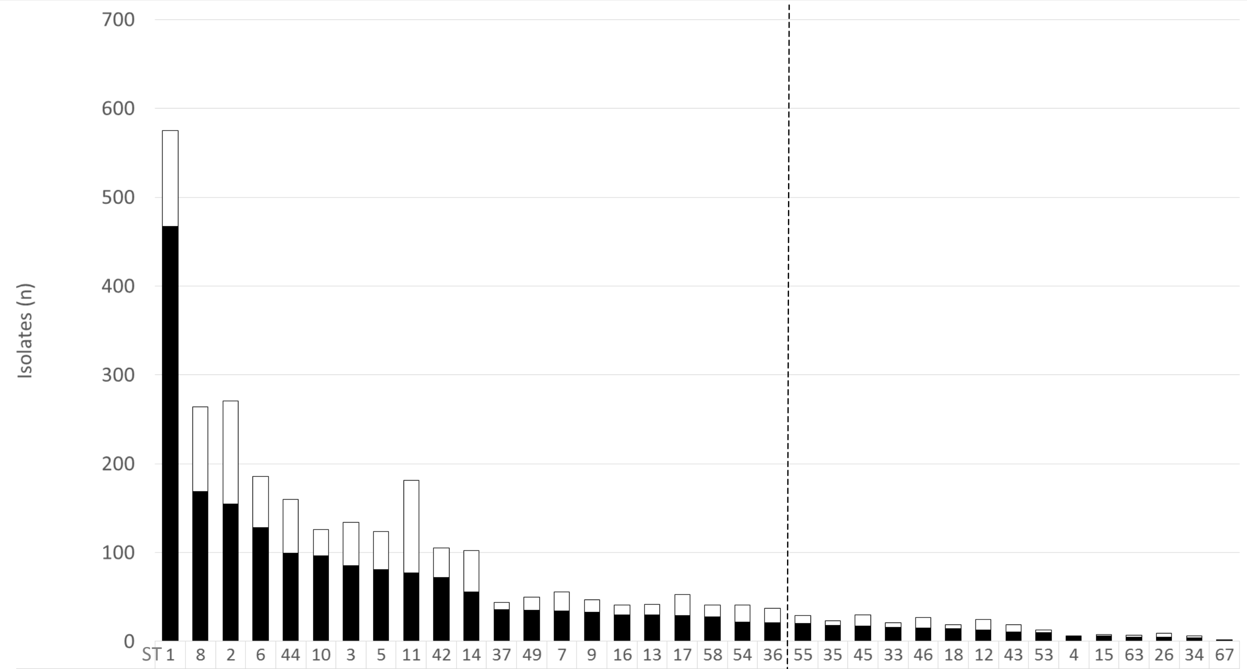

ST	1	8	2	6	44	10	3	5	11	42	14	37	49	7	9	16	13	17	58	54	36
□ Leeds Clinical	108	95	116	58	61	30	49	43	104	33	46	8	15	22	14	11	12	24	13	19	16
■ Oxford Clinical	467	169	155	128	99	96	85	81	77	72	56	36	35	34	33	30	30	29	28	22	21

ST	55	35	45	33	46	18	12	43	53	4	15	63	26	34	67
□ Leeds Clinical	9	5	13	5	12	5	12	8	3		2	2	4	2	
■ Oxford Clinical	20	18	17	16	15	14	13	11	10	6	6	5	5	4	2

Figure S3 continued. Prevalence of *C. difficile* genotypes (defined by multilocus sequence type, ST) in Oxfordshire and Leeds.

(B) Incidence of genotypes containing >10% fluoroquinolone resistant isolates in Oxfordshire (EIA positive clinical isolates only). IRR=Annual incidence rate ratio. Black bars denote samples with unknown fluoroquinolone susceptibility.

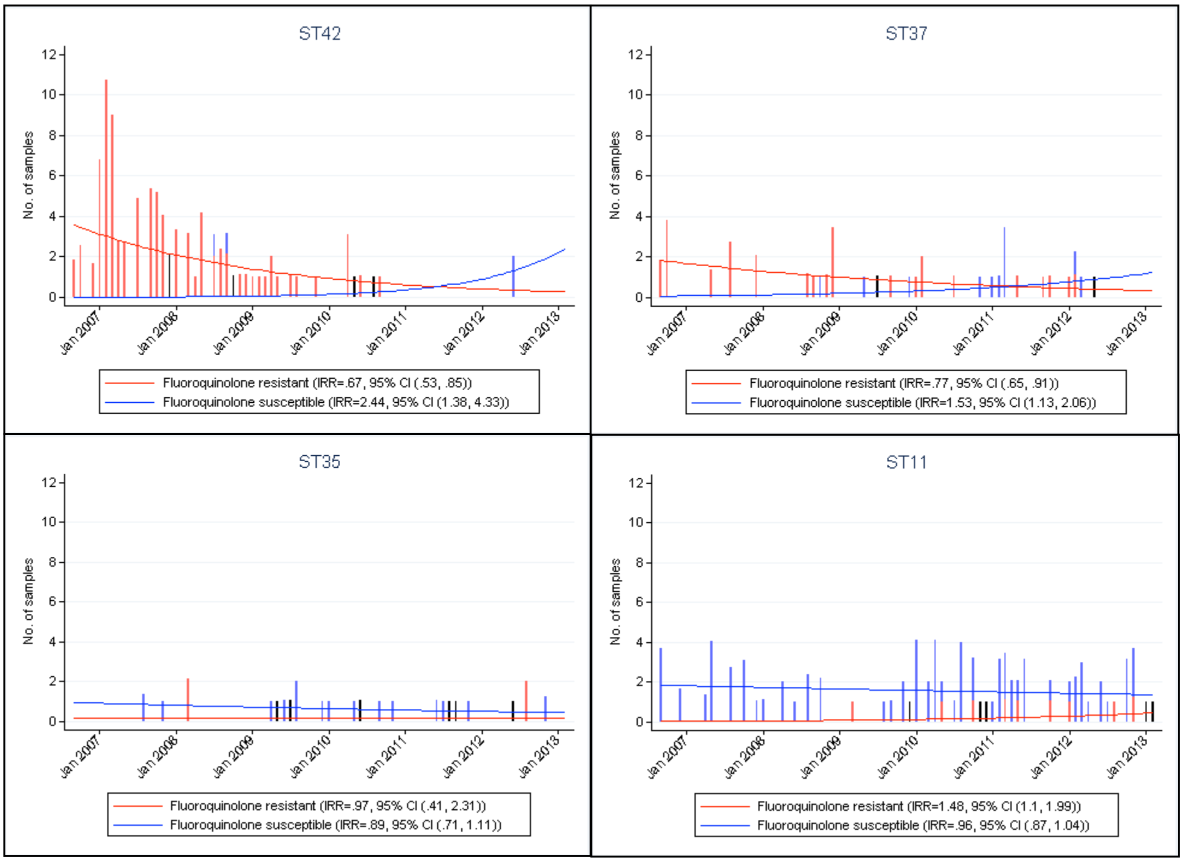

Figure S3 continued. Prevalence of *C. difficile* genotypes (defined by multilocus sequence type) in Oxfordshire and Leeds.

(C) Incidence of genotypes lacking or containing only sporadic fluoroquinolone resistant isolates in Oxfordshire (EIA positive clinical isolates only). IRR=Annual incidence rate ratio. Black bars denote samples with unknown fluoroquinolone susceptibility.

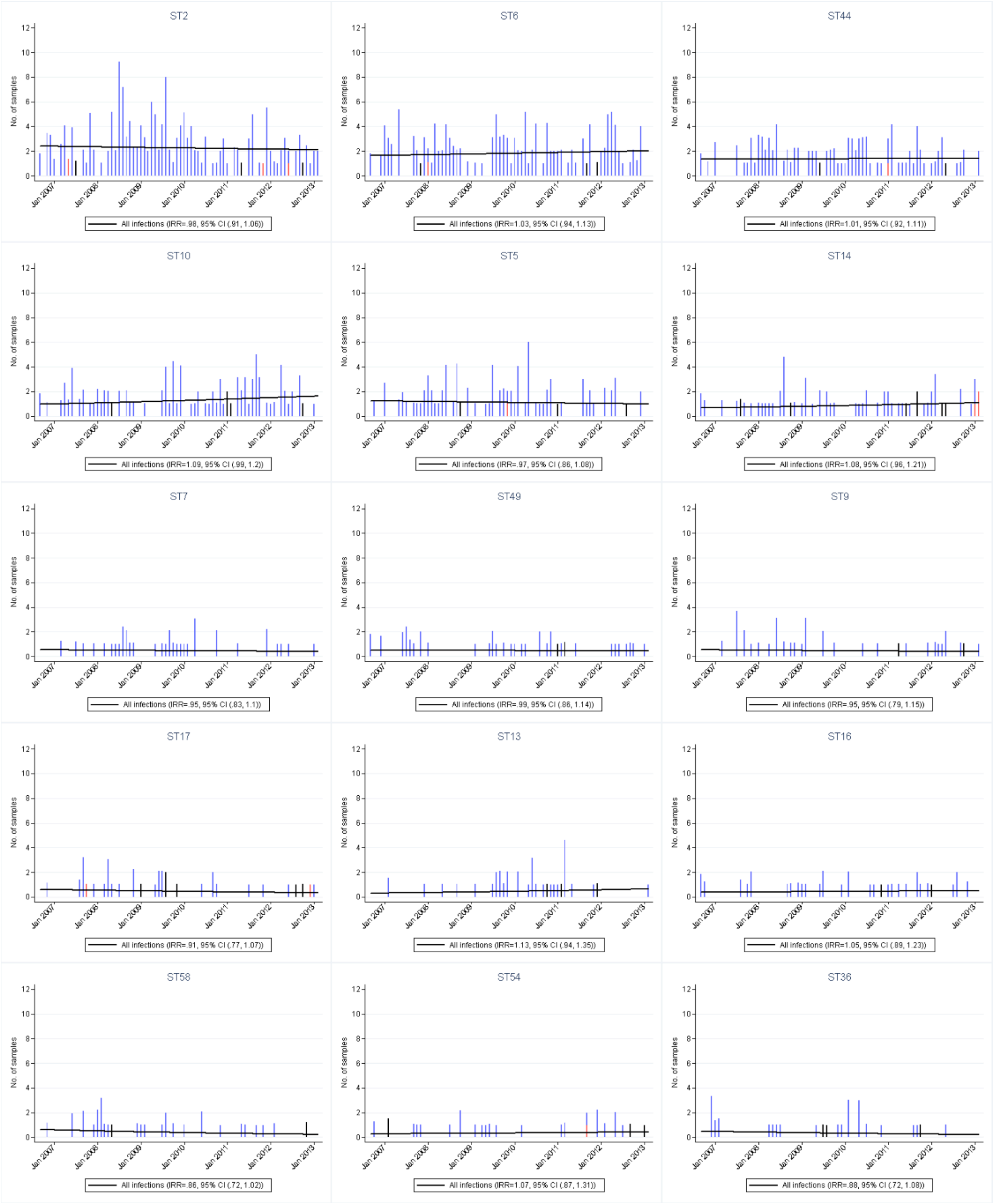

Figure S3 continued. Prevalence of *C. difficile* genotypes (defined by multilocus sequence type) in Oxfordshire and Leeds.

(C) continued

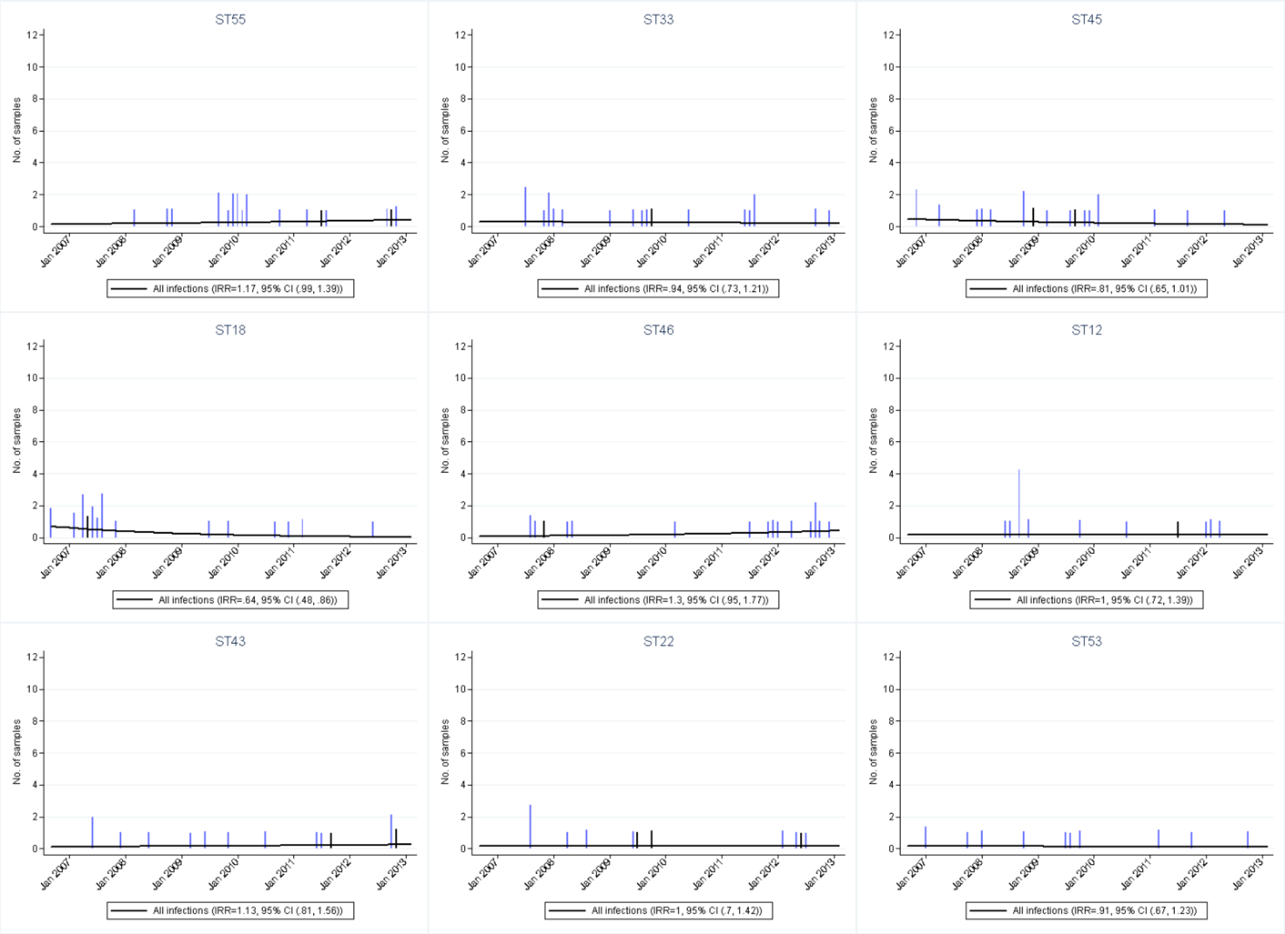

(D) Incidence of genotypes excluding ST1(027) in Oxfordshire (EIA positive clinical isolates only). IRR=Annual incidence rate ratio. Black bars denote samples with unknown fluoroquinolone susceptibility.

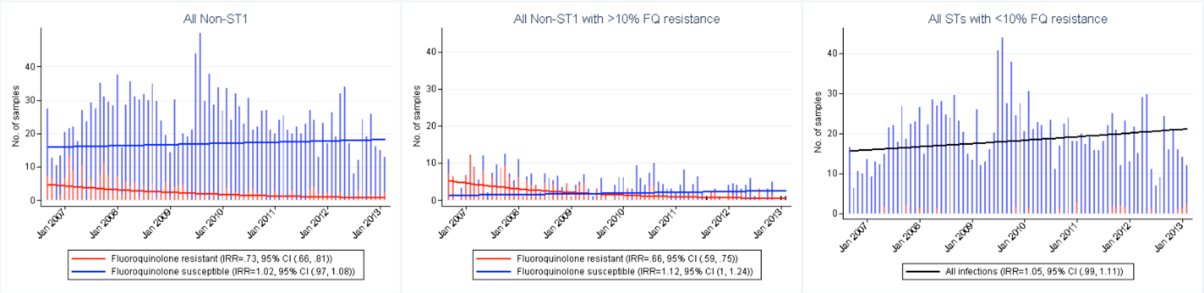

Figure S4. Incidence of CDI together with prescribing of fluoroquinolones and cephalosporins at Leeds teaching hospitals.

The proportion of fluoroquinolone resistance in a given year was based on all isolates submitted to the *C. difficile* Ribotyping Network (resistant genotypes were assumed to be PCR-ribotypes 027, 001, 106 and 017). Data were based on financial years (Apr-Mar).

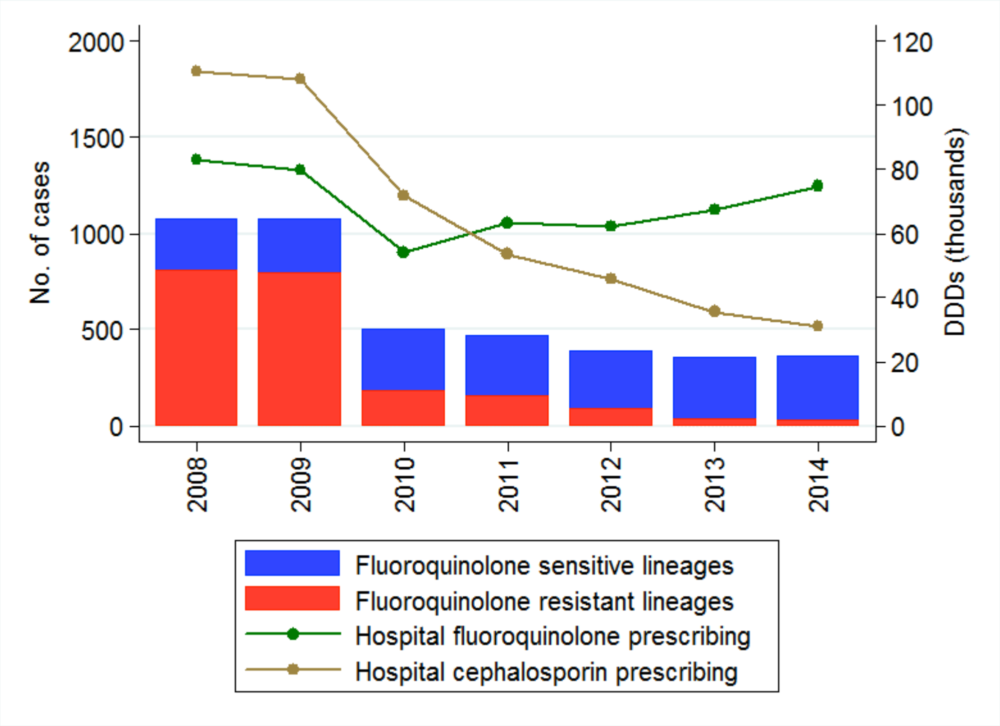

Figure S5. Phylogenetic analysis of each genotype.

(A) Phylogenies of four genotypes containing fluoroquinolone resistant regions. Phylogenies were scaled to be directly comparable post-1990; the grey shaded region prior to 1990 represents the region that should not be compared.

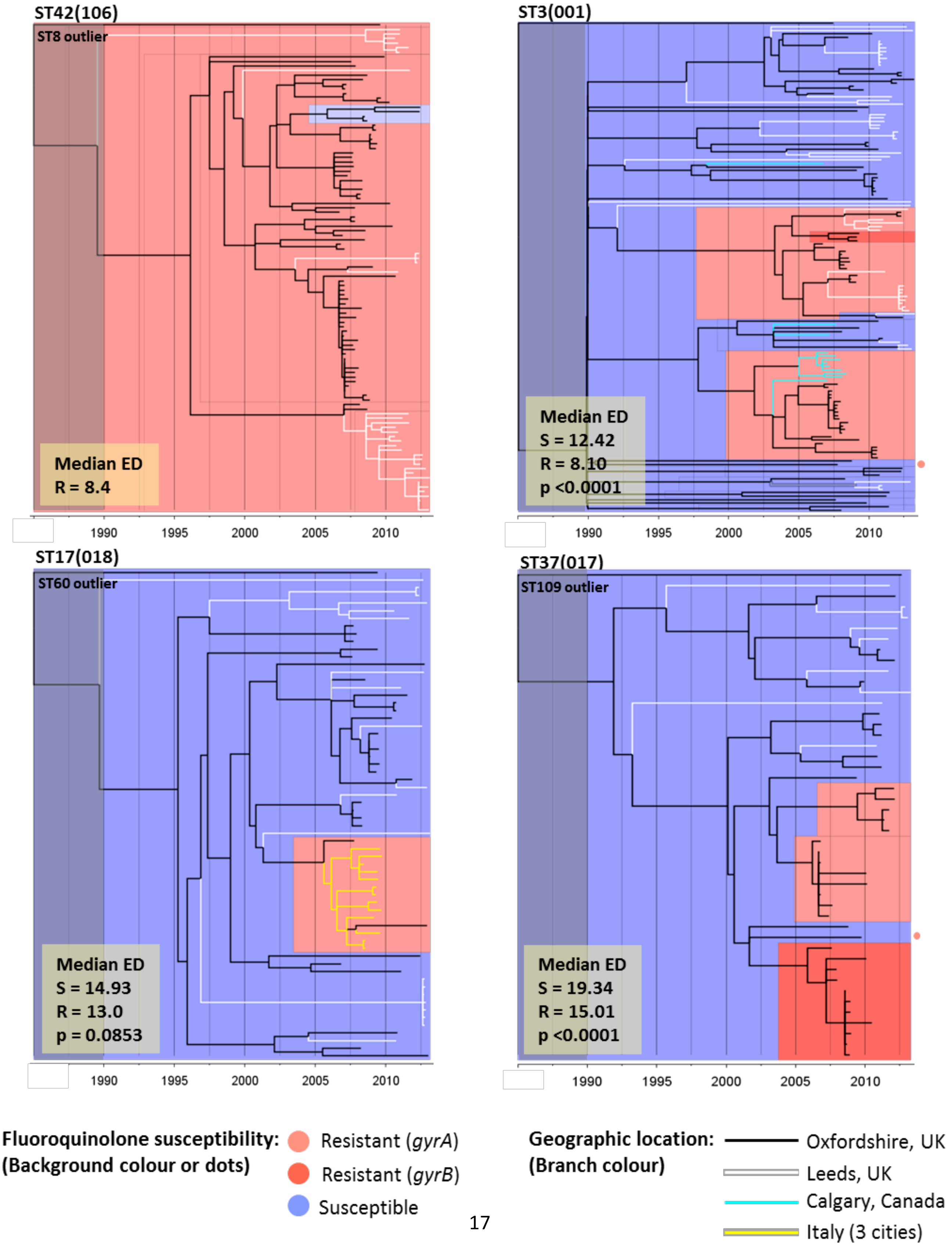

Figure S5 continued. **Phylogenetic analysis of each genotype.**

(B) Phylogenies of six prevalent genotypes lacking, or containing only sporadic fluoroquinolone resistance. Phylogenies were scaled to be directly comparable post-1990; the grey shaded region prior to 1990 should not be compared.

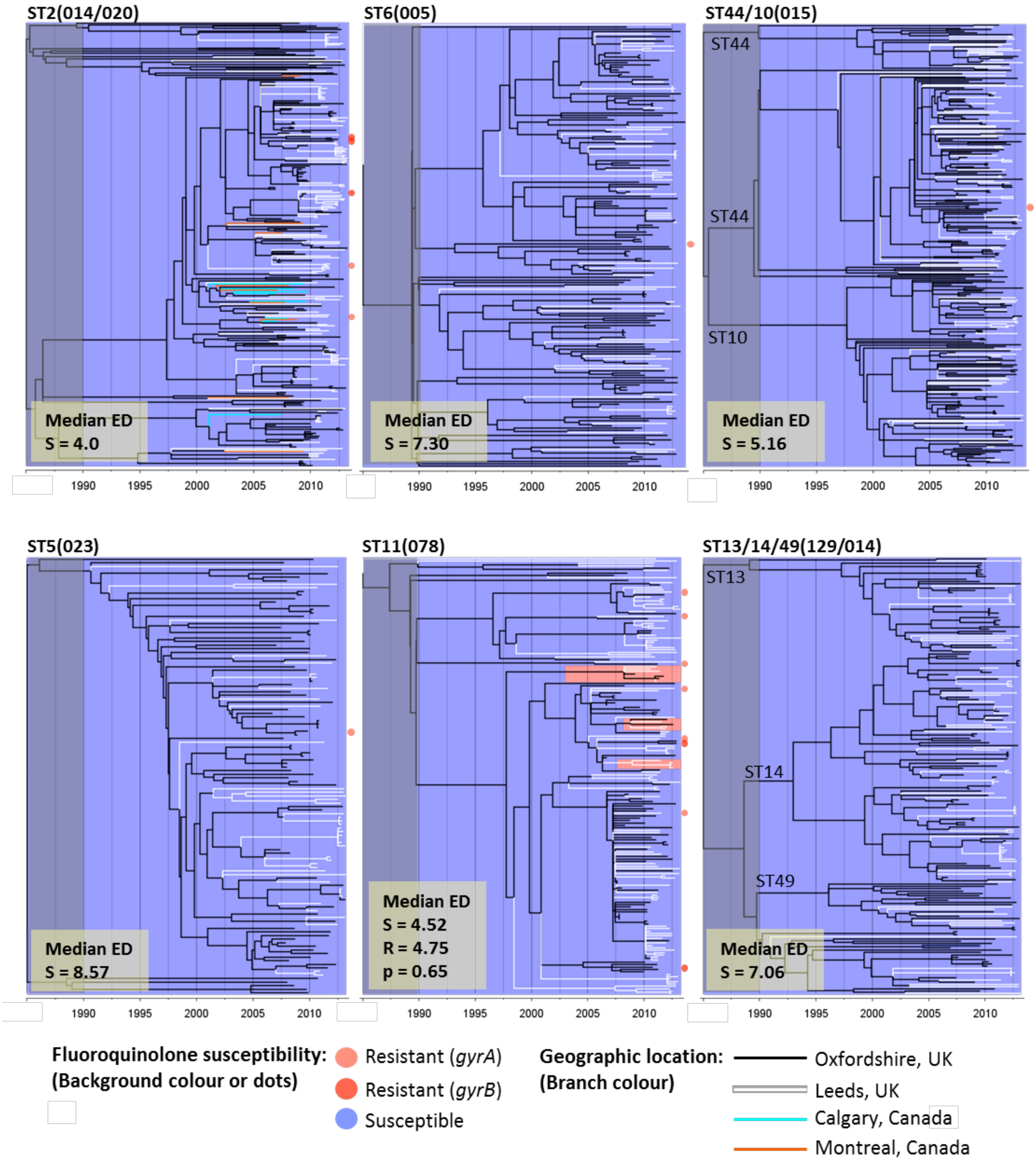

Figure S5 continued. Phylogenetic analysis of each genotype.

(C) Phylogenies of six genotypes lacking or showing only sporadic instances of fluoroquinolone resistance. Phylogenies were scaled to be directly comparable post-1990; the grey shaded region prior to 1990 should not be compared.

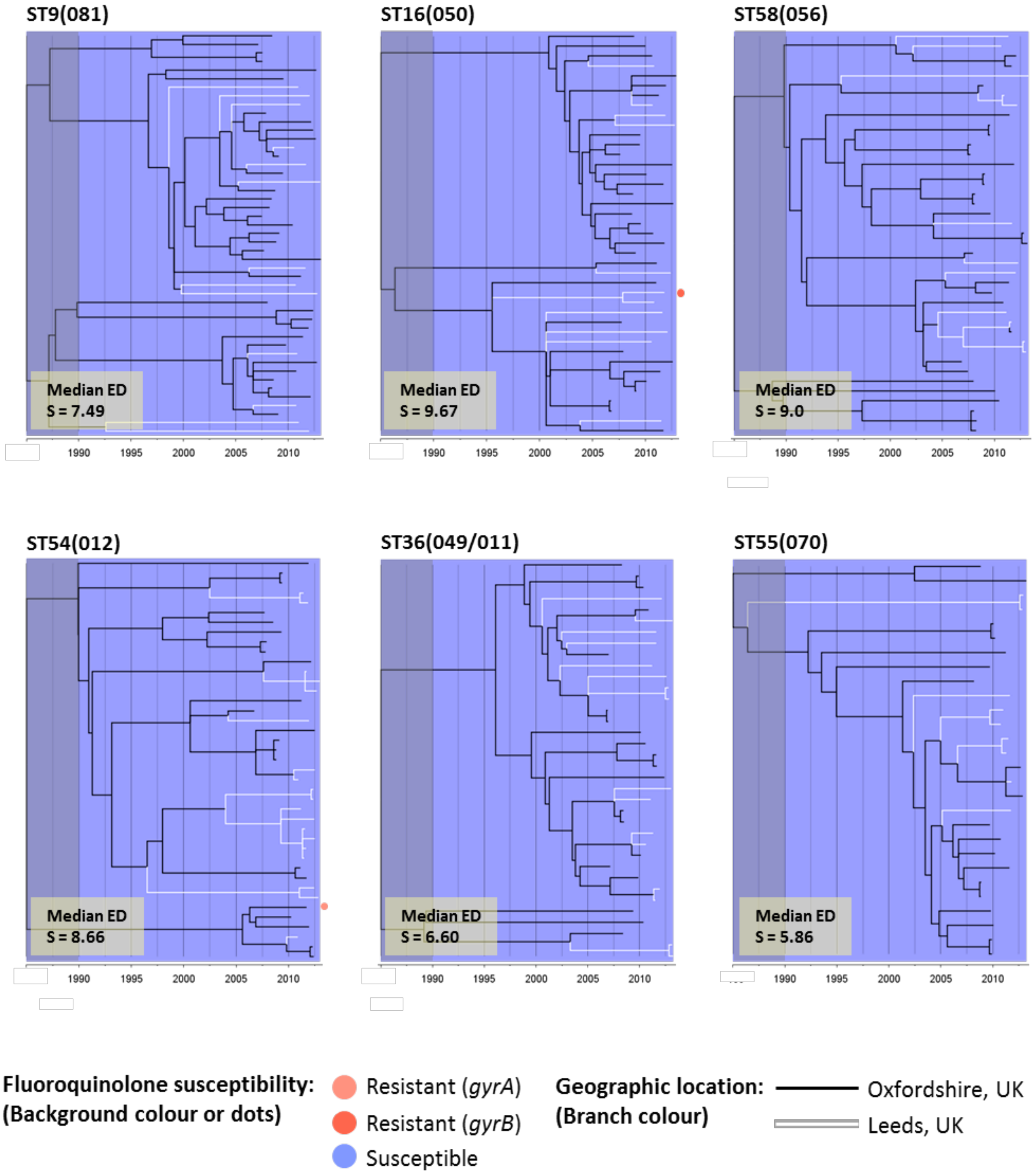

Figure S5 continued. Phylogenetic analysis of each genotype.

(D) Phylogeny of fluoroquinolone susceptible ST7(026), containing both toxigenic (lower region of tree) and non-toxigenic (upper region) isolates. Phylogenies were scaled to be directly comparable post-1990; the grey shaded region prior to 1990 should not be compared.

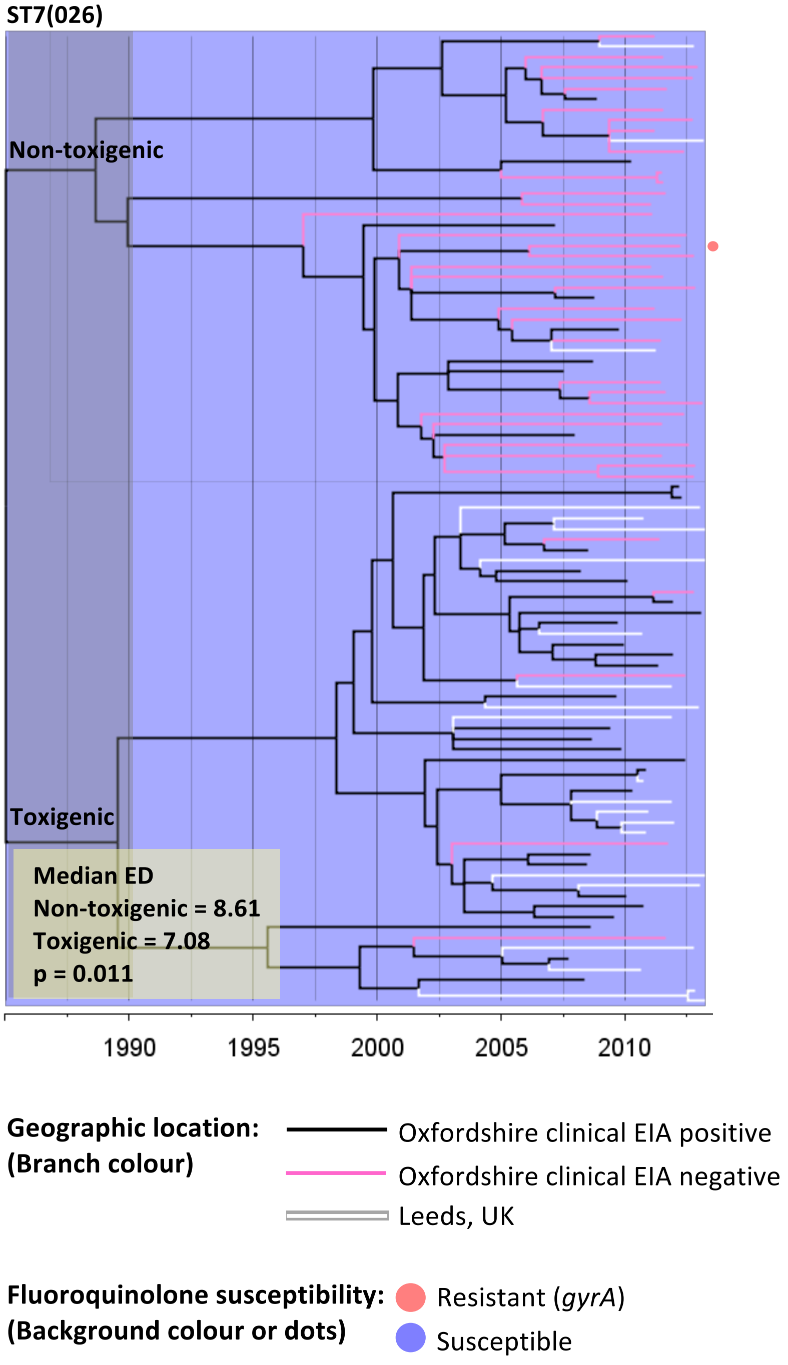

Figure S6. Phylogenies of three prevalent fluoroquinolone susceptible genotypes are similar with respect to branch length whether sampled (A) (inter)nationally (upper panel) or (B) regionally (lower panel).

Phylogenies representing the same genotype (ST) are arranged vertically above each other in the upper and lower panels, to facilitate comparison. For ST8(002) every second Oxfordshire isolate by date was included in the upper panel, whereas all available were included in the lower panel. Phylogenies are directly comparable post-1990.

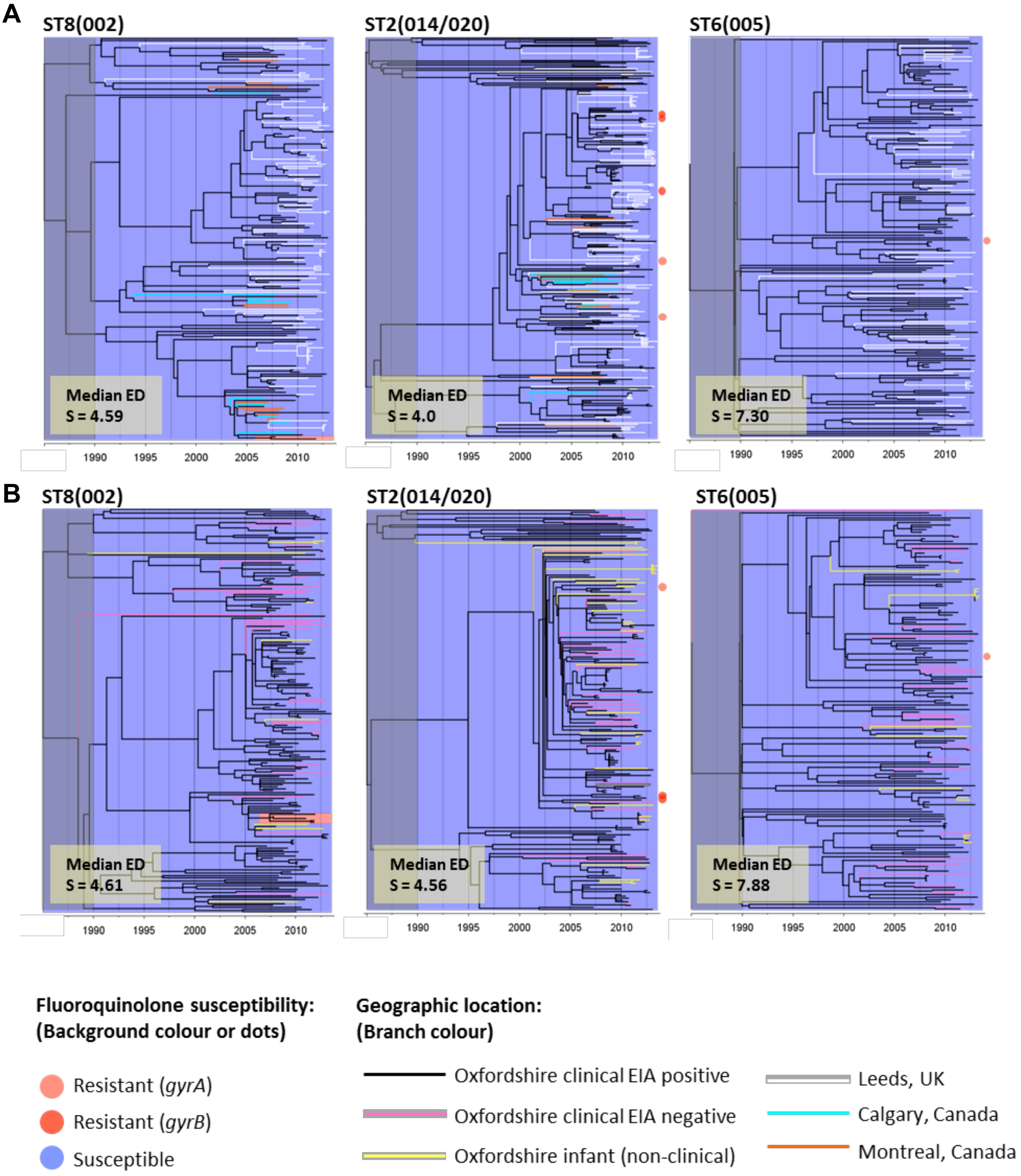

Figure S7. Quinolone prescribing in Leeds, UK by specialty.
Trend lines represent the three month average.

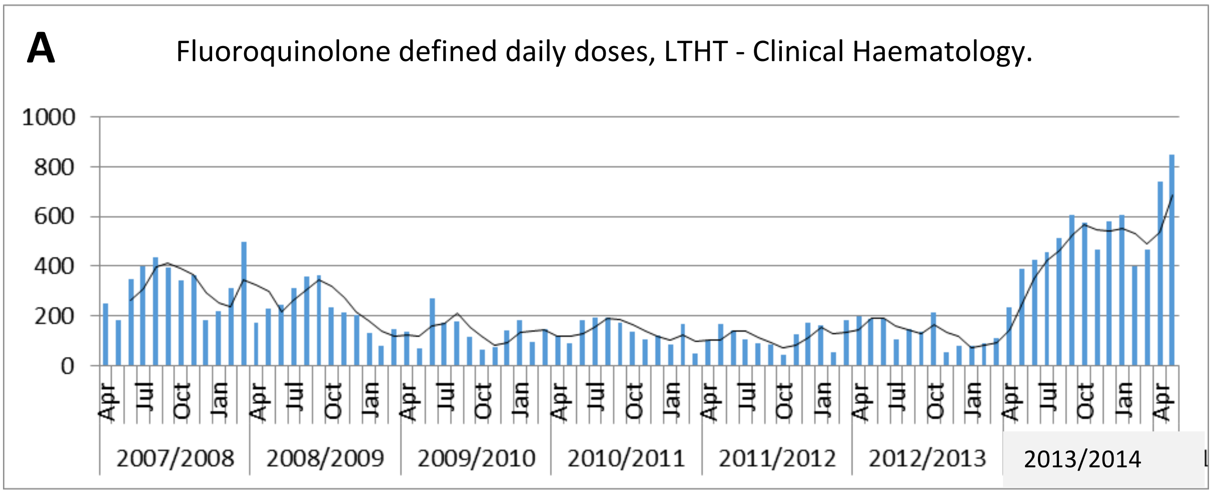

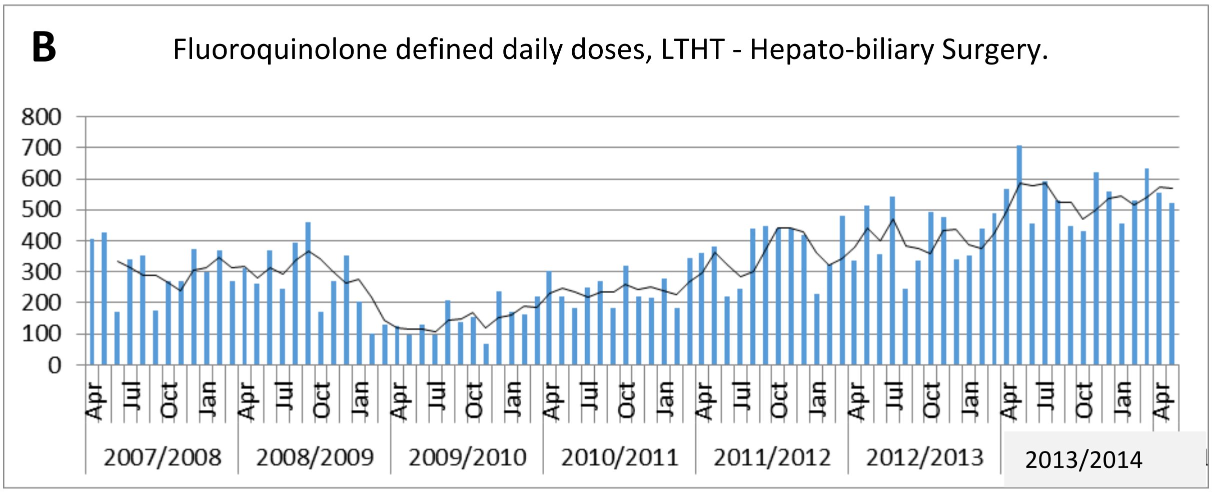

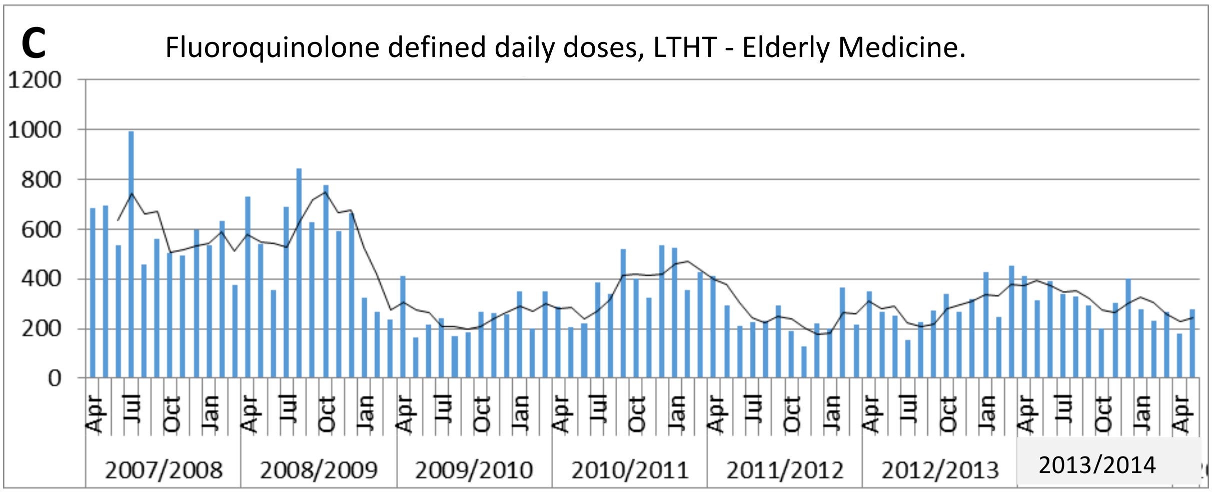

Figure S8. The incidence of CDI in Oxfordshire for inferred secondary cases. i.e. subsequent cases caused by *C. difficile* isolates that are genetically closely related (≤2 single nucleotide variants) to the original case, assessed separately for fluoroquinolone-resistant (ST1 and non-ST1) versus susceptible cases, and cases 'with' versus 'without' hospital-based contact. IRR=Annual incidence rate ratio.

A. Fluoroquinolone-resistant inferred secondary cases with a hospital link – ST1.

B. Fluoroquinolone-resistant inferred secondary cases with no hospital link – ST1.

C. Fluoroquinolone-resistant inferred secondary cases with a hospital link – non-ST1.

D. Fluoroquinolone-resistant inferred secondary cases with no hospital link – non-ST1.

E. Fluoroquinolone-susceptible inferred secondary cases with a hospital link.

F. Fluoroquinolone-susceptible inferred secondary cases with no hospital link.

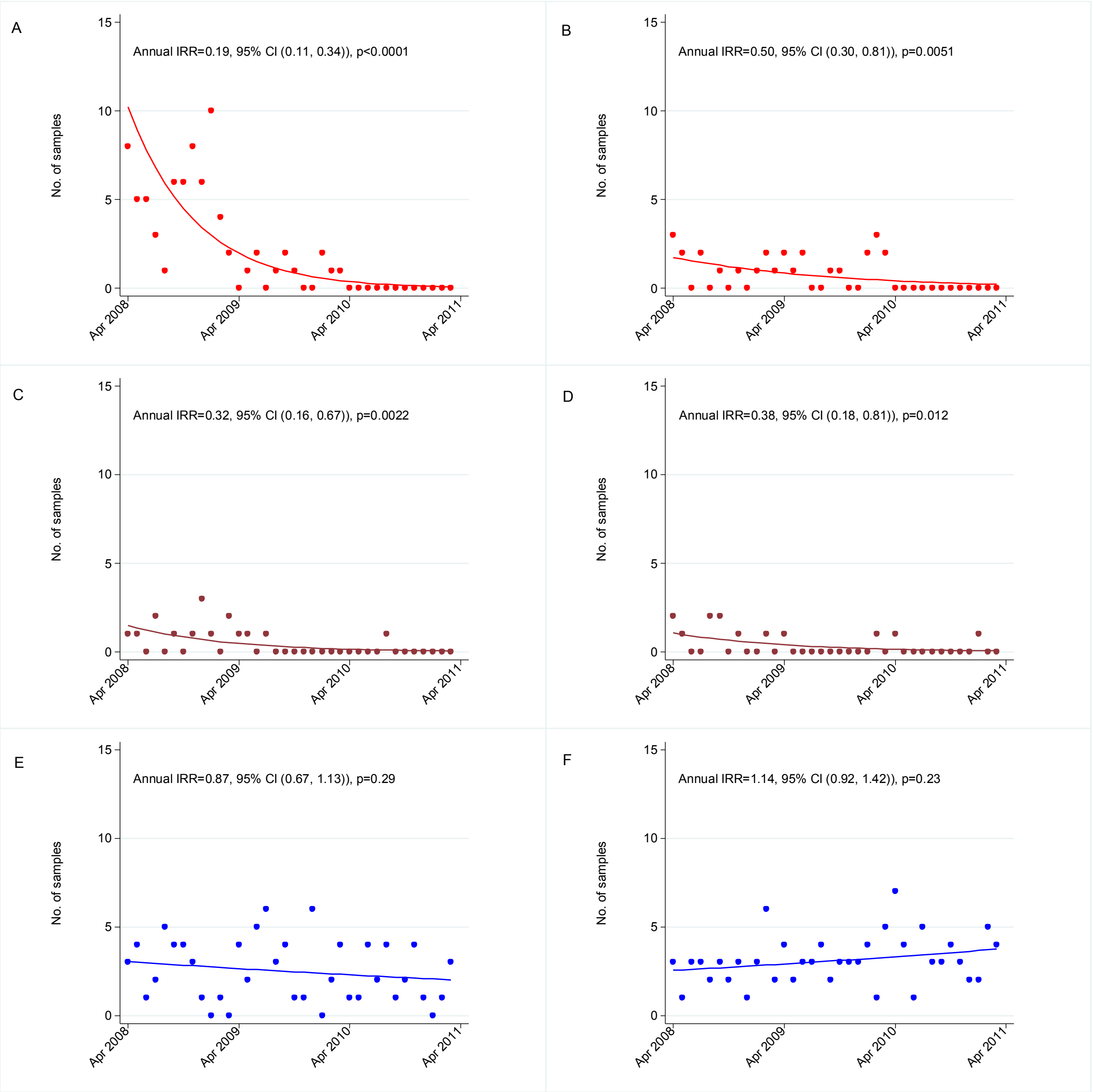

Figure S9. Incidence of CDI and hospital prescribing of extended spectrum and beta-lactamase resistant penicillins in Oxfordshire, (predominantly amoxicillin and flucloxacillin respectively).

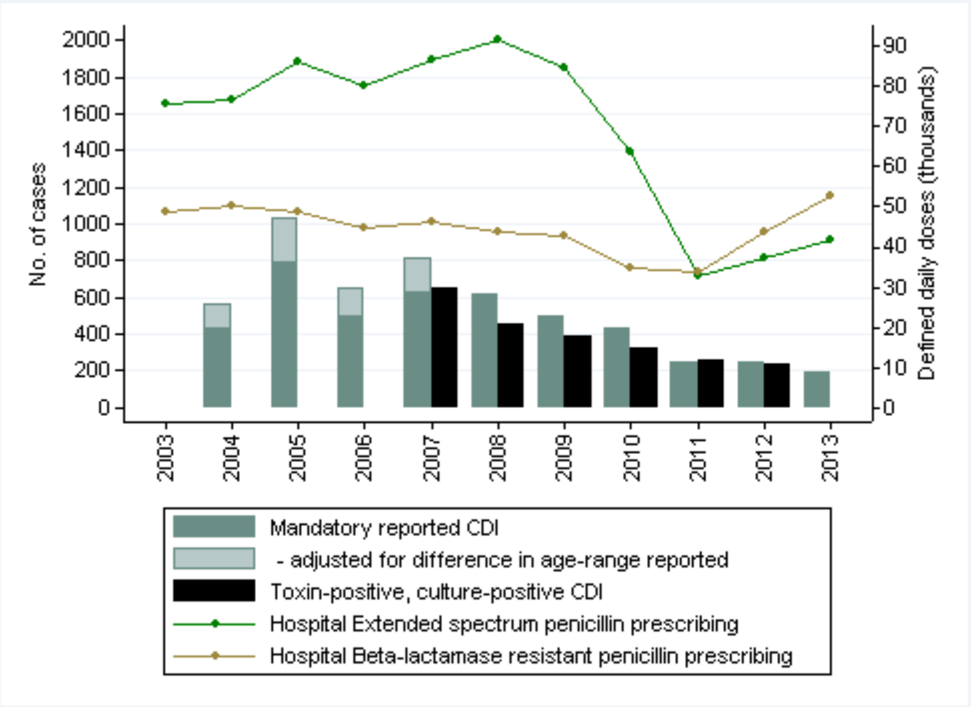

Table S1 Five isolate collections used in the study (designated a – e below).
Isolates included in phylogenies are shaded in cream and a 'Y' is indicated in the 'phylogeny' column. Isolates not included in the phylogenies are on a white background and an 'N' is indicated in the 'Phylogeny' column. 'Y both' in this column for ST8 indicates the isolate was included in both ST8 phylogenies (Figures 4D, S6A-B, and 'Y Oxford only' indicates that the genome was only included in the Oxfordshire ST8 phylogeny (Figure S6B).
All genomes (short reads) have been submitted to NCBI with the BioProject ID PRJNA304087. Accession numbers are listed in the right hand column.
gyrA or *gyrB* 'R' on a brown background; isolate predicted to be phenotypically resistant to fluoroquinolones due to classical mutation in *gyrA* or *gyrB* gene.
gyrA or *gyrB* 'S'; isolate predicted to be phenotypically susceptible to fluoroquinolones due to presence of 'wild type' *gyrA* or *gyrB* gene variant lacking mutation associated with resistance.

(a) Isolate collection: Oxfordshire Human Clinical Isolates (from Enzyme Immunoassay Positive Samples)

Genome Identification Number	Isolate Name	Isolation Date	ST	Phylogeny	clade	*gyrA*	*gyrB*	PaLoc	Country	SRA Accession
C00005042	3-p1	12-Sep-06	1	Y	2	R	S	+	UK	SRR3332970
C00005076	2-p1	13-Sep-06	1	N	2	R	S	+	UK	SRR3334055
C00005043	4-p1	14-Sep-06	1	Y	2	R	S	+	UK	SRR3334101
C00005044	5b-p1	14-Sep-06	1	N	2	R	S	+	UK	SRR3333611
C00005045	7-p1	14-Sep-06	1	N	2	R	S	+	UK	SRR3334455
C00005046	8-p1	16-Sep-06	1	N	2	R	S	+	UK	ERS346834
C00005048	11-p1	16-Sep-06	1	Y	2	R	S	+	UK	SRR3333033
C00005047	10-p1	17-Sep-06	1	N	2	R	S	+	UK	SRR3340225
C00005049	13-p1	18-Sep-06	1	N	2	R	S	+	UK	SRR3333335
C00005050	15-p1	19-Sep-06	1	Y	2	R	S	+	UK	SRR3333352
C00005051	21-p1	22-Sep-06	1	N	2	R	S	+	UK	SRR3333458
C00005053	23-p1	24-Sep-06	1	N	2	R	S	+	UK	SRR3333689
C00005057	25-p1	24-Sep-06	1	Y	2	R	S	+	UK	SRR3333641
C00005052	22-p1	25-Sep-06	1	N	2	R	S	+	UK	SRR3340254
C00005055	24-p1	25-Sep-06	1	N	2	R	S	+	UK	SRR3333420
C00005058	28-p1	26-Sep-06	1	Y	2	R	S	+	UK	SRR3333030
C00005059	29-p1	27-Sep-06	1	N	2	R	S	+	UK	SRR3332984
C00009894	30-p2	28-Sep-06	1	N	2	R	S	+	UK	SRR3340201
C00005061	32-p1	29-Sep-06	1	Y	2	R	S	+	UK	ERS227432
C00005062	36-p1	29-Sep-06	1	N	2	R	S	+	UK	ERS227399

C00005063	38-p1	29-Sep-06	1	N	2	R	S	+	UK	SRR3334442
C00005064	39-p1	01-Oct-06	1	Y	2	R	S	+	UK	SRR3333099
C00005065	45-p1	05-Oct-06	1	N	2	R	S	+	UK	SRR3340061
C00005066	46-p1	06-Oct-06	1	N	2	R	S	+	UK	SRR3334205
C00005067	47-p1	06-Oct-06	1	Y	2	R	S	+	UK	SRR3334102
C00005069	49-p1	07-Oct-06	1	N	2	R	S	+	UK	SRR3332958
C00005070	50a-p1	07-Oct-06	1	N	2	R	S	+	UK	SRR3333020
C00005068	48-p1	08-Oct-06	1	Y	2	R	S	+	UK	SRR3333687
C00005071	51-p1	09-Oct-06	1	N	2	R	S	+	UK	SRR3333754
C00005072	52-p1	10-Oct-06	1	N	2	R	S	+	UK	SRR3333241
C00005073	53-p1	11-Oct-06	1	N	2	R	S	+	UK	ERS227374
C00005074	54-p1	11-Oct-06	1	Y	2	R	S	+	UK	SRR3334095
C00005079	55-p1	17-Oct-06	1	N	2	R	S	+	UK	SRR3333603
C00005080	56-p1	17-Oct-06	1	Y	2	R	S	+	UK	ERS346835
C00005081	59-p1	17-Oct-06	1	N	2	R	S	+	UK	SRR3334056
C00005083	61-p1	22-Oct-06	1	N	2	R	S	+	UK	SRR3333548
C00005084	62-p1	22-Oct-06	1	Y	2	R	S	+	UK	SRR3340218
C00005085	65-p1	23-Oct-06	1	N	2	R	S	+	UK	SRR3333662
C00005086	67-p1	25-Oct-06	1	N	2	R	S	+	UK	SRR3340217
C00005087	70-p1	28-Oct-06	1	Y	2	R	S	+	UK	SRR3333247
C00005089	72-p1	29-Oct-06	1	N	2	R	S	+	UK	SRR3332969
C00007782	75-p2	29-Oct-06	1	N	2	R	S	+	UK	SRR3334153
C00005088	71-p1	30-Oct-06	1	Y	2	R	S	+	UK	ERS227382
C00005091	76-p1	30-Oct-06	1	N	2	R	S	+	UK	SRR3340043
C00005090	74-p1	31-Oct-06	1	N	2	R	S	+	UK	ERS227398
C00005092	77-p1	31-Oct-06	1	Y	2	R	S	+	UK	SRR3333646
C00005093	78-p1	01-Nov-06	1	N	2	R	S	+	UK	SRR3340041
C00005095	83-p1	06-Nov-06	1	N	2	R	S	+	UK	SRR3333432
C00005094	82-p1	07-Nov-06	1	Y	2	R	S	+	UK	SRR3339981
C00005099	88-p1	10-Nov-06	1	N	2	R	S	+	UK	SRR3333053
C00005096	85-p1	11-Nov-06	1	N	2	R	S	+	UK	ERS346836

C00005097	86-p1	13-Nov-06	1	Y	2	R	S	+	UK	SRR3333370
C00005100	89-p1	13-Nov-06	1	N	2	R	S	+	UK	SRR3332987
C00005098	87-p1	14-Nov-06	1	N	2	R	S	+	UK	SRR3333666
C00005102	91-p1	16-Nov-06	1	Y	2	R	S	+	UK	ERS346838
C00005103	93-p1	17-Nov-06	1	N	2	R	S	+	UK	SRR3333729
C00005104	94-p1	17-Nov-06	1	N	2	R	S	+	UK	SRR3340239
C00007806	95-p2	18-Nov-06	1	Y	2	R	S	+	UK	SRR3333313
C00005105	96-p1	19-Nov-06	1	N	2	R	S	+	UK	SRR3334130
C00005106	98-p1	23-Nov-06	1	Y	2	R	S	+	UK	ERS227367
C00005107	99-p1	23-Nov-06	1	N	2	R	S	+	UK	SRR3333043
C00005108	100-p1	23-Nov-06	1	N	2	R	S	+	UK	SRR3334043
C00005109	101-p1	23-Nov-06	1	N	2	R	S	+	UK	SRR3334417
C00005110	105-p1	25-Nov-06	1	Y	2	R	S	+	UK	SRR3333736
C00005129	147-p1	06-Dec-06	1	N	2	R	S	+	UK	ERS227433
C00005112	114-p1	14-Dec-06	1	N	2	R	S	+	UK	SRR3334071
C00005114	115-p1	14-Dec-06	1	Y	2	R	S	+	UK	SRR3333681
C00005115	118a-p1	15-Dec-06	1	N	2	R	S	+	UK	SRR3334171
C00005116	119-p1	15-Dec-06	1	N	2	R	S	+	UK	SRR3333746
C00005117	121-p1	16-Dec-06	1	Y	2	R	S	+	UK	SRR3333426
C00005118	122-p1	19-Dec-06	1	N	2	R	S	+	UK	SRR3333819
C00005119	123-p1	19-Dec-06	1	N	2	R	S	+	UK	SRR3334145
C00005121	125-p1	21-Dec-06	1	Y	2	R	S	+	UK	SRR3334063
C00000205	Oxf129	31-Dec-06	1	N	2	R	S	+	UK	ERS149852
C00005122	127-p1	31-Dec-06	1	N	2	R	S	+	UK	ERS346841
C00005123	130-p1	04-Jan-07	1	Y	2	R	S	+	UK	SRR3334460
C00005125	137-p1	07-Jan-07	1	N	2	R	S	+	UK	SRR3333271
C00005662	136-p1	08-Jan-07	1	N	2	R	S	+	UK	ERS346852
C00005124	133-p1	09-Jan-07	1	Y	2	R	S	+	UK	ERS346842
C00005127	140-p1	11-Jan-07	1	N	2	R	S	+	UK	SRR3333626
C00005128	141-p1	13-Jan-07	1	N	2	R	S	+	UK	SRR3340187
C00005663	142-p1	15-Jan-07	1	Y	2	R	S	+	UK	ERS346853

C00005130	150-p1	18-Jan-07	1	N	2	R	S	+	UK	SRR3333809
C00005664	154-p1	19-Jan-07	1	N	2	R	S	+	UK	ERS346854
C00000066	Oxf152	21-Jan-07	1	Y	2	R	S	+	UK	ERS149791
C00005131	156-p1	25-Jan-07	1	N	2	R	S	+	UK	SRR3334078
C00000090	Oxf160	28-Jan-07	1	N	2	R	S	+	UK	ERS139371
C00005665	162-p1	28-Jan-07	1	Y	2	R	S	+	UK	ERS346855
C00003952	Oxf163-p1	29-Jan-07	1	N	2	R	S	+	UK	ERS346602
C00000206	Oxf169	02-Feb-07	1	N	2	R	S	+	UK	ERS149853
C00005133	171-p1	04-Feb-07	1	Y	2	R	S	+	UK	SRR3333025
C00005134	172-p1	05-Feb-07	1	N	2	R	S	+	UK	SRR3333704
C00005135	173-p1	05-Feb-07	1	N	2	R	S	+	UK	ERS227393
C00005136	177-p1	05-Feb-07	1	Y	2	R	S	+	UK	SRR3333578
C00005137	178-p1	05-Feb-07	1	N	2	R	S	+	UK	SRR3333635
C00005138	181-p1	07-Feb-07	1	N	2	R	S	+	UK	SRR3340223
C00000091	Oxf183	09-Feb-07	1	Y	2	R	S	+	UK	ERS149812
C00005140	184-p1	09-Feb-07	1	N	2	R	S	+	UK	SRR3333363
C00005141	185-p1	09-Feb-07	1	N	2	R	S	+	UK	SRR3340234
C00005142	186-p1	11-Feb-07	1	Y	2	R	S	+	UK	SRR3333369
C00000207	Oxf187	12-Feb-07	1	Y	2	R	S	+	UK	ERS149854
C00005143	188-p1	12-Feb-07	1	N	2	R	S	+	UK	SRR3340236
C00005146	197a-p1	12-Feb-07	1	N	2	R	S	+	UK	SRR3334069
C00005145	196-p1	13-Feb-07	1	N	2	R	S	+	UK	SRR3333758
C00005148	198-p1	15-Feb-07	1	N	2	R	S	+	UK	SRR3332986
C00007818	201-p2	18-Feb-07	1	Y	2	R	S	+	UK	SRR3334113
C00005149	205-p1	19-Feb-07	1	N	2	R	S	+	UK	SRR3340012
C00005152	209-p1	20-Feb-07	1	N	2	R	S	+	UK	SRR3340205
C00005153	213-p1	27-Feb-07	1	Y	2	R	S	+	UK	SRR3334039
C00005154	214-p1	27-Feb-07	1	N	2	R	S	+	UK	SRR3340261
C00005155	218-p1	01-Mar-07	1	Y	2	R	S	+	UK	SRR3333766
C00005668	217-p1	01-Mar-07	1	N	2	R	S	+	UK	ERS346857
C00005159	231-p1	02-Mar-07	1	N	2	R	S	+	UK	SRR3333284

C00005158	229-p1	03-Mar-07	1	N	2	R	S	+	UK	SRR3333256
C00000067	Oxf224	04-Mar-07	1	N	2	R	S	+	UK	ERS149792
C00000208	Oxf223	04-Mar-07	1	N	2	R	S	+	UK	ERS149855
C00005156	220-p1	04-Mar-07	1	Y	2	R	S	+	UK	SRR3334305
C00005157	226-p1	05-Mar-07	1	Y	2	R	S	+	UK	ERS346844
C00005160	232-p1	05-Mar-07	1	N	2	R	S	+	UK	SRR3333031
C00003953	Oxf234-p1	06-Mar-07	1	N	2	R	S	+	UK	ERS346603
C00005162	239-p1	06-Mar-07	1	Y	2	R	S	+	UK	SRR3334463
C00005161	238-p1	07-Mar-07	1	N	2	R	S	+	UK	SRR3334082
C00005165	242-p1	08-Mar-07	1	N	2	R	S	+	UK	SRR3333068
C00005166	246-p1	09-Mar-07	1	Y	2	R	S	+	UK	SRR3340229
C00000209	Oxf249	10-Mar-07	1	N	2	R	S	+	UK	ERS149856
C00005167	248-p1	11-Mar-07	1	N	2	R	S	+	UK	SRR3334468
C00005175	250-p1	11-Mar-07	1	Y	2	R	S	+	UK	SRR3333009
C00005176	252-p1	13-Mar-07	1	N	2	R	S	+	UK	SRR3333648
C00005178	258-p1	16-Mar-07	1	N	2	R	S	+	UK	SRR3333579
C00005177	257-p1	17-Mar-07	1	Y	2	R	S	+	UK	SRR3333390
C00005179	259-p1	17-Mar-07	1	N	2	R	S	+	UK	SRR3334183
C00000068	Oxf262	18-Mar-07	1	N	2	R	S	+	UK	ERS149793
C00005180	263-p1	20-Mar-07	1	Y	2	R	S	+	UK	SRR3333267
C00005182	264-p1	20-Mar-07	1	N	2	R	S	+	UK	SRR3333703
C00005183	266-p1	21-Mar-07	1	N	2	R	S	+	UK	SRR3334438
C00005184	270-p1	26-Mar-07	1	Y	2	R	S	+	UK	SRR3340025
C00000210	Oxf271	27-Mar-07	1	N	2	R	S	+	UK	ERS149857
C00000069	Oxf275	28-Mar-07	1	N	2	R	S	+	UK	ERS149794
C00005185	278-p1	29-Mar-07	1	Y	2	R	S	+	UK	SRR3333355
C00005187	280-p1	30-Mar-07	1	N	2	R	S	+	UK	SRR3334435
C00005195	295-p1	04-Apr-07	1	N	2	R	S	+	UK	SRR3333311
C00000211	Oxf285	06-Apr-07	1	N	2	R	S	+	UK	ERS149858
C00005188	282-p1	06-Apr-07	1	Y	2	R	S	+	UK	SRR3333400
C00005189	284-p1	06-Apr-07	1	N	2	R	S	+	UK	SRR3333022

C00005190	286-p1	08-Apr-07	1	Y	2	R	S	+	UK	SRR3340184
C00005191	290-p1	10-Apr-07	1	N	2	R	S	+	UK	SRR3333558
C00005192	291-p1	11-Apr-07	1	N	2	R	S	+	UK	SRR3340001
C00005193	293-p1	12-Apr-07	1	N	2	R	S	+	UK	SRR3333470
C00005194	294-p1	12-Apr-07	1	Y	2	R	S	+	UK	SRR3339974
C00005196	297-p1	12-Apr-07	1	Y	2	R	S	+	UK	SRR3332960
C00005198	299-p1	12-Apr-07	1	N	2	R	S	+	UK	SRR3333294
C00000044	Oxf298	13-Apr-07	1	N	2	R	S	+	UK	ERS149772
C00005199	300-p1	13-Apr-07	1	N	2	R	S	+	UK	SRR3333011
C00005200	302-p1	14-Apr-07	1	Y	2	R	S	+	UK	SRR3333446
C00005201	303-p1	14-Apr-07	1	N	2	R	S	+	UK	SRR3334167
C00005202	305-p1	15-Apr-07	1	N	2	R	S	+	UK	SRR3332949
C00005203	308-p1	15-Apr-07	1	Y	2	R	S	+	UK	SRR3333399
C00009906	313-p2	17-Apr-07	1	N	2	R	S	+	UK	SRR3334115
C00005204	311-p1	18-Apr-07	1	N	2	R	S	+	UK	SRR3340032
C00000045	Oxf314	19-Apr-07	1	Y	2	R	S	+	UK	ERS149773
C00005206	319-p1	22-Apr-07	1	N	2	R	S	+	UK	SRR3334021
C00005207	321-p1	24-Apr-07	1	N	2	R	S	+	UK	SRR3333806
C00005208	322-p1	24-Apr-07	1	Y	2	R	S	+	UK	SRR3340279
C00005209	323-p1	24-Apr-07	1	N	2	R	S	+	UK	SRR3333636
C00005211	330-p1	24-Apr-07	1	N	2	R	S	+	UK	SRR3333544
C00005212	331-p1	25-Apr-07	1	Y	2	R	S	+	UK	SRR3333464
C00009918	326-p2	25-Apr-07	1	N	2	R	S	+	UK	SRR3334136
C00005214	335-p1	27-Apr-07	1	N	2	R	S	+	UK	ERS227417
C00005213	334-p1	28-Apr-07	1	Y	2	R	S	+	UK	SRR3334156
C00005215	342-p1	01-May-07	1	N	2	R	S	+	UK	SRR3340182
C00005216	343-p1	01-May-07	1	N	2	R	S	+	UK	SRR3333057
C00005217	347-p1	03-May-07	1	Y	2	R	S	+	UK	SRR3334015
C00005218	351-p1	04-May-07	1	N	2	R	S	+	UK	SRR3334016
C00005219	356-p1	04-May-07	1	Y	2	R	S	+	UK	SRR3333816
C00005672	353-p1	04-May-07	1	N	2	R	S	+	UK	ERS346859

C00005223	361-p1	06-May-07	1	N	2	R	S	+	UK	SRR3334220
C00005221	359-p1	07-May-07	1	N	2	R	S	+	UK	SRR3334169
C00005222	360-p1	07-May-07	1	Y	2	R	S	+	UK	SRR3333312
C00005224	362-p1	10-May-07	1	N	2	R	S	+	UK	SRR3334075
C00000212	Oxf368	12-May-07	1	N	2	R	S	+	UK	ERS149859
C00009930	363-p2	12-May-07	1	Y	2	R	S	+	UK	SRR3340176
C00005226	367-p1	13-May-07	1	N	2	R	S	+	UK	ERS227409
C00005227	373-p1	15-May-07	1	N	2	R	S	+	UK	ERS227412
C00005228	375a-p1	16-May-07	1	Y	2	R	S	+	UK	SRR3333235
C00005229	376-p1	17-May-07	1	N	2	R	S	+	UK	SRR3333477
C00005231	385-p1	23-May-07	1	N	2	R	S	+	UK	SRR3333630
C00005230	383-p1	24-May-07	1	N	2	R	S	+	UK	SRR3333291
C00005232	387-p1	24-May-07	1	Y	2	R	S	+	UK	SRR3333424
C00005233	388-p1	25-May-07	1	N	2	R	S	+	UK	SRR3340230
C00005236	396-p1	29-May-07	1	Y	2	R	S	+	UK	SRR3333298
C00005234	393-p1	30-May-07	1	N	2	R	S	+	UK	SRR3333737
C00005235	395-p1	30-May-07	1	N	2	R	S	+	UK	SRR3333790
C00005237	401-p1	31-May-07	1	Y	2	R	S	+	UK	SRR3334428
C00005238	402-p1	31-May-07	1	N	2	R	S	+	UK	SRR3340021
C00005239	403-p1	31-May-07	1	N	2	R	S	+	UK	SRR3340265
C00005240	414-p1	07-Jun-07	1	Y	2	R	S	+	UK	SRR3333306
C00000094	Oxf424	08-Jun-07	1	N	2	R	S	+	UK	ERS149813
C00005241	418-p1	08-Jun-07	1	N	2	R	S	+	UK	SRR3333065
C00005243	426-p1	13-Jun-07	1	Y	2	R	S	+	UK	SRR3333295
C00005244	439-p1	17-Jun-07	1	N	2	R	S	+	UK	SRR3333410
C00000095	Oxf438	18-Jun-07	1	N	2	R	S	+	UK	ERS149814
C00005245	441b-p1	18-Jun-07	1	Y	2	R	S	+	UK	SRR3340258
C00005246	450a-p1	22-Jun-07	1	N	2	R	S	+	UK	SRR3333104
C00005247	451-p1	22-Jun-07	1	N	2	R	S	+	UK	SRR3333414
C00005248	452-p1	25-Jun-07	1	Y	2	R	S	+	UK	SRR3340074
C00000165	Oxf457-2	30-Jun-07	1	N	2	R	S	+	UK	SRR3339966

C00005249	459-p1	01-Jul-07	1	N	2	R	S	+	UK	SRR3334010
C00005251	461-p1	03-Jul-07	1	N	2	R	S	+	UK	SRR3332993
C00005675	465-p1	03-Jul-07	1	Y	2	R	S	+	UK	ERS346861
C00005250	460-p1	04-Jul-07	1	N	2	R	S	+	UK	SRR3333705
C00001639	Oxf463	05-Jul-07	1	N	2	R	S	+	UK	ERS150024
C00005253	470-p1	05-Jul-07	1	Y	2	R	S	+	UK	SRR3333797
C00005252	469-p1	06-Jul-07	1	Y	2	R	S	+	UK	SRR3333765
C00005256	474-p1	06-Jul-07	1	N	2	R	S	+	UK	SRR3333371
C00009937	468-p2	06-Jul-07	1	N	2	R	S	+	UK	SRR3334150
C00005254	472-p1	07-Jul-07	1	N	2	R	S	+	UK	SRR3340256
C00005257	477-p1	08-Jul-07	1	Y	2	R	S	+	UK	SRR3333265
C00009942	478-p2	08-Jul-07	1	N	2	R	S	+	UK	SRR3334447
C00005261	483-p1	12-Jul-07	1	N	2	R	S	+	UK	SRR3340252
C00005262	484-p1	12-Jul-07	1	Y	2	R	S	+	UK	SRR3333768
C00005263	488-p1	13-Jul-07	1	N	2	R	S	+	UK	SRR3334229
C00000162	Oxf490	14-Jul-07	1	N	2	R	S	+	UK	ERS149838
C00004795	494-p1	14-Jul-07	1	Y	2	R	S	+	UK	SRR3333249
C00004871	489-p1	14-Jul-07	1	N	2	R	S	+	UK	ERS227371
C00000163	Oxf491	15-Jul-07	1	N	2	R	S	+	UK	ERS149839
C00004818	492-p1	15-Jul-07	1	Y	2	R	S	+	UK	SRR3333338
C00004816	502-p1	16-Jul-07	1	N	2	R	S	+	UK	SRR3333811
C00004850	504-p1	17-Jul-07	1	N	2	R	S	+	UK	SRR3334038
C00004851	507-p1	19-Jul-07	1	Y	2	R	S	+	UK	SRR3333112
C00004840	524-p1	25-Jul-07	1	N	2	R	S	+	UK	ERS227376
C00004799	531-p1	28-Jul-07	1	N	2	R	S	+	UK	SRR3333016
C00004854	533-p1	29-Jul-07	1	Y	2	R	S	+	UK	SRR3333587
C00004861	534-p1	29-Jul-07	1	N	2	R	S	+	UK	SRR3334182
C00001632	Oxf537	30-Jul-07	1	N	2	R	S	+	UK	SRR3333384
C00005677	538-p1	30-Jul-07	1	Y	2	R	S	+	UK	ERS346862
C00004842	542-p1	31-Jul-07	1	N	2	R	S	+	UK	SRR3333658
C00004836	544-p1	01-Aug-07	1	Y	2	R	S	+	UK	SRR3334014

C00009966	547-p2	01-Aug-07	1	N	2	R	S	+	UK	SRR3340263
C00000202	Oxf549	02-Aug-07	1	N	2	R	S	+	UK	ERS149850
C00003964	Oxf550-p1	03-Aug-07	1	N	2	R	S	+	UK	ERS346608
C00004835	567-p1	03-Aug-07	1	Y	2	R	S	+	UK	SRR3333991
C00000204	Oxf558	05-Aug-07	1	N	2	R	S	+	UK	ERS149851
C00004874	570-p1	10-Aug-07	1	N	2	R	S	+	UK	ERS346796
C00004829	577-p1	13-Aug-07	1	N	2	R	S	+	UK	SRR3333253
C00004868	573-p1	13-Aug-07	1	Y	2	R	S	+	UK	SRR3334185
C00000047	Oxf578	14-Aug-07	1	N	2	R	S	+	UK	ERS149775
C00004800	583-p1	14-Aug-07	1	N	2	R	S	+	UK	SRR3333073
C00004887	580-p1	14-Aug-07	1	Y	2	R	S	+	UK	SRR3333616
C00004821	589-p1	17-Aug-07	1	N	2	R	S	+	UK	SRR3333802
C00005679	585-p1	20-Aug-07	1	Y	2	R	S	+	UK	ERS346864
C00004883	587-p1	21-Aug-07	1	N	2	R	S	+	UK	SRR3333320
C00004831	600-p1	23-Aug-07	1	N	2	R	S	+	UK	SRR3333054
C00004797	601-p1	25-Aug-07	1	Y	2	R	S	+	UK	SRR3334449
C00004798	610-p1	25-Aug-07	1	N	2	R	S	+	UK	SRR3333070
C00004862	611-p1	29-Aug-07	1	N	2	R	S	+	UK	SRR3334126
C00009978	612-p2	29-Aug-07	1	Y	2	R	S	+	UK	SRR3340026
C00004805	620-p1	30-Aug-07	1	N	2	R	S	+	UK	SRR3334152
C00005746	619-p1	30-Aug-07	1	N	2	R	S	+	UK	SRR3333767
C00004822	626-p1	31-Aug-07	1	Y	2	R	S	+	UK	SRR3334093
C00000054	Oxf629	01-Sep-07	1	N	2	R	S	+	UK	ERS149782
C00008987	632m-p1	03-Sep-07	1	Y	2	R	S	+	UK	ERS227506
C00008999	631m-p1	03-Sep-07	1	N	2	R	S	+	UK	ERS227509
C00000055	Oxf640	04-Sep-07	1	N	2	R	S	+	UK	ERS149783
C00004869	645-p1	07-Sep-07	1	N	2	R	S	+	UK	ERS346794
C00004878	656-p1	07-Sep-07	1	Y	2	R	S	+	UK	ERS346800
C00004853	664-p1	12-Sep-07	1	N	2	R	S	+	UK	ERS346784
C00004808	678-p1	17-Sep-07	1	Y	2	R	S	+	UK	SRR3340207
C00004811	677-p1	17-Sep-07	1	N	2	R	S	+	UK	ERS346765

C00004825	689-p1	18-Sep-07	1	N	2	R	S	+	UK	ERS346773
C00004876	690-p1	18-Sep-07	1	N	2	R	S	+	UK	ERS346798
C00004877	691b-p1	19-Sep-07	1	Y	2	R	S	+	UK	ERS346799
C00004803	706-p1	25-Sep-07	1	N	2	R	S	+	UK	ERS346761
C00004859	711-p1	26-Sep-07	1	N	2	R	S	+	UK	ERS346787
C00005744	720-p1	01-Oct-07	1	Y	2	R	S	+	UK	ERS346911
C00006367	723-p2	03-Oct-07	1	N	2	R	S	+	UK	ERS347162
C00005747	724-p1	05-Oct-07	1	N	2	R	S	+	UK	ERS346912
C00005748	726-p1	05-Oct-07	1	Y	2	R	S	+	UK	ERS346913
C00005749	729-p1	08-Oct-07	1	N	2	R	S	+	UK	ERS346914
C00005750	730-p1	08-Oct-07	1	N	2	R	S	+	UK	ERS346915
C00005751	734-p1	08-Oct-07	1	Y	2	R	S	+	UK	ERS346916
C00005752	744-p1	15-Oct-07	1	N	2	R	S	+	UK	ERS346917
C00005753	745-p1	16-Oct-07	1	N	2	R	S	+	UK	ERS346918
C00005755	757-p1	17-Oct-07	1	Y	2	R	S	+	UK	ERS346920
C00005754	754-p1	18-Oct-07	1	N	2	R	S	+	UK	ERS346919
C00005757	759-p1	20-Oct-07	1	N	2	R	S	+	UK	ERS346921
C00000087	Oxf763	21-Oct-07	1	Y	2	R	S	+	UK	ERS149810
C00005758	764-p1	21-Oct-07	1	N	2	R	S	+	UK	ERS346922
C00005760	766-p1	22-Oct-07	1	N	2	R	S	+	UK	ERS346923
C00000088	Oxf768	23-Oct-07	1	Y	2	R	S	+	UK	ERS149811
C00005761	789c-p1	31-Oct-07	1	N	2	R	S	+	UK	ERS346924
C00005762	803-p1	08-Nov-07	1	N	2	R	S	+	UK	ERS346925
C00000065	Oxf806	10-Nov-07	1	Y	2	R	S	+	UK	ERS149790
C00005763	812-p1	11-Nov-07	1	N	2	R	S	+	UK	ERS346926
C00005764	813-p1	12-Nov-07	1	N	2	R	S	+	UK	ERS346927
C00005765	818-p1	13-Nov-07	1	Y	2	R	S	+	UK	ERS346928
C00005766	830-p1	17-Nov-07	1	N	2	R	S	+	UK	ERS346929
C00005767	832-p1	19-Nov-07	1	N	2	R	S	+	UK	ERS346930
C00005768	836-p1	20-Nov-07	1	Y	2	R	S	+	UK	ERS346931
C00000216	Oxf846	26-Nov-07	1	N	2	R	S	+	UK	ERS149861

C00005769	847-p1	26-Nov-07	1	N	2	R	S	+	UK	ERS346932
C00005770	863-p1	01-Dec-07	1	Y	2	R	S	+	UK	ERS346933
C00000042	Oxf865	02-Dec-07	1	N	2	R	S	+	UK	ERS149770
C00005771	873-p1	04-Dec-07	1	N	2	R	S	+	UK	ERS227384
C00005772	885-p1	11-Dec-07	1	Y	2	R	S	+	UK	ERS346934
C00009907	894-p2	12-Dec-07	1	N	2	R	S	+	UK	ERS347373
C00000515	Oxf898	14-Dec-07	1	N	2	R	S	+	UK	ERS149889
C00000221	Oxf905	19-Dec-07	1	Y	2	R	S	+	UK	ERS149865
C00005774	911-p1	21-Dec-07	1	N	2	R	S	+	UK	ERS346935
C00005775	916-p1	24-Dec-07	1	N	2	R	S	+	UK	ERS346936
C00005776	917-p1	24-Dec-07	1	Y	2	R	S	+	UK	ERS346937
C00005777	919-p1	25-Dec-07	1	N	2	R	S	+	UK	ERS346938
C00005778	925-p1	27-Dec-07	1	N	2	R	S	+	UK	ERS346939
C00003974	Oxf937-p1	30-Dec-07	1	N	2	R	S	+	UK	ERS346612
C00005779	930-p1	30-Dec-07	1	Y	2	R	S	+	UK	ERS346940
C00000152	Oxf945	04-Jan-08	1	N	2	R	S	+	UK	ERS149830
C00005780	958-p1	10-Jan-08	1	Y	2	R	S	+	UK	ERS346941
C00005781	961-p1	11-Jan-08	1	N	2	R	S	+	UK	ERS346942
C00005782	968-p1	13-Jan-08	1	N	2	R	S	+	UK	ERS227410
C00005784	975-p1	15-Jan-08	1	Y	2	R	S	+	UK	ERS346943
C00000153	Oxf984	18-Jan-08	1	N	2	R	S	+	UK	ERS149831
C00005785	982-p1	18-Jan-08	1	N	2	R	S	+	UK	ERS346944
C00005786	985-p1	18-Jan-08	1	Y	2	R	S	+	UK	ERS346945
C00005787	1008-p1	23-Jan-08	1	N	2	R	S	+	UK	ERS346946
C00009919	1011-p2	24-Jan-08	1	N	2	R	S	+	UK	ERS347375
C00005788	1015-p1	26-Jan-08	1	Y	2	R	S	+	UK	ERS346947
C00000154	Oxf1035	31-Jan-08	1	N	2	R	S	+	UK	ERS149832
C00005789	1040-p1	03-Feb-08	1	N	2	R	S	+	UK	ERS346948
C00006359	1041-p1	04-Feb-08	1	Y	2	R	S	+	UK	ERS347156
C00005790	1087-p1	27-Feb-08	1	N	2	R	S	+	UK	ERS346949
C00005791	1096-p1	01-Mar-08	1	N	2	R	S	+	UK	SRR3334448

C00005792	1119-p1	07-Mar-08	1	Y	2	R	S	+	UK	ERS346950
C00005793	1131-p1	12-Mar-08	1	N	2	R	S	+	UK	ERS346951
C00005794	1136-p1	13-Mar-08	1	N	2	R	S	+	UK	ERS346952
C00005911	1137-p1	14-Mar-08	1	Y	2	R	S	+	UK	ERS347042
C00005797	1146-p1	20-Mar-08	1	N	2	R	S	+	UK	ERS346953
C00005798	1151-p1	23-Mar-08	1	N	2	R	S	+	UK	ERS346954
C00005799	1168-p1	01-Apr-08	1	Y	2	R	S	+	UK	ERS346955
C00000052	Oxf1175	04-Apr-08	1	N	2	R	S	+	UK	ERS149780
C00005800	1173-p1	04-Apr-08	1	N	2	R	S	+	UK	ERS346956
C00005801	1183-p1	10-Apr-08	1	Y	2	R	S	+	UK	ERS346957
C00000198	Oxf1201	15-Apr-08	1	N	2	R	S	+	UK	ERS149847
C00005802	1208-p1	18-Apr-08	1	N	2	R	S	+	UK	ERS227427
C00005803	1210-p1	19-Apr-08	1	Y	2	R	S	+	UK	ERS346958
C00005805	1216-p1	20-Apr-08	1	N	2	R	S	+	UK	ERS346959
C00005806	1220-p1	22-Apr-08	1	N	2	R	S	+	UK	ERS346960
C00005807	1222-p1	24-Apr-08	1	N	2	R	S	+	UK	ERS346961
C00005808	1224-p1	24-Apr-08	1	Y	2	R	S	+	UK	ERS227357
C00005809	1240-p1	28-Apr-08	1	N	2	R	S	+	UK	ERS346962
C00005810	1243-p1	29-Apr-08	1	Y	2	R	S	+	UK	ERS346963
C00000514	Oxf1257	02-May-08	1	N	2	R	S	+	UK	ERS149888
C00005811	1263-p1	03-May-08	1	N	2	R	S	+	UK	ERS346964
C00003976	Oxf1267-p1	05-May-08	1	Y	2	R	S	+	UK	ERS346614
C00005812	1277-p1	09-May-08	1	N	2	R	S	+	UK	ERS346965
C00005813	1292-p1	17-May-08	1	N	2	R	S	+	UK	ERS227361
C00005814	1298-p1	21-May-08	1	Y	2	R	S	+	UK	ERS346966
C00005815	1301-p1	26-May-08	1	N	2	R	S	+	UK	ERS346967
C00005816	1317c-p1	31-May-08	1	N	2	R	S	+	UK	ERS346968
C00000494	Oxf1323	04-Jun-08	1	Y	2	R	S	+	UK	ERS149876
C00005817	1352-p1	17-Jun-08	1	N	2	R	S	+	UK	ERS346969
C00005818	1353-p1	19-Jun-08	1	N	2	R	S	+	UK	ERS346970
C00005819	1360-p1	24-Jun-08	1	Y	2	R	S	+	UK	ERS346971

C00005820	1363-p1	26-Jun-08	1	N	2	R	S	+	UK	ERS346972
C00005821	1372-p1	28-Jun-08	1	N	2	R	S	+	UK	ERS346973
C00005822	1374-p1	01-Jul-08	1	Y	2	R	S	+	UK	ERS346974
C00005823	1376-p1	03-Jul-08	1	N	2	R	S	+	UK	ERS346975
C00005824	1385-p1	09-Jul-08	1	N	2	R	S	+	UK	ERS346976
C00005825	1404-p1	18-Jul-08	1	Y	2	R	S	+	UK	ERS346977
C00005826	1407-p1	21-Jul-08	1	N	2	R	S	+	UK	ERS346978
C00005827	1421-p1	29-Jul-08	1	N	2	R	S	+	UK	ERS346979
C00005828	1442-p1	15-Aug-08	1	Y	2	R	S	+	UK	ERS227400
C00000128	Oxf1448	18-Aug-08	1	N	2	R	S	+	UK	ERS149820
C00005829	1484-p1	10-Sep-08	1	N	2	R	S	+	UK	ERS346980
C00005830	1489-p1	12-Sep-08	1	Y	2	R	S	+	UK	ERS346981
C00000199	Oxf1491	14-Sep-08	1	N	2	R	S	+	UK	ERS149848
C00005831	1490-p1	15-Sep-08	1	N	2	R	S	+	UK	ERS346982
C00005832	1503a-p1	19-Sep-08	1	Y	2	R	S	+	UK	ERS346983
C00010618	1506-p3	20-Sep-08	1	N	2	R	S	+	UK	SRR3333038
C00000079	Oxf1512	22-Sep-08	1	N	2	R	S	+	UK	ERS149802
C00000200	Oxf1517	24-Sep-08	1	Y	2	R	S	+	UK	ERS149849
C00004547	1527-9-p1	26-Sep-08	1	N	2	R	S	+	UK	ERS346755
C00005836	1524-p1	26-Sep-08	1	N	2	R	S	+	UK	ERS346984
C00010619	1532-p2	30-Sep-08	1	Y	2	R	S	+	UK	ERS347387
C00005838	1538a-p1	06-Oct-08	1	N	2	R	S	+	UK	ERS346985
C00005840	1550-p1	09-Oct-08	1	N	2	R	S	+	UK	ERS346986
C00005841	1573-p1	25-Oct-08	1	Y	2	R	S	+	UK	ERS346987
C00005844	1582-p1	29-Oct-08	1	N	2	R	S	+	UK	ERS346988
C00010620	1579-p2	29-Oct-08	1	N	2	R	S	+	UK	ERS347388
C00005846	1583-p1	30-Oct-08	1	Y	2	R	S	+	UK	ERS346989
C00005848	1584-p1	31-Oct-08	1	N	2	R	S	+	UK	ERS346990
C00005849	1588-p1	04-Nov-08	1	N	2	R	S	+	UK	ERS346991
C00005850	1592-p1	10-Nov-08	1	Y	2	R	S	+	UK	ERS346992
C00003987	Oxf1605-p1	13-Nov-08	1	N	2	R	S	+	UK	ERS346620

C00000130	Oxf1607	15-Nov-08	1	N	2	R	S	+	UK	ERS149821
C00005851	1611-p1	16-Nov-08	1	Y	2	R	S	+	UK	ERS346993
C00005852	1614-p1	17-Nov-08	1	N	2	R	S	+	UK	ERS346994
C00000132	Oxf1626-1	19-Nov-08	1	Y	2	R	S	+	UK	SRR3333732
C00005854	1625-p1	19-Nov-08	1	N	2	R	S	+	UK	ERS346995
C00005855	1627-p1	20-Nov-08	1	N	2	R	S	+	UK	ERS346996
C00000135	Oxf1632	24-Nov-08	1	N	2	R	S	+	UK	ERS149822
C00001460	Oxf1639	28-Nov-08	1	Y	2	R	S	+	UK	ERS149915
C00010621	1641-p2	01-Dec-08	1	N	2	R	S	+	UK	ERS347389
C00005857	1645-p1	03-Dec-08	1	N	2	R	S	+	UK	ERS346997
C00005858	1653-p1	05-Dec-08	1	Y	2	R	S	+	UK	ERS346998
C00005859	1663-p1	12-Dec-08	1	N	2	R	S	+	UK	ERS346999
C00005860	1669-p1	15-Dec-08	1	N	2	R	S	+	UK	ERS347000
C00000136	Oxf1681	19-Dec-08	1	Y	2	R	S	+	UK	ERS149823
C00005861	1692-p1	07-Jan-09	1	N	2	R	S	+	UK	ERS347001
C00005862	1694-p1	07-Jan-09	1	N	2	R	S	+	UK	ERS347002
C00005863	1699-p1	09-Jan-09	1	Y	2	R	S	+	UK	ERS347003
C00005864	1706-p1	10-Jan-09	1	N	2	R	S	+	UK	ERS347004
C00003994	Oxf1712-p1	13-Jan-09	1	N	2	R	S	+	UK	ERS346623
C00003995	Oxf1720-p1	16-Jan-09	1	Y	2	R	S	+	UK	ERS346624
C00005865	1726-p1	19-Jan-09	1	N	2	R	S	+	UK	ERS347005
C00005867	1734-p1	25-Jan-09	1	N	2	R	S	+	UK	ERS347006
C00000084	Oxf1743	31-Jan-09	1	N	2	R	S	+	UK	ERS149807
C00005868	1741-p1	31-Jan-09	1	Y	2	R	S	+	UK	ERS347007
C00005869	1744-p1	31-Jan-09	1	N	2	R	S	+	UK	ERS347008
C00005870	1762-p1	07-Feb-09	1	Y	2	R	S	+	UK	ERS347009
C00005872	1765-p1	08-Feb-09	1	N	2	R	S	+	UK	ERS347010
C00000085	Oxf1768	09-Feb-09	1	N	2	R	S	+	UK	ERS149808
C00000086	Oxf1777	15-Feb-09	1	Y	2	R	S	+	UK	ERS149809
C00005873	1780-p1	16-Feb-09	1	N	2	R	S	+	UK	SRR3339955
C00000075	Oxf1795	25-Feb-09	1	N	2	R	S	+	UK	ERS149799

C00005874	1802a-p1	27-Feb-09	1	Y	2	R	S	+	UK	ERS347011
C00005875	1815-p1	06-Mar-09	1	N	2	R	S	+	UK	ERS347012
C00005876	1826-p1	10-Mar-09	1	N	2	R	S	+	UK	ERS347013
C00005877	1840b-p1	16-Mar-09	1	Y	2	R	S	+	UK	ERS347014
C00005878	1862-p1	09-Apr-09	1	N	2	R	S	+	UK	ERS347015
C00005879	1877-p1	20-Apr-09	1	N	2	R	S	+	UK	ERS347016
C00005880	1902-p1	09-May-09	1	Y	2	R	S	+	UK	ERS347017
C00005881	1908-p1	15-May-09	1	N	2	R	S	+	UK	ERS347018
C00005882	1939b-p1	03-Jun-09	1	N	2	R	S	+	UK	ERS347019
C00005883	1943-p1	07-Jun-09	1	Y	2	R	S	+	UK	ERS347020
C00005705	1954-p1	10-Jun-09	1	N	2	R	S	+	UK	ERS346885
C00001478	Oxf1968a	18-Jun-09	1	N	2	R	S	+	UK	ERS149927
C00005884	2089a-p1	23-Aug-09	1	Y	2	R	S	+	UK	ERS347021
C00005885	2117-p1	07-Sep-09	1	N	2	R	S	+	UK	ERS227386
C00005886	2141-p1	18-Sep-09	1	N	2	R	S	+	UK	ERS347022
C00005887	2143-p1	18-Sep-09	1	Y	2	R	S	+	UK	ERS347023
C00005888	2158-p1	30-Sep-09	1	N	2	R	S	+	UK	SRR3339963
C00005889	2189-p1	20-Oct-09	1	Y	2	R	S	+	UK	ERS347024
C00005890	2201-p1	20-Oct-09	1	N	2	R	S	+	UK	ERS347025
C00005892	2212-p1	29-Oct-09	1	N	2	R	S	+	UK	ERS347027
C00005894	2259-p1	21-Nov-09	1	N	2	R	S	+	UK	ERS347029
C00005895	2261-p1	25-Nov-09	1	Y	2	R	S	+	UK	ERS347030
C00005896	2336a-p1	04-Jan-10	1	N	2	R	S	+	UK	ERS347031
C00005897	2348-p1	13-Jan-10	1	N	2	R	S	+	UK	ERS227389
C00005898	2353-p1	15-Jan-10	1	Y	2	R	S	+	UK	ERS347032
C00005899	2354-p1	15-Jan-10	1	N	2	R	S	+	UK	ERS347033
C00005900	2369-p1	24-Jan-10	1	N	2	R	S	+	UK	ERS347034
C00005901	2383-p1	04-Feb-10	1	Y	2	R	S	+	UK	ERS347035
C00005902	2394-p1	10-Feb-10	1	N	2	R	S	+	UK	ERS347036
C00005903	2405-p1	14-Feb-10	1	N	2	R	S	+	UK	ERS347037
C00005905	2422-p1	21-Feb-10	1	Y	2	R	S	+	UK	ERS347038

C00010622	2455-p2	12-Mar-10	1	N	2	R	S	+	UK	ERS347390
C00005906	2468-p1	21-Mar-10	1	N	2	R	S	+	UK	ERS347039
C00005907	2474-p1	23-Mar-10	1	Y	2	R	S	+	UK	ERS347040
C00005908	2608-p1	09-Jun-10	1	N	2	R	S	+	UK	ERS347041
C00007255	2712-p0	23-Aug-10	1	N	2	R	S	+	UK	ERS140991
C00021201	2741-p2	25-Sep-10	1	Y	2	R	S	+	UK	SRR3334207
C00008118	2833-p1	16-Dec-10	1	N	2	R	S	+	UK	ERS347266
C00007797	3020-p1	15-Jun-11	1	N	2	R	S	+	UK	ERS347212
C00007789	3050-p1	27-Jul-11	1	Y	2	R	S	+	UK	SRR3340017
C00007778	3057-p1	04-Aug-11	1	N	2	R	S	+	UK	SRR3334141
C00007864	3080-p1	25-Aug-11	1	N	2	R	S	+	UK	SRR3332995
C00009870	3094-p1	16-Sep-11	1	Y	2	R	S	+	UK	SRR3333623
C00021219	3887-p1	02-Apr-13	1	N	2	R	S	+	UK	SRR3334466
C00021148	33-p1	29-Sep-06	2	Y	1	S	S	+	UK	SRR3333108
6ac7ad35-92ab-4eef-a7fb-faa31676a739	f6d6f7_2	22-Nov-06	2	Y	1	S	S	+	UK	SRR3317182
C00021157	102-p1	23-Nov-06	2	Y	1	S	S	+	UK	SRR3334147
C00021160	106-p1	26-Nov-06	2	Y	1	S	S	+	UK	SRR3334103
C00021163	113-p1	04-Dec-06	2	Y	1	S	S	+	UK	SRR3333674
C00021173	148-p1	07-Dec-06	2	Y	1	S	S	+	UK	SRR3334224
C00021176	161-p1	28-Jan-07	2	Y	1	S	S	+	UK	SRR3332982
C00006369	255-p1	14-Mar-07	2	Y	1	S	S	+	UK	SRR3333542
C00006371	256-p1	15-Mar-07	2	Y	1	S	S	+	UK	SRR3333671
C00006373	288-p1	08-Apr-07	2	Y	1	S	S	+	UK	SRR3333342
C00000020	Oxf312-2	18-Apr-07	2	Y	1	S	S	+	UK	SRR3333357
C00006374	404-p1	31-May-07	2	Y	1	R	S	+	UK	SRR3333087
C00006375	410-p1	06-Jun-07	2	Y	1	S	S	+	UK	SRR3333563
C00006376	415-p1	08-Jun-07	2	Y	1	S	S	+	UK	SRR3334426
C00000098	Oxf709	25-Sep-07	2	Y	1	S	S	+	UK	ERS139373
C00006378	713-p1	25-Sep-07	2	Y	1	S	S	+	UK	ERS347164
C00010623	788-p2	30-Oct-07	2	Y	1	S	S	+	UK	ERS347391

C00010624	791-p2	02-Nov-07	2	Y	1	S	S	+	UK	ERS347392
C00006385	800-p1	07-Nov-07	2	Y	1	S	S	+	UK	ERS227360
C00000217	Oxf807	10-Nov-07	2	Y	1	S	S	+	UK	ERS149862
C00006388	866-p1	02-Dec-07	2	Y	1	S	S	+	UK	ERS347170
C00006389	880-p1	06-Dec-07	2	Y	1	S	S	+	UK	ERS347171
C00010627	1067-p2	18-Feb-08	2	Y	1	S	S	+	UK	ERS347394
C00010629	1190-p2	13-Apr-08	2	Y	1	S	S	+	UK	ERS347396
C00006395	1209-p1	19-Apr-08	2	Y	1	S	S	+	UK	ERS347173
C00000517	Oxf1284	14-May-08	2	Y	1	S	S	+	UK	ERS346586
C00001472	Oxf1300a	22-May-08	2	Y	1	S	S	+	UK	SRR3334017
C00006401	1308a-p1	29-May-08	2	Y	1	S	S	+	UK	ERS347179
C00006402	1329-p1	04-Jun-08	2	Y	1	S	S	+	UK	ERS227366
C00006403	1335-p1	06-Jun-08	2	Y	1	S	S	+	UK	ERS347180
C00006405	1378-p1	05-Jul-08	2	Y	1	S	S	+	UK	ERS347182
C00001475	Oxf1384	08-Jul-08	2	Y	1	S	S	+	UK	ERS149924
C00003979	Oxf1382-p1	08-Jul-08	2	Y	1	S	S	+	UK	ERS346616
C00006406	1386-p1	09-Jul-08	2	Y	1	S	S	+	UK	ERS347183
C00006408	1399-p1	15-Jul-08	2	Y	1	S	S	+	UK	ERS347185
C00006409	1411-p1	23-Jul-08	2	Y	1	S	S	+	UK	ERS347186
C00006411	1415-p1	26-Jul-08	2	Y	1	S	S	+	UK	ERS347188
C00006412	1416-p1	26-Jul-08	2	Y	1	S	S	+	UK	ERS347189
C00006413	1425-p1	30-Jul-08	2	Y	1	S	S	+	UK	ERS347190
C00006415	1459-p1	09-Aug-08	2	Y	1	S	S	+	UK	ERS347192
C00001466	Oxf1435	13-Aug-08	2	Y	1	S	S	+	UK	ERS149920
C00001476	Oxf1443	15-Aug-08	2	Y	1	S	S	+	UK	ERS149925
C00001477	Oxf1447	18-Aug-08	2	Y	1	S	S	+	UK	ERS149926
C00006414	1451-p1	19-Aug-08	2	Y	1	S	S	+	UK	ERS347191
C00003980	Oxf1457-p1	25-Aug-08	2	Y	1	S	S	+	UK	ERS346617
C00006417	1475-p1	05-Sep-08	2	Y	1	S	S	+	UK	ERS139420
C00006418	1536-p1	04-Oct-08	2	Y	1	S	S	+	UK	ERS347194
C00000081	Oxf1540	07-Oct-08	2	Y	1	S	S	+	UK	ERS149804

C00001535	Oxf1559	17-Oct-08	2	Y	1	S	S	+	UK	ERS149956
C00000475	Oxf1574	27-Oct-08	2	Y	1	S	S	+	UK	ERS149868
C00006421	1593-p1	09-Nov-08	2	Y	1	S	S	+	UK	ERS347197
C00003989	Oxf1616-p1	16-Nov-08	2	Y	1	S	S	+	UK	ERS346621
C00006238	1664-p1	12-Dec-08	2	Y	1	S	S	+	UK	ERS347083
C00006239	1676-p1	16-Dec-08	2	Y	1	S	S	+	UK	ERS347084
C00006240	1691-p1	05-Jan-09	2	Y	1	S	S	+	UK	ERS347085
C00000025	Oxf1701-1	11-Jan-09	2	Y	1	S	S	+	UK	SRR3333232
C00006011	1708-p1	12-Jan-09	2	Y	1	S	S	+	UK	SRR3334232
C00010630	1713-p2	14-Jan-09	2	Y	1	S	S	+	UK	ERS347397
C00010631	1764-p2	09-Feb-09	2	Y	1	S	S	+	UK	ERS347398
C00006246	1827-p1	10-Mar-09	2	Y	1	S	S	+	UK	ERS347089
C00006248	1853-p1	02-Apr-09	2	Y	1	S	S	+	UK	ERS347090
C00006250	1873-p1	17-Apr-09	2	Y	1	S	S	+	UK	ERS347091
C00006251	1887-p1	27-Apr-09	2	Y	1	S	S	+	UK	ERS347092
C00006252	1889-p1	30-Apr-09	2	Y	1	S	S	+	UK	ERS347093
C00009891	1891-p1	30-Apr-09	2	Y	1	S	S	+	UK	SRR3334139
C00006254	1905-p1	12-May-09	2	Y	1	S	S	+	UK	ERS347095
C00000510	Oxf1910	15-May-09	2	Y	1	S	S	+	UK	ERS149885
C00006255	1921-p1	26-May-09	2	Y	1	S	S	+	UK	ERS347096
C00006257	1930-p1	30-May-09	2	Y	1	S	S	+	UK	ERS347097
C00006259	1949-p1	04-Jun-09	2	Y	1	S	S	+	UK	ERS347099
C00006264	1984-p1	04-Jul-09	2	Y	1	S	S	+	UK	ERS347102
C00006265	1987-p1	04-Jul-09	2	Y	1	S	S	+	UK	ERS347103
C00006269	2004-p1	14-Jul-09	2	Y	1	S	S	+	UK	ERS347105
C00005707	2027a-p1	27-Jul-09	2	Y	1	S	S	+	UK	ERS346886
C00005955	2052a-p2	10-Aug-09	2	Y	1	S	S	+	UK	ERS347051
C00006272	2067-p1	15-Aug-09	2	Y	1	S	S	+	UK	ERS347107
C00010633	2081a-p2	20-Aug-09	2	Y	1	S	S	+	UK	ERS347400
C00004017	Oxf2087b-p1	22-Aug-09	2	Y	1	S	S	+	UK	ERS346633
C00006274	2095-p1	26-Aug-09	2	Y	1	S	S	+	UK	ERS347108

C00006280	2130-p1	12-Sep-09	2	Y	1	S	S	+	UK	ERS347113
C00006290	2188-p1	20-Oct-09	2	Y	1	S	S	+	UK	ERS227413
C00006297	2235-p1	09-Nov-09	2	Y	1	S	S	+	UK	ERS347125
C00006306	2272-p1	03-Dec-09	2	Y	1	S	S	+	UK	ERS347130
C00006308	2279-p1	03-Dec-09	2	Y	1	S	S	+	UK	ERS347132
C00006310	2286-p1	08-Dec-09	2	Y	1	S	S	+	UK	ERS347134
C00006311	2300-p1	17-Dec-09	2	Y	1	S	S	+	UK	ERS347135
C00009951	2330-p1	02-Jan-10	2	Y	1	S	S	+	UK	SRR3333810
C00006317	2333-p1	03-Jan-10	2	Y	1	S	S	+	UK	ERS347140
C00006319	2340-p1	07-Jan-10	2	Y	1	S	S	+	UK	ERS347142
C00006321	2363-p1	20-Jan-10	2	Y	1	S	S	+	UK	ERS347144
C00006322	2366-p1	21-Jan-10	2	Y	1	S	S	+	UK	ERS347145
C00005957	2390-p1	08-Feb-10	2	Y	1	S	S	+	UK	ERS347052
C00005958	2392-p1	08-Feb-10	2	Y	1	S	S	+	UK	ERS347053
C00005959	2395-p1	10-Feb-10	2	Y	1	S	S	+	UK	ERS347054
C00005964	2436-p1	01-Mar-10	2	Y	1	S	S	+	UK	ERS227378
C00008328	2445-p1	05-Mar-10	2	Y	1	S	S	+	UK	ERS347349
C00005969	2466-p1	20-Mar-10	2	Y	1	S	S	+	UK	ERS347059
C00005970	2467-p1	21-Mar-10	2	Y	1	S	S	+	UK	ERS227377
C00005976	2534-p1	22-Apr-10	2	Y	1	S	S	+	UK	ERS347063
C00005978	2542-p1	30-Apr-10	2	Y	1	S	S	+	UK	ERS347065
C00005980	2547-p1	03-May-10	2	Y	1	S	S	+	UK	ERS347067
C00006742	2587-p1	25-May-10	2	Y	1	S	S	+	UK	ERS347200
C00005985	2622-p1	16-Jun-10	2	Y	1	S	S	+	UK	ERS347070
C00005987	2640-p1	07-Jul-10	2	Y	1	S	S	+	UK	ERS347071
C00008282	2647-p1	12-Jul-10	2	Y	1	S	S	+	UK	ERS347315
C00005990	2678-p1	23-Jul-10	2	Y	1	S	S	+	UK	ERS227396
C00008078	2730-p1	12-Sep-10	2	Y	1	S	S	+	UK	ERS347240
C00008320	2753-p1	03-Oct-10	2	Y	1	S	S	+	UK	ERS347343
C00008334	2806-p1	24-Nov-10	2	Y	1	S	S	+	UK	ERS347354
C00008299	2811-p1	30-Nov-10	2	Y	1	S	S	+	UK	ERS347326

C00008264	2823-p1	09-Dec-10	2	Y	1	S	S	+	UK	ERS347302
C00008082	2828-p1	14-Dec-10	2	Y	1	S	S	+	UK	ERS347242
C00008288	2840-p1	29-Dec-10	2	Y	1	S	S	+	UK	ERS347319
C00008314	2864-p1	29-Jan-11	2	Y	1	S	S	+	UK	ERS347338
C00008086	2894-p1	04-Mar-11	2	Y	1	S	S	+	UK	ERS347246
C00007978	2923-p1	03-Apr-11	2	Y	1	S	S	+	UK	SRR3334212
C00008075	2936-p1	25-Apr-11	2	Y	1	S	S	+	UK	SRR3333721
C00007775	3034-p1	05-Jul-11	2	Y	1	S	S	+	UK	SRR3340247
C00007811	3037-p1	11-Jul-11	2	Y	1	S	S	+	UK	SRR3334201
C00007863	3072-p1	12-Aug-11	2	Y	1	S	S	+	UK	SRR3333019
C00007839	3070-p1	15-Aug-11	2	Y	1	S	S	+	UK	SRR3333660
C00007828	3077-p1	17-Aug-11	2	Y	1	S	S	+	UK	SRR3333079
C00009836	3115-p1	02-Oct-11	2	Y	1	S	S	+	UK	SRR3333040
C00009826	3149-p1	21-Nov-11	2	Y	1	S	R	+	UK	SRR3333333
C00009851	3162-p1	08-Dec-11	2	Y	1	S	S	+	UK	SRR3333607
C00009828	3168-p1	16-Dec-11	2	Y	1	S	S	+	UK	SRR3333395
C00009867	3172-p1	22-Dec-11	2	Y	1	S	S	+	UK	SRR3333999
C00011217	3175-p1	28-Dec-11	2	Y	1	S	S	+	UK	SRR3333268
C00011241	3177-p1	29-Dec-11	2	Y	1	S	S	+	UK	SRR3334133
b20a5db0-b14c-4462-b32b-0b6455822d4c	f6d6f7_28	05-Jan-12	2	Y	1	S	S	+	UK	SRR3317190
C00011232	3201-p1	06-Feb-12	2	Y	1	S	S	+	UK	SRR3340049
C00011790	3227-p1	16-Mar-12	2	Y	1	S	S	+	UK	SRR3333290
C00013822	3380-p1	10-Apr-12	2	Y	1	S	S	+	UK	SRR3332983
C00012667	3259-p1	13-Apr-12	2	Y	1	S	S	+	UK	SRR3340066
C00012697	3297-p1	10-May-12	2	Y	1	S	S	+	UK	SRR3340212
C00012730	3344-p1	19-May-12	2	Y	1	S	S	+	UK	SRR3333670
C00012733	3349-p1	22-May-12	2	Y	1	S	S	+	UK	SRR3332985
C00013846	3411-p1	01-Jun-12	2	Y	1	S	S	+	UK	SRR3334186
C00013873	3438-p1	05-Jun-12	2	Y	1	S	R	+	UK	SRR3333745
C00014330	3505-p1	03-Aug-12	2	Y	1	S	S	+	UK	SRR3333547
C00014374	3566-p1	20-Aug-12	2	Y	1	S	S	+	UK	SRR3333421

C00015473	3604-p1	11-Sep-12	2	Y	1	S	S	+	UK	SRR3334414
C00015456	3584-p1	21-Sep-12	2	Y	1	S	S	+	UK	SRR3332972
C00015468	3598-p1	24-Sep-12	2	Y	1	S	S	+	UK	SRR3340249
C00016342	3680-p1	28-Oct-12	2	Y	1	S	S	+	UK	SRR3333468
C00016359	3702-p1	06-Nov-12	2	Y	1	S	S	+	UK	SRR3333462
C00020113	3737-p1	28-Nov-12	2	Y	1	S	S	+	UK	SRR3334053
C00020125	3752-p1	07-Dec-12	2	Y	1	S	S	+	UK	SRR3334085
C00020140	3768-p1	06-Jan-13	2	Y	1	S	S	+	UK	SRR3333085
C00020395	3815-p1	31-Jan-13	2	Y	1	S	S	+	UK	SRR3333368
C00020396	3817-p1	07-Feb-13	2	Y	1	S	S	+	UK	SRR3333553
C00020407	3834-p1	26-Feb-13	2	Y	1	S	S	+	UK	SRR3333549
C00020240	3891-p1	04-Apr-13	2	Y	1	S	S	+	UK	SRR3333734
C00012861	19-p1	21-Sep-06	3	Y	1	R	S	+	UK	SRR3333711
C00012862	20-p1	23-Sep-06	3	Y	1	R	S	+	UK	SRR3333374
C00012863	195-p1	12-Feb-07	3	Y	1	R	S	+	UK	SRR3340219
C00006005	338-p1	30-Apr-07	3	Y	1	R	S	+	UK	ERS227387
f2146f66-2189-4a0d-b597-c84b164d1847	f6d6f7_5	01-May-07	3	Y	1	R	S	+	UK	SRR3317204
C00006006	346-p1	02-May-07	3	Y	1	R	S	+	UK	ERS243578
C00003961	Oxf374-p1	15-May-07	3	Y	1	R	S	+	UK	ERS243579
C00006019	428-p1	14-Jun-07	3	Y	1	S	S	+	UK	ERS139415
C00006022	458-p1	03-Jul-07	3	Y	1	S	S	+	UK	ERS227370
C00006435	528-p1	27-Jul-07	3	Y	1	R	S	+	UK	ERS243580
C00006326	598-p1	21-Aug-07	3	Y	1	S	S	+	UK	ERS243581
C00004369	688-p1	18-Sep-07	3	Y	1	R	S	+	UK	ERS243582
C00008986	697m-p1	19-Sep-07	3	N	1	R	S	+	UK	ERS227505
C00004374	738-p1	10-Oct-07	3	Y	1	R	S	+	UK	ERS243584
C00000057	Oxf741	15-Oct-07	3	Y	1	R	S	+	UK	ERS149785
C00000059	Oxf746b	16-Oct-07	3	Y	1	R	S	+	UK	ERS149786
C00004375	771-p1	24-Oct-07	3	Y	1	R	S	+	UK	ERS243585
C00004376	792-p1	03-Nov-07	3	Y	1	R	S	+	UK	ERS243586

C00004377	848-p1	26-Nov-07	3	Y	1	R	S	+	UK	ERS243587
C00004380	943-p1	02-Jan-08	3	Y	1	R	S	+	UK	ERS243588
C00004383	973-p1	14-Jan-08	3	Y	1	S	S	+	UK	ERS243589
C00006358	1003-p1	21-Jan-08	3	N	1	S	S	-	UK	ERS347155
C00004385	1009-p1	24-Jan-08	3	Y	1	S	S	+	UK	ERS243590
C00004386	1028-p1	30-Jan-08	3	Y	1	R	S	+	UK	ERS243591
C00009977	1030-p2	30-Jan-08	3	Y	1	R	S	+	UK	ERS347385
C00005001	1091-p1	29-Feb-08	3	Y	1	R	S	+	UK	SRR3334134
C00004392	1107-p1	04-Mar-08	3	N	1	S	S	-	UK	ERS346742
C00004394	1172-p1	04-Apr-08	3	Y	1	R	S	+	UK	ERS243594
C00004395	1199-p1	15-Apr-08	3	Y	1	S	S	+	UK	ERS243595
C00004397	1230-p1	24-Apr-08	3	Y	1	R	S	+	UK	ERS243596
C00005022	1241-p1	30-Apr-08	3	Y	1	R	S	+	UK	ERS243597
C00004403	1338-p1	07-Jun-08	3	Y	1	R	S	+	UK	ERS243598
C00004406	1390-p1	11-Jul-08	3	Y	1	R	S	+	UK	ERS243599
C00004408	1397-p1	15-Jul-08	3	Y	1	S	S	+	UK	ERS243600
C00004410	1420-p1	29-Jul-08	3	Y	1	R	S	+	UK	ERS243601
C00004412	1474-p1	05-Sep-08	3	Y	1	R	S	+	UK	ERS243602
C00004985	1537-p1	04-Oct-08	3	Y	1	R	S	+	UK	ERS243603
C00004987	1560a-p1	21-Oct-08	3	Y	1	S	S	+	UK	ERS243621
C00004988	1638-p1	28-Nov-08	3	Y	1	S	S	+	UK	ERS243604
C00004991	1770-p1	10-Feb-09	3	Y	1	R	S	+	UK	ERS243605
C00004993	1792-p1	24-Feb-09	3	Y	1	S	R	+	UK	ERS243606
C00004997	1829-p1	26-Feb-09	3	Y	1	S	S	+	UK	ERS243609
C00004995	1813-p1	05-Mar-09	3	Y	1	R	S	+	UK	ERS243607
C00004001	Oxf1819b-p1	08-Mar-09	3	Y	1	S	R	+	UK	ERS243622
C00004996	1823-p1	09-Mar-09	3	Y	1	S	S	+	UK	ERS243608
C00000482	Oxf1876	20-Apr-09	3	Y	1	S	R	+	UK	SRR3332967
C00000520	Oxf1880	23-Apr-09	3	Y	1	S	S	+	UK	ERS149891
C00004998	1881-p1	23-Apr-09	3	Y	1	R	S	+	UK	ERS243610
C00005005	2056-p1	12-Aug-09	3	Y	1	S	S	+	UK	ERS243611

C00005006	2066-p1	17-Aug-09	3	Y	1	S	S	+	UK	ERS243612
C00005011	2219-p1	03-Nov-09	3	Y	1	S	S	+	UK	ERS243613
C00005654	2307-p1	21-Dec-09	3	Y	1	S	S	+	UK	SRR3334204
C00005017	2314a-p1	24-Dec-09	3	Y	1	S	S	+	UK	ERS243623
C00005018	2316-p1	25-Dec-09	3	Y	1	S	S	+	UK	ERS243615
C00005023	2488-p1	30-Mar-10	3	Y	1	S	S	+	UK	ERS243616
C00005025	2532-p1	21-Apr-10	3	Y	1	S	S	+	UK	ERS243617
C00008293	2538-p1	26-Apr-10	3	N	1	S	S	-	UK	ERS347322
C00008317	2554-p1	06-May-10	3	N	1	S	S	+	UK	ERS243625
C00005028	2572-p1	15-May-10	3	Y	1	S	S	+	UK	ERS243618
C00005032	2598-p1	02-Jun-10	3	Y	1	S	S	+	UK	ERS227397
C00005033	2613-p1	11-Jun-10	3	Y	1	S	S	+	UK	ERS243619
C00008270	2639-p1	07-Jul-10	3	Y	1	S	S	+	UK	ERS243626
C00005036	2679-p1	24-Jul-10	3	Y	1	R	S	+	UK	SRR3334064
C00007249	2689-p0	01-Aug-10	3	Y	1	S	S	+	UK	ERS140986
C00005038	2692a-p1	05-Aug-10	3	Y	1	R	S	+	UK	SRR3333781
C00010635	2696-p2	08-Aug-10	3	Y	1	R	S	+	UK	ERS347402
C00007254	2706-p0	13-Aug-10	3	Y	1	R	S	+	UK	ERS140990
C00008124	2705-p1	13-Aug-10	3	Y	1	S	S	+	UK	ERS243630
C00008125	2723-p1	02-Sep-10	3	Y	1	S	S	+	UK	ERS243632
C00008954	2733-p2	14-Sep-10	3	Y	1	S	S	+	UK	ERS243633
C00008285	2758-p1	09-Oct-10	3	Y	1	S	S	+	UK	ERS243634
C00008071	2837-p1	26-Dec-10	3	Y	1	S	S	+	UK	ERS243635
C00008001	2915-p1	30-Mar-11	3	Y	1	S	S	+	UK	ERS243636
C00008159	2944-p1	08-May-11	3	Y	1	S	S	+	UK	ERS243637
C00007819	3006-p1	02-Jun-11	3	Y	1	S	S	+	UK	ERS243639
C00007821	3022-p1	17-Jun-11	3	Y	1	S	S	+	UK	ERS243640
C00011182	3180-p1	02-Jan-12	3	Y	1	S	S	+	UK	SRR3340062
C00009463	3249-p1	16-Apr-12	3	Y	1	R	S	+	UK	SRR3334008
C00012691	3286-p1	26-Apr-12	3	Y	1	R	S	+	UK	SRR3334036
C00012712	3322-p1	06-May-12	3	Y	1	S	S	+	UK	SRR3333365

C00012729	3343-p1	12-May-12	3	Y	1	S	S	+	UK	SRR3333121
C00012734	3350-p1	21-May-12	3	Y	1	S	S	+	UK	SRR3333314
C00013872	3437-p1	05-Jun-12	3	Y	1	S	S	+	UK	SRR3334083
C00021215	3880-p1	24-Mar-13	3	Y	1	S	S	+	UK	SRR3340008
C00006377	553-p1	03-Aug-07	4	N	1	S	S	+	UK	SRR3333382
C00000038	Oxf1145-2	19-Mar-08	4	N	1	S	S	+	UK	SRR3333417
C00005954	1365-p2	27-Jun-08	4	N	1	S	S	+	UK	ERS347050
C00005973	2489-p1	30-Mar-10	4	N	1	S	S	+	UK	ERS347061
C00014306	3235-p1	27-Mar-12	4	N	1	S	S	+	UK	SRR3333717
C00001539	Oxf43	03-Oct-06	5	Y	3	S	S	+	UK	ERS149958
C00021168	134-p1	11-Jan-07	5	Y	3	S	S	+	UK	SRR3334042
C00001501	Oxf158	25-Jan-07	5	Y	3	S	S	+	UK	ERS149941
C00006017	408-p1	31-May-07	5	Y	3	S	S	+	UK	SRR3333110
C00006021	446a-p1	19-Jun-07	5	Y	3	S	S	+	UK	SRR3333257
C00000072	Oxf476	08-Jul-07	5	Y	3	S	S	+	UK	ERS149796
C00004371	716-p1	27-Sep-07	5	Y	3	S	S	+	UK	ERS346732
C00004372	727-p1	05-Oct-07	5	Y	3	S	S	+	UK	ERS346733
C00004378	850-p1	27-Nov-07	5	Y	3	S	S	+	UK	ERS346734
C00004379	926-p1	28-Dec-07	5	Y	3	S	S	+	UK	ERS346735
C00009973	935-p1	31-Dec-07	5	Y	3	S	S	+	UK	ERS347382
C00004381	970-p1	14-Jan-08	5	Y	3	S	S	+	UK	ERS346736
C00004384	1007-p1	23-Jan-08	5	Y	3	S	S	+	UK	ERS346738
C00004388	1033-p1	31-Jan-08	5	Y	3	S	S	+	UK	ERS346739
C00004389	1071-p1	19-Feb-08	5	Y	3	S	S	+	UK	ERS346740
C00004393	1139-p1	13-Mar-08	5	Y	3	S	S	+	UK	ERS346743
C00004396	1229-p1	24-Apr-08	5	Y	3	S	S	+	UK	ERS346744
C00004399	1256-p1	01-May-08	5	Y	3	S	S	+	UK	ERS346745
C00004400	1303a-p1	27-May-08	5	Y	3	S	S	+	UK	ERS346746
C00004401	1322-p1	03-Jun-08	5	Y	3	S	S	+	UK	ERS346747
C00004404	1346-p1	11-Jun-08	5	Y	3	S	S	+	UK	ERS346749

C00004405	1349-p1	13-Jun-08	5	Y	3	S	S	+	UK	ERS346750
C00001636	Oxf1355	20-Jun-08	5	Y	3	S	S	+	UK	ERS150022
C00004411	1465-p1	30-Aug-08	5	Y	3	S	S	+	UK	ERS346752
C00004983	1485-p1	10-Sep-08	5	Y	3	S	S	+	UK	ERS216157
C00007805	1482-p2	10-Sep-08	5	Y	3	S	S	+	UK	ERS347215
C00004984	1518-p1	24-Sep-08	5	Y	3	S	S	+	UK	ERS346810
C00005007	1523-p2	25-Sep-08	5	Y	3	S	S	+	UK	SRR3333322
C00004989	1672-p1	16-Dec-08	5	Y	3	S	S	+	UK	ERS346811
C00004990	1682-p1	22-Dec-08	5	Y	3	S	S	+	UK	ERS346812
C00004992	1783-p1	18-Feb-09	5	Y	3	S	S	+	UK	ERS346813
C00001503	Oxf1895	03-May-09	5	Y	3	S	S	+	UK	ERS149942
C00004999	1977a-p1	30-Jun-09	5	Y	3	S	S	+	UK	ERS346814
C00005000	1996a-p1	09-Jul-09	5	Y	3	S	S	+	UK	ERS346815
C00005002	2005-p1	14-Jul-09	5	Y	3	S	S	+	UK	ERS346816
C00005003	2026-p1	27-Jul-09	5	Y	3	S	S	+	UK	ERS346817
C00005004	2034a-p1	31-Jul-09	5	Y	3	S	S	+	UK	ERS346818
C00001504	Oxf2154	27-Sep-09	5	Y	3	S	S	+	UK	ERS149943
C00005009	2172a-p1	13-Oct-09	5	Y	3	S	S	+	UK	ERS346820
C00005010	2183-p1	17-Oct-09	5	Y	3	S	S	+	UK	ERS139411
C00005012	2233-p1	08-Nov-09	5	Y	3	R	S	+	UK	ERS346821
C00005014	2264-p1	27-Nov-09	5	Y	3	S	S	+	UK	ERS346822
C00005015	2282-p1	07-Dec-09	5	Y	3	S	S	+	UK	ERS346823
C00008268	2365-p1	20-Jan-10	5	Y	3	S	S	+	UK	ERS347305
C00005020	2378-p1	01-Feb-10	5	Y	3	S	S	+	UK	ERS346825
C00005021	2381-p1	02-Feb-10	5	Y	3	S	S	+	UK	ERS227416
C00001505	Oxf2408	17-Feb-10	5	Y	3	S	S	+	UK	ERS149944
C00008280	2416-p1	20-Feb-10	5	Y	3	S	S	+	UK	ERS347313
C00005024	2490-p1	02-Apr-10	5	Y	3	S	S	+	UK	ERS346826
C00005026	2546a-p1	02-May-10	5	Y	3	S	S	+	UK	ERS346827
C00005027	2561-p1	11-May-10	5	Y	3	S	S	+	UK	ERS346828
C00007829	2565-p2	12-May-10	5	Y	3	S	S	+	UK	ERS227395

C00005029	2575-p1	16-May-10	5	Y	3	S	S	+	UK	ERS346829
C00005030	2577-p1	18-May-10	5	Y	3	S	S	+	UK	ERS346830
C00005031	2580-p1	18-May-10	5	Y	3	S	S	+	UK	ERS346831
C00005035	2661-p1	15-Jul-10	5	Y	3	S	S	+	UK	ERS346832
C00008101	2717-p1	29-Aug-10	5	Y	3	S	S	+	UK	ERS347256
C00008272	2748-p1	28-Sep-10	5	Y	3	S	S	+	UK	ERS347307
C00008344	2755-p1	04-Oct-10	5	Y	3	S	S	+	UK	ERS347363
C00008139	2778-p1	27-Oct-10	5	Y	3	S	S	+	UK	ERS347276
C00008092	2787-p1	07-Nov-10	5	Y	3	S	S	+	UK	ERS347249
C00008116	2789-p1	10-Nov-10	5	Y	3	S	S	+	UK	ERS347265
C00008128	2793-p1	13-Nov-10	5	Y	3	S	S	+	UK	ERS347270
C00008074	2893-p1	25-Feb-11	5	Y	3	S	S	+	UK	ERS347238
C00007779	3065-p1	11-Aug-11	5	Y	3	S	S	+	UK	SRR3333299
C00007791	3066-p1	11-Aug-11	5	Y	3	S	S	+	UK	SRR3334424
C00007815	3068-p1	12-Aug-11	5	Y	3	S	S	+	UK	SRR3333738
C00010639	3109-p1	25-Sep-11	5	Y	3	S	S	+	UK	SRR3333055
C00010644	3121-p1	12-Oct-11	5	Y	3	S	S	+	UK	SRR3340019
C00009813	3138-p1	14-Nov-11	5	Y	3	S	S	+	UK	SRR3333740
C00011196	3198-p1	04-Feb-12	5	Y	3	S	S	+	UK	SRR3333776
C00011784	3221-p1	29-Feb-12	5	Y	3	S	S	+	UK	SRR3333567
C00013815	3366-p1	23-Apr-12	5	Y	3	S	S	+	UK	SRR3333752
C00012698	3298-p1	10-May-12	5	Y	3	S	S	+	UK	SRR3333764
C00012735	3352-p1	15-May-12	5	Y	3	S	S	+	UK	SRR3339990
C00013836	3399-p1	23-May-12	5	Y	3	S	S	+	UK	SRR3333280
C00020129	3757-p1	13-Dec-12	5	Y	3	S	S	+	UK	SRR3339980
C00020130	3758-p1	28-Dec-12	5	Y	3	S	S	+	UK	SRR3333574
C00020420	3852-p1	08-Mar-13	5	Y	3	S	S	+	UK	SRR3333372
C00021146	27-p1	25-Sep-06	6	Y	1	S	S	+	UK	SRR3334107
C00021165	128-p1	30-Dec-06	6	Y	1	S	S	+	UK	SRR3333589
C00000032	Oxf165-2	30-Jan-07	6	Y	1	S	S	+	UK	SRR3333600

C00021177	164-p1	30-Jan-07	6	Y	1	S	S	+	UK	SRR3333475
C00028682	167-p1	30-Jan-07	6	Y	1	S	S	+	UK	SRR3333460
18bcc782-b42c-43f7-b2ea-362040682ba8	f6d6f7_3	17-Feb-07	6	Y	1	S	S	+	UK	SRR3317167
C00021187	207-p1	20-Feb-07	6	Y	1	S	S	+	UK	SRR3333239
C00001642	Oxf221	04-Mar-07	6	Y	1	S	S	+	UK	ERS150027
1ba21b34-3a16-4c75-bef7-050311c993ae	f6d6f7_6	07-May-07	6	Y	1	S	S	+	UK	SRR3317169
C00006012	379-p1	22-May-07	6	Y	1	S	S	+	UK	ERS243646
C00006013	389-p1	29-May-07	6	Y	1	S	S	+	UK	ERS243647
C00002742	Oxf694-p1	19-Sep-07	6	Y	1	S	S	+	UK	ERS150089
C00002743	Oxf695-p1	20-Sep-07	6	Y	1	S	S	+	UK	ERS150090
C00008974	703m-p1	24-Sep-07	6	Y	1	S	S	+	UK	ERS227501
C00002494	Oxf762	21-Oct-07	6	Y	1	S	S	+	UK	ERS150040
C00002495	Oxf780	25-Oct-07	6	Y	1	S	S	+	UK	ERS150041
C00002497	Oxf876	06-Dec-07	6	Y	1	S	S	+	UK	ERS150043
C00002498	Oxf890	11-Dec-07	6	Y	1	S	S	+	UK	ERS150044
C00002499	Oxf928	28-Dec-07	6	Y	1	S	S	+	UK	ERS150045
C00002500	Oxf942	01-Jan-08	6	Y	1	R	S	+	UK	ERS243648
C00002501	Oxf967	12-Jan-08	6	Y	1	S	S	+	UK	ERS150046
C00002502	Oxf1075	21-Feb-08	6	Y	1	S	S	+	UK	ERS150047
C00002506	Oxf1132	13-Mar-08	6	Y	1	S	S	+	UK	ERS150050
C00000222	Oxf1140	15-Mar-08	6	Y	1	S	S	+	UK	ERS149866
C00002507	Oxf1141	17-Mar-08	6	Y	1	S	S	+	UK	ERS150051
C00002508	Oxf1179a	07-Apr-08	6	Y	1	S	S	+	UK	ERS150052
C00002509	Oxf1236	28-Apr-08	6	Y	1	S	S	+	UK	ERS150053
C00002510	Oxf1272	06-May-08	6	Y	1	S	S	+	UK	ERS150054
C00002511	Oxf1282	09-May-08	6	Y	1	S	S	+	UK	ERS150055
C00001464	Oxf1325	05-Jun-08	6	Y	1	S	S	+	UK	ERS149918
C00006361	1327-p1	05-Jun-08	6	Y	1	S	S	+	UK	ERS227426
C00002512	Oxf1340	08-Jun-08	6	Y	1	S	S	+	UK	ERS150056
C00005949	1354-p1	19-Jun-08	6	Y	1	S	S	+	UK	ERS243649
C00002513	Oxf1391	10-Jul-08	6	Y	1	S	S	+	UK	ERS150057

C00002514	Oxf1400a	15-Jul-08	6	Y	1	S	S	+	UK	ERS150058
C00002517	Oxf1410	23-Jul-08	6	Y	1	S	S	+	UK	SRR3333293
C00002796	Oxf1456-p1	23-Aug-08	6	Y	1	S	S	+	UK	ERS150139
C00002518	Oxf1466	31-Aug-08	6	Y	1	S	S	+	UK	ERS150060
C00002519	Oxf1483	09-Sep-08	6	Y	1	S	S	+	UK	ERS150061
C00002520	Oxf1515	24-Sep-08	6	Y	1	S	S	+	UK	ERS150062
C00002521	Oxf1534	02-Oct-08	6	Y	1	S	S	+	UK	ERS150063
C00002808	Oxf1545a-p1	08-Oct-08	6	Y	1	S	S	+	UK	ERS150145
C00002522	Oxf1660	10-Dec-08	6	Y	1	S	S	+	UK	ERS150064
C00002523	Oxf1748	02-Feb-09	6	Y	1	S	S	+	UK	ERS150065
C00000195	Oxf1879	22-Apr-09	6	Y	1	S	S	+	UK	ERS149845
C00000076	Oxf2007	15-Jul-09	6	Y	1	S	S	+	UK	ERS149800
C00002525	Oxf2023a	22-Jul-09	6	Y	1	S	S	+	UK	ERS150067
C00002524	Oxf2020	23-Jul-09	6	Y	1	S	S	+	UK	ERS150066
C00002526	Oxf2035	01-Aug-09	6	Y	1	S	S	+	UK	ERS150068
C00002821	Oxf2060a-p1	13-Aug-09	6	Y	1	S	S	+	UK	ERS150158
C00002823	Oxf2078-p1	19-Aug-09	6	Y	1	S	S	+	UK	ERS150160
C00002824	Oxf2094-p1	25-Aug-09	6	Y	1	S	S	+	UK	ERS150161
C00002826	Oxf2110-p1	31-Aug-09	6	Y	1	S	S	+	UK	ERS150163
C00002828	Oxf2119-p1	09-Sep-09	6	Y	1	S	S	+	UK	ERS150165
C00002830	Oxf2140-p1	18-Sep-09	6	Y	1	S	S	+	UK	ERS150166
C00002832	Oxf2165a-p1	07-Oct-09	6	Y	1	S	S	+	UK	ERS150168
C00002833	Oxf2178a-p1	14-Oct-09	6	Y	1	S	S	+	UK	ERS150169
C00002834	Oxf2198-p1	24-Oct-09	6	Y	1	S	S	+	UK	ERS150170
C00002836	Oxf2248a-p1	11-Nov-09	6	Y	1	S	S	+	UK	ERS150172
C00002839	Oxf2258a-p1	21-Nov-09	6	Y	1	S	S	+	UK	ERS150175
C00002841	Oxf2278a-p1	07-Dec-09	6	Y	1	S	S	+	UK	ERS150177
C00002846	Oxf2341-p1	07-Jan-10	6	Y	1	S	S	+	UK	ERS150180
C00002847	Oxf2355-p1	16-Jan-10	6	Y	1	S	S	+	UK	ERS150181
C00002848	Oxf2357a-p1	16-Jan-10	6	Y	1	S	S	+	UK	ERS150182
C00002851	Oxf2398-p1	11-Feb-10	6	Y	1	S	S	+	UK	ERS150185

C00005039	2431-p1	27-Feb-10	6	Y	1	S	S	+	UK	ERS346833
C00002856	Oxf2444-p1	03-Mar-10	6	Y	1	S	S	+	UK	ERS150188
C00009975	2453-p1	11-Mar-10	6	Y	1	S	S	+	UK	SRR3334157
C00002858	Oxf2496-p1	06-Apr-10	6	Y	1	S	S	+	UK	ERS150190
C00002867	Oxf2530-p1	20-Apr-10	6	Y	1	S	S	+	UK	ERS150198
C00002868	Oxf2537-p1	26-Apr-10	6	Y	1	S	S	+	UK	ERS150199
C00002872	Oxf2581-p1	19-May-10	6	Y	1	S	S	+	UK	ERS150201
C00002884	Oxf2625-p1	22-Jun-10	6	Y	1	S	S	+	UK	ERS150211
C00002886	Oxf2641-p1	09-Jul-10	6	Y	1	S	S	+	UK	ERS243651
C00002887	Oxf2645-p1	12-Jul-10	6	Y	1	S	S	+	UK	ERS243652
C00002888	Oxf2646-p1	12-Jul-10	6	Y	1	S	S	+	UK	ERS243653
C00002890	Oxf2669-p1	19-Jul-10	6	Y	1	S	S	+	UK	ERS243654
C00008307	2743-p1	27-Sep-10	6	Y	1	S	S	+	UK	ERS243655
C00008273	2757-p1	08-Oct-10	6	Y	1	S	S	+	UK	ERS243656
C00008321	2762-p1	11-Oct-10	6	Y	1	S	S	+	UK	ERS243657
C00008091	2771-p1	22-Oct-10	6	Y	1	S	S	+	UK	ERS243658
C00008103	2774-p1	25-Oct-10	6	Y	1	S	S	+	UK	ERS243659
C00008081	2798-p1	18-Nov-10	6	Y	1	S	S	+	UK	ERS243661
C00008117	2803-p1	19-Nov-10	6	Y	1	S	S	+	UK	ERS243662
C00008347	2822-p1	08-Dec-10	6	Y	1	S	S	+	UK	ERS243663
C00008106	2831-p1	16-Dec-10	6	Y	1	S	S	+	UK	ERS243664
C00008312	2843-p1	03-Jan-11	6	Y	1	S	S	+	UK	ERS243665
C00008324	2845-p1	04-Jan-11	6	Y	1	S	S	+	UK	ERS243666
C00008073	2884-p1	18-Feb-11	6	Y	1	S	S	+	UK	ERS243667
C00008097	2886-p1	23-Feb-11	6	Y	1	S	S	+	UK	ERS243668
C00008158	2934-p1	19-Apr-11	6	Y	1	S	S	+	UK	SRR3334163
C00008111	2939-p1	02-May-11	6	Y	1	S	S	+	UK	ERS243669
C00008123	2940-p1	03-May-11	6	Y	1	S	S	+	UK	ERS243670
C00007810	3029-p1	28-Jun-11	6	Y	1	S	S	+	UK	ERS243680
C00007792	3074-p1	16-Aug-11	6	Y	1	S	S	+	UK	ERS243682
C00010649	3126-p1	03-Oct-11	6	Y	1	S	S	+	UK	SRR3333301

C00010645	3122-p1	12-Oct-11	6	Y	1	S	S	+	UK	SRR3339973
C00009802	3147-p1	15-Oct-11	6	Y	1	S	S	+	UK	SRR3334354
C00010651	3129-p1	24-Oct-11	6	Y	1	S	S	+	UK	SRR3340024
C00011183	3189-p1	20-Jan-12	6	Y	1	S	S	+	UK	SRR3333682
C00011219	3192-p1	27-Jan-12	6	Y	1	S	S	+	UK	SRR3340192
C00011782	3219-p1	27-Feb-12	6	Y	1	S	S	+	UK	SRR3334225
C00011788	3225-p1	13-Mar-12	6	Y	1	S	S	+	UK	SRR3333250
C00011791	3228-p1	16-Mar-12	6	Y	1	S	S	+	UK	SRR3333678
C00014302	3231-p1	22-Mar-12	6	Y	1	S	S	+	UK	SRR3333353
C00014311	3241-p1	28-Mar-12	6	Y	1	S	S	+	UK	SRR3333323
C00013809	3356-p1	03-Apr-12	6	Y	1	S	S	+	UK	SRR3339999
C00009453	3244-p1	11-Apr-12	6	Y	1	S	S	+	UK	SRR3334068
C00012668	3261-p1	20-Apr-12	6	Y	1	S	S	+	UK	SRR3333084
C00012708	3317-p1	23-Apr-12	6	Y	1	S	S	+	UK	SRR3333716
C00012682	3278-p1	27-Apr-12	6	Y	1	S	S	+	UK	SRR3334452
C00013850	3415-p1	28-May-12	6	Y	1	S	S	+	UK	SRR3333644
C00013863	3427-p1	28-May-12	6	Y	1	S	S	+	UK	SRR3333719
C00013880	3448-p1	06-Jun-12	6	Y	1	S	S	+	UK	SRR3339958
C00013896	3470-p1	17-Jun-12	6	Y	1	S	S	+	UK	SRR3334196
C00014328	3502-p1	08-Jul-12	6	Y	1	S	S	+	UK	SRR3334416
C00015470	3600-p1	21-Sep-12	6	Y	1	S	S	+	UK	SRR3333318
C00013413	3637-p1	04-Oct-12	6	Y	1	S	S	+	UK	SRR3333774
C00015520	3673-p1	21-Oct-12	6	Y	1	S	S	+	UK	SRR3340007
C00016365	3712-p1	12-Nov-12	6	Y	1	S	S	+	UK	SRR3333386
C00020122	3749b-p1	07-Dec-12	6	Y	1	S	S	+	UK	SRR3333773
C00020128	3755-p1	10-Dec-12	6	Y	1	S	S	+	UK	SRR3333362
C00020141	3769-p1	15-Dec-12	6	Y	1	S	S	+	UK	SRR3334089
C00020138	3766-p1	17-Dec-12	6	Y	1	S	S	+	UK	SRR3333269
C00020427	3859-p1	07-Mar-13	6	Y	1	S	S	+	UK	SRR3334080
C00021220	3890-p1	21-Mar-13	6	Y	1	S	S	+	UK	SRR3333661
C00021217	3882-p1	26-Mar-13	6	Y	1	S	S	+	UK	SRR3333571

6e4be9a3-63dd-4b54-9a12-99929e4ca7b6	f6d6f7_79	19-Aug-13	6	Y	1	S	S	+	UK	SRR3317183
C00005170	245-p1	09-Mar-07	7	Y	1	S	S	-	UK	ERS243683
C00005171	464a-p1	05-Jul-07	7	Y	1	S	S	-	UK	ERS216158
C00004132	699-p1	20-Sep-07	7	Y	1	S	S	+	UK	ERS243684
C00004142	899-p1	15-Dec-07	7	Y	1	S	S	-	UK	ERS243685
C00004150	1153-p1	24-Mar-08	7	Y	1	S	S	+	UK	ERS243686
C00004154	1268-p1	05-May-08	7	Y	1	S	S	+	UK	ERS243687
C00004162	1356-p1	21-Jun-08	7	Y	1	S	S	+	UK	ERS346661
C00004164	1380-p1	07-Jul-08	7	Y	1	S	S	+	UK	ERS216159
C00004169	1427-p1	05-Aug-08	7	Y	1	S	S	+	UK	ERS243688
C00000476	Oxf1437a	13-Aug-08	7	Y	1	S	S	+	UK	SRR3340273
C00004174	1473-p1	03-Sep-08	7	Y	1	S	S	+	UK	ERS243689
C00004175	1508-p1	20-Sep-08	7	Y	1	S	S	-	UK	ERS243690
C00004176	1549-p1	10-Oct-08	7	Y	1	S	S	-	UK	ERS243691
C00004179	1602-p1	13-Nov-08	7	Y	1	S	S	-	UK	ERS227420
C00004195	1923-p1	29-May-09	7	Y	1	S	S	+	UK	ERS227414
C00004205	2021-p1	23-Jul-09	7	Y	1	S	S	+	UK	ERS346685
C00004209	2101-p1	27-Aug-09	7	Y	1	S	S	+	UK	SRR3333577
C00004214	2146a-p1	20-Sep-09	7	Y	1	S	S	+	UK	ERS346692
C00004218	2162-p1	06-Oct-09	7	Y	1	S	S	-	UK	ERS243693
C00004319	2237-p1	08-Nov-09	7	Y	1	S	S	+	UK	ERS243694
C00004322	2290-p1	09-Dec-09	7	Y	1	S	S	+	UK	ERS243695
C00004324	2361-p1	19-Jan-10	7	Y	1	S	S	+	UK	ERS346700
C00004332	2397-p1	10-Feb-10	7	Y	1	S	S	+	UK	ERS243696
C00004346	2502-p1	09-Apr-10	7	Y	1	S	S	-	UK	ERS243697
C00004348	2533-p1	22-Apr-10	7	Y	1	S	S	+	UK	ERS346715
C00008308	2752-p1	01-Oct-10	7	Y	1	S	S	+	UK	ERS347334
C00008115	2775-p1	27-Oct-10	7	Y	1	S	S	+	UK	ERS347264
C00008087	2937-p1	27-Apr-11	7	Y	1	S	S	+	UK	ERS243698
C00009872	3157-p1	02-Dec-11	7	Y	1	S	S	+	UK	SRR3333474

C00009882	3164-p1	10-Dec-11	7	Y	1	S	S	+	UK	ERS243699
C00011786	3223-p1	03-Mar-12	7	Y	1	S	S	+	UK	ERS243700
C00009456	3247-p1	14-Apr-12	7	Y	1	S	S	+	UK	SRR3333024
C00013864	3428-p1	10-Jun-12	7	Y	1	S	S	+	UK	SRR3333144
C00020389	3806-p1	29-Jan-13	7	Y	1	S	S	+	UK	SRR3332975
C00021142	12-p1	18-Sep-06	8	Y both	1	S	S	+	UK	SRR3333343
e90d02e9-1fea-4c55-afb7-a38890d00d88	f6d6f7_1	02-Oct-06	8	Y Oxford	1	S	S	+	UK	SRR3317202
C00005909	230-p1	02-Mar-07	8	Y both	1	S	S	+	UK	SRR3333254
C00005910	267-p1	21-Mar-07	8	Y Oxford	1	S	S	+	UK	ERS227411
C00005915	341b-p1	01-May-07	8	Y both	1	S	S	+	UK	SRR3334105
C00005916	349-p1	03-May-07	8	Y Oxford	1	S	S	+	UK	SRR3333594
C00005917	370-p1	14-May-07	8	Y both	1	S	S	+	UK	SRR3333605
C00005920	427-p1	13-Jun-07	8	Y Oxford	1	S	S	+	UK	SRR3334425
C00005921	440-p1	18-Jun-07	8	Y both	1	S	S	+	UK	SRR3334119
C00005922	487-p1	13-Jul-07	8	Y Oxford	1	S	S	+	UK	SRR3339986
C00005923	495-p1	17-Jul-07	8	Y both	1	S	S	+	UK	ERS227355
C00005926	588-p1	16-Aug-07	8	Y Oxford	1	S	S	+	UK	SRR3333555
C00005928	604-p1	28-Aug-07	8	Y both	1	S	S	+	UK	SRR3333735
C00005688	731-p1	08-Oct-07	8	Y Oxford	1	S	S	+	UK	ERS346870
C00005933	732-p1	08-Oct-07	8	Y both	1	S	S	+	UK	ERS347046
C00005934	736-p1	09-Oct-07	8	Y Oxford	1	S	S	+	UK	ERS347047
C00006304	750-p1	16-Oct-07	8	Y Oxford	1	S	S	+	UK	SRR3334132
C00006382	747-p1	16-Oct-07	8	Y both	1	S	S	+	UK	ERS227380
C00006332	779-p2	25-Oct-07	8	Y both	1	R	S	+	UK	ERS347148
C00004823	785-p1	29-Oct-07	8	Y Oxford	1	S	S	+	UK	ERS346771
C00004820	855-p1	27-Nov-07	8	Y both	1	S	S	+	UK	ERS346770
C00004866	900-p1	15-Dec-07	8	Y Oxford	1	S	S	+	UK	ERS346792
C00005692	909-p1	21-Dec-07	8	Y both	1	S	S	+	UK	ERS346874
C00004875	956-p1	09-Jan-08	8	Y Oxford	1	S	S	+	UK	ERS346797
C00006333	977-p2	16-Jan-08	8	Y both	1	S	S	+	UK	ERS347149

C00004886	1002-p1	21-Jan-08	8	Y Oxford	1	S	S	+	UK	ERS346806
C00004828	1024-p1	28-Jan-08	8	Y both	1	S	S	+	UK	ERS346775
C00004879	1111-p1	05-Mar-08	8	Y Oxford	1	S	S	+	UK	ERS346801
C00004827	1142-p1	17-Mar-08	8	Y both	1	S	S	+	UK	ERS346774
C00004802	1144-p1	18-Mar-08	8	Y Oxford	1	S	S	+	UK	ERS346760
C00006334	1148-p2	20-Mar-08	8	Y both	1	S	S	+	UK	ERS227394
C00009024	1192m-p1	14-Apr-08	8	Y Oxford	1	S	S	+	UK	ERS227518
C00004860	1221-p1	23-Apr-08	8	Y both	1	S	S	+	UK	ERS346788
C00004838	1259-p1	02-May-08	8	Y Oxford	1	S	S	+	UK	ERS346780
C00004863	1283-p1	11-May-08	8	Y both	1	S	S	+	UK	ERS346789
C00004809	1295-p1	19-May-08	8	Y both	1	S	S	+	UK	ERS346764
C00006335	1294-p2	19-May-08	8	Y Oxford	1	S	S	+	UK	ERS347150
C00004882	1362-p1	25-Jun-08	8	Y Oxford	1	S	S	+	UK	ERS346803
C00004885	1364-p1	27-Jun-08	8	Y both	1	S	S	+	UK	ERS346805
C00004846	1398-p1	14-Jul-08	8	Y Oxford	1	S	S	+	UK	ERS346782
C00004890	1445-p1	15-Aug-08	8	Y both	1	S	S	+	UK	ERS346809
C00004817	1519a-p1	24-Sep-08	8	Y Oxford	1	S	S	+	UK	ERS346768
C00004889	1514-p1	24-Sep-08	8	Y both	1	S	S	+	UK	ERS346808
C00004837	1520-p1	25-Sep-08	8	Y Oxford	1	S	S	+	UK	ERS346779
C00004870	1529-p1	27-Sep-08	8	Y both	1	S	S	+	UK	ERS346795
C00004855	1548-p1	09-Oct-08	8	Y Oxford	1	S	S	+	UK	ERS346785
C00004865	1600-p1	12-Nov-08	8	Y both	1	S	S	+	UK	ERS346791
C00004812	1622-p1	17-Nov-08	8	Y both	1	S	S	+	UK	ERS227358
C00004832	1623-p1	17-Nov-08	8	Y Oxford	1	S	S	+	UK	ERS346777
C00004867	1659-p1	09-Dec-08	8	Y Oxford	1	S	S	+	UK	ERS346793
C00004858	1666-p1	14-Dec-08	8	Y both	1	S	S	+	UK	SRR3333391
C00004804	1678-p1	17-Dec-08	8	Y Oxford	1	S	S	+	UK	ERS346762
C00000191	Oxf1697	09-Jan-09	8	Y both	1	S	S	+	UK	ERS149843
C00004884	1711-p1	13-Jan-09	8	Y Oxford	1	S	S	+	UK	ERS346804
C00004888	1717-p1	15-Jan-09	8	Y both	1	S	S	+	UK	ERS346807
C00004834	1755-p1	03-Feb-09	8	Y Oxford	1	S	S	+	UK	ERS346778

C00004844	1803-p1	27-Feb-09	8	Y both	1	S	S	+	UK	ERS346781
C00004880	1830-p1	10-Mar-09	8	Y Oxford	1	S	S	+	UK	ERS346802
C00009925	1835-p2	13-Mar-09	8	Y both	1	S	S	+	UK	ERS347376
C00004864	1843b-p1	20-Mar-09	8	Y Oxford	1	S	S	+	UK	ERS346790
C00001462	Oxf1846	30-Mar-09	8	Y both	1	S	S	+	UK	ERS149917
C00004806	1911-p1	19-May-09	8	Y Oxford	1	S	S	+	UK	ERS346763
C00004824	1962-p1	14-Jun-09	8	Y both	1	S	S	+	UK	ERS346772
C00006266	1990-p1	07-Jul-09	8	Y Oxford	1	S	S	+	UK	ERS347104
C00004796	2024-p1	23-Jul-09	8	Y both	1	S	S	+	UK	ERS346758
C00004849	2030-p1	27-Jul-09	8	Y Oxford	1	S	S	+	UK	ERS346783
C00004815	2042-p1	04-Aug-09	8	Y Oxford	1	S	S	+	UK	ERS346767
C00004814	2048-p1	09-Aug-09	8	Y both	1	S	S	+	UK	ERS346766
C00004830	2051-p1	09-Aug-09	8	Y both	1	S	S	+	UK	ERS346776
C00004807	2074-p1	18-Aug-09	8	Y both	1	S	S	+	UK	ERS227419
C00004856	2073-p1	18-Aug-09	8	Y Oxford	1	S	S	+	UK	ERS346786
C00006275	2098-p1	26-Aug-09	8	Y Oxford	1	S	S	+	UK	ERS347109
C00006283	2144-p1	20-Sep-09	8	Y both	1	S	S	+	UK	ERS347115
C00006287	2171-p1	13-Oct-09	8	Y Oxford	1	S	S	+	UK	ERS347118
C00006288	2176a-p1	14-Oct-09	8	Y both	1	S	S	+	UK	ERS347119
C00006289	2180-p1	15-Oct-09	8	Y Oxford	1	S	S	+	UK	ERS347120
C00006291	2195-p1	22-Oct-09	8	Y both	1	S	S	+	UK	ERS347121
C00006292	2199a-p1	24-Oct-09	8	Y Oxford	1	S	S	+	UK	ERS347122
C00006295	2227-p1	05-Nov-09	8	Y both	1	S	S	+	UK	ERS347124
C00006299	2242-p1	11-Nov-09	8	Y Oxford	1	S	S	+	UK	ERS227421
C00006300	2246-p1	12-Nov-09	8	Y both	1	S	S	+	UK	ERS347127
C00006305	2268-p1	01-Dec-09	8	Y Oxford	1	S	S	+	UK	ERS347129
C00006309	2284-p1	07-Dec-09	8	Y both	1	S	S	+	UK	ERS347133
C00005956	2305-p2	19-Dec-09	8	Y Oxford	1	S	S	+	UK	ERS227373
C00006313	2318-p1	26-Dec-09	8	Y both	1	S	S	+	UK	ERS347136
C00006314	2326-p1	31-Dec-09	8	Y Oxford	1	S	S	+	UK	ERS347137
C00006315	2328-p1	01-Jan-10	8	Y both	1	S	S	+	UK	ERS347138

C00006318	2334-p1	04-Jan-10	8	Y Oxford	1	S	S	+	UK	ERS347141
C00006320	2350-p1	14-Jan-10	8	Y both	1	S	S	+	UK	ERS347143
C00006323	2367-p1	22-Jan-10	8	Y Oxford	1	S	S	+	UK	ERS347146
C00006324	2371-p1	26-Jan-10	8	Y both	1	S	S	+	UK	ERS347147
C00005962	2410-p1	17-Feb-10	8	Y Oxford	1	S	S	+	UK	ERS227415
C00005965	2437-p1	01-Mar-10	8	Y both	1	S	S	+	UK	ERS347056
C00005966	2451-p1	11-Mar-10	8	Y Oxford	1	S	S	+	UK	ERS227392
C00005967	2463-p1	17-Mar-10	8	Y both	1	S	S	+	UK	ERS347057
C00005968	2464-p1	18-Mar-10	8	Y Oxford	1	S	S	+	UK	ERS347058
C00005974	2494-p1	03-Apr-10	8	Y both	1	S	S	+	UK	ERS347062
C00008257	2518-p1	14-Apr-10	8	Y Oxford	1	S	S	+	UK	ERS347295
C00008281	2531-p1	21-Apr-10	8	Y both	1	S	S	+	UK	ERS347314
C00005979	2545-p1	02-May-10	8	Y Oxford	1	S	S	+	UK	ERS347066
C00005981	2569-p1	15-May-10	8	Y both	1	S	S	+	UK	ERS347068
C00005983	2616-p1	14-Jun-10	8	Y Oxford	1	S	S	+	UK	ERS347069
C00005988	2652-p1	12-Jul-10	8	Y both	1	S	S	+	UK	ERS347072
C00005989	2674-p1	20-Jul-10	8	Y Oxford	1	S	S	+	UK	ERS347073
C00008318	2690-p1	02-Aug-10	8	Y both	1	S	S	+	UK	ERS347341
C00005993	2695-p1	06-Aug-10	8	Y Oxford	1	S	S	+	UK	ERS227381
C00008077	2715-p1	26-Aug-10	8	Y both	1	S	S	+	UK	ERS347239
C00008066	2729-p1	09-Sep-10	8	Y Oxford	1	S	S	+	UK	ERS347233
C00008126	2736-p1	17-Sep-10	8	Y both	1	S	S	+	UK	ERS347268
C00008150	2738-p1	22-Sep-10	8	Y Oxford	1	S	S	+	UK	ERS347286
C00008332	2754-p1	05-Oct-10	8	Y both	1	S	S	+	UK	ERS347352
C00008297	2760-p1	11-Oct-10	8	Y Oxford	1	S	S	+	UK	ERS347324
C00008068	2783-p1	05-Nov-10	8	Y both	1	S	S	+	UK	ERS347235
C00008298	2791-p1	12-Nov-10	8	Y Oxford	1	S	S	+	UK	ERS347325
C00008105	2801-p1	19-Nov-10	8	Y both	1	S	S	+	UK	ERS347259
C00008323	2814-p1	05-Dec-10	8	Y Oxford	1	S	S	+	UK	ERS347345
C00008276	2839-p1	28-Dec-10	8	Y Oxford	1	S	S	+	UK	ERS347309
C00008277	2851-p1	10-Jan-11	8	Y both	1	S	S	+	UK	ERS347310

C00008278	2860-p1	23-Jan-11	8	Y Oxford	1	R	S	+	UK	ERS347311
C00008095	2866-p1	30-Jan-11	8	Y both	1	S	S	+	UK	ERS347252
C00008085	2885-p1	22-Feb-11	8	Y Oxford	1	S	S	+	UK	ERS347245
C00008098	2895-p1	08-Mar-11	8	Y both	1	S	S	+	UK	ERS347254
C00008315	2905-p1	14-Mar-11	8	Y Oxford	1	S	S	+	UK	ERS347339
C00008061	2920-p1	01-Apr-11	8	Y Oxford	1	S	S	+	UK	SRR3333236
C00008146	2933-p1	19-Apr-11	8	Y both	1	S	S	+	UK	SRR3334192
C00008099	2938-p1	27-Apr-11	8	Y Oxford	1	S	S	+	UK	SRR3334129
C00008135	2942-p1	06-May-11	8	Y both	1	S	S	+	UK	SRR3333444
C00008147	2943-p1	06-May-11	8	Y Oxford	1	S	S	+	UK	ERS347283
C00007807	3005-p1	23-May-11	8	Y both	1	S	S	+	UK	SRR3333332
C00007856	3017-p1	09-Jun-11	8	Y Oxford	1	S	S	+	UK	SRR3333113
C00007834	3031-p1	02-Jul-11	8	Y both	1	S	S	+	UK	SRR3334469
C00007860	3048-p1	27-Jul-11	8	Y Oxford	1	S	S	+	UK	SRR3340213
C00007802	3059-p1	06-Aug-11	8	Y both	1	S	S	+	UK	SRR3334020
C00007827	3069-p1	14-Aug-11	8	Y Oxford	1	S	S	+	UK	SRR3333653
C00008015	3086-p1	10-Sep-11	8	Y both	1	S	S	+	UK	SRR3333789
C00009875	3143-p1	17-Oct-11	8	Y Oxford	1	S	S	+	UK	SRR3333683
C00010653	3131-p1	27-Oct-11	8	Y both	1	S	S	+	UK	SRR3339967
C00010654	3132-p1	29-Oct-11	8	Y Oxford	1	R	S	+	UK	SRR3334202
C00011181	3136-p1	09-Nov-11	8	Y both	1	R	S	+	UK	SRR3334446
decf4033-d6b3-4119-8e7c-8c602623d061	f6d6f7_22	10-Nov-11	8	Y Oxford	1	S	S	+	UK	SRR3317199
C00009840	3169-p1	13-Dec-11	8	Y Oxford	1	R	S	+	UK	SRR3333050
C00009888	3165-p1	13-Dec-11	8	Y both	1	S	S	+	UK	SRR3333244
C00009816	3167-p1	18-Dec-11	8	Y both	1	S	S	+	UK	SRR3340006
C00011208	3199-p1	04-Feb-12	8	Y Oxford	1	S	S	+	UK	SRR3333778
C00014307	3236-p1	27-Mar-12	8	Y both	1	S	S	+	UK	SRR3333083
C00013810	3357-p1	09-Apr-12	8	Y Oxford	1	S	S	+	UK	SRR3339965
C00009455	3246-p1	14-Apr-12	8	Y both	1	S	S	+	UK	SRR3340058
C00009457	3250-p1	21-Apr-12	8	Y Oxford	1	S	S	+	UK	SRR3333297
C00009461	3254-p1	26-Apr-12	8	Y both	1	S	S	+	UK	SRR3333303

C00012724	3335-p1	13-May-12	8	Y Oxford	1	S	S	+	UK	SRR3333655
C00012717	3328-p1	14-May-12	8	Y both	1	S	S	+	UK	SRR3339983
C00013878	3444-p1	30-May-12	8	Y Oxford	1	S	S	+	UK	SRR3333793
C00013901	3476-p1	15-Jun-12	8	Y both	1	S	S	+	UK	SRR3340241
C00015465	3595-p1	10-Sep-12	8	Y Oxford	1	S	S	+	UK	SRR3333862
C00016351	3691-p1	02-Nov-12	8	Y both	1	S	S	+	UK	SRR3333013
C00020102	3724-p1	16-Nov-12	8	Y Oxford	1	S	S	+	UK	SRR3333652
C00020115	3739-p1	27-Nov-12	8	Y both	1	S	S	+	UK	SRR3334213
C00020116	3743-p1	03-Dec-12	8	Y Oxford	1	S	S	+	UK	SRR3340047
C00020123	3750-p1	08-Dec-12	8	Y both	1	S	S	+	UK	SRR3333258
C00020134	3762-p1	27-Dec-12	8	Y Oxford	1	S	S	+	UK	SRR3333270
C00020365	3778-p1	01-Jan-13	8	Y both	1	S	S	+	UK	SRR3333098
C00020368	3781-p1	02-Jan-13	8	Y Oxford	1	S	S	+	UK	SRR3340204
C00020402	3825-p1	25-Feb-13	8	Y both	1	S	S	+	UK	SRR3333618
C00020416	3846-p1	04-Mar-13	8	Y Oxford	1	S	S	+	UK	SRR3333675
C00020422	3854-p1	09-Mar-13	8	Y both	1	S	S	+	UK	SRR3333447
C00021212	3877-p1	22-Mar-13	8	Y Oxford	1	S	S	+	UK	SRR3333989
75b5e956-6256-4742-8547-09365e5169d4	f6d6f7_81	10-Jul-13	8	Y both	1	S	S	+	UK	SRR3317184
C00005996	227-p1	01-Mar-07	9	Y	1	S	S	+	UK	SRR3333595
C00006024	462-p1	03-Jul-07	9	Y	1	S	S	+	UK	SRR3334086
C00006778	516-p1	23-Jul-07	9	Y	1	S	S	+	UK	ERS347202
C00006030	523-p1	25-Jul-07	9	Y	1	S	S	+	UK	SRR3332978
C00004129	653-p1	09-Sep-07	9	Y	1	S	S	+	UK	ERS346641
C00004134	839-p1	21-Nov-07	9	Y	1	S	S	+	UK	ERS346644
C00004146	969-p1	13-Jan-08	9	Y	1	S	S	+	UK	ERS346651
C00004151	1169-p1	02-Apr-08	9	Y	1	S	S	+	UK	ERS346655
C00004159	1319-p1	01-Jun-08	9	Y	1	S	S	+	UK	ERS346658
C00004161	1351-p1	15-Jun-08	9	Y	1	S	S	+	UK	ERS346660
C00004163	1373-p1	30-Jun-08	9	Y	1	S	S	+	UK	ERS346662
C00004172	1431-p1	11-Aug-08	9	Y	1	S	S	+	UK	ERS346665

C00004177	1554a-p1	14-Oct-08	9	Y	1	S	S	+	UK	ERS346666
C00004180	1624-p1	19-Nov-08	9	Y	1	S	S	+	UK	ERS346667
C00004186	1735-p1	26-Jan-09	9	Y	1	S	S	+	UK	ERS346671
C00004193	1820-p1	19-Feb-09	9	Y	1	S	S	+	UK	ERS227402
C00004192	1798b-p1	26-Feb-09	9	Y	1	S	S	+	UK	ERS346676
C00004200	1978a-p2	01-Jul-09	9	Y	1	S	S	+	UK	ERS346682
C00004204	2014-p1	18-Jul-09	9	Y	1	S	S	+	UK	ERS346684
C00004219	2170-p1	13-Oct-09	9	Y	1	S	S	+	UK	ERS346694
C00004356	2606-p1	08-Jun-10	9	Y	1	S	S	+	UK	ERS346722
C00008079	2770-p1	19-Oct-10	9	Y	1	S	S	+	UK	ERS347241
C00007801	3051-p1	09-Jun-11	9	Y	1	S	S	+	UK	ERS347213
C00009876	3163-p1	09-Dec-11	9	Y	1	S	S	+	UK	SRR3333373
C00011780	3216-p1	20-Feb-12	9	Y	1	S	S	+	UK	SRR3339982
C00011792	3229-p1	16-Mar-12	9	Y	1	S	S	+	UK	SRR3340270
C00012709	3319-p1	29-Apr-12	9	Y	1	S	S	+	UK	SRR3333120
C00013868	3433-p1	28-May-12	9	Y	1	S	S	+	UK	SRR3334041
C00013855	3420-p1	31-May-12	9	Y	1	S	S	+	UK	SRR3333064
C00014367	3554-p1	31-Aug-12	9	Y	1	S	S	+	UK	SRR3333071
C00015489	3626-p1	27-Sep-12	9	Y	1	S	S	+	UK	SRR3334189
C00020405	3829-p1	12-Feb-13	9	Y	1	S	S	+	UK	SRR3333559
C00021140	1-p1	13-Sep-06	10	Y	1	S	S	+	UK	SRR3333339
C00021159	104-p1	24-Nov-06	10	Y	1	S	S	+	UK	SRR3332974
C00005995	225-p1	02-Mar-07	10	Y	1	S	S	+	UK	SRR3333805
C00006482	283-p1	10-Apr-07	10	Y	1	S	S	+	UK	SRR3340038
C00006002	318-p1	22-Apr-07	10	Y	1	S	S	+	UK	SRR3334131
C00006015	400-p1	31-May-07	10	Y	1	S	S	+	UK	SRR3334018
C00006016	406-p1	02-Jun-07	10	Y	1	S	S	+	UK	SRR3334127
C00006020	437-p1	17-Jun-07	10	Y	1	S	S	+	UK	SRR3332992
C00000074	Oxf554	04-Aug-07	10	Y	1	S	S	+	UK	ERS149798
C00001641	Oxf660	10-Sep-07	10	Y	1	S	S	+	UK	ERS150026

C00000110	Oxf663b	11-Sep-07	10	Y	1	S	S	+	UK	ERS149817
C00002756	Oxf825-p1	16-Nov-07	10	Y	1	S	S	+	UK	ERS150102
C00002801	Oxf913-p1	22-Dec-07	10	Y	1	S	S	+	UK	SRR3333082
C00002767	Oxf1010-p1	24-Jan-08	10	Y	1	S	S	+	UK	ERS150113
C00009928	1025-p2	28-Jan-08	10	Y	1	S	S	+	UK	ERS347378
C00002770	Oxf1074-p1	21-Feb-08	10	Y	1	S	S	+	UK	ERS150115
C00002772	Oxf1101-p1	02-Mar-08	10	Y	1	S	S	+	UK	ERS150117
C00002776	Oxf1115-p1	06-Mar-08	10	Y	1	S	S	+	UK	ERS150120
C00002784	Oxf1247-p1	26-Apr-08	10	Y	1	S	S	+	UK	ERS150128
C00002785	Oxf1252-p1	30-Apr-08	10	Y	1	S	S	+	UK	ERS150129
C00000565	Oxf1383	09-Jul-08	10	Y	1	S	S	+	UK	ERS346589
C00000215	Oxf1419	30-Jul-08	10	Y	1	S	S	+	UK	ERS149860
C00001548	Oxf1493	14-Sep-08	10	Y	1	S	S	+	UK	ERS149963
C00001547	Oxf1522	26-Sep-08	10	Y	1	S	S	+	UK	SRR3334100
C00001622	Oxf1555	15-Oct-08	10	Y	1	S	S	+	UK	ERS150015
C00001549	Oxf1630	21-Nov-08	10	Y	1	S	S	+	UK	ERS149964
C00001551	Oxf1804c	27-Feb-09	10	Y	1	S	S	+	UK	ERS149965
C00001555	Oxf1985b	05-Jul-09	10	Y	1	S	S	+	UK	ERS149967
C00001556	Oxf2019	22-Jul-09	10	Y	1	S	S	+	UK	ERS149968
C00001557	Oxf2061	13-Aug-09	10	Y	1	S	S	+	UK	ERS149969
C00001560	Oxf2069a	17-Aug-09	10	Y	1	S	S	+	UK	ERS149970
C00001561	Oxf2085	22-Aug-09	10	Y	1	S	S	+	UK	ERS346592
C00001563	Oxf2105a	28-Aug-09	10	Y	1	S	S	+	UK	ERS149972
C00001564	Oxf2134	14-Sep-09	10	Y	1	S	S	+	UK	ERS149973
C00001566	Oxf2203	26-Oct-09	10	Y	1	S	S	+	UK	ERS149975
C00001567	Oxf2204a	27-Oct-09	10	Y	1	S	S	+	UK	ERS149976
C00001569	Oxf2208	27-Oct-09	10	Y	1	S	S	+	UK	ERS149978
C00001572	Oxf2211	29-Oct-09	10	Y	1	S	S	+	UK	ERS346593
C00002738	Oxf2243-p1	11-Nov-09	10	Y	1	S	S	+	UK	ERS150085
C00001575	Oxf2281	07-Dec-09	10	Y	1	S	S	+	UK	ERS149981
C00001576	Oxf2292	13-Dec-09	10	Y	1	S	S	+	UK	ERS149982

C00001577	Oxf2310	23-Dec-09	10	Y	1	S	S	+	UK	ERS149983
C00001578	Oxf2320	29-Dec-09	10	Y	1	S	S	+	UK	ERS149984
C00001579	Oxf2487a	29-Mar-10	10	Y	1	S	S	+	UK	ERS149985
C00002862	Oxf2510-p1	11-Apr-10	10	Y	1	S	S	+	UK	ERS150194
C00002875	Oxf2591-p1	27-May-10	10	Y	1	S	S	+	UK	ERS150204
C00002876	Oxf2592-p1	27-May-10	10	Y	1	S	S	+	UK	ERS150205
C00008258	2634-p1	01-Jul-10	10	Y	1	S	S	+	UK	ERS347296
C00008089	2716-p1	27-Aug-10	10	Y	1	S	S	+	UK	ERS347247
C00008114	2735-p1	19-Sep-10	10	Y	1	S	S	+	UK	ERS347263
C00008343	2746-p1	26-Sep-10	10	Y	1	S	S	+	UK	ERS347362
C00008333	2763-p1	13-Oct-10	10	Y	1	S	S	+	UK	ERS347353
C00008310	2792-p1	13-Nov-10	10	Y	1	S	S	+	UK	ERS347335
C00008322	2805-p1	22-Nov-10	10	Y	1	S	S	+	UK	ERS347344
C00008346	2807-p1	27-Nov-10	10	Y	1	S	S	+	UK	ERS347365
C00008142	2835-p1	21-Dec-10	10	Y	1	S	S	+	UK	ERS347279
C00008014	2927-p1	06-Apr-11	10	Y	1	S	S	+	UK	SRR3333685
C00008122	2931-p1	17-Apr-11	10	Y	1	S	S	+	UK	SRR3333005
C00009002	2946-p1	10-May-11	10	Y	1	S	S	+	UK	SRR3333709
C00007772	3009b-p1	05-Jun-11	10	Y	1	S	S	+	UK	SRR3333690
C00007857	3025a-p1	22-Jun-11	10	Y	1	S	S	+	UK	ERS347227
C00007798	3028-p1	25-Jun-11	10	Y	1	S	S	+	UK	SRR3334033
C00007824	3045-p1	21-Jul-11	10	Y	1	S	S	+	UK	SRR3333664
C00007790	3058-p1	05-Aug-11	10	Y	1	S	S	+	UK	SRR3334214
C00007780	3073-p1	11-Aug-11	10	Y	1	S	S	+	UK	SRR3339997
C00008036	3096-p1	15-Sep-11	10	Y	1	S	S	+	UK	SRR3339964
C00008012	3095-p1	16-Sep-11	10	Y	1	S	S	+	UK	SRR3333276
C00010638	3107-p1	23-Sep-11	10	Y	1	S	S	+	UK	SRR3333554
C00009865	3117-p1	05-Oct-11	10	Y	1	S	S	+	UK	SRR3340253
C00010648	3125-p1	17-Oct-11	10	Y	1	S	S	+	UK	SRR3333638
C00009803	3158-p1	02-Dec-11	10	Y	1	S	S	+	UK	SRR3333441
C00011207	3191-p1	24-Jan-12	10	Y	1	S	S	+	UK	SRR3333092

C00011233	3210-p1	12-Feb-12	10	Y	1	S	S	+	UK	SRR3334188
C00009451	3242-p1	01-Apr-12	10	Y	1	S	S	+	UK	SRR3333230
C00013824	3383-p1	17-Apr-12	10	Y	1	S	S	+	UK	SRR3340196
C00012688	3284-p1	19-Apr-12	10	Y	1	S	S	+	UK	SRR3333467
C00012710	3320-p1	29-Apr-12	10	Y	1	S	S	+	UK	SRR3333445
C00012693	3288-p1	02-May-12	10	Y	1	S	S	+	UK	SRR3334473
C00012728	3342-p1	12-May-12	10	Y	1	S	S	+	UK	SRR3340233
C00013856	3421-p1	06-Jun-12	10	Y	1	S	S	+	UK	SRR3334412
C00014349	3531-p1	18-Jul-12	10	Y	1	S	S	+	UK	SRR3333354
C00014334	3510-p1	25-Jul-12	10	Y	1	S	S	+	UK	SRR3333438
C00014375	3568-p1	04-Sep-12	10	Y	1	S	S	+	UK	SRR3333588
C00015472	3603-p1	09-Sep-12	10	Y	1	S	S	+	UK	SRR3334472
C00012855	3634-p1	27-Sep-12	10	Y	1	S	S	+	UK	SRR3332991
C00020385	3802-p1	23-Jan-13	10	Y	1	S	S	+	UK	SRR3333713
C00020425	3857-p1	03-Mar-13	10	Y	1	S	S	+	UK	SRR3340069
C00021213	3878-p1	21-Mar-13	10	Y	1	S	S	+	UK	SRR3340191
C00021218	3885-p1	27-Mar-13	10	Y	1	S	S	+	UK	SRR3332999
C00001512	Oxf14	20-Sep-06	11	Y	5	S	S	+	UK	SRR3334159
C00012860	16-p1	20-Sep-06	11	Y	5	S	S	+	UK	SRR3333625
C00012864	116-p1	10-Dec-06	11	Y	5	S	S	+	UK	SRR3333770
C00001494	Oxf328a	25-Apr-07	11	Y	5	S	S	+	UK	ERS149938
C00006009	369-p1	12-May-07	11	Y	5	S	S	+	UK	SRR3340010
C00006007	365-p1	13-May-07	11	Y	5	S	S	+	UK	SRR3333090
C00006014	398-p1	30-May-07	11	Y	5	S	S	+	UK	SRR3333796
C00005783	575-p2	11-Aug-07	11	Y	5	S	S	+	UK	SRR3333710
C00006471	584-p1	19-Aug-07	11	Y	5	S	S	+	UK	SRR3333275
C00002748	Oxf774a-p1	24-Oct-07	11	Y	5	S	S	+	UK	ERS150095
C00021193	773-p3	25-Oct-07	11	Y	5	S	S	+	UK	SRR3334195
C00008256	789a-p1	31-Oct-07	11	Y	5	S	S	+	UK	ERS347294
C00002759	Oxf903-p1	18-Dec-07	11	Y	5	S	S	+	UK	ERS150105

C00002763	Oxf947-p1	04-Jan-08	11	Y	5	S	S	+	UK	ERS150109
C00001514	Oxf1193	14-Apr-08	11	Y	5	S	S	+	UK	ERS149949
C00002782	Oxf1237-p1	28-Apr-08	11	Y	5	S	S	+	UK	ERS150126
C00009926	1369-p2	29-Jun-08	11	Y	5	S	S	+	UK	ERS347377
C00003947	Oxf1428-p1	07-Aug-08	11	Y	5	S	S	+	UK	ERS346597
C00002794	Oxf1434-p1	13-Aug-08	11	Y	5	S	S	+	UK	ERS150137
C00001516	Oxf1542	08-Oct-08	11	Y	5	S	S	+	UK	ERS149950
C00002809	Oxf1557-p1	15-Oct-08	11	Y	5	S	S	+	UK	ERS150146
C00002732	Oxf1841a-p1	17-Mar-09	11	Y	5	R	S	+	UK	ERS150081
C00001517	Oxf2071a	16-Aug-09	11	Y	5	S	S	+	UK	ERS149951
C00002827	Oxf2111-p1	02-Sep-09	11	Y	5	S	S	+	UK	ERS150164
C00009939	2225-p1	05-Nov-09	11	Y	5	S	S	+	UK	SRR3334208
C00002838	Oxf2253-p1	17-Nov-09	11	Y	5	S	S	+	UK	ERS150174
C00001519	Oxf2332	03-Jan-10	11	Y	5	S	S	+	UK	ERS149953
C00002849	Oxf2374-p1	27-Jan-10	11	Y	5	S	S	+	UK	ERS150183
C00002850	Oxf2375-p1	28-Jan-10	11	Y	5	S	S	+	UK	ERS150184
C00001520	Oxf2377	31-Jan-10	11	Y	5	S	S	+	UK	ERS149954
C00003942	Oxf2439-p1	01-Mar-10	11	Y	5	S	S	+	UK	ERS346595
C00001521	Oxf2443	03-Mar-10	11	Y	5	S	S	+	UK	ERS149955
C00002859	Oxf2497-p1	06-Apr-10	11	Y	5	S	S	+	UK	ERS150191
C00002863	Oxf2516a-p1	14-Apr-10	11	Y	5	S	S	+	UK	ERS150195
C00008269	2520-p1	15-Apr-10	11	Y	5	S	S	+	UK	ERS347306
C00002864	Oxf2521a-p1	17-Apr-10	11	Y	5	S	S	+	UK	ERS150196
C00009904	2557-p1	09-May-10	11	Y	5	R	S	+	UK	SRR3340206
C00002874	Oxf2588-p1	26-May-10	11	Y	5	S	S	+	UK	ERS150203
C00003949	Oxf2677-p1	23-Jul-10	11	Y	5	S	S	+	UK	ERS346599
C00008148	2709-p1	15-Aug-10	11	Y	5	S	S	+	UK	ERS347284
C00007256	2720-p0	28-Aug-10	11	Y	5	S	S	+	UK	ERS140992
C00008113	2722-p1	31-Aug-10	11	Y	5	S	S	+	UK	ERS347262
C00008296	2751-p1	02-Oct-10	11	Y	5	S	S	+	UK	ERS347323
C00008274	2768-p1	18-Oct-10	11	Y	5	S	R	+	UK	ERS347308

C00008151	2779-p1	29-Oct-10	11	Y	5	S	S	+	UK	ERS347287
C00008302	2863-p1	28-Jan-11	11	Y	5	S	S	+	UK	ERS347329
C00008072	2874-p1	04-Feb-11	11	Y	5	S	S	+	UK	ERS347237
C00008084	2875-p1	07-Feb-11	11	Y	5	S	S	+	UK	ERS347244
C00008096	2877-p1	08-Feb-11	11	Y	5	S	S	+	UK	ERS347253
C00008350	2898-p1	09-Mar-11	11	Y	5	S	S	+	UK	ERS347367
C00008291	2903-p1	13-Mar-11	11	Y	5	S	S	+	UK	ERS347321
C00007977	2913-p1	28-Mar-11	11	Y	5	R	S	+	UK	ERS347229
C00008049	2919-p1	01-Apr-11	11	Y	5	S	S	+	UK	SRR3333755
C00008002	2925-p1	05-Apr-11	11	Y	5	S	S	+	UK	SRR3334203
C00009014	2947-p1	10-May-11	11	Y	5	R	S	+	UK	SRR3333263
9edb10c7-ffc7-42d3-8138-d45b37aad5cc	f6d6f7_17	30-May-11	11	Y	5	S	S	+	UK	SRR3317187
C00007843	3008-p1	05-Jun-11	11	Y	5	S	S	+	UK	SRR3332980
C00007808	3012-p1	08-Jun-11	11	Y	5	S	S	+	UK	SRR3340068
C00010637	3016-p1	09-Jun-11	11	Y	5	S	S	+	UK	SRR3333350
C00009871	3120-p1	11-Oct-11	11	Y	5	R	S	+	UK	SRR3334009
C00009887	3146-p1	15-Oct-11	11	Y	5	S	S	+	UK	SRR3332944
C00011194	3181-p1	06-Jan-12	11	Y	5	S	S	+	UK	SRR3333654
C00011195	3190-p1	24-Jan-12	11	Y	5	R	S	+	UK	SRR3333047
C00011244	3202-p1	06-Feb-12	11	Y	5	S	S	+	UK	SRR3333784
C00011779	3215-p1	20-Feb-12	11	Y	5	S	S	+	UK	SRR3339954
C00011787	3224-p1	07-Mar-12	11	Y	5	S	S	+	UK	SRR3333041
C00014308	3237-p1	26-Mar-12	11	Y	5	S	S	+	UK	SRR3334054
C00012702	3308-p1	31-Mar-12	11	Y	5	S	S	+	UK	SRR3337754
C00009458	3251-p1	23-Apr-12	11	Y	5	S	S	+	UK	SRR3337758
C00013892	3465-p1	29-Jun-12	11	Y	5	S	S	+	UK	SRR3335295
C00014340	3518-p1	16-Jul-12	11	Y	5	S	S	+	UK	SRR3339369
C00014356	3542-p1	11-Aug-12	11	Y	5	R	S	+	UK	SRR3335302
C00013412	3640-p1	01-Oct-12	11	Y	5	S	S	+	UK	SRR3338967
C00015511	3661-p1	16-Oct-12	11	Y	5	S	S	+	UK	SRR3338225
C00016347	3687-p1	30-Oct-12	11	Y	5	S	S	+	UK	SRR3339393

C00016352	3694-p1	04-Nov-12	11	Y	5	S	S	+	UK	SRR3338919
C00020101	3723-p1	19-Nov-12	11	Y	5	S	R	+	UK	SRR3335196
C00020105	3727-p1	23-Nov-12	11	Y	5	S	S	+	UK	SRR3338529
C00002724	Oxf1320a-p1	02-Jun-08	12	N	1	S	S	+	UK	ERS150074
C00000478	Oxf1424	30-Jul-08	12	N	1	S	S	+	UK	SRR3335921
C00002725	Oxf1470-p1	01-Sep-08	12	N	1	S	S	+	UK	ERS150075
C00000491	Oxf1478	05-Sep-08	12	N	1	S	S	+	UK	ERS149873
C00000493	Oxf1488	12-Sep-08	12	N	1	S	S	+	UK	ERS149875
C00000558	Oxf1526	26-Sep-08	12	N	1	S	S	+	UK	ERS346588
C00000490	Oxf1609	16-Nov-08	12	N	1	S	S	+	UK	ERS149872
C00000492	Oxf2186	19-Oct-09	12	N	1	S	S	+	UK	ERS149874
C00003951	Oxf2693-p1	06-Aug-10	12	N	1	S	S	+	UK	ERS346601
C00011242	3186-p1	13-Jan-12	12	N	1	S	S	+	UK	SRR3338562
C00011220	3200-p1	06-Feb-12	12	N	1	S	S	+	UK	SRR3336067
C00013814	3365-p1	23-Apr-12	12	N	1	S	S	+	UK	SRR3335832
C00021188	208-p1	20-Feb-07	13	Y	1	S	S	+	UK	SRR3337521
C00004143	902-p1	18-Dec-07	13	Y	1	S	S	+	UK	ERS346649
C00004155	1279-p1	09-May-08	13	Y	1	S	S	+	UK	ERS227401
C00000149	Oxf1533	30-Sep-08	13	Y	1	S	S	+	UK	ERS139376
C00004191	1791-p1	25-Feb-09	13	Y	1	S	S	+	UK	ERS346675
C00004203	2008-p1	16-Jul-09	13	Y	1	S	S	+	UK	ERS346683
C00004207	2053-p1	10-Aug-09	13	Y	1	S	S	+	UK	ERS346687
C00000511	Oxf2080	20-Aug-09	13	Y	1	S	S	+	UK	ERS149886
C00004211	2114-p1	03-Sep-09	13	Y	1	S	S	+	UK	ERS346689
C00004215	2151a-p1	25-Sep-09	13	Y	1	S	S	+	UK	ERS346693
C00004217	2159-p1	04-Oct-09	13	Y	1	S	S	+	UK	ERS227369
C00004222	2221-p1	04-Nov-09	13	Y	1	S	S	+	UK	ERS346696
C00004330	2388-p1	07-Feb-10	13	Y	1	S	S	+	UK	ERS346704
C00004339	2412-p1	19-Feb-10	13	Y	1	S	S	+	UK	ERS346708
C00004354	2586-p1	24-May-10	13	Y	1	S	S	+	UK	ERS346720

C00004355	2604-p1	04-Jun-10	13	Y	1	S	S	+	UK	ERS346721
C00004358	2614-p1	13-Jun-10	13	Y	1	S	S	+	UK	ERS346724
C00004359	2628-p1	24-Jun-10	13	Y	1	S	S	+	UK	ERS346725
C00007252	2686-p0	29-Jul-10	13	Y	1	S	S	+	UK	SRR3336056
C00021200	2725-p2	05-Sep-10	13	Y	1	S	S	+	UK	SRR3335973
C00008093	2800-p1	19-Nov-10	13	Y	1	S	S	+	UK	ERS347250
C00008300	2842-p1	29-Dec-10	13	Y	1	S	S	+	UK	ERS347327
C00008348	2847-p1	07-Jan-11	13	Y	1	S	S	+	UK	ERS347366
C00008327	2907-p1	14-Mar-11	13	Y	1	S	S	+	UK	ERS347348
C00008351	2909-p1	17-Mar-11	13	Y	1	S	S	+	UK	ERS347368
C00007989	2914-p1	29-Mar-11	13	Y	1	S	S	+	UK	ERS347230
C00008990	2945-p1	09-May-11	13	Y	1	S	S	+	UK	SRR3334850
C00010656	3134-p1	04-Nov-11	13	Y	1	S	S	+	UK	SRR3335186
C00020412	3841-p1	03-Feb-13	13	Y	1	S	S	+	UK	SRR3338629
C00020426	3858-p1	07-Mar-13	13	Y	1	S	S	+	UK	SRR3335555
C00021141	6a-p1	15-Sep-06	14	Y	1	S	S	+	UK	SRR3337784
C00021151	40-p1	02-Oct-06	14	Y	1	S	S	+	UK	SRR3335953
C00006766	261-p1	18-Mar-07	14	Y	1	S	S	+	UK	SRR3337518
C00006026	486-p1	13-Jul-07	14	Y	1	S	S	+	UK	SRR3334935
C00004130	657-p1	09-Sep-07	14	Y	1	S	S	+	UK	ERS346642
C00004137	845-p1	25-Nov-07	14	Y	1	S	S	+	UK	ERS346646
C00004147	986-p1	19-Jan-08	14	Y	1	S	S	+	UK	ERS346652
C00001471	Oxf1055	11-Feb-08	14	Y	1	S	S	+	UK	ERS149922
C00000223	Oxf1161	28-Mar-08	14	Y	1	S	S	+	UK	ERS149867
C00004152	1185-p1	09-Apr-08	14	Y	1	S	S	+	UK	ERS346656
C00004156	1287-p1	16-May-08	14	Y	1	S	S	+	UK	ERS227356
C00004167	1406-p1	21-Jul-08	14	Y	1	S	S	+	UK	ERS346663
C00004168	1423-p1	30-Jul-08	14	Y	1	S	S	+	UK	ERS346664
C00000158	Oxf1438	07-Aug-08	14	Y	1	S	S	+	UK	ERS149836
C00004382	1441-p1	15-Aug-08	14	Y	1	S	S	+	UK	ERS346737

C00000479	Oxf1449	19-Aug-08	14	Y	1	S	S	+	UK	SRR3336089
C00000160	Oxf1463	26-Aug-08	14	Y	1	S	S	+	UK	ERS139377
C00004181	1636-p1	27-Nov-08	14	Y	1	S	S	+	UK	ERS346668
C00004184	1715-p1	13-Jan-09	14	Y	1	S	S	+	UK	ERS346669
C00002731	Oxf1754-p1	03-Feb-09	14	Y	1	S	S	+	UK	ERS150080
C00004187	1752-p1	03-Feb-09	14	Y	1	S	S	+	UK	ERS346672
C00004194	1870-p1	16-Apr-09	14	Y	1	S	S	+	UK	ERS346677
C00004197	1951-p1	08-Jun-09	14	Y	1	S	S	+	UK	ERS346679
C00004006	Oxf1958-p1	12-Jun-09	14	Y	1	S	S	+	UK	SRR3338670
C00004208	2093-p1	24-Aug-09	14	Y	1	S	S	+	UK	ERS227390
C00004212	2132-p1	14-Sep-09	14	Y	1	S	S	+	UK	ERS346690
C00004220	2179-p1	14-Oct-09	14	Y	1	S	S	+	UK	ERS227405
C00004347	2505-p1	11-Apr-10	14	Y	1	S	S	+	UK	ERS346714
C00004351	2558-p1	10-May-10	14	Y	1	S	S	+	UK	ERS346717
C00004360	2629-p1	21-Jun-10	14	Y	1	S	S	+	UK	ERS346726
C00004363	2664-p1	15-Jul-10	14	Y	1	S	S	+	UK	ERS346728
C00004364	2681-p1	26-Jul-10	14	Y	1	S	S	+	UK	ERS346729
C00008067	2769-p1	19-Oct-10	14	Y	1	S	S	+	UK	ERS347234
C00008335	2817-p1	07-Dec-10	14	Y	1	S	S	+	UK	ERS347355
C00008130	2834-p1	17-Dec-10	14	Y	1	S	S	+	UK	ERS347272
C00008336	2846-p1	05-Jan-11	14	Y	1	S	S	+	UK	ERS347356
C00008337	2857-p1	17-Jan-11	14	Y	1	S	S	+	UK	ERS347357
C00008145	2891-p1	25-Feb-11	14	Y	1	S	S	+	UK	ERS347282
C00007990	2924-p1	05-Apr-11	14	Y	1	S	S	+	UK	SRR3338264
C00007783	3003-p1	26-May-11	14	Y	1	S	S	+	UK	SRR3335442
C00007813	3052-p1	30-Jul-11	14	Y	1	S	S	+	UK	SRR3335528
C00011264	3135-p1	07-Nov-11	14	Y	1	S	S	+	UK	SRR3335273
C00009873	3173-p1	26-Dec-11	14	Y	1	S	S	+	UK	SRR3335997
C00011230	3185-p1	14-Jan-12	14	Y	1	S	S	+	UK	SRR3335468
C00011255	3195-p1	30-Jan-12	14	Y	1	S	S	+	UK	SRR3337581
C00011268	3204-p1	08-Feb-12	14	Y	1	S	S	+	UK	SRR3338776

C00011209	3208-p1	13-Feb-12	14	Y	1	S	S	+	UK	SRR3338535
C00014301	3217-p1	26-Feb-12	14	Y	1	S	S	+	UK	SRR3335220
C00015497	3638-p1	30-Sep-12	14	Y	1	S	S	+	UK	SRR3337675
C00020117	3744-p1	01-Dec-12	14	Y	1	S	S	+	UK	SRR3339279
C00020372	3787-p1	07-Jan-13	14	Y	1	S	R	+	UK	SRR3338176
C00020376	3793-p1	16-Jan-13	14	Y	1	S	S	+	UK	SRR3334836
C00020381	3798-p1	16-Jan-13	14	Y	1	S	S	+	UK	SRR3338208
C00020400	3823-p1	11-Feb-13	14	Y	1	S	R	+	UK	SRR3337706
C00020423	3855-p1	25-Feb-13	14	Y	1	S	R	+	UK	SRR3335435
C00005172	1342-p1	09-Jun-08	15	N	1	S	S	-	UK	ERS139412
C00005174	1631-p1	24-Nov-08	15	N	1	S	S	-	UK	ERS346846
C00010617	1761-p2	07-Feb-09	15	N	1	S	S	-	UK	ERS347386
C00006349	1796-p1	26-Feb-09	15	N	1	S	S	-	UK	ERS347152
C00006350	1799-p1	26-Feb-09	15	N	1	S	S	-	UK	ERS347153
C00006351	1851-p1	01-Apr-09	15	N	1	S	S	-	UK	ERS347154
C00021150	35-p1	29-Sep-06	16	Y	1	S	S	+	UK	SRR3338570
C00021153	66-p1	24-Oct-06	16	Y	1	S	S	+	UK	SRR3335498
C00006483	590-p1	17-Aug-07	16	Y	1	S	S	+	UK	ERS139421
C00004133	742-p1	15-Oct-07	16	Y	1	S	S	+	UK	SRR3337712
C00004138	849-p1	26-Nov-07	16	Y	1	S	S	+	UK	ERS346647
C00007793	1505-p2	19-Sep-08	16	Y	1	S	S	+	UK	ERS347210
C00000512	Oxf1580	29-Oct-08	16	Y	1	S	S	+	UK	SRR3335288
C00009929	1654-p2	06-Dec-08	16	Y	1	S	S	+	UK	SRR3338510
C00004185	1733-p1	24-Jan-09	16	Y	1	S	S	+	UK	ERS346670
C00000194	Oxf1790	24-Feb-09	16	Y	1	S	S	+	UK	ERS149844
C00004198	1971-p1	26-Jun-09	16	Y	1	S	S	+	UK	ERS346680
C00009953	1989a-p2	07-Jul-09	16	Y	1	S	S	+	UK	ERS347381
C00004206	2037-p1	31-Jul-09	16	Y	1	S	S	+	UK	ERS346686
C00004323	2321-p1	28-Dec-09	16	Y	1	S	S	+	UK	ERS346699
C00009965	2393-p2	10-Feb-10	16	Y	1	S	S	+	UK	SRR3335300

C00004334	2404-p1	13-Feb-10	16	Y	1	S	S	+	UK	ERS346706
C00008342	2699-p1	11-Aug-10	16	Y	1	S	S	+	UK	ERS347361
C00008138	2737-p1	20-Sep-10	16	Y	1	S	S	+	UK	ERS347275
C00008325	2856-p1	16-Jan-11	16	Y	1	S	S	+	UK	ERS347346
C00008131	2869-p1	04-Feb-11	16	Y	1	S	S	+	UK	ERS347273
C00008038	2929-p1	12-Apr-11	16	Y	1	S	S	+	UK	SRR3338771
C00007820	3013-p1	07-Jun-11	16	Y	1	S	S	+	UK	SRR3335457
C00009811	3101-p1	05-Sep-11	16	Y	1	S	S	+	UK	SRR3338277
C00008039	3088-p1	09-Sep-11	16	Y	1	S	S	+	UK	SRR3334879
C00009838	3150-p1	13-Oct-11	16	Y	1	S	S	+	UK	SRR3338158
C00009804	3166-p1	14-Dec-11	16	Y	1	S	S	+	UK	SRR3336088
C00013895	3469-p1	04-Jul-12	16	Y	1	S	S	+	UK	SRR3334934
C00014352	3535-p1	06-Aug-12	16	Y	1	S	S	+	UK	SRR3334874
C00014353	3537-p1	09-Aug-12	16	Y	1	S	S	+	UK	SRR3335833
C00020107	3729-p1	22-Nov-12	16	Y	1	S	S	+	UK	SRR3338469
C00000473	Oxf79	01-Nov-06	17	Y	1	S	S	+	UK	SRR3338276
C00002721	Oxf572-p1	12-Aug-07	17	Y	1	S	S	+	UK	ERS150071
C00000107	Oxf663a-1	11-Sep-07	17	Y	1	S	S	+	UK	SRR3338552
C00001635	Oxf665	12-Sep-07	17	Y	1	S	S	+	UK	ERS150021
C00009949	668b-p2	12-Sep-07	17	Y	1	S	S	+	UK	ERS347380
C00000503	Oxf728	08-Oct-07	17	Y	1	R	S	+	UK	ERS149880
C00000218	Oxf868	03-Dec-07	17	Y	1	S	S	+	UK	ERS149863
C00000500	Oxf1155	25-Mar-08	17	Y	1	S	S	+	UK	ERS346584
C00000102	Oxf1184	10-Apr-08	17	Y	1	S	S	+	UK	ERS149815
C00000143	Oxf1192e	14-Apr-08	17	Y	1	S	S	+	UK	ERS139375
C00000501	Oxf1262	02-May-08	17	Y	1	S	S	+	UK	ERS346585
C00000502	Oxf1388	10-Jul-08	17	Y	1	S	S	+	UK	ERS149879
C00000123	Oxf1591-2	09-Nov-08	17	Y	1	S	S	+	UK	SRR3337610
C00000113	Oxf1613	17-Nov-08	17	Y	1	S	S	+	UK	SRR3338537
C00000504	Oxf1896	05-May-09	17	Y	1	S	S	+	UK	ERS149881
C00006364	1938-p1	04-Jun-09	17	Y	1	S	S	+	UK	ERS347159

C00000498	Oxf1963	16-Jun-09	17	Y	1	S	S	+	UK	ERS346583
C00002818	Oxf1982-p1	02-Jul-09	17	Y	1	S	S	+	UK	ERS150155
C00002820	Oxf2013a-p1	16-Jul-09	17	Y	1	S	S	+	UK	ERS150157
C00002881	Oxf2617-p1	14-Jun-10	17	Y	1	S	S	+	UK	ERS150208
C00008966	2739-p2	23-Sep-10	17	Y	1	S	S	+	UK	ERS347369
C00008260	2747-p1	27-Sep-10	17	Y	1	S	S	+	UK	ERS347298
C00008127	2777-p1	24-Oct-10	17	Y	1	S	S	+	UK	ERS347269
C00007859	3041-p1	18-Jul-11	17	Y	1	S	S	+	UK	SRR3338718
C00009860	3153-p1	22-Nov-11	17	Y	1	S	S	+	UK	SRR3335559
C00013894	3467-p1	14-Jun-12	17	Y	1	S	S	+	UK	SRR3338740
C00015499	3642-p1	01-Oct-12	17	Y	1	S	S	+	UK	SRR3337597
C00020127	3754-p1	03-Dec-12	17	Y	1	R	S	+	UK	SRR3338095
C00020133	3761-p1	04-Jan-13	17	Y	1	S	S	+	UK	SRR3335157
C00021149	34-p1	29-Sep-06	18	N	1	S	S	+	UK	SRR3335719
C00005667	193-p1	12-Feb-07	18	N	1	S	S	+	UK	ERS346856
C00005912	292-p1	11-Apr-07	18	N	1	S	S	+	UK	SRR3338914
C00005919	407-p1	04-Jun-07	18	N	1	S	S	+	UK	SRR3334927
C00005925	532b-p1	29-Jul-07	18	N	1	S	S	+	UK	SRR3338925
C00005927	596-p1	22-Aug-07	18	N	1	S	S	+	UK	SRR3337605
C00005929	618-p1	30-Aug-07	18	N	1	S	S	+	UK	SRR3338960
C00006387	811a-p1	11-Nov-07	18	N	1	S	S	+	UK	ERS347169
C00006262	1983-p1	03-Jul-09	18	N	1	S	S	+	UK	ERS347101
C00006298	2239-p1	10-Nov-09	18	N	1	S	S	+	UK	ERS347126
C00008978	2740-p2	23-Sep-10	18	N	1	S	S	+	UK	ERS347370
C00008141	2824-p1	10-Dec-10	18	N	1	S	S	+	UK	ERS347278
C00008267	2900-p1	11-Mar-11	18	N	1	S	S	+	UK	SRR3334937
C00013898	3472-p1	18-Jun-12	18	N	1	S	S	+	UK	SRR3335853
C00006410	1412-p1	25-Jul-08	19	N	1	S	S	+	UK	ERS347187
C00005932	676-p1	16-Sep-07	21	N	1	S	S	+	UK	ERS347045
C00006446	561-p1	06-Aug-07	22	N	3	S	S	+	UK	SRR3334939

C00001508	Oxf1170	02-Apr-08	22	N	3	S	S	+	UK	ERS149946
C00001509	Oxf1430	09-Aug-08	22	N	3	S	S	+	UK	ERS149947
C00001510	Oxf1945	07-Jun-09	22	N	3	S	S	+	UK	ERS149948
C00011257	3212-p1	17-Feb-12	22	N	3	S	S	+	UK	SRR3338200
C00013869	3434-p1	29-May-12	22	N	3	S	S	+	UK	SRR3338977
C00014335	3513-p1	26-Jul-12	22	N	3	S	S	+	UK	SRR3334900
C00006282	904-p1	18-Dec-07	23	N	4	S	S	-	UK	ERS139416
C00006394	1205-p1	17-Apr-08	24	N	1	S	S	+	UK	ERS347172
C00002723	Oxf1232-p1	26-Apr-08	25	N	3	S	S	+	UK	ERS150073
C00006352	2157-p1	01-Oct-09	26	N	1	S	S	-	UK	ERS227364
C00008316	2433-p1	28-Feb-10	26	N	1	R	S	-	UK	ERS347340
C00005975	2514-p1	12-Apr-10	26	N	1	S	S	-	UK	SRR3335578
C00008070	2826-p1	11-Dec-10	26	N	1	S	S	-	UK	ERS347236
C00008133	2890-p1	22-Feb-11	26	N	1	S	S	-	UK	ERS347274
C00011793	3230-p1	19-Mar-12	29	N	1	S	S	-	UK	SRR3334918
C00012694	3289-p1	02-May-12	29	N	1	S	S	-	UK	SRR3337680
C00006247	1839b-p1	16-Mar-09	31	N	1	S	S	+	UK	SRR3338519
C00006028	506-p1	20-Jul-07	33	N	1	S	S	+	UK	SRR3338218
C00004136	840-p1	21-Nov-07	33	N	1	S	S	+	UK	ERS346645
C00004140	888-p1	11-Dec-07	33	N	1	S	S	+	UK	ERS346648
C00004144	931-p1	30-Dec-07	33	N	1	S	S	+	UK	ERS346650
C00004148	1004-p1	22-Jan-08	33	N	1	S	S	+	UK	ERS346653
C00004149	1147-p1	20-Mar-08	33	N	1	S	S	+	UK	ERS346654
C00004183	1710-p1	14-Jan-09	33	N	1	S	S	+	UK	ERS227407
C00004196	1932-p1	01-Jun-09	33	N	1	S	S	+	UK	ERS346678
C00004210	2102-p1	28-Aug-09	33	N	1	S	S	+	UK	ERS346688
C00004213	2138-p1	17-Sep-09	33	N	1	S	S	+	UK	ERS346691
C00004357	2610-p1	09-Jun-10	33	N	1	S	S	+	UK	ERS346723
C00007786	3027-p1	24-Jun-11	33	N	1	S	S	+	UK	SRR3338239
C00007812	3044-p1	21-Jul-11	33	N	1	S	S	+	UK	SRR3335156

C00007979	3083-p1	31-Aug-11	33	N	1	S	S	+	UK	SRR3335816
C00015478	3607-p1	18-Sep-12	33	N	1	S	S	+	UK	SRR3335919
C00020124	3751-p1	08-Dec-12	33	N	1	S	S	+	UK	SRR3334944
C00010628	1177-p2	06-Apr-08	34	N	1	S	S	+	UK	ERS347395
C00008149	2728-p1	08-Sep-10	34	N	1	R	S	+	UK	ERS347285
C00011266	3188-p1	13-Jan-12	34	N	1	S	S	+	UK	SRR3335948
C00020393	3812-p1	01-Feb-13	34	N	1	S	S	+	UK	SRR3337676
C00006459	557-p1	05-Aug-07	35	N	1	S	S	+	UK	SRR3335229
C00002752	Oxf794-p1	05-Nov-07	35	N	1	S	S	+	UK	ERS150099
C00000182	Oxf1121	08-Mar-08	35	N	1	R	S	+	UK	ERS139379
C00002778	Oxf1133-p1	13-Mar-08	35	N	1	R	S	+	UK	ERS150122
C00008965	1888m-p1	28-Apr-09	35	N	1	S	S	+	UK	ERS227500
C00001586	Oxf1956	13-Jun-09	35	N	1	S	S	+	UK	ERS149987
C00001582	Oxf2092	24-Aug-09	35	N	1	S	S	+	UK	SRR3337776
C00001589	Oxf2303	19-Dec-09	35	N	1	S	S	+	UK	ERS149990
C00001593	Oxf2337a	05-Jan-10	35	N	1	S	S	+	UK	ERS149992
C00009893	2566-p2	12-May-10	35	N	1	S	S	+	UK	ERS347371
C00008331	2745-p1	27-Sep-10	35	N	1	S	S	+	UK	ERS347351
C00008287	2810-p1	30-Nov-10	35	N	1	S	S	+	UK	ERS347318
C00007773	3018-p1	14-Jun-11	35	N	1	S	S	+	UK	SRR3338505
C00007822	3030-p1	02-Jul-11	35	N	1	S	S	+	UK	ERS347218
C00009837	3140-p1	18-Nov-11	35	N	1	S	S	+	UK	SRR3338785
C00014370	3559-p1	23-Aug-12	35	N	1	R	S	+	UK	SRR3334802
C00028631	3725-p1	23-Nov-12	35	N	1	S	S	+	UK	SRR3338768
C00021161	110-p1	01-Dec-06	36	Y	1	S	S	+	UK	SRR3335290
C00021164	118b-p1	15-Dec-06	36	Y	1	S	S	+	UK	SRR3338116
C00021166	131-p1	07-Jan-07	36	Y	1	S	S	+	UK	SRR3339384
C00021189	215-p2	28-Feb-07	36	Y	1	S	S	+	UK	SRR3335117
C00004153	1218-p1	21-Apr-08	36	Y	1	S	S	+	UK	SRR3338783
C00004158	1317a-p1	31-May-08	36	Y	1	S	S	+	UK	ERS346657

C00004160	1326-p1	05-Jun-08	36	Y	1	S	S	+	UK	ERS346659
C00007781	1381-p2	08-Jul-08	36	Y	1	S	S	+	UK	ERS347209
C00004004	Oxf1916-p1	21-May-09	36	Y	1	S	S	+	UK	ERS346627
C00004320	2247-p1	12-Nov-09	36	Y	1	S	S	+	UK	ERS346697
C00004321	2289-p1	10-Dec-09	36	Y	1	S	S	+	UK	ERS346698
C00004338	2411-p1	17-Feb-10	36	Y	1	S	S	+	UK	ERS346707
C00009963	2425-p1	24-Feb-10	36	Y	1	S	S	+	UK	SRR3335570
C00004352	2559-p1	10-May-10	36	Y	1	S	S	+	UK	ERS346718
C00004353	2574-p1	17-May-10	36	Y	1	S	S	+	UK	ERS346719
C00004365	2682-p1	23-Jul-10	36	Y	1	S	S	+	UK	ERS346730
C00008104	2788-p1	09-Nov-10	36	Y	1	S	S	+	UK	ERS347258
C00007816	3076-p1	17-Aug-11	36	Y	1	S	S	+	UK	SRR3337780
C00008050	3081-p1	01-Sep-11	36	Y	1	S	S	+	UK	SRR3334883
C00012678	3272-p1	25-May-12	36	Y	1	S	S	+	UK	SRR3337729
C00000549	Oxf9a	17-Sep-06	37	Y	4	R	S	+	UK	SRR3338244
C00000553	Oxf60	19-Oct-06	37	Y	4	R	S	+	UK	ERS149907
C00000550	Oxf63	21-Oct-06	37	Y	4	R	S	+	UK	ERS149905
C00000548	Oxf69	26-Oct-06	37	Y	4	R	S	+	UK	SRR3338237
C00000552	Oxf391	30-May-07	37	Y	4	R	S	+	UK	ERS149906
C00000556	Oxf574	11-Aug-07	37	Y	4	S	R	+	UK	SRR3338984
C00001492	Oxf603	26-Aug-07	37	Y	4	R	S	+	UK	ERS149936
C00001537	Oxf872	04-Dec-07	37	Y	4	S	R	+	UK	SRR3335872
C00000089	Oxf920	25-Dec-07	37	Y	4	S	R	+	UK	ERS139370
C00000554	Oxf1458	09-Aug-08	37	Y	4	S	R	+	UK	ERS149908
C00001482	Oxf1492	14-Sep-08	37	Y	4	S	R	+	UK	ERS149928
C00001483	Oxf1563	22-Oct-08	37	Y	4	S	S	+	UK	ERS149929
C00001484	Oxf1633	25-Nov-08	37	Y	4	S	R	+	UK	ERS149930
C00002730	Oxf1656-p1	08-Dec-08	37	Y	4	S	R	+	UK	ERS150079
C00001486	Oxf1677	17-Dec-08	37	Y	4	S	R	+	UK	ERS149932
C00001487	Oxf1685	28-Dec-08	37	Y	4	S	R	+	UK	ERS149933

C00005871	1922-p2	27-May-09	37	Y	4	S	S	+	UK	SRR3339304
C00005732	2148-p1	21-Sep-09	37	Y	4	R	S	+	UK	ERS243550
C00005738	2283-p1	08-Dec-09	37	Y	4	S	S	+	UK	ERS346907
C00005743	2370-p1	25-Jan-10	37	Y	4	S	R	+	UK	ERS346910
C00004329	2386-p1	04-Feb-10	37	Y	4	R	S	+	UK	ERS227404
C00004341	2429-p1	26-Feb-10	37	Y	4	R	S	+	UK	ERS346710
C00004362	2637-p1	05-Jul-10	37	Y	4	S	R	+	UK	ERS346727
C00008152	2797-p1	17-Nov-10	37	Y	4	S	S	+	UK	ERS347288
C00008301	2853-p1	14-Jan-11	37	Y	4	S	S	+	UK	ERS347328
C00008108	2878-p1	08-Feb-11	37	Y	4	S	S	+	UK	ERS243549
C00008338	2897-p1	08-Mar-11	37	Y	4	S	S	+	UK	ERS347358
C00008303	2904-p1	14-Mar-11	37	Y	4	S	S	+	UK	ERS347330
C00008339	2908-p1	15-Mar-11	37	Y	4	S	S	+	UK	ERS347359
C00007795	3004-p1	27-May-11	37	Y	4	R	S	+	UK	SRR3338741
C00009880	3106-p1	20-Sep-11	37	Y	4	R	S	+	UK	SRR3337683
C00009824	3113-p1	02-Oct-11	37	Y	4	R	S	+	UK	SRR3335949
C00011267	3196-p1	31-Jan-12	37	Y	4	R	S	+	UK	ERS240775
C00011197	3206-p1	13-Feb-12	37	Y	4	R	S	+	UK	ERS243551
C00014300	3207-p1	13-Feb-12	37	Y	4	S	S	+	UK	SRR3335445
C00011789	3226-p1	15-Mar-12	37	Y	4	S	S	+	UK	SRR3338238
C00000224	Oxf1307	29-May-08	41	N	2	S	S	+	UK	ERS139382
C00002505	Oxf1802b	27-Feb-09	41	N	2	S	S	+	UK	SRR3338109
C00002516	Oxf2287	09-Dec-09	41	N	2	S	S	+	UK	SRR3335197
C00021145	26-p1	26-Sep-06	42	Y	1	R	S	+	UK	SRR3339333
C00021152	64-p1	23-Oct-06	42	Y	1	R	S	+	UK	SRR3335506
C00021154	68-p1	24-Oct-06	42	Y	1	R	S	+	UK	SRR3335826
C00021174	149-p1	07-Dec-06	42	Y	1	R	S	+	UK	SRR3335889
C00001536	Oxf135	09-Jan-07	42	Y	1	R	S	+	UK	ERS149957
C00021169	138-p1	10-Jan-07	42	Y	1	R	S	+	UK	SRR3338653
C00021172	144-p1	16-Jan-07	42	Y	1	R	S	+	UK	SRR3335850

C00021175	159-p1	28-Jan-07	42	Y	1	R	S	+	UK	SRR3335571
C00021178	166-p1	30-Jan-07	42	Y	1	R	S	+	UK	SRR3337592
C00021180	168-p1	01-Feb-07	42	Y	1	R	S	+	UK	SRR3338631
C00021183	176-p1	01-Feb-07	42	Y	1	R	S	+	UK	SRR3338500
C00021181	174-p1	05-Feb-07	42	Y	1	R	S	+	UK	SRR3338733
C00021182	175-p1	05-Feb-07	42	Y	1	R	S	+	UK	SRR3338142
C00021184	179-p1	07-Feb-07	42	Y	1	R	S	+	UK	SRR3338681
C00021185	200-p1	18-Feb-07	42	Y	1	R	S	+	UK	SRR3335501
C00021186	204-p1	18-Feb-07	42	Y	1	R	S	+	UK	SRR3338747
C00005994	219-p1	02-Mar-07	42	Y	1	R	S	+	UK	ERS227385
C00005998	235a-p1	06-Mar-07	42	Y	1	R	S	+	UK	ERS347076
C00005999	244-p1	08-Mar-07	42	Y	1	R	S	+	UK	SRR3338484
C00007830	243-p2	08-Mar-07	42	Y	1	R	S	+	UK	SRR3339391
C00006001	269-p1	23-Mar-07	42	Y	1	R	S	+	UK	SRR3337513
C00000184	Oxf277-1	28-Mar-07	42	Y	1	R	S	+	UK	SRR3338262
558a6d71-97a5-4484-8374-f2fbd8afa321	f6d6f7_4	19-Apr-07	42	Y	1	R	S	+	UK	SRR3317179
C00003958	Oxf348-p1	02-May-07	42	Y	1	R	S	+	UK	ERS346604
C00006008	366-p1	13-May-07	42	Y	1	R	S	+	UK	SRR3338660
C00001465	Oxf473	09-Jul-07	42	Y	1	R	S	+	UK	ERS149919
C00006447	530-p1	27-Jul-07	42	Y	1	R	S	+	UK	SRR3338574
C00000046	Oxf539	31-Jul-07	42	Y	1	R	S	+	UK	ERS149774
C00002740	Oxf644-p1	06-Sep-07	42	Y	1	R	S	+	UK	ERS150087
C00000048	Oxf659	10-Sep-07	42	Y	1	R	S	+	UK	ERS149776
C00000049	Oxf671	14-Sep-07	42	Y	1	R	S	+	UK	ERS149777
C00000051	Oxf704	23-Sep-07	42	Y	1	R	S	+	UK	ERS149779
C00002746	Oxf765-p1	22-Oct-07	42	Y	1	R	S	+	UK	ERS150093
C00002747	Oxf770-p1	24-Oct-07	42	Y	1	R	S	+	UK	ERS150094
C00002749	Oxf777-p1	24-Oct-07	42	Y	1	R	S	+	UK	ERS150096
C00000060	Oxf782	26-Oct-07	42	Y	1	R	S	+	UK	ERS149787
C00002750	Oxf790-p1	31-Oct-07	42	Y	1	R	S	+	UK	ERS150097
C00002751	Oxf793-p1	05-Nov-07	42	Y	1	R	S	+	UK	ERS150098

C00002755	Oxf808-p1	10-Nov-07	42	Y	1	R	S	+	UK	ERS150101
C00003971	Oxf829-p1	17-Nov-07	42	Y	1	R	S	+	UK	SRR3335957
C00002757	Oxf837-p1	21-Nov-07	42	Y	1	R	S	+	UK	ERS150103
C00002764	Oxf960-p1	10-Jan-08	42	Y	1	R	S	+	UK	ERS150110
C00004513	1036-9-p1	31-Jan-08	42	Y	1	R	S	+	UK	SRR3338766
C00002774	Oxf1109-p1	04-Mar-08	42	Y	1	R	S	+	UK	ERS150119
C00002777	Oxf1123-p1	11-Mar-08	42	Y	1	R	S	+	UK	ERS150121
C00002779	Oxf1154-p1	24-Mar-08	42	Y	1	R	S	+	UK	ERS150123
C00002781	Oxf1202-p1	16-Apr-08	42	Y	1	R	S	+	UK	ERS150125
C00006234	1260-p2	02-May-08	42	Y	1	R	S	+	UK	ERS347080
C00001491	Oxf1274	07-May-08	42	Y	1	R	S	+	UK	ERS149935
C00002789	Oxf1290-p1	17-May-08	42	Y	1	R	S	+	UK	ERS150132
C00002790	Oxf1379-p1	06-Jul-08	42	Y	1	S	S	+	UK	ERS150133
C00002791	Oxf1387-p1	10-Jul-08	42	Y	1	R	S	+	UK	ERS150134
C00002792	Oxf1408-p1	22-Jul-08	42	Y	1	R	S	+	UK	ERS150135
C00002795	Oxf1446-p1	18-Aug-08	42	Y	1	R	S	+	UK	ERS150138
C00002805	Oxf1497-p1	16-Sep-08	42	Y	1	R	S	+	UK	ERS150142
C00002806	Oxf1498-p1	17-Sep-08	42	Y	1	R	S	+	UK	ERS150143
C00002807	Oxf1499-p1	17-Sep-08	42	Y	1	S	S	+	UK	ERS150144
C00002810	Oxf1594-p1	10-Nov-08	42	Y	1	R	S	+	UK	ERS150147
C00002811	Oxf1683-p1	22-Dec-08	42	Y	1	R	S	+	UK	ERS150148
C00002812	Oxf1725-p1	19-Jan-09	42	Y	1	R	S	+	UK	ERS150149
C00002813	Oxf1753-p1	04-Feb-09	42	Y	1	R	S	+	UK	ERS150150
C00002814	Oxf1847-p1	30-Mar-09	42	Y	1	R	S	+	UK	ERS150151
C00002815	Oxf1868-p1	15-Apr-09	42	Y	1	R	S	+	UK	ERS150152
C00002816	Oxf1885-p1	27-Apr-09	42	Y	1	R	S	+	UK	ERS150153
C00002817	Oxf1926a-p1	28-May-09	42	Y	1	R	S	+	UK	ERS150154
C00002819	Oxf1994-p1	07-Jul-09	42	N	1	R	S	+	UK	ERS150156
C00002825	Oxf2100-p1	27-Aug-09	42	N	1	R	S	+	UK	ERS150162
C00002840	Oxf2263-p1	26-Nov-09	42	Y	1	R	S	+	UK	ERS150176
C00008340	2498-p1	05-Apr-10	42	Y	1	R	S	+	UK	ERS347360

C00009976	2543-p2	29-Apr-10	42	Y	1	R	S	+	UK	ERS347384
C00002885	Oxf2626-p1	22-Jun-10	42	Y	1	R	S	+	UK	ERS150212
C00008102	2734-p1	17-Sep-10	42	Y	1	R	S	+	UK	ERS347257
C00013887	3457-p1	03-Jun-12	42	Y	1	S	S	+	UK	SRR3337599
C00014314	3479-p1	23-Jun-12	42	Y	1	S	S	+	UK	SRR3335426
C00005918	405b-p1	04-Jun-07	43	N	1	S	S	+	UK	SRR3335201
C00010626	932-p2	29-Dec-07	43	N	1	S	S	+	UK	SRR3339378
C00006404	1370-p1	29-Jun-08	43	N	1	S	S	+	UK	ERS347181
C00000516	Oxf1844	27-Mar-09	43	N	1	S	S	+	UK	ERS139385
C00006258	1935-p1	03-Jun-09	43	N	1	S	S	+	UK	ERS347098
C00006302	2265-p1	27-Nov-09	43	N	1	S	S	+	UK	ERS347128
C00005992	2687-p1	30-Jul-10	43	N	1	S	S	+	UK	ERS347075
C00007832	3014-p1	08-Jun-11	43	N	1	S	S	+	UK	SRR3335525
C00007835	3039-p1	13-Jul-11	43	N	1	S	S	+	UK	SRR3335959
C00015491	3630-p1	05-Oct-12	43	N	1	S	S	+	UK	SRR3335280
C00012859	3663-p1	17-Oct-12	43	N	1	S	S	+	UK	SRR3337731
C00021143	17-p1	21-Sep-06	44	Y	1	S	S	+	UK	SRR3335955
C00021155	80-p1	01-Nov-06	44	Y	1	S	S	+	UK	SRR3337742
C00021167	132-p1	06-Jan-07	44	Y	1	S	S	+	UK	SRR3335182
C00000043	Oxf157	25-Jan-07	44	Y	1	S	S	+	UK	ERS149771
C00000024	Oxf466	05-Jul-07	44	Y	1	S	S	+	UK	ERS139366
C00003945	Oxf467-p1	06-Jul-07	44	Y	1	S	S	+	UK	SRR3335821
C00002741	Oxf662-p1	11-Sep-07	44	Y	1	S	S	+	UK	ERS150088
C00002745	Oxf755-p1	18-Oct-07	44	Y	1	S	S	+	UK	ERS150092
C00002754	Oxf802-p1	08-Nov-07	44	Y	1	S	S	+	UK	ERS150100
C00000041	Oxf821a	14-Nov-07	44	Y	1	S	S	+	UK	ERS149769
C00006233	838-p2	21-Nov-07	44	Y	1	S	S	+	UK	ERS347079
C00002758	Oxf875-p1	05-Dec-07	44	Y	1	S	S	+	UK	ERS150104
C00002765	Oxf978-p1	17-Jan-08	44	Y	1	S	S	+	UK	ERS150111
C00000477	Oxf990	20-Jan-08	44	Y	1	S	S	+	UK	ERS139383

C00002766	Oxf1005-p1	23-Jan-08	44	Y	1	S	S	+	UK	ERS150112
C00000062	Oxf1073	21-Feb-08	44	Y	1	S	S	+	UK	ERS149788
C00002771	Oxf1090-p1	28-Feb-08	44	Y	1	S	S	+	UK	ERS150116
C00002773	Oxf1106-p1	03-Mar-08	44	Y	1	S	S	+	UK	ERS150118
C00028683	1114-p3	05-Mar-08	44	Y	1	S	S	+	UK	SRR3337660
C00002780	Oxf1189-p1	13-Apr-08	44	Y	1	S	S	+	UK	ERS150124
C00002804	Oxf1197-p1	15-Apr-08	44	Y	1	S	S	+	UK	ERS150141
C00002783	Oxf1244-p1	30-Apr-08	44	Y	1	S	S	+	UK	ERS150127
C00021196	1254-p3	01-May-08	44	Y	1	S	S	+	UK	SRR3339026
C00006235	1309-p2	29-May-08	44	Y	1	S	S	+	UK	ERS347081
C00001629	Oxf1343	09-Jun-08	44	Y	1	S	S	+	UK	ERS150019
C00001594	Oxf1357	21-Jun-08	44	Y	1	S	S	+	UK	ERS149993
C00001595	Oxf1361	25-Jun-08	44	Y	1	S	S	+	UK	ERS149994
C00001599	Oxf1455	20-Aug-08	44	Y	1	S	S	+	UK	ERS149998
C00002727	Oxf1502-p1	19-Sep-08	44	Y	1	S	S	+	UK	ERS150076
C00002728	Oxf1575-p1	26-Oct-08	44	Y	1	S	S	+	UK	ERS150077
C00001459	Oxf1589	04-Nov-08	44	Y	1	S	S	+	UK	ERS149914
C00002729	Oxf1637-p1	28-Nov-08	44	Y	1	S	S	+	UK	ERS150078
C00001596	Oxf1649	05-Dec-08	44	Y	1	S	S	+	UK	ERS149995
C00001598	Oxf1825	10-Mar-09	44	Y	1	S	S	+	UK	ERS149997
C00001600	Oxf1842	18-Mar-09	44	Y	1	S	S	+	UK	ERS243745
C00001601	Oxf1852	01-Apr-09	44	Y	1	S	S	+	UK	ERS149999
C00005951	1884-p1	24-Apr-09	44	Y	1	S	S	+	UK	SRR3338644
C00002733	Oxf1906-p1	12-May-09	44	Y	1	S	S	+	UK	ERS150082
C00001606	Oxf2058	13-Aug-09	44	Y	1	S	S	+	UK	ERS150002
C00001607	Oxf2097	24-Aug-09	44	Y	1	S	S	+	UK	ERS150003
C00001609	Oxf2127	11-Sep-09	44	Y	1	S	S	+	UK	ERS243746
C00001610	Oxf2153a	27-Sep-09	44	Y	1	S	S	+	UK	ERS150004
C00001612	Oxf2161	05-Oct-09	44	Y	1	S	S	+	UK	ERS150006
C00001613	Oxf2238	09-Nov-09	44	Y	1	S	S	+	UK	ERS150007
C00002842	Oxf2297-p1	15-Dec-09	44	Y	1	S	S	+	UK	ERS150178

C00001614	Oxf2364	20-Jan-10	44	Y	1	S	S	+	UK	ERS150008
C00001615	Oxf2407	16-Feb-10	44	Y	1	S	S	+	UK	ERS150009
C00002852	Oxf2406-p1	16-Feb-10	44	Y	1	S	S	+	UK	ERS150186
C00001616	Oxf2440	25-Feb-10	44	Y	1	S	S	+	UK	ERS150010
C00001617	Oxf2449	09-Mar-10	44	Y	1	S	S	+	UK	ERS150011
C00001618	Oxf2458a	16-Mar-10	44	Y	1	S	S	+	UK	ERS150012
C00001619	Oxf2472	22-Mar-10	44	Y	1	S	S	+	UK	ERS150013
C00003943	Oxf2526-p1	19-Apr-10	44	Y	1	S	S	+	UK	ERS243733
C00002866	Oxf2528-p1	20-Apr-10	44	Y	1	S	S	+	UK	ERS150197
C00002873	Oxf2584-p1	23-May-10	44	Y	1	S	S	+	UK	ERS150202
C00002877	Oxf2595-p1	31-May-10	44	Y	1	S	S	+	UK	ERS150206
C00009905	2596-p2	01-Jun-10	44	Y	1	S	S	+	UK	SRR3338743
C00002882	Oxf2618-p1	15-Jun-10	44	Y	1	S	S	+	UK	ERS150209
C00002883	Oxf2619-p1	15-Jun-10	44	Y	1	S	S	+	UK	ERS150210
C00003948	Oxf2675-p1	20-Jul-10	44	Y	1	S	S	+	UK	ERS346598
C00003950	Oxf2683-p1	27-Jul-10	44	Y	1	S	S	+	UK	ERS346600
C00021199	2701-p2	12-Aug-10	44	Y	1	S	S	+	UK	SRR3338826
C00008309	2761-p1	09-Oct-10	44	Y	1	S	S	+	UK	ERS243734
C00008275	2809-p1	30-Nov-10	44	Y	1	S	S	+	UK	ERS243735
C00008265	2850-p1	09-Jan-11	44	Y	1	S	R	+	UK	ERS347303
C00008289	2852-p1	10-Jan-11	44	Y	1	S	S	+	UK	ERS243747
C00008349	2858-p1	19-Jan-11	44	Y	1	S	S	+	UK	ERS243736
C00008155	2871-p1	05-Feb-11	44	Y	1	S	S	+	UK	ERS347291
C00008132	2881-p1	14-Feb-11	44	Y	1	S	S	+	UK	ERS243748
C00008109	2888-p1	23-Feb-11	44	Y	1	S	S	+	UK	ERS243737
C00008121	2889-p1	23-Feb-11	44	Y	1	S	S	+	UK	ERS347267
C00008134	2932-p1	19-Apr-11	44	Y	1	S	S	+	UK	ERS243738
C00008943	2950-p1	12-May-11	44	Y	1	S	S	+	UK	SRR3338471
C00007846	3032-p1	30-Jun-11	44	Y	1	S	S	+	UK	SRR3339024
C00007776	3042-p1	19-Jul-11	44	Y	1	S	S	+	UK	SRR3338265
C00007848	3047-p1	22-Jul-11	44	Y	1	S	S	+	UK	ERS347224

C00007851	3071-p1	15-Aug-11	44	Y	1	S	S	+	UK	SRR3334827
C00009835	3103-p1	21-Sep-11	44	Y	1	S	S	+	UK	SRR3335891
C00010640	3112-p1	30-Sep-11	44	Y	1	S	S	+	UK	SRR3337671
C00010642	3118-p1	06-Oct-11	44	Y	1	S	S	+	UK	SRR3337737
C00009850	3152-p1	08-Oct-11	44	Y	1	S	S	+	UK	SRR3334933
C00010655	3133-p1	03-Nov-11	44	Y	1	S	S	+	UK	SRR3338098
C00011265	3179-p2	01-Jan-12	44	Y	1	S	S	+	UK	SRR3335849
C00011781	3218-p1	27-Feb-12	44	Y	1	S	S	+	UK	SRR3338758
C00011785	3222-p1	01-Mar-12	44	Y	1	S	S	+	UK	SRR3337719
C00014304	3233-p1	24-Mar-12	44	Y	1	S	S	+	UK	SRR3338153
C00009452	3243-p1	10-Apr-12	44	Y	1	S	S	+	UK	SRR3335154
C00009462	3248-p1	15-Apr-12	44	Y	1	S	S	+	UK	SRR3335459
C00012704	3311-p1	16-Apr-12	44	Y	1	S	S	+	UK	SRR3335989
C00013844	3408-p1	13-May-12	44	Y	1	S	S	+	UK	SRR3339013
C00014343	3523-p1	03-Aug-12	44	Y	1	S	S	+	UK	SRR3334834
C00015461	3591-p1	13-Sep-12	44	Y	1	S	S	+	UK	SRR3339348
C00015492	3631-p1	01-Oct-12	44	Y	1	S	S	+	UK	SRR3336101
C00015519	3671-p1	15-Oct-12	44	Y	1	S	S	+	UK	SRR3335430
C00020411	3840-p1	03-Feb-13	44	Y	1	S	S	+	UK	SRR3339331
C00020403	3827-p1	05-Feb-13	44	Y	1	S	S	+	UK	SRR3337794
adf47793-fed7-474a-be93-b74b092e3017	f6d6f7_70	21-Mar-13	44	Y	1	S	S	+	UK	SRR3317188
27c0e18c-36e9-4c11-b632-ca4982bb12d6	f6d6f7_76	20-Jun-13	44	Y	1	S	S	+	UK	SRR3317171
C00021156	92-p1	16-Nov-06	45	N	1	S	S	+	UK	SRR3338765
C00021158	103-p1	24-Nov-06	45	N	1	S	S	+	UK	SRR3335909
C00021190	333-p2	28-Apr-07	45	N	1	S	S	+	UK	SRR3335233
C00001637	Oxf887	11-Dec-07	45	N	1	S	S	+	UK	ERS150023
C00005716	954-p1	08-Jan-08	45	N	1	S	S	+	UK	ERS346890
C00005719	1165-p1	30-Mar-08	45	N	1	S	S	+	UK	ERS346892
C00005721	1577-p1	28-Oct-08	45	N	1	S	S	+	UK	ERS346894
C00006363	1578-p1	28-Oct-08	45	N	1	S	S	+	UK	ERS347158

C00009941	1837a-p2	16-Mar-09	45	N	1	S	S	+	UK	ERS347379
C00004014	Oxf2050b-p1	10-Aug-09	45	N	1	S	S	+	UK	ERS346630
C00005735	2260-p1	25-Nov-09	45	N	1	S	S	+	UK	ERS346904
C00005740	2294-p1	14-Dec-09	45	N	1	S	S	+	UK	ERS139413
C00004333	2402-p1	13-Feb-10	45	N	1	S	S	+	UK	ERS346705
C00008144	2882-p1	15-Feb-11	45	N	1	S	S	+	UK	ERS347281
C00008967	3091-p1	08-Sep-11	45	N	1	S	S	+	UK	SRR3336050
C00013833	3393-p1	16-May-12	45	N	1	S	S	+	UK	SRR3338959
9e5d32e5-bf6c-4965-bfbf-5d605aac7920	f6d6f7_78	25-Jul-13	45	N	1	S	S	+	UK	SRR3317186
C00005678	571-p1	12-Aug-07	46	N	1	S	S	+	UK	ERS346863
C00005931	670-p1	13-Sep-07	46	N	1	S	S	+	UK	ERS347044
C00006397	1228-p1	25-Apr-08	46	N	1	S	S	+	UK	ERS347175
C00006400	1285a-p1	13-May-08	46	N	1	S	S	+	UK	ERS347178
C00005972	2476-p1	23-Mar-10	46	N	1	S	S	+	UK	SRR3334923
C00007840	3078-p1	21-Jul-11	46	N	1	S	S	+	UK	SRR3339029
C00007867	3148a-p1	21-Nov-11	46	N	1	S	S	+	UK	SRR3338509
C00011205	3174-p1	27-Dec-11	46	N	1	S	S	+	UK	SRR3335251
C00009003	3182-p1	07-Jan-12	46	N	1	S	S	+	UK	SRR3334930
C00012687	3283-p1	12-Apr-12	46	N	1	S	S	+	UK	SRR3338915
C00014331	3506-p1	03-Aug-12	46	N	1	S	S	+	UK	SRR3338557
C00015450	3574-p1	05-Sep-12	46	N	1	S	S	+	UK	SRR3335234
C00015453	3579-p1	07-Sep-12	46	N	1	S	S	+	UK	SRR3338942
C00016339	3677-p1	23-Oct-12	46	N	1	S	S	+	UK	SRR3338485
C00020118	3745-p1	01-Dec-12	46	N	1	S	S	+	UK	SRR3337612
C00005984	2621b-p1	15-Jun-10	47	N	2	S	S	+	UK	ERS139414
C00006353	2167-p1	11-Oct-09	48	N	1	S	S	-	UK	ERS139419
C00010634	2250-p2	15-Nov-09	48	N	1	S	S	+	UK	ERS347401
C00021144	18-p1	22-Sep-06	49	Y	1	S	S	+	UK	SRR3337604
C00021162	112-p1	02-Dec-06	49	Y	1	S	S	+	UK	SRR3338899
C00006018	409-p1	06-Jun-07	49	Y	1	S	S	+	UK	SRR3335485

C00006025	480-p1	11-Jul-07	49	Y	1	S	S	+	UK	SRR3335922
C00006027	498-p1	18-Jul-07	49	Y	1	S	S	+	UK	ERS227365
C00006325	593-p1	14-Aug-07	49	Y	1	S	S	+	UK	ERS139417
C00005713	702-p1	22-Sep-07	49	Y	1	S	S	+	UK	ERS346887
C00005714	797-p1	06-Nov-07	49	Y	1	S	S	+	UK	ERS346888
C00005715	810-p1	09-Nov-07	49	Y	1	S	S	+	UK	ERS346889
C00009917	981-p2	18-Jan-08	49	Y	1	S	S	+	UK	ERS347374
C00005725	1747-p1	01-Feb-09	49	Y	1	S	S	+	UK	ERS346898
C00004199	1972-p3	26-Jun-09	49	Y	1	S	S	+	UK	ERS346681
C00005727	1986-p1	05-Jul-09	49	Y	1	S	S	+	UK	ERS227422
C00005728	2000-p1	12-Jul-09	49	Y	1	S	S	+	UK	ERS346899
C00005729	2062-p1	14-Aug-09	49	Y	1	S	S	+	UK	ERS346900
C00005733	2185-p1	19-Oct-09	49	Y	1	S	S	+	UK	ERS346902
C00005736	2269-p1	02-Dec-09	49	Y	1	S	S	+	UK	ERS346905
C00005742	2362-p1	19-Jan-10	49	Y	1	S	S	+	UK	ERS227363
C00004345	2485-p1	26-Mar-10	49	Y	1	S	S	+	UK	ERS346713
C00008305	2544-p1	30-Apr-10	49	Y	1	S	S	+	UK	ERS347332
C00008259	2700-p1	12-Aug-10	49	Y	1	S	S	+	UK	ERS347297
C00008100	2703-p1	13-Aug-10	49	Y	1	S	S	+	UK	ERS347255
C00008261	2756-p1	05-Oct-10	49	Y	1	S	S	+	UK	ERS347299
C00008286	2790-p1	09-Nov-10	49	Y	1	S	S	+	UK	ERS347317
C00008129	2804-p1	22-Nov-10	49	Y	1	S	S	+	UK	ERS347271
C00008156	2883-p1	16-Feb-11	49	Y	1	S	S	+	UK	ERS347292
C00007845	3024-p1	23-Jun-11	49	Y	1	S	S	+	UK	SRR3338547
C00012705	3313-p1	16-Apr-12	49	Y	1	S	S	+	UK	SRR3338926
C00013851	3416-p1	29-May-12	49	Y	1	S	S	+	UK	SRR3338657
C00013874	3439-p1	11-Jun-12	49	Y	1	S	S	+	UK	SRR3339387
C00014369	3558-p1	22-Aug-12	49	Y	1	S	S	+	UK	SRR3335211
C00014371	3562-p1	03-Sep-12	49	Y	1	S	S	+	UK	SRR3335612
C00015512	3664-p1	17-Oct-12	49	Y	1	S	S	+	UK	SRR3337785

b2e2c128-84b6-4498-b7a6-5ea4b52871a3	f6d6f7_60	30-Jan-13	49	Y	1	S	S	+	UK	SRR3317191
C00006277	2109-p1	30-Aug-09	50	N	1	S	S	+	UK	ERS227379
C00006416	1472-p1	02-Sep-08	51	N	1	S	S	+	UK	ERS347193
C00006270	2011a-p1	17-Jul-09	51	N	1	S	S	+	UK	ERS227388
C00008090	2731-p1	12-Sep-10	51	N	1	S	S	+	UK	ERS347248
C00008140	2794-p1	14-Nov-10	51	N	1	S	S	+	UK	ERS347277
C00020126	3753-p1	10-Dec-12	51	N	1	S	S	+	UK	SRR3338524
C00006396	1223-p1	24-Apr-08	52	N	1	S	S	+	UK	ERS347174
C00021171	143-p1	16-Jan-07	53	N	1	S	S	+	UK	SRR3338196
C00006379	737-p1	09-Oct-07	53	N	1	S	S	+	UK	ERS347165
C00005953	962-p2	11-Jan-08	53	N	1	S	S	+	UK	ERS347049
C00006419	1568-p1	23-Oct-08	53	N	1	S	S	+	UK	ERS347195
C00006267	1993-p1	07-Jul-09	53	N	1	S	S	+	UK	ERS227430
C00006271	2038a-p1	02-Aug-09	53	N	1	S	S	+	UK	ERS347106
C00006286	2169a-p1	13-Oct-09	53	N	1	S	S	+	UK	ERS347117
C00008279	2901-p1	11-Mar-11	53	N	1	S	S	+	UK	ERS347312
C00010652	3130-p1	24-Oct-11	53	N	1	S	S	+	UK	SRR3338533
C00015515	3667-p1	19-Oct-12	53	N	1	S	S	+	UK	SRR3338546
C00000567	Oxf42	02-Oct-06	54	Y	1	S	S	+	UK	ERS149913
C00000521	Oxf666	12-Sep-07	54	Y	1	S	S	+	UK	ERS243564
C00000528	Oxf749	16-Oct-07	54	Y	1	S	S	+	UK	ERS149896
C00000525	Oxf860	29-Nov-07	54	Y	1	S	S	+	UK	ERS149895
C00000529	Oxf1396	14-Jul-08	54	Y	1	S	S	+	UK	ERS139387
C00000526	Oxf1567	23-Oct-08	54	Y	1	S	S	+	UK	ERS139386
C00000524	Oxf1572	27-Oct-08	54	Y	1	S	S	+	UK	ERS149894
C00000527	Oxf1794	25-Feb-09	54	Y	1	S	S	+	UK	ERS243565
C00000532	Oxf1882a	24-Apr-09	54	Y	1	S	S	+	UK	ERS149898
C00000522	Oxf1907	12-May-09	54	Y	1	S	S	+	UK	ERS149892
C00000523	Oxf1931a	01-Jun-09	54	Y	1	S	S	+	UK	ERS149893
C00002476	Oxf2055	11-Aug-09	54	N	1	S	S	+	UK	ERS243566

C00002479	Oxf2478	25-Mar-10	54	Y	1	S	S	+	UK	ERS139403
C00008120	2880-p1	12-Feb-11	54	Y	1	S	S	+	UK	ERS243567
C00008025	2917-p1	31-Mar-11	54	Y	1	S	S	+	UK	ERS243568
C00008063	3090-p1	13-Sep-11	54	Y	1	R	S	+	UK	ERS243569
C00009874	3105-p1	20-Sep-11	54	Y	1	S	S	+	UK	SRR3335940
C00009827	3160-p1	05-Dec-11	54	Y	1	S	S	+	UK	SRR3338634
C00009852	3170-p1	16-Dec-11	54	Y	1	S	S	+	UK	SRR3338127
C00011254	3214_p1	20-Feb-12	54	Y	1	S	S	+	UK	SRR3338745
C00013827	3386-p1	25-Apr-12	54	Y	1	S	S	+	UK	SRR3338185
C00013834	3396-p1	23-May-12	54	Y	1	S	S	+	UK	SRR3338162
C00014325	3496-p1	06-Jul-12	54	Y	1	S	S	+	UK	SRR3338221
C00005718	1105-p1	03-Mar-08	55	Y	1	S	S	+	UK	ERS346891
C00009962	1566-p1	23-Oct-08	55	Y	1	S	S	+	UK	SRR3338174
C00005722	1590-p1	06-Nov-08	55	Y	1	S	S	+	UK	ERS346895
C00005723	1617-p1	16-Nov-08	55	Y	1	S	S	+	UK	ERS346896
C00005730	2139-p1	17-Sep-09	55	Y	1	S	S	+	UK	ERS227362
C00005731	2147a-p1	20-Sep-09	55	Y	1	S	S	+	UK	ERS346901
C00005734	2218-p1	02-Nov-09	55	Y	1	S	S	+	UK	ERS346903
C00005737	2276-p1	06-Dec-09	55	Y	1	S	S	+	UK	ERS346906
C00005739	2291-p1	10-Dec-09	55	Y	1	S	S	+	UK	ERS346908
C00004325	2372-p1	26-Jan-10	55	Y	1	S	S	+	UK	ERS346701
C00004327	2380-p1	02-Feb-10	55	Y	1	S	S	+	UK	ERS346703
C00004342	2454-p1	12-Mar-10	55	Y	1	S	S	+	UK	ERS346711
C00004343	2469a-p1	21-Mar-10	55	Y	1	S	S	+	UK	ERS346712
C00008262	2765-p1	13-Oct-10	55	Y	1	S	S	+	UK	ERS347300
C00008026	2928-p1	10-Apr-11	55	Y	1	S	S	+	UK	SRR3338165
C00007803	3067-p1	12-Aug-11	55	Y	1	S	S	+	UK	SRR3334876
C00014376	3569-p1	04-Sep-12	55	Y	1	S	S	+	UK	SRR3338488
C00016368	3715-p1	13-Nov-12	55	Y	1	S	S	+	UK	SRR3335120
C00021206	3868-p1	15-Mar-13	55	Y	1	S	S	+	UK	SRR3335272

C00006380	739-p1	13-Oct-07	56	N	1	S	S	+	UK	ERS347166
C00006420	1586-p1	31-Oct-08	56	N	1	S	S	+	UK	ERS347196
C00006276	2099-p1	27-Aug-09	56	N	1	S	S	+	UK	ERS347110
C00008153	2825-p1	10-Dec-10	56	N	1	S	S	+	UK	ERS347289
C00009026	2948-p2	12-May-11	56	N	1	S	S	+	UK	SRR3335996
C00012706	3314-p1	16-Apr-12	56	N	1	S	S	+	UK	SRR3338780
C00000157	Oxf1341a	08-Jun-08	57	N	1	S	S	+	UK	ERS149835
C00000082	Oxf1596	11-Nov-08	57	N	1	S	S	+	UK	ERS149805
C00000563	Oxf84	09-Nov-06	58	Y	1	S	S	+	UK	ERS243556
C00000543	Oxf447	20-Jun-07	58	Y	1	S	S	+	UK	ERS243557
C00000533	Oxf701	21-Sep-07	58	Y	1	S	S	+	UK	ERS149899
C00000509	Oxf707	24-Sep-07	58	Y	1	S	S	+	UK	ERS139384
C00000541	Oxf884	07-Dec-07	58	Y	1	S	S	+	UK	ERS240776
C00000537	Oxf946	04-Jan-08	58	Y	1	S	S	+	UK	ERS149901
C00000178	Oxf963-3	11-Jan-08	58	Y	1	S	S	+	UK	ERS149841
C00000538	Oxf1058	11-Feb-08	58	Y	1	S	S	+	UK	ERS243558
C00000540	Oxf1064	17-Feb-08	58	Y	1	S	S	+	UK	ERS243559
C00002473	Oxf1150	23-Mar-08	58	Y	1	S	S	+	UK	ERS150029
C00000053	Oxf1182a	09-Apr-08	58	Y	1	S	S	+	UK	ERS149781
C00000544	Oxf1647	04-Dec-08	58	Y	1	S	S	+	UK	ERS149904
C00000534	Oxf1730	21-Jan-09	58	Y	1	S	S	+	UK	ERS227383
C00000542	Oxf1769	09-Feb-09	58	Y	1	S	S	+	UK	ERS149903
C00002474	Oxf2032	30-Jul-09	58	Y	1	S	S	+	UK	ERS150030
C00002475	Oxf2039	04-Aug-09	58	Y	1	S	S	+	UK	ERS150031
C00002477	Oxf2076	19-Aug-09	58	Y	1	S	S	+	UK	ERS150032
C00002478	Oxf2166	11-Oct-09	58	Y	1	S	S	+	UK	ERS150033
C00002488	Oxf2368	24-Jan-10	58	Y	1	S	S	+	UK	ERS150039
C00008341	2630-p1	27-Jun-10	58	Y	1	S	S	+	UK	ERS243560
C00008080	2786-p1	06-Nov-10	58	Y	1	S	S	+	UK	ERS243561
C00008955	2951-p1	17-May-11	58	Y	1	S	S	+	UK	ERS243562

C00007833	3023-p1	20-Jun-11	58	Y	1	S	S	+	UK	SRR3338713
C00008060	3098-p1	02-Sep-11	58	Y	1	S	S	+	UK	ERS243563
C00011193	3155-p1	28-Nov-11	58	Y	1	S	S	+	UK	SRR3336048
C00011245	3211-p1	11-Feb-12	58	Y	1	S	S	+	UK	SRR3338205
C00016348	3688-p1	30-Oct-12	58	Y	1	S	S	+	UK	SRR3337727
C00021210	3875-p1	12-Mar-13	58	Y	1	S	S	+	UK	SRR3337796
C00001463	Oxf1893	03-May-09	59	N	1	S	S	+	UK	ERS139392
C00005977	2536-p1	26-Apr-10	59	N	1	S	S	+	UK	ERS347064
C00006256	1924-p1	29-May-09	60	Y	1	S	S	+	UK	ERS216168
C00002482	Oxf1918	24-May-09	63	N	1	S	S	+	UK	ERS139404
C00002483	Oxf1933	01-Jun-09	63	N	1	S	S	+	UK	ERS150034
C00002484	Oxf1948	08-Jun-09	63	N	1	S	S	+	UK	ERS150035
C00002486	Oxf2003	13-Jul-09	63	N	1	S	S	+	UK	ERS150037
C00002481	Oxf2029	26-Jul-09	63	N	1	S	S	+	UK	SRR3337645
C00005950	1964a-p1	16-Jun-09	65	N	1	S	S	+	UK	SRR3339306
C00005960	2396-p1	10-Feb-10	65	N	1	S	S	+	UK	ERS347055
C00004020	Oxf2121-p1	09-Sep-09	66	N	1	S	S	+	UK	ERS346636
C00005991	2685b-p1	29-Jul-10	66	N	1	S	S	+	UK	ERS347074
C00007991	3084-p1	03-Sep-11	66	N	1	S	S	+	UK	SRR3338490
C00005952	2041-p1	04-Aug-09	67	N	2	S	S	+	UK	ERS347048
C00011218	3184-p1	13-Jan-12	67	N	2	S	S	+	UK	SRR3335308
C00006279	2120-p1	09-Sep-09	68	N	1	S	S	+	UK	ERS347112
C00016358	3701-p1	06-Nov-12	69	N	1	S	S	+	UK	SRR3338532
C00006399	1253-p1	01-May-08	70	N	1	R	S	+	UK	ERS347177
C00006398	1233-p1	28-Apr-08	71	N	1	S	S	+	UK	ERS347176
C00005930	634-p1	02-Sep-07	72	N	1	S	S	+	UK	ERS347043
C00006307	2273-p1	04-Dec-09	72	N	1	S	S	+	UK	ERS347131
C00006312	2304-p1	20-Dec-09	72	N	1	S	S	+	UK	ERS227403
C00006244	1774-p1	11-Feb-09	73	N	1	S	S	+	UK	ERS347087

C00006381	740-p1	15-Oct-07	74	N	1	S	S	+	UK	ERS347167
C00006245	1824-p1	09-Mar-09	75	N	1	S	S	+	UK	ERS347088
C00006260	1952-p1	09-Jun-09	75	N	1	S	S	+	UK	ERS347100
C00006391	1029-p1	30-Jan-08	76	N	1	S	S	+	UK	ERS227425
C00000196	Oxf2002	14-Jul-09	77	N	1	S	S	+	UK	ERS149846
C00000197	Oxf2017	21-Jul-09	77	N	1	S	S	+	UK	ERS139381
C00006278	2118-p1	09-Sep-09	78	N	1	S	S	+	UK	ERS347111
C00008083	2838-p1	27-Dec-10	80	N	1	S	S	+	UK	ERS347243
C00014377	3571-p1	05-Sep-12	80	N	1	S	S	+	UK	SRR3339032
C00006281	2133-p1	14-Sep-09	90	N	1	S	S	+	UK	ERS347114
C00009849	3142-p1	14-Oct-11	90	N	1	S	S	+	UK	SRR3334822
C00006284	2149-p1	24-Sep-09	91	N	1	S	S	+	UK	ERS347116
C00006293	2202-p1	26-Oct-09	92	N	1	S	S	+	UK	ERS347123
C00008319	2744-p1	26-Sep-10	92	N	1	S	S	+	UK	ERS347342
C00008263	2808-p1	29-Nov-10	92	N	1	S	S	+	UK	ERS347301
C00008143	2870-p1	07-Feb-11	92	N	1	S	S	+	UK	ERS347280
C00006730	2423-p1	23-Feb-10	97	N	2	S	S	+	UK	ERS347199
C00008311	2812-p1	01-Dec-10	98	N	1	S	S	+	UK	ERS347336
C00008112	2704-p1	13-Aug-10	99	N	1	S	S	+	UK	ERS347261
C00007799	3036-p1	09-Jul-11	103	N	1	S	S	+	UK	ERS216170
C00008304	2424-p1	24-Feb-10	107	N	1	S	S	-	UK	ERS347331
C00008313	2855-p1	15-Jan-11	107	N	1	S	S	-	UK	ERS347337
C00008306	2666-p1	16-Jul-10	111	N	1	S	S	+	UK	ERS347333
C00008329	2560a-p1	11-May-10	112	N	1	S	S	+	UK	ERS347350
C00008284	2749-p1	01-Oct-10	113	N	1	S	S	+	UK	ERS347316
C00008345	2764-p1	12-Oct-10	114	N	2	S	S	+	UK	ERS347364
C00015485	3617-p1	19-Sep-12	114	N	2	S	S	+	UK	SRR3338946
C00013418	3699-p1	06-Nov-12	114	N	2	S	S	+	UK	SRR3338074

C00016366	3713-p1	13-Nov-12	114	N	2	S	S	+	UK	SRR3337789
C00014347	3528-p1	24-Jul-12	122	N	-	S	S	+	UK	SRR3336064
C00012711	3321-p1	06-May-12	133	N	1	S	S	+	UK	SRR3339298
C00011783	3220-p1	02-Mar-12	150	N	1	S	S	+	UK	SRR3338201
C00020378	3795-p1	17-Jan-13	160	N	1	S	S	+	UK	SRR3335307
C00011221	3209-p1	12-Feb-12	176	N	2	S	S	+	UK	SRR3337740
C00014309	3239-p1	29-Mar-12	246	N	1	S	S	+	UK	SRR3335509
C00007796	3011-p1	07-Jun-11	248	N	1	S	S	+	UK	ERS347211
C00006249	1855-p1	07-Apr-09	249	N	1	S	S	+	UK	SRR3335161
C00020391	3808-p1	07-Feb-13	257	N	1	S	S	+	UK	SRR3335311
C00007866	3141-p1	18-Nov-11	268	N	1	S	S	+	UK	SRR3334885
C00007868	3156-p1	29-Nov-11	268	N	1	S	S	+	UK	SRR3339324
C00008979	3092-p1	15-Sep-11	269	N	2	S	S	+	UK	SRR3339039
C00012676	3270-p1	18-May-12	270	N	1	S	S	+	UK	SRR3336087
C00020363	3776-p1	31-Dec-12	271	N	1	S	S	+	UK	SRR3334932

(b) Isolate Collection: Leeds Human Clinical Isolates.

Genome Identification Number	Isolate Name	Isolation Date	ST	Phylogeny	clade	*gyrA*	*gyrB*	PaLoc	Country	SRA Accession
C00008430	L-111-68-p1	15-Aug-10	1	Y	2	R	S	+	UK	SRR3335817
C00008405	L-110-01-p1	20-Aug-10	1	Y	2	R	S	+	UK	ERS352104
C00012096	L-L-12-7901272-p1	01-Sep-10	1	Y	2	R	S	+	UK	SRR3337690
C00008052	L-112-4b-p1	18-Sep-10	1	Y	2	R	S	+	UK	SRR3338179
C00011473	L-L-12-7901305-p1	01-Oct-10	1	Y	2	R	S	+	UK	SRR3335259
C00008359	L-113-22-p1	08-Oct-10	1	Y	2	R	S	+	UK	ERS352060
C00008371	L-114-06-p1	23-Oct-10	1	Y	2	R	S	+	UK	ERS352071
C00008383	L-114-10-p1	26-Oct-10	1	Y	2	R	S	+	UK	ERS352083
C00008053	L-114-61b-p1	01-Nov-10	1	Y	2	R	S	+	UK	SRR3335595
C00011915	L-124-42-p1	01-Nov-10	1	Y	2	R	S	+	UK	SRR3338553
C00008054	L-115-58b-p1	13-Nov-10	1	Y	2	R	S	+	UK	SRR3338517
C00008384	L-115-74-p1	16-Nov-10	1	Y	2	R	S	+	UK	ERS352084
C00011902	L-115-67-p1	16-Nov-10	1	Y	2	R	S	+	UK	SRR3336100
C00008372	L-115-70-p1	17-Nov-10	1	Y	2	R	S	+	UK	ERS352072
C00010713	L-117-06-p1	20-Nov-10	1	Y	2	R	S	+	UK	SRR3337673
C00008420	L-118-17-p2	22-Nov-10	1	Y	2	R	S	+	UK	SRR3335306
C00010709	L-116-59-p1	23-Nov-10	1	Y	2	R	S	+	UK	SRR3334832
C00008396	L-116-40-p1	26-Nov-10	1	Y	2	R	S	+	UK	SRR3335865
C00010707	L-116-38-p1	28-Nov-10	1	Y	2	R	S	+	UK	SRR3339321
C00008055	L-116-71b-p1	04-Dec-10	1	Y	2	R	S	+	UK	SRR3334869
C00010712	L-117-04-p1	07-Dec-10	1	Y	2	R	S	+	UK	SRR3338483
C00011911	L-122-43-p1	08-Dec-10	1	Y	2	R	S	+	UK	SRR3338788
C00010716	L-117-36-p1	09-Dec-10	1	Y	2	R	S	+	UK	SRR3339301
C00006879	L-117-05-p1	10-Dec-10	1	Y	2	R	S	+	UK	SRR3338738
C00011917	L-124-65-p1	13-Dec-10	1	Y	2	R	S	+	UK	SRR3334884
C00011813	L-9-06-p1	14-Dec-10	1	Y	2	R	S	+	UK	SRR3338150
C00011907	L-118-46-p1	31-Dec-10	1	Y	2	R	S	+	UK	SRR3339382

C00011908	L-118-66-p1	31-Dec-10	1	Y	2	R	S	+	UK	SRR3336096
C00010727	L-118-35-p1	02-Jan-11	1	Y	2	R	S	+	UK	SRR3335312
C00010724	L-118-20-p1	03-Jan-11	1	Y	2	R	S	+	UK	SRR3335176
C00008056	L-118-55b-p1	07-Jan-11	1	Y	2	R	S	+	UK	SRR3335502
C00008444	L-118-54-p1	07-Jan-11	1	Y	2	R	S	+	UK	SRR3338182
C00008432	L-118-53-p1	11-Jan-11	1	Y	2	R	S	+	UK	SRR3338189
C00011824	L-9-74-p1	11-Jan-11	1	Y	2	R	S	+	UK	SRR3335511
C00008409	L-119-65-p2	16-Jan-11	1	Y	2	R	S	+	UK	SRR3338622
C00008373	L-118-73-p1	17-Jan-11	1	Y	2	R	S	+	UK	ERS352073
C00011827	L-9-91-p1	18-Jan-11	1	Y	2	R	S	+	UK	SRR3338916
C00010734	L-119-19-p1	22-Jan-11	1	Y	2	R	S	+	UK	SRR3338906
C00008434	L-121-5-p1	27-Jan-11	1	Y	2	R	S	+	UK	ERS352127
C00008445	L-120-28-p1	28-Jan-11	1	Y	2	R	S	+	UK	SRR3335142
C00011833	L-10-28-p1	01-Feb-11	1	Y	2	R	S	+	UK	SRR3335168
C00008057	L-120-64b-p1	02-Feb-11	1	Y	2	R	S	+	UK	SRR3335998
C00011501	L-L-12-7901364-p1	04-Feb-11	1	Y	2	R	S	+	UK	SRR3338827
C00011838	L-10-46-p1	06-Feb-11	1	Y	2	R	S	+	UK	SRR3335212
C00008410	L-121-19-p1	09-Feb-11	1	Y	2	R	S	+	UK	ERS352107
C00011841	L-10-61-p1	11-Feb-11	1	Y	2	R	S	+	UK	SRR3334899
C00011843	L-10-65-p1	14-Feb-11	1	Y	2	R	S	+	UK	SRR3335268
C00011844	L-10-70-p1	15-Feb-11	1	Y	2	R	S	+	UK	SRR3335294
C00008058	L-122-20b-p1	23-Feb-11	1	Y	2	R	S	+	UK	SRR3335602
C00010576	L-124-58-p1	20-Mar-11	1	Y	2	R	S	+	UK	SRR3338944
C00010572	L-124-4-p1	21-Mar-11	1	Y	2	R	S	+	UK	SRR3336092
C00011860	L-11-61-p1	27-Mar-11	1	Y	2	R	S	+	UK	SRR3338978
C00011866	L-11-76-p1	01-Apr-11	1	Y	2	R	S	+	UK	SRR3339031
C00008496	L-124-48-p1	03-Apr-11	1	Y	2	R	S	+	UK	ERS352161
C00011918	L-124-66-p1	04-Apr-11	1	Y	2	R	S	+	UK	SRR3338161
C00010580	L-124-78-p1	05-Apr-11	1	Y	2	R	S	+	UK	SRR3334799
C00011870	L-11-92-p1	05-Apr-11	1	Y	2	R	S	+	UK	SRR3337743
C00010584	L-125-7-p1	10-Apr-11	1	Y	2	R	S	+	UK	SRR3338463

C00011872	L-12-08-p1	13-Apr-11	1	Y	2	R	S	+	UK	SRR3338561
C00010591	L-125-46-p1	15-Apr-11	1	Y	2	R	S	+	UK	SRR3338895
C00010600	L-126-49-p1	02-May-11	1	Y	2	R	S	+	UK	SRR3337578
C00012110	L-L-12-7901405-p1	09-May-11	1	Y	2	R	S	+	UK	SRR3337732
C00010601	L-126-50-p1	10-May-11	1	Y	2	R	S	+	UK	SRR3335227
C00011880	L-12-69-p1	12-May-11	1	Y	2	R	S	+	UK	SRR3335190
C00012111	L-L-12-7901408-p1	13-May-11	1	Y	2	R	S	+	UK	SRR3338234
C00010602	L-127-4-p1	15-May-11	1	Y	2	R	S	+	UK	SRR3335464
C00011078	L-127-59-p1	18-May-11	1	Y	2	R	S	+	UK	SRR3335114
C00010611	L-127-38-p1	23-May-11	1	Y	2	R	S	+	UK	SRR3335892
C00010613	L-127-41-p1	23-May-11	1	Y	2	R	S	+	UK	SRR3338970
C00010616	L-127-58-p1	26-May-11	1	Y	2	R	S	+	UK	SRR3338913
C00010615	L-127-51-p1	28-May-11	1	Y	2	R	S	+	UK	SRR3338609
C00011089	L-129-38-p1	12-Jun-11	1	Y	2	R	S	+	UK	SRR3339318
C00011526	L-L-12-7901427-p1	17-Jun-11	1	Y	2	R	S	+	UK	SRR3338550
C00011096	L-130-36-p1	01-Jul-11	1	Y	2	R	S	+	UK	SRR3338961
C00011106	L-131-75-p1	17-Jul-11	1	Y	2	R	S	+	UK	SRR3338589
C00012113	L-L-12-7901441-p1	19-Jul-11	1	Y	2	R	S	+	UK	SRR3338243
C00011541	L-L-12-7901444-p1	24-Jul-11	1	Y	2	R	S	+	UK	SRR3339367
C00011545	L-L-12-7901449-p1	27-Jul-11	1	Y	2	R	S	+	UK	SRR3339208
C00011549	L-L-12-7901453-p1	07-Aug-11	1	Y	2	R	S	+	UK	SRR3335557
C00011551	L-L-12-7901455-p1	09-Aug-11	1	Y	2	R	S	+	UK	SRR3337606
C00011934	L-134-54-p1	12-Aug-11	1	Y	2	R	S	+	UK	SRR3335825
C00011118	L-133-71-p1	22-Aug-11	1	Y	2	R	S	+	UK	SRR3338167
C00011114	L-133-37-p1	24-Aug-11	1	Y	2	R	S	+	UK	SRR3335910
C00011111	L-133-35-p1	25-Aug-11	1	Y	2	R	S	+	UK	SRR3338117
C00011123	L-134-35-p1	26-Aug-11	1	Y	2	R	S	+	UK	SRR3335839
C00011138	L-136-58-p1	26-Sep-11	1	Y	2	R	S	+	UK	SRR3337787
C00011670	L-L-12-7901482-p1	29-Sep-11	1	Y	2	R	S	+	UK	SRR3335827
C00011671	L-L-12-7901483-p1	03-Oct-11	1	Y	2	R	S	+	UK	SRR3335270
C00011681	L-L-12-7901495-p1	12-Oct-11	1	Y	2	R	S	+	UK	SRR3336069

C00011144	L-139-35-p1	14-Oct-11	1	Y	2	R	S	+	UK	SRR3335923
C00011960	L-142-40-p1	18-Nov-11	1	Y	2	R	S	+	UK	SRR3338067
C00011942	L-141-35-p1	29-Nov-11	1	Y	2	R	S	+	UK	SRR3335490
C00011958	L-142-38-p1	11-Dec-11	1	Y	2	R	S	+	UK	SRR3338731
C00011961	L-142-49-p1	20-Dec-11	1	Y	2	R	S	+	UK	SRR3335814
C00012134	L-L-12-7901549-p1	09-Jan-12	1	Y	2	R	S	+	UK	SRR3337722
C00012139	L-L-12-7901562-p1	06-Feb-12	1	Y	2	R	S	+	UK	SRR3338937
C00011745	L-L-12-7901580-p1	13-Mar-12	1	Y	2	R	S	+	UK	SRR3335515
C00012645	L-L-12-7901597-p1	15-Apr-12	1	Y	2	R	S	+	UK	SRR3334803
C00012179	L-149-54-p1	20-Apr-12	1	Y	2	R	S	+	UK	SRR3334896
C00012180	L-149-64-p1	25-Apr-12	1	Y	2	R	S	+	UK	SRR3337770
C00012190	L-151-32-p1	16-May-12	1	Y	2	R	S	+	UK	SRR3334818
C00025274	I00019274	07-Jul-12	1	Y	2	R	S	+	UK	SRR3337587
C00025194	I00019197	31-Jul-12	1	Y	2	R	S	+	UK	SRR3335520
C00025318	I00019317	29-Oct-12	1	Y	2	R	S	+	UK	SRR3335250
C00011890	L-111-6-p1	02-Aug-10	2	Y	1	S	S	+	UK	SRR3335541
C00012220	L-L-12-7901255-p1	04-Aug-10	2	Y	1	S	S	+	UK	SRR3338760
C00012222	L-L-12-7901258-p1	11-Aug-10	2	Y	1	S	S	+	UK	SRR3337611
C00012223	L-L-12-7901261-p1	16-Aug-10	2	Y	1	S	S	+	UK	SRR3334924
C00011149	L-151-11-p1	28-Aug-10	2	Y	1	S	S	+	UK	SRR3337595
C00010666	L-111-55-p1	14-Sep-10	2	Y	1	S	S	+	UK	SRR3334901
C00011886	L-155-35-p1	14-Sep-10	2	Y	1	S	S	+	UK	SRR3338997
C00011465	L-L-12-7901288-p1	16-Sep-10	2	Y	1	S	S	+	UK	SRR3338649
C00011154	L-151-16-p1	18-Sep-10	2	Y	1	S	S	+	UK	SRR3338472
C00010686	L-114-26-p1	21-Oct-10	2	Y	1	S	S	+	UK	SRR3338146
C00011478	L-L-12-7901316-p1	21-Oct-10	2	Y	1	S	S	+	UK	SRR3338601
C00010688	L-114-56-p1	31-Oct-10	2	Y	1	S	S	+	UK	SRR3334889
C00010693	L-115-01-p1	02-Nov-10	2	Y	1	S	S	+	UK	SRR3334913
C00010705	L-116-34-p1	05-Nov-10	2	Y	1	S	S	+	UK	SRR3337756
C00010701	L-116-16-p1	10-Nov-10	2	Y	1	S	S	+	UK	SRR3339312

C00010706	L-116-37-p1	23-Nov-10	2	Y	1	S	S	+	UK	SRR3337678
C00011807	L-8-92-p1	09-Dec-10	2	Y	1	S	S	+	UK	SRR3338903
C00010729	L-118-56-p1	16-Dec-10	2	Y	1	S	S	+	UK	SRR3335942
C00011816	L-9-17-p1	20-Dec-10	2	Y	1	S	S	+	UK	SRR3335943
C00011817	L-9-18-p1	20-Dec-10	2	Y	1	S	S	+	UK	SRR3337753
C00010725	L-118-30-p1	21-Dec-10	2	Y	1	S	S	+	UK	SRR3338626
C00010723	L-118-16-p1	31-Dec-10	2	Y	1	S	S	+	UK	SRR3338646
C00011821	L-9-62-p1	06-Jan-11	2	Y	1	S	S	+	UK	SRR3335597
C00012103	L-L-12-7901346-p1	06-Jan-11	2	Y	1	S	S	+	UK	SRR3334907
C00010730	L-118-64-p1	12-Jan-11	2	Y	1	S	S	+	UK	SRR3338710
C00010752	L-123-19-p1	08-Mar-11	2	Y	1	S	S	+	UK	SRR3335166
C00011864	L-11-74-p1	01-Apr-11	2	Y	1	S	S	+	UK	SRR3337681
C00011916	L-124-63-p1	02-Apr-11	2	Y	1	S	S	+	UK	SRR3338928
C00010579	L-124-77-p1	04-Apr-11	2	Y	1	S	S	+	UK	SRR3338531
C00010589	L-125-44-p1	15-Apr-11	2	Y	1	S	S	+	UK	SRR3334860
C00010596	L-126-14-p1	19-Apr-11	2	Y	1	S	S	+	UK	SRR3337736
C00011881	L-12-74-p1	14-May-11	2	Y	1	S	S	+	UK	SRR3338544
C00010609	L-127-36-p1	16-May-11	2	Y	1	S	S	+	UK	SRR3338075
C00010604	L-127-21-p1	19-May-11	2	Y	1	S	S	+	UK	SRR3338732
C00010608	L-127-35-p1	23-May-11	2	Y	1	S	S	+	UK	SRR3335908
C00011525	L-L-12-7901426-p1	13-Jun-11	2	Y	1	S	S	+	UK	SRR3334917
C00011087	L-129-31-p1	15-Jun-11	2	Y	1	S	S	+	UK	SRR3336001
C00012014	L-K6-1-p1	17-Jun-11	2	Y	1	S	S	+	UK	SRR3334881
C00012018	L-K6-17-p1	21-Jun-11	2	Y	1	S	S	+	UK	SRR3338641
C00011528	L-L-12-7901429-p1	22-Jun-11	2	Y	1	S	S	+	UK	SRR3338138
C00011529	L-L-12-7901431-p1	26-Jun-11	2	Y	1	S	S	+	UK	SRR3337774
C00012168	L-K6-27-p1	28-Jun-11	2	Y	1	S	S	+	UK	SRR3335539
C00011532	L-L-12-7901434-p1	30-Jun-11	2	Y	1	S	S	+	UK	SRR3339000
C00011102	L-130-60-p1	08-Jul-11	2	Y	1	S	S	+	UK	SRR3338652
C00012212	L-8-34-p1	08-Jul-11	2	Y	1	S	S	+	UK	SRR3339042
C00011101	L-130-59-p1	10-Jul-11	2	Y	1	S	S	+	UK	SRR3338639

C00012211	L-8-30-p1	12-Jul-11	2	Y	1	R	S	+	UK	SRR3338216
C00011105	L-131-52-p1	13-Jul-11	2	Y	1	S	S	+	UK	SRR3335289
C00011930	L-133-19-p1	11-Aug-11	2	Y	1	S	S	+	UK	SRR3339033
C00012115	L-L-12-7901456-p1	11-Aug-11	2	Y	1	S	S	+	UK	SRR3335932
C00011122	L-134-06-p1	24-Aug-11	2	Y	1	S	S	+	UK	SRR3339001
C00012216	L-8-38-p1	04-Sep-11	2	Y	1	S	S	+	UK	SRR3337646
C00011134	L-135-60-p1	05-Sep-11	2	Y	1	S	S	+	UK	SRR3338541
C00011126	L-134-81-p1	08-Sep-11	2	Y	1	S	S	+	UK	SRR3335145
C00011665	L-L-12-7901475-p1	12-Sep-11	2	Y	1	S	S	+	UK	SRR3335860
C00011666	L-L-12-7901476-p1	14-Sep-11	2	Y	1	S	S	+	UK	SRR3338210
C00011678	L-L-12-7901493-p1	14-Oct-11	2	Y	1	S	S	+	UK	SRR3338932
C00011941	L-141-34-p1	30-Nov-11	2	Y	1	S	R	+	UK	SRR3338586
C00011707	L-L-12-7901531-p1	09-Dec-11	2	Y	1	S	S	+	UK	SRR3338648
C00011714	L-L-12-7901540-p1	27-Dec-11	2	Y	1	S	S	+	UK	SRR3338164
C00011715	L-L-12-7901541-p1	27-Dec-11	2	Y	1	S	S	+	UK	SRR3336104
C00011732	L-L-12-7901565-p1	08-Feb-12	2	Y	1	S	S	+	UK	SRR3338240
C00011740	L-L-12-7901574-p1	01-Mar-12	2	Y	1	S	S	+	UK	SRR3334929
C00012141	L-L-12-7901575-p1	03-Mar-12	2	Y	1	S	S	+	UK	SRR3338744
C00011743	L-L-12-7901578-p1	06-Mar-12	2	Y	1	S	S	+	UK	SRR3338910
C00011744	L-L-12-7901579-p1	12-Mar-12	2	Y	1	S	S	+	UK	SRR3338520
C00012183	L-150-31-p1	24-Apr-12	2	Y	1	S	S	+	UK	SRR3335866
C00025144	I00019148	11-May-12	2	Y	1	S	S	+	UK	SRR3338069
C00025266	I00019267	11-May-12	2	Y	1	S	S	+	UK	SRR3338149
C00025190	I00019193	23-May-12	2	Y	1	S	S	+	UK	SRR3338494
C00012161	L-L-12-7901635-p1	24-May-12	2	Y	1	S	S	+	UK	SRR3338664
C00025198	I00019201	24-May-12	2	Y	1	S	S	+	UK	SRR3335918
C00025127	I00019131	26-May-12	2	Y	1	S	S	+	UK	SRR3337734
C00012164	L-L-12-7901638-p1	31-May-12	2	Y	1	S	S	+	UK	SRR3334814
C00025135	I00019139	31-May-12	2	Y	1	S	S	+	UK	SRR3338508
C00025207	I00019209	07-Jun-12	2	Y	1	S	S	+	UK	SRR3339047
C00025209	I00019211	18-Jun-12	2	Y	1	S	S	+	UK	SRR3335482

C00025257	I00019259	22-Jun-12	2	Y	1	S	S	+	UK	SRR3338466
C00025281	I00019281	27-Jun-12	2	Y	1	S	S	+	UK	SRR3334858
C00025282	I00019282	07-Jul-12	2	Y	1	S	S	+	UK	SRR3339389
C00025290	I00019290	07-Jul-12	2	Y	1	S	S	+	UK	SRR3339335
C00025298	I00019298	08-Jul-12	2	Y	1	S	S	+	UK	SRR3335837
C00025243	I00019245	14-Jul-12	2	Y	1	S	S	+	UK	SRR3338267
C00025299	I00019299	17-Jul-12	2	Y	1	S	S	+	UK	SRR3338929
C00025178	I00019181	30-Jul-12	2	Y	1	S	S	+	UK	SRR3335941
C00025284	I00019284	07-Aug-12	2	Y	1	S	S	+	UK	SRR3337710
C00025221	I00019223	12-Aug-12	2	Y	1	S	S	+	UK	SRR3335219
C00025229	I00019231	14-Aug-12	2	Y	1	S	S	+	UK	SRR3338227
C00025261	I00019262	24-Aug-12	2	Y	1	S	S	+	UK	SRR3335469
C00025368	I00019366	04-Oct-12	2	Y	1	S	S	+	UK	SRR3336077
C00025381	I00019379	22-Oct-12	2	Y	1	S	S	+	UK	SRR3337659
C00025345	I00019343	23-Oct-12	2	Y	1	S	S	+	UK	SRR3334845
C00025326	I00019324	29-Oct-12	2	Y	1	S	S	+	UK	SRR3334902
C00025335	I00019333	09-Nov-12	2	Y	1	S	S	+	UK	SRR3337522
C00025367	I00019365	14-Nov-12	2	Y	1	S	S	+	UK	SRR3335174
C00025432	I00019428	17-Nov-12	2	Y	1	S	S	+	UK	SRR3335980
C00025440	I00019436	18-Nov-12	2	Y	1	S	S	+	UK	SRR3335188
C00025449	I00019445	09-Dec-12	2	Y	1	S	S	+	UK	SRR3338936
C00025402	I00019400	22-Dec-12	2	Y	1	S	S	+	UK	SRR3337767
C00025426	I00019422	25-Dec-12	2	Y	1	S	S	+	UK	SRR3338493
C00025323	I00019321	31-Dec-12	2	Y	1	S	S	+	UK	SRR3335309
C00025482	I00019478	01-Jan-13	2	Y	1	S	S	+	UK	SRR3338620
C00025490	I00019485	03-Jan-13	2	Y	1	S	S	+	UK	SRR3339310
C00025801	I00019781	07-Jan-13	2	Y	1	S	S	+	UK	SRR3335500
C00025435	I00019431	14-Jan-13	2	Y	1	S	S	+	UK	SRR3334842
C00025363	I00019361	18-Jan-13	2	Y	1	S	S	+	UK	SRR3338761
C00025817	I00019797	18-Jan-13	2	Y	1	S	S	+	UK	SRR3335961
C00025420	I00019416	21-Jan-13	2	Y	1	S	S	+	UK	SRR3338955

C00025405	I00019403	18-Feb-13	2	Y	1	S	S	+	UK	SRR3338996
C00025861	I00019840	01-Mar-13	2	Y	1	S	S	+	UK	SRR3335471
C00025852	I00019831	06-Mar-13	2	Y	1	S	S	+	UK	SRR3335830
C00025868	I00019847	15-Mar-13	2	Y	1	S	S	+	UK	SRR3335873
C00025806	I00019786	17-Mar-13	2	Y	1	S	S	+	UK	SRR3338461
C00025847	I00019826	04-Apr-13	2	Y	1	S	S	+	UK	SRR3338549
C00025863	I00019842	05-Apr-13	2	Y	1	S	S	+	UK	SRR3335987
C00025125	I00019129	01-May-13	2	Y	1	S	S	+	UK	SRR3335304
C00011475	L-L-12-7901310-p1	11-Oct-10	3	Y	1	S	S	+	UK	SRR3335936
C00010699	L-115-62-p1	18-Oct-10	3	Y	1	S	S	+	UK	SRR3337720
C00011798	L-7-54-p1	18-Oct-10	3	Y	1	R	S	+	UK	SRR3334945
C00007971	L-114-67-p2	22-Oct-10	3	Y	1	S	S	+	UK	ERS352014
C00008523	L-115-03-p2	08-Nov-10	3	Y	1	S	S	+	UK	ERS352174
C00007758	L-115-59-p1	12-Nov-10	3	Y	1	S	S	+	UK	ERS352002
C00010704	L-116-32-p1	22-Nov-10	3	Y	1	S	S	+	UK	SRR3338107
C00011905	L-117-25-p1	06-Dec-10	3	Y	1	R	S	+	UK	SRR3338086
C00010715	L-117-24-p1	08-Dec-10	3	Y	1	S	S	+	UK	SRR3335990
C00010732	L-118-80-p1	19-Dec-10	3	Y	1	S	S	+	UK	SRR3338560
C00010719	L-117-48-p1	20-Dec-10	3	Y	1	S	S	+	UK	SRR3339313
C00012102	L-L-12-7901345-p1	04-Jan-11	3	Y	1	S	S	+	UK	SRR3338272
C00010742	L-120-12-p1	18-Jan-11	3	Y	1	R	S	+	UK	SRR3339278
C00011829	L-10-17-p1	28-Jan-11	3	Y	1	S	S	+	UK	SRR3335194
C00010746	L-121-53-p1	16-Feb-11	3	Y	1	S	S	+	UK	SRR3337713
C00010751	L-123-18-p1	28-Feb-11	3	Y	1	R	S	+	UK	SRR3339362
C00010567	L-123-56-p1	13-Mar-11	3	Y	1	S	S	+	UK	SRR3338618
C00010575	L-124-54-p1	02-Apr-11	3	Y	1	S	S	+	UK	SRR3339356
C00010586	L-125-20-p1	09-Apr-11	3	Y	1	S	S	+	UK	SRR3335185
C00010590	L-125-45-p1	15-Apr-11	3	Y	1	S	S	+	UK	SRR3335200
C00011530	L-L-12-7901432-p1	29-Jun-11	3	Y	1	S	S	+	UK	SRR3338746
C00011097	L-130-46-p1	03-Jul-11	3	Y	1	S	S	+	UK	SRR3335900

C00011560	L-L-12-7901465-p1	31-Aug-11	3	Y	1	S	S	+	UK	SRR3335230
C00011950	L-142-20-p1	21-Nov-11	3	Y	1	S	S	+	UK	SRR3335946
C00011972	L-143-51-p1	06-Jan-12	3	Y	1	S	S	+	UK	SRR3335605
C00011979	L-145-18-p1	29-Jan-12	3	Y	1	S	S	+	UK	SRR3339375
C00011746	L-L-12-7901581-p1	13-Mar-12	3	Y	1	S	S	+	UK	SRR3336079
C00012181	L-149-81-p1	22-Apr-12	3	Y	1	S	S	+	UK	SRR3338918
C00012657	L-L-12-7901610-p1	26-Apr-12	3	Y	1	S	S	+	UK	SRR3337515
C00012184	L-151-05-p1	18-May-12	3	Y	1	R	S	+	UK	SRR3335975
C00025134	I00019138	18-May-12	3	Y	1	R	S	+	UK	SRR3337711
C00025143	I00019147	02-Jun-12	3	Y	1	R	S	+	UK	SRR3338215
C00025232	I00019234	07-Jun-12	3	Y	1	S	S	+	UK	SRR3334914
C00025240	I00019242	08-Jun-12	3	Y	1	R	S	+	UK	SRR3336054
C00025265	I00019266	26-Jun-12	3	Y	1	R	S	+	UK	SRR3335852
C00025268	I00019269	06-Aug-12	3	Y	1	R	S	+	UK	SRR3336086
C00025237	I00019239	21-Aug-12	3	Y	1	R	S	+	UK	SRR3335834
C00025132	I00019136	19-Sep-12	3	Y	1	S	S	+	UK	SRR3338790
C00025336	I00019334	28-Sep-12	3	Y	1	R	S	+	UK	SRR3335984
C00025372	I00019370	10-Oct-12	3	Y	1	S	S	+	UK	SRR3336083
C00025325	I00019323	17-Oct-12	3	Y	1	R	S	+	UK	SRR3335503
C00025383	I00019381	14-Nov-12	3	Y	1	R	S	+	UK	SRR3334805
C00025418	I00019414	24-Dec-12	3	Y	1	S	S	+	UK	SRR3335581
C00025450	I00019446	28-Dec-12	3	Y	1	S	S	+	UK	SRR3335222
C00025403	I00019401	08-Jan-13	3	Y	1	S	S	+	UK	SRR3339381
C00025459	I00019455	19-Jan-13	3	Y	1	S	S	+	UK	SRR3338522
C00025460	I00019456	31-Jan-13	3	N	1	S	S	-	UK	SRR3338530
C00025845	I00019824	28-Feb-13	3	Y	1	S	S	+	UK	SRR3335432
C00025831	I00019810	03-Apr-13	3	Y	1	S	S	+	UK	SRR3335253
C00014379	L-L-12-7901252-p1	03-Aug-10	5	Y	3	S	S	+	UK	SRR3338587
C00010668	L-112-48-p1	22-Aug-10	5	Y	3	S	S	+	UK	SRR3338712
C00007686	L-112-20-p1	19-Sep-10	5	Y	3	S	S	+	UK	ERS351933
C00010667	L-111-66-p1	19-Sep-10	5	Y	3	S	S	+	UK	SRR3337583

C00007674	L-112-14-p1	23-Sep-10	5	Y	3	S	S	+	UK	ERS351923
C00010676	L-113-17-p1	10-Oct-10	5	Y	3	S	S	+	UK	SRR3334916
C00011486	L-L-12-7901334-p1	23-Nov-10	5	Y	3	S	S	+	UK	SRR3338891
C00010728	L-118-42-p1	05-Dec-10	5	Y	3	S	S	+	UK	SRR3334894
C00011491	L-L-12-7901344-p1	30-Dec-10	5	Y	3	S	S	+	UK	SRR3335480
C00011826	L-9-90-p1	18-Jan-11	5	Y	3	S	S	+	UK	SRR3338640
C00011861	L-11-68-p1	29-Mar-11	5	Y	3	S	S	+	UK	SRR3338206
C00011876	L-12-46-p1	30-Apr-11	5	Y	3	S	S	+	UK	SRR3335843
C00012009	L-K5-56-p1	01-Jun-11	5	Y	3	S	S	+	UK	SRR3334906
C00012214	L-8-40-p1	08-Jul-11	5	Y	3	S	S	+	UK	SRR3334830
C00011554	L-L-12-7901459-p1	19-Aug-11	5	Y	3	S	S	+	UK	SRR3336005
C00020357	L-L-12-7901511-p2	07-Nov-11	5	Y	3	S	S	+	UK	SRR3338759
C00011944	L-141-63-p1	21-Nov-11	5	Y	3	S	S	+	UK	SRR3338527
C00012131	L-L-12-7901539-p1	25-Dec-11	5	Y	3	S	S	+	UK	SRR3335187
C00011975	L-143-78-p1	17-Jan-12	5	Y	3	S	S	+	UK	SRR3339338
C00025159	I00019163	04-Jun-12	5	Y	3	S	S	+	UK	SRR3339363
C00025129	I00019133	15-Jun-12	5	Y	3	S	S	+	UK	SRR3338890
C00025219	I00019221	10-Jul-12	5	Y	3	S	S	+	UK	SRR3338576
C00025138	I00019142	16-Jul-12	5	Y	3	S	S	+	UK	SRR3335159
C00025154	I00019158	23-Jul-12	5	Y	3	S	S	+	UK	SRR3338621
C00025300	I00019300	10-Aug-12	5	Y	3	S	S	+	UK	SRR3337747
C00025356	I00019354	08-Oct-12	5	Y	3	S	S	+	UK	SRR3338585
C00025396	I00019394	13-Oct-12	5	Y	3	S	S	+	UK	SRR3334798
C00025342	I00019340	30-Oct-12	5	Y	3	S	S	+	UK	SRR3338259
C00025361	I00019359	30-Oct-12	5	Y	3	S	S	+	UK	SRR3337661
C00025351	I00019349	12-Nov-12	5	Y	3	S	S	+	UK	SRR3335257
C00025306	I00019305	19-Nov-12	5	Y	3	S	S	+	UK	SRR3334825
C00025393	I00019391	19-Nov-12	5	Y	3	S	S	+	UK	SRR3338923
C00025480	I00019476	27-Nov-12	5	Y	3	S	S	+	UK	SRR3339359
C00025322	I00019320	28-Nov-12	5	Y	3	S	S	+	UK	SRR3335893
C00025378	I00019376	10-Dec-12	5	Y	3	S	S	+	UK	SRR3337603

C00025474	I00019470	30-Dec-12	5	Y	3	S	S	+	UK	SRR3335917
C00025371	I00019369	24-Jan-13	5	Y	3	S	S	+	UK	SRR3339344
C00025387	I00019385	28-Jan-13	5	Y	3	S	S	+	UK	SRR3335954
C00025308	I00019307	04-Feb-13	5	Y	3	S	S	+	UK	SRR3338750
C00025324	I00019322	20-Feb-13	5	Y	3	S	S	+	UK	SRR3337607
C00025860	I00019839	07-Mar-13	5	Y	3	S	S	+	UK	SRR3335920
C00025846	I00019825	21-Mar-13	5	Y	3	S	S	+	UK	SRR3338542
C00025862	I00019841	23-Mar-13	5	Y	3	S	S	+	UK	SRR3337591
C00010675	L-112-77-p1	22-Aug-10	6	Y	1	S	S	+	UK	SRR3335855
C00010669	L-112-49-p1	27-Aug-10	6	Y	1	S	S	+	UK	SRR3339366
C00011883	L-155-33-p1	06-Sep-10	6	Y	1	S	S	+	UK	SRR3338080
C00011155	L-151-17-p1	19-Sep-10	6	Y	1	S	S	+	UK	SRR3335828
C00010670	L-112-56-p1	25-Sep-10	6	Y	1	S	S	+	UK	SRR3335868
C00011920	L-124-75-p1	19-Oct-10	6	Y	1	S	S	+	UK	SRR3338173
C00011904	L-116-27-p1	04-Nov-10	6	Y	1	S	S	+	UK	SRR3338172
C00010697	L-115-43-p1	07-Nov-10	6	Y	1	S	S	+	UK	SRR3335519
C00010717	L-117-42-p1	02-Dec-10	6	Y	1	S	S	+	UK	SRR3335818
C00011912	L-123-50-p1	31-Dec-10	6	Y	1	S	S	+	UK	SRR3338762
C00010738	L-120-8-p1	18-Jan-11	6	Y	1	S	S	+	UK	SRR3335547
C00010743	L-120-20-p1	27-Jan-11	6	Y	1	S	S	+	UK	SRR3334810
C00010564	L-123-53-p1	15-Mar-11	6	Y	1	S	S	+	UK	SRR3338757
C00011116	L-133-60-p1	06-Jul-11	6	Y	1	S	S	+	UK	SRR3335952
C00011540	L-L-12-7901443-p1	20-Jul-11	6	Y	1	S	S	+	UK	SRR3338575
C00012199	L-7-40-p1	19-Aug-11	6	Y	1	S	S	+	UK	SRR3335489
C00011113	L-133-36-p1	23-Aug-11	6	Y	1	S	S	+	UK	SRR3338502
C00011119	L-133-80-p1	24-Aug-11	6	Y	1	S	S	+	UK	SRR3337615
C00011127	L-135-01-p1	09-Sep-11	6	Y	1	S	S	+	UK	SRR3335239
C00011136	L-136-39-p1	11-Sep-11	6	Y	1	S	S	+	UK	SRR3339392
C00011968	L-143-16-p1	30-Dec-11	6	Y	1	S	S	+	UK	SRR3334938
C00011727	L-L-12-7901559-p1	27-Jan-12	6	Y	1	S	S	+	UK	SRR3336075

C00011729	L-L-12-7901561-p1	04-Feb-12	6	Y	1	S	S	+	UK	SRR3337721
C00012038	L-147-48-p1	06-Mar-12	6	Y	1	S	S	+	UK	SRR3339350
C00012177	L-149-06-p1	01-Apr-12	6	Y	1	S	S	+	UK	SRR3338584
C00025160	I00019164	15-May-12	6	Y	1	S	S	+	UK	SRR3335543
C00025126	I00019130	18-May-12	6	Y	1	S	S	+	UK	SRR3335835
C00012155	L-L-12-7901629-p1	19-May-12	6	Y	1	S	S	+	UK	SRR3338976
C00025150	I00019154	19-May-12	6	Y	1	S	S	+	UK	SRR3335979
C00025200	I00019195	07-Jun-12	6	Y	1	S	S	+	UK	SRR3335822
C00025121	I00019125	14-Jun-12	6	Y	1	S	S	+	UK	SRR3338376
C00025153	I00019157	22-Jun-12	6	Y	1	S	S	+	UK	SRR3338982
C00025161	I00019165	25-Jun-12	6	Y	1	S	S	+	UK	SRR3335512
C00025297	I00019297	27-Jun-12	6	Y	1	S	S	+	UK	SRR3338953
C00025234	I00019236	03-Jul-12	6	Y	1	S	S	+	UK	SRR3337608
C00025201	I00019203	05-Jul-12	6	Y	1	S	S	+	UK	SRR3338577
C00025235	I00019237	13-Jul-12	6	Y	1	S	S	+	UK	SRR3335271
C00025260	I00019261	04-Aug-12	6	Y	1	S	S	+	UK	SRR3338152
C00025123	I00019127	06-Aug-12	6	Y	1	S	S	+	UK	SRR3338492
C00025139	I00019143	09-Aug-12	6	Y	1	S	S	+	UK	SRR3335314
C00025292	I00019292	10-Aug-12	6	Y	1	S	S	+	UK	SRR3338468
C00025301	I00019301	04-Sep-12	6	Y	1	S	S	+	UK	SRR3334915
C00025254	I00019256	10-Sep-12	6	Y	1	S	S	+	UK	SRR3337648
C00025287	I00019287	27-Sep-12	6	Y	1	S	S	+	UK	SRR3335927
C00025312	I00019311	28-Sep-12	6	Y	1	S	S	+	UK	SRR3337751
C00025204	I00019206	05-Oct-12	6	Y	1	S	S	+	UK	SRR3338155
C00025365	I00019363	22-Oct-12	6	Y	1	S	S	+	UK	SRR3338578
C00025398	I00019396	06-Nov-12	6	Y	1	S	S	+	UK	SRR3339303
C00025319	I00019318	07-Nov-12	6	Y	1	S	S	+	UK	SRR3338905
C00025327	I00019325	07-Nov-12	6	Y	1	S	S	+	UK	SRR3338229
C00025391	I00019389	14-Nov-12	6	Y	1	S	S	+	UK	SRR3336071
C00025456	I00019452	20-Nov-12	6	Y	1	S	S	+	UK	SRR3338952
C00025401	I00019399	30-Nov-12	6	Y	1	S	S	+	UK	SRR3337579

C00025362	I00019360	03-Dec-12	6	Y	1	S	S	+	UK	SRR3338769
C00025370	I00019368	03-Dec-12	6	Y	1	S	S	+	UK	SRR3335513
C00025466	I00019462	29-Dec-12	6	Y	1	S	S	+	UK	SRR3338962
C00025428	I00019424	21-Jan-13	6	Y	1	S	S	+	UK	SRR3338496
C00025838	I00019817	21-Mar-13	6	Y	1	S	S	+	UK	SRR3338085
C00010660	L-110-19-p1	18-Aug-10	7	Y	1	S	S	+	UK	ERS243701
C00011884	L-155-34-p1	10-Sep-10	7	Y	1	S	S	+	UK	SRR3338924
C00011472	L-L-12-7901304-p1	30-Sep-10	7	Y	1	S	S	+	UK	SRR3338988
C00010678	L-113-20-p1	06-Oct-10	7	Y	1	S	S	+	UK	ERS243702
C00011479	L-L-12-7901322-p1	28-Oct-10	7	Y	1	S	S	+	UK	ERS243706
C00010711	L-116-81-p1	02-Dec-10	7	Y	1	S	S	+	UK	ERS243703
C00011507	L-L-12-7901387-p1	05-Apr-11	7	Y	1	S	S	-	UK	ERS243707
C00011939	L-140-65-p1	20-Nov-11	7	Y	1	S	S	+	UK	ERS243704
C00011951	L-142-21-p1	21-Nov-11	7	Y	1	S	S	+	UK	ERS243705
C00011945	L-141-64-p1	24-Nov-11	7	Y	1	S	S	+	UK	SRR3338684
C00012032	L-146-54-p1	27-Dec-11	7	Y	1	S	S	+	UK	SRR3338251
C00025364	I00019362	08-Oct-12	7	Y	1	S	S	-	UK	SRR3338197
C00025313	I00019312	11-Oct-12	7	Y	1	S	S	+	UK	SRR3335569
C00025366	I00019364	03-Nov-12	7	Y	1	S	S	+	UK	SRR3335252
C00025465	I00019461	13-Dec-12	7	Y	1	S	S	+	UK	SRR3338102
C00025339	I00019337	07-Jan-13	7	Y	1	S	S	-	UK	SRR3335504
C00025419	I00019415	09-Jan-13	7	Y	1	S	S	+	UK	SRR3338178
C00025469	I00019465	25-Feb-13	7	Y	1	S	S	-	UK	SRR3335465
C00025822	I00019802	19-Mar-13	7	Y	1	S	S	+	UK	SRR3338666
C00025878	I00019857	27-Mar-13	7	Y	1	S	S	+	UK	SRR3335591
C00025886	I00019865	27-Mar-13	7	Y	1	S	S	+	UK	SRR3338136
C00025799	I00019779	30-Mar-13	7	Y	1	S	S	+	UK	SRR3335950
C00011891	L-111-67-p1	06-Aug-10	8	Y	1	S	S	+	UK	SRR3337672
C00011887	L-109-06-p1	07-Aug-10	8	Y	1	S	S	+	UK	SRR3335216
C00010662	L-110-39-p1	17-Aug-10	8	Y	1	S	S	+	UK	SRR3335470

C00012225	L-L-12-7901264-p1	23-Aug-10	8	Y	1	S	S	+	UK	SRR3335914
C00012228	L-L-12-7901268-p1	26-Aug-10	8	Y	1	S	S	+	UK	SRR3338111
C00010681	L-113-70-p1	18-Sep-10	8	Y	1	S	S	+	UK	SRR3338778
C00011470	L-L-12-7901299-p1	23-Sep-10	8	Y	1	S	S	+	UK	SRR3339309
C00011157	L-151-19-p1	24-Sep-10	8	Y	1	S	S	+	UK	SRR3339022
C00010722	L-118-15-p1	18-Oct-10	8	Y	1	S	S	+	UK	SRR3335550
C00011801	L-7-69-p1	23-Oct-10	8	Y	1	S	S	+	UK	SRR3335894
C00011802	L-7-75-p1	24-Oct-10	8	Y	1	S	S	+	UK	SRR3338922
C00010691	L-114-66-p1	25-Oct-10	8	Y	1	S	S	+	UK	SRR3336057
C00011984	L-K1-34-p1	08-Nov-10	8	Y	1	S	S	+	UK	SRR3337735
C00010721	L-117-62-p1	16-Dec-10	8	Y	1	S	S	+	UK	SRR3335478
C00010726	L-118-34-p1	03-Jan-11	8	Y	1	S	S	+	UK	SRR3337795
C00010733	L-119-01-p1	09-Jan-11	8	Y	1	S	S	+	UK	SRR3335530
C00011909	L-119-66-p1	20-Jan-11	8	Y	1	S	S	+	UK	SRR3338254
C00010740	L-120-10-p1	21-Jan-11	8	Y	1	S	S	+	UK	SRR3334811
C00010739	L-120-9-p1	24-Jan-11	8	Y	1	S	S	+	UK	SRR3337509
C00010747	L-121-65-p1	03-Feb-11	8	Y	1	S	S	+	UK	SRR3335474
C00011842	L-10-62-p1	11-Feb-11	8	Y	1	S	S	+	UK	SRR3338748
C00011083	L-128-22-p1	17-Feb-11	8	Y	1	S	S	+	UK	SRR3334971
C00010561	L-123-22-p1	25-Feb-11	8	Y	1	S	S	+	UK	SRR3335935
C00010581	L-124-79-p1	18-Mar-11	8	Y	1	S	S	+	UK	SRR3337745
C00010574	L-124-17-p1	21-Mar-11	8	Y	1	S	S	+	UK	SRR3339379
C00010577	L-124-64-p1	04-Apr-11	8	Y	1	S	S	+	UK	SRR3337507
C00010578	L-124-76-p1	06-Apr-11	8	Y	1	S	S	+	UK	SRR3337506
C00010587	L-125-36-p1	14-Apr-11	8	Y	1	S	S	+	UK	SRR3336085
C00011954	L-142-24-p1	12-May-11	8	Y	1	S	S	+	UK	SRR3337670
C00011088	L-129-37-p1	17-Jun-11	8	Y	1	S	S	+	UK	SRR3337614
C00011094	L-130-25-p1	23-Jun-11	8	Y	1	S	S	+	UK	SRR3338270
C00011099	L-130-52-p1	02-Jul-11	8	Y	1	S	S	+	UK	SRR3337797
C00012169	L-K6-45-p1	04-Jul-11	8	Y	1	S	S	+	UK	SRR3337708
C00012021	L-K6-47-p1	08-Jul-11	8	Y	1	S	S	+	UK	SRR3335313

C00012024	L-K6-63-p1	19-Jul-11	8	Y	1	S	S	+	UK	SRR3338249
C00011108	L-132-61-p1	07-Aug-11	8	Y	1	S	S	+	UK	SRR3339003
C00011137	L-136-40-p1	10-Aug-11	8	Y	1	S	S	+	UK	SRR3334875
C00011555	L-L-12-7901460-p1	23-Aug-11	8	Y	1	S	S	+	UK	SRR3338958
C00011115	L-133-38-p1	24-Aug-11	8	Y	1	S	S	+	UK	SRR3335609
C00011657	L-L-12-7901466-p1	01-Sep-11	8	Y	1	S	S	+	UK	SRR3335177
C00011663	L-L-12-7901473-p1	07-Sep-11	8	Y	1	S	S	+	UK	SRR3339297
C00011684	L-L-12-7901498-p1	16-Oct-11	8	Y	1	S	S	+	UK	SRR3338900
C00011687	L-L-12-7901501-p1	21-Oct-11	8	Y	1	S	S	+	UK	SRR3337709
C00011691	L-L-12-7901507-p1	01-Nov-11	8	Y	1	S	S	+	UK	SRR3338715
C00012124	L-L-12-7901513-p1	12-Nov-11	8	Y	1	S	S	+	UK	SRR3335293
C00011949	L-142-19-p1	14-Nov-11	8	Y	1	S	S	+	UK	SRR3335315
C00011946	L-141-65-p1	18-Nov-11	8	Y	1	S	S	+	UK	SRR3338659
C00011723	L-L-12-7901553-p1	14-Jan-12	8	Y	1	S	S	+	UK	SRR3336038
C00012138	L-L-12-7901556-p1	24-Jan-12	8	Y	1	S	S	+	UK	SRR3335446
C00012026	L-145-68-p1	15-Feb-12	8	Y	1	S	S	+	UK	SRR3337602
C00012140	L-L-12-7901568-p1	18-Feb-12	8	Y	1	S	S	+	UK	SRR3338175
C00012028	L-146-41-p1	24-Feb-12	8	Y	1	S	S	+	UK	SRR3338674
C00011739	L-L-12-7901573-p1	28-Feb-12	8	Y	1	S	S	+	UK	SRR3339396
C00012035	L-146-68-p1	01-Mar-12	8	Y	1	S	S	+	UK	SRR3335521
C00012145	L-L-12-7901604-p1	22-Apr-12	8	Y	1	S	S	+	UK	SRR3334856
C00012654	L-L-12-7901606-p1	23-Apr-12	8	Y	1	S	S	+	UK	SRR3338911
C00012147	L-L-12-7901619-p1	11-May-12	8	Y	1	S	S	+	UK	SRR3334846
C00025136	I00019140	11-May-12	8	Y	1	S	S	+	UK	SRR3339008
C00025165	I00019169	13-May-12	8	Y	1	S	S	+	UK	SRR3335282
C00025173	I00019177	14-May-12	8	Y	1	S	S	+	UK	SRR3338268
C00025197	I00019200	15-May-12	8	Y	1	S	S	+	UK	SRR3336078
C00012189	L-151-31-p1	21-May-12	8	Y	1	S	S	+	UK	SRR3335881
C00025158	I00019162	21-May-12	8	Y	1	S	S	+	UK	SRR3338981
C00025175	I00019179	05-Jun-12	8	Y	1	S	S	+	UK	SRR3335846
C00025288	I00019288	15-Jun-12	8	Y	1	S	S	+	UK	SRR3336103

C00025225	I00019227	21-Jun-12	8	Y	1	S	S	+	UK	SRR3339347
C00025122	I00019126	10-Jul-12	8	Y	1	S	S	+	UK	SRR3334819
C00025291	I00019291	17-Jul-12	8	Y	1	S	S	+	UK	SRR3335493
C00025220	I00019222	29-Jul-12	8	Y	1	S	S	+	UK	SRR3338204
C00025236	I00019238	01-Aug-12	8	Y	1	S	S	+	UK	SRR3338495
C00025155	I00019159	13-Aug-12	8	Y	1	S	S	+	UK	SRR3337688
C00025245	I00019247	21-Aug-12	8	Y	1	S	S	+	UK	SRR3338556
C00025179	I00019182	23-Aug-12	8	Y	1	S	S	+	UK	SRR3335988
C00025277	I00019277	27-Aug-12	8	Y	1	S	S	+	UK	SRR3338688
C00025222	I00019224	06-Sep-12	8	Y	1	S	S	+	UK	SRR3338767
C00025215	I00019217	18-Sep-12	8	Y	1	S	S	+	UK	SRR3338473
C00025223	I00019225	19-Sep-12	8	Y	1	S	S	+	UK	SRR3338271
C00025231	I00019233	20-Sep-12	8	Y	1	S	S	+	UK	SRR3334855
C00025239	I00019241	21-Sep-12	8	Y	1	S	S	+	UK	SRR3335542
C00025156	I00019160	26-Sep-12	8	Y	1	S	S	+	UK	SRR3337793
C00025388	I00019386	11-Oct-12	8	Y	1	S	S	+	UK	SRR3335841
C00025329	I00019327	21-Oct-12	8	Y	1	S	S	+	UK	SRR3334905
C00025357	I00019355	22-Oct-12	8	Y	1	S	S	+	UK	SRR3337589
C00025369	I00019367	01-Nov-12	8	Y	1	S	S	+	UK	SRR3338228
C00025390	I00019388	05-Nov-12	8	Y	1	S	S	+	UK	SRR3339322
C00025448	I00019444	19-Nov-12	8	Y	1	S	S	+	UK	SRR3335933
C00025433	I00019429	07-Dec-12	8	Y	1	S	S	+	UK	SRR3335862
C00025315	I00019314	19-Dec-12	8	Y	1	S	S	+	UK	SRR3337511
C00025434	I00019430	25-Dec-12	8	Y	1	S	S	+	UK	SRR3338489
C00025411	I00019408	09-Jan-13	8	Y	1	S	S	+	UK	SRR3335553
C00025451	I00019447	16-Jan-13	8	Y	1	S	S	+	UK	SRR3338921
C00025379	I00019377	25-Jan-13	8	Y	1	S	S	+	UK	SRR3337520
C00025837	I00019816	27-Feb-13	8	Y	1	S	S	+	UK	SRR3338555
C00025794	I00019774	28-Feb-13	8	Y	1	S	S	+	UK	SRR3338725
C00025869	I00019848	06-Mar-13	8	Y	1	S	S	+	UK	SRR3339354

C00012218	L-L-12-7901251-p1	02-Aug-10	9	Y	1	S	S	+	UK	SRR3335495
C00010680	L-113-28-p1	28-Sep-10	9	Y	1	S	S	+	UK	SRR3338134
C00011795	L-7-47-p1	12-Oct-10	9	Y	1	S	S	+	UK	SRR3338245
C00010703	L-116-30-p1	24-Nov-10	9	Y	1	S	S	+	UK	SRR3335256
C00011989	L-K2-48-p1	20-Dec-10	9	Y	1	S	S	+	UK	SRR3338073
C00010741	L-120-11-p1	20-Jan-11	9	Y	1	S	S	+	UK	SRR3338501
C00010565	L-123-54-p1	11-Mar-11	9	Y	1	S	S	+	UK	SRR3338261
C00011559	L-L-12-7901464-p1	27-Aug-11	9	Y	1	S	S	+	UK	SRR3339349
C00012208	L-8-14-p1	21-Sep-11	9	Y	1	S	S	+	UK	SRR3338214
C00011718	L-L-12-7901544-p1	01-Jan-12	9	Y	1	S	S	+	UK	SRR3334892
C00011724	L-L-12-7901555-p1	20-Jan-12	9	Y	1	S	S	+	UK	SRR3338683
C00025294	I00019294	15-Sep-12	9	Y	1	S	S	+	UK	SRR3335264
C00025309	I00019308	15-Oct-12	9	Y	1	S	S	+	UK	SRR3338737
C00025484	I00019480	09-Feb-13	9	Y	1	S	S	+	UK	SRR3338792
C00007722	L-112-38-p2	24-Sep-10	10	Y	1	S	S	+	UK	ERS351969
C00011914	L-124-31-p1	21-Jan-11	10	Y	1	S	S	+	UK	SRR3339386
C00011855	L-11-18-p1	08-Mar-11	10	Y	1	S	S	+	UK	SRR3338896
C00010598	L-126-16-p1	25-Mar-11	10	Y	1	S	S	+	UK	SRR3335456
C00010607	L-127-34-p1	31-Mar-11	10	Y	1	S	S	+	UK	SRR3338491
C00011140	L-137-34-p1	15-Apr-11	10	Y	1	S	S	+	UK	SRR3338772
C00012010	L-K5-67-p1	02-Jun-11	10	Y	1	S	S	+	UK	SRR3338954
C00011120	L-134-04-p1	13-Aug-11	10	Y	1	S	S	+	UK	SRR3335944
C00011141	L-138-49-p1	12-Oct-11	10	Y	1	S	S	+	UK	SRR3337772
C00011690	L-L-12-7901505-p1	26-Oct-11	10	Y	1	S	S	+	UK	SRR3338088
C00011692	L-L-12-7901508-p1	01-Nov-11	10	Y	1	S	S	+	UK	SRR3338209
C00011694	L-L-12-7901510-p1	03-Nov-11	10	Y	1	S	S	+	UK	SRR3337763
C00011959	L-142-39-p1	01-Dec-11	10	Y	1	S	S	+	UK	SRR3335287
C00011973	L-143-52-p1	20-Dec-11	10	Y	1	S	S	+	UK	SRR3337725
C00011977	L-144-67-p1	28-Jan-12	10	Y	1	S	S	+	UK	SRR3335285
C00025264	I00019265	13-Jun-12	10	Y	1	S	S	+	UK	SRR3338564

C00025210	I00019212	28-Jun-12	10	Y	1	S	S	+	UK	SRR3335566
C00025228	I00019230	30-Jul-12	10	Y	1	S	S	+	UK	SRR3334829
C00025213	I00019215	10-Aug-12	10	Y	1	S	S	+	UK	SRR3335548
C00025195	I00019198	27-Aug-12	10	Y	1	S	S	+	UK	SRR3338115
C00025116	I00019120	04-Sep-12	10	Y	1	S	S	+	UK	SRR3335579
C00025148	I00019152	24-Sep-12	10	Y	1	S	S	+	UK	SRR3338721
C00025279	I00019279	26-Sep-12	10	Y	1	S	S	+	UK	SRR3336072
C00025295	I00019295	27-Sep-12	10	Y	1	S	S	+	UK	SRR3338786
C00025328	I00019326	28-Sep-12	10	Y	1	S	S	+	UK	SRR3335995
C00025389	I00019387	23-Oct-12	10	Y	1	S	S	+	UK	SRR3339370
C00025424	I00019420	15-Nov-12	10	Y	1	S	S	+	UK	SRR3334877
C00025452	I00019448	29-Jan-13	10	Y	1	S	S	+	UK	SRR3335969
C00025854	I00019833	23-Mar-13	10	Y	1	S	S	+	UK	SRR3335858
C00025870	I00019849	24-Mar-13	10	Y	1	S	S	+	UK	SRR3337669
C00011889	L-110-03-p1	02-Aug-10	11	Y	5	S	S	+	UK	SRR3338089
C00012221	L-L-12-7901257-p1	11-Aug-10	11	Y	5	S	S	+	UK	SRR3338148
C00011147	L-151-09-p1	12-Aug-10	11	Y	5	S	S	+	UK	SRR3338459
C00010659	L-110-18-p1	13-Aug-10	11	Y	5	R	S	+	UK	SRR3335028
C00012224	L-L-12-7901263-p1	17-Aug-10	11	Y	5	S	S	+	UK	SRR3335466
C00010671	L-112-57-p1	02-Oct-10	11	Y	5	S	S	+	UK	SRR3334844
C00011476	L-L-12-7901311-p1	11-Oct-10	11	Y	5	S	S	+	UK	SRR3337598
C00011797	L-7-51-p1	16-Oct-10	11	Y	5	S	S	+	UK	SRR3335254
C00010696	L-115-17-p1	08-Nov-10	11	Y	5	S	S	+	UK	SRR3339336
C00011482	L-L-12-7901328-p1	11-Nov-10	11	Y	5	S	S	+	UK	SRR3338656
C00011804	L-8-50-p1	21-Nov-10	11	Y	5	S	S	+	UK	SRR3335558
C00011987	L-K1-74-p1	22-Nov-10	11	Y	5	S	S	+	UK	SRR3334921
C00010708	L-116-39-p1	26-Nov-10	11	Y	5	S	S	+	UK	SRR3337613
C00011806	L-8-82-p1	06-Dec-10	11	Y	5	S	S	+	UK	SRR3338594
C00011815	L-9-12-p1	17-Dec-10	11	Y	5	S	S	+	UK	SRR3337768
C00010731	L-118-65-p1	04-Jan-11	11	Y	5	S	S	+	UK	SRR3335606

C00011823	L-9-69-p1	07-Jan-11	11	Y	5	S	S	+	UK	SRR3338177
C00012105	L-L-12-7901361-p1	31-Jan-11	11	Y	5	S	S	+	UK	SRR3338160
C00011910	L-122-03-p1	04-Feb-11	11	Y	5	S	S	+	UK	SRR3338689
C00010750	L-122-52-p1	12-Feb-11	11	Y	5	R	S	+	UK	SRR3335982
C00011847	L-10-77-p1	20-Feb-11	11	Y	5	R	S	+	UK	SRR3338187
C00011849	L-10-88-p1	24-Feb-11	11	Y	5	S	S	+	UK	SRR3335524
C00010570	L-123-76-p1	05-Mar-11	11	Y	5	S	S	+	UK	SRR3339360
C00010563	L-123-52-p1	11-Mar-11	11	Y	5	S	S	+	UK	SRR3339299
C00010568	L-123-59-p1	12-Mar-11	11	Y	5	S	S	+	UK	SRR3338770
C00010566	L-123-55-p1	15-Mar-11	11	Y	5	S	S	+	UK	SRR3337723
C00012193	L-4-47-p1	15-Mar-11	11	Y	5	S	S	+	UK	SRR3338230
C00010562	L-123-51-p1	16-Mar-11	11	Y	5	S	S	+	UK	SRR3337679
C00011867	L-11-79-p1	01-Apr-11	11	Y	5	S	S	+	UK	SRR3336003
C00010610	L-127-37-p1	13-Apr-11	11	Y	5	S	S	+	UK	SRR3334865
C00011873	L-12-13-p1	15-Apr-11	11	Y	5	S	S	+	UK	SRR3335165
C00011509	L-L-12-7901392-p1	18-Apr-11	11	Y	5	S	S	+	UK	SRR3335611
C00010594	L-126-3-p1	26-Apr-11	11	Y	5	S	S	+	UK	SRR3335204
C00011879	L-12-67-p1	11-May-11	11	Y	5	S	S	+	UK	SRR3337601
C00011086	L-128-29-p1	24-May-11	11	Y	5	S	S	+	UK	SRR3335235
C00011514	L-L-12-7901414-p1	24-May-11	11	Y	5	R	S	+	UK	SRR3338513
C00012008	L-K5-51-p1	29-May-11	11	Y	5	S	S	+	UK	SRR3335150
C00011095	L-130-26-p1	05-Jun-11	11	Y	5	R	S	+	UK	SRR3338464
C00011522	L-L-12-7901422-p1	08-Jun-11	11	Y	5	S	S	+	UK	SRR3335912
C00012167	L-K6-22-p1	25-Jun-11	11	Y	5	S	S	+	UK	SRR3335458
C00012019	L-K6-29-p1	27-Jun-11	11	Y	5	R	S	+	UK	SRR3335487
C00011982	L-K6-71-p1	22-Jul-11	11	Y	5	R	S	+	UK	SRR3335792
C00012114	L-L-12-7901448-p1	27-Jul-11	11	Y	5	S	S	+	UK	SRR3335878
C00011109	L-132-65-p1	03-Aug-11	11	Y	5	S	S	+	UK	SRR3338950
C00011933	L-134-45-p1	09-Aug-11	11	Y	5	S	S	+	UK	SRR3335199
C00012196	L-7-34-p1	18-Aug-11	11	Y	5	R	S	+	UK	SRR3338498
C00011131	L-135-46-p1	09-Sep-11	11	Y	5	S	S	+	UK	SRR3338145

C00011669	L-L-12-7901481-p1	28-Sep-11	11	Y	5	S	S	+	UK	SRR3338930
C00011953	L-142-23-p1	05-Oct-11	11	Y	5	S	S	+	UK	SRR3335887
C00012119	L-L-12-7901489-p1	09-Oct-11	11	Y	5	S	S	+	UK	SRR3335864
C00011682	L-L-12-7901496-p1	14-Oct-11	11	Y	5	S	S	+	UK	SRR3338632
C00011689	L-L-12-7901504-p1	21-Oct-11	11	Y	5	S	S	+	UK	SRR3338217
C00011937	L-140-32-p1	13-Nov-11	11	Y	5	S	S	+	UK	SRR3335510
C00011697	L-L-12-7901515-p1	14-Nov-11	11	Y	5	S	S	+	UK	SRR3336061
C00011943	L-141-36-p1	29-Nov-11	11	Y	5	S	S	+	UK	SRR3338203
C00011705	L-L-12-7901527-p1	03-Dec-11	11	Y	5	S	S	+	UK	SRR3338487
C00011947	L-141-66-p1	04-Dec-11	11	Y	5	S	S	+	UK	SRR3335163
C00012129	L-L-12-7901529-p1	08-Dec-11	11	Y	5	S	S	+	UK	SRR3337585
C00011967	L-142-72-p1	16-Dec-11	11	Y	5	S	S	+	UK	SRR3335890
C00011966	L-142-64-p1	23-Dec-11	11	Y	5	R	S	+	UK	SRR3338211
C00011965	L-142-63-p1	25-Dec-11	11	Y	5	S	S	+	UK	SRR3338679
C00011974	L-143-53-p1	15-Jan-12	11	Y	5	R	S	+	UK	SRR3338966
C00011730	L-L-12-7901563-p1	06-Feb-12	11	Y	5	S	S	+	UK	SRR3339374
C00012030	L-146-52-p1	29-Feb-12	11	Y	5	S	S	+	UK	SRR3338692
C00012036	L-146-69-p1	02-Mar-12	11	Y	5	S	S	+	UK	SRR3338105
C00012641	L-L-12-7901591-p1	01-Apr-12	11	Y	5	S	S	+	UK	SRR3338253
C00012143	L-L-12-7901592-p1	02-Apr-12	11	Y	5	S	S	+	UK	SRR3339357
C00012642	L-L-12-7901593-p1	11-Apr-12	11	Y	5	S	S	+	UK	SRR3337783
C00012176	L-148-76-p1	12-Apr-12	11	Y	5	S	S	+	UK	SRR3338104
C00012144	L-L-12-7901595-p1	14-Apr-12	11	Y	5	S	S	+	UK	SRR3338113
C00012146	L-L-12-7901616-p1	08-May-12	11	Y	5	S	S	+	UK	SRR3337516
C00025128	I00019132	08-May-12	11	Y	5	S	S	+	UK	SRR3335947
C00025217	I00019219	20-Jun-12	11	Y	5	S	S	+	UK	SRR3338665
C00025241	I00019243	22-Jun-12	11	Y	5	R	S	+	UK	SRR3337588
C00025169	I00019173	28-Jun-12	11	Y	5	R	S	+	UK	SRR3335274
C00025185	I00019188	29-Jun-12	11	Y	5	S	S	+	UK	SRR3335992
C00025242	I00019244	05-Jul-12	11	Y	5	S	S	+	UK	SRR3335836
C00025211	I00019213	09-Jul-12	11	Y	5	S	S	+	UK	SRR3335577

C00025130	I00019134	12-Jul-12	11	Y	5	R	S	+	UK	SRR3338186
C00025267	I00019268	15-Jul-12	11	Y	5	S	S	+	UK	SRR3336070
C00025146	I00019150	22-Jul-12	11	Y	5	S	S	+	UK	SRR3338066
C00025170	I00019174	27-Jul-12	11	Y	5	R	S	+	UK	SRR3338994
C00025230	I00019232	07-Sep-12	11	Y	5	S	S	+	UK	SRR3335119
C00025238	I00019240	07-Sep-12	11	Y	5	R	S	+	UK	SRR3335305
C00025262	I00019263	11-Sep-12	11	Y	5	S	S	+	UK	SRR3335214
C00025164	I00019168	27-Sep-12	11	Y	5	S	S	+	UK	SRR3335178
C00025188	I00019191	04-Oct-12	11	Y	5	S	S	+	UK	SRR3335476
C00025360	I00019358	04-Oct-12	11	Y	5	S	S	+	UK	SRR3339388
C00025340	I00019338	07-Oct-12	11	Y	5	S	S	+	UK	SRR3338566
C00025373	I00019371	22-Oct-12	11	Y	5	S	S	+	UK	SRR3335861
C00025358	I00019356	01-Nov-12	11	Y	5	S	S	+	UK	SRR3338623
C00025311	I00019310	06-Nov-12	11	Y	5	S	S	+	UK	SRR3334928
C00025399	I00019397	14-Nov-12	11	Y	5	S	S	+	UK	SRR3335217
C00025409	I00019406	01-Dec-12	11	Y	5	S	S	+	UK	SRR3339365
C00025417	I00019413	03-Dec-12	11	Y	5	S	S	+	UK	SRR3338147
C00025441	I00019437	07-Dec-12	11	Y	5	S	S	+	UK	SRR3339030
C00025475	I00019471	20-Jan-13	11	Y	5	S	S	+	UK	SRR3339385
C00025491	I00019486	20-Jan-13	11	Y	5	S	S	+	UK	SRR3338247
C00025316	I00019315	06-Feb-13	11	Y	5	S	S	+	UK	SRR3335534
C00025492	I00019487	14-Feb-13	11	Y	5	S	S	+	UK	SRR3338799
C00025885	I00019864	08-Mar-13	11	Y	5	S	S	+	UK	SRR3338931
C00025821	I00019801	26-Mar-13	11	Y	5	S	S	+	UK	SRR3338658
C00025815	I00019795	01-Apr-13	11	Y	5	S	S	+	UK	SRR3337781
C00025839	I00019818	04-Apr-13	11	Y	5	S	S	+	UK	SRR3338800
C00011151	L-151-13-p1	03-Sep-10	12	N	1	S	S	+	UK	SRR3337755
C00010682	L-113-71-p1	18-Oct-10	12	N	1	S	S	+	UK	SRR3335438
C00010718	L-117-47-p1	14-Dec-10	12	N	1	S	S	+	UK	SRR3338951
C00011822	L-9-68-p1	10-Jan-11	12	N	1	S	S	+	UK	SRR3339364

C00011553	L-L-12-7901458-p1	19-Aug-11	12	N	1	S	S	+	UK	SRR3337517
C00011133	L-135-59-p1	08-Sep-11	12	N	1	S	S	+	UK	SRR3337765
C00011145	L-139-62-p1	25-Oct-11	12	N	1	S	S	+	UK	SRR3338168
C00011969	L-143-18-p1	22-Nov-11	12	N	1	S	S	+	UK	SRR3335475
C00011736	L-L-12-7901570-p1	22-Feb-12	12	N	1	S	S	+	UK	SRR3338680
C00025212	I00019214	23-Jul-12	12	N	1	S	S	+	UK	SRR3336094
C00025314	I00019313	21-Nov-12	12	N	1	S	S	+	UK	SRR3338567
C00025330	I00019328	29-Nov-12	12	N	1	S	S	+	UK	SRR3336033
C00025307	I00019306	14-Dec-12	12	N	1	S	S	+	UK	SRR3337582
C00025461	I00019457	21-Feb-13	12	N	1	S	S	+	UK	SRR3337687
C00011130	L-135-43-p1	10-Sep-11	13	Y	1	S	S	+	UK	SRR3334862
C00012173	L-148-25-p1	22-Mar-12	13	Y	1	S	S	+	UK	SRR3337586
C00012188	L-151-27-p1	04-May-12	13	Y	1	S	S	+	UK	SRR3336098
C00025133	I00019137	04-May-12	13	Y	1	S	S	+	UK	SRR3335175
C00012187	L-151-26-p1	16-May-12	13	Y	1	S	S	+	UK	SRR3338166
C00025118	I00019122	16-May-12	13	Y	1	S	S	+	UK	SRR3338559
C00028667	I00019171	04-Jun-12	13	Y	1	S	S	+	UK	SRR3338233
C00025203	I00019205	31-Aug-12	13	Y	1	S	S	+	UK	SRR3335488
C00025278	I00019278	12-Sep-12	13	Y	1	S	S	+	UK	SRR3339010
C00025394	I00019392	14-Dec-12	13	Y	1	S	S	+	UK	SRR3338736
C00025193	I00019196	19-Dec-12	13	Y	1	S	S	+	UK	SRR3335823
C00025429	I00019425	20-Feb-13	13	Y	1	S	S	+	UK	SRR3335840
C00011892	L-112-76-p1	06-Aug-10	14	Y	1	S	S	+	UK	SRR3335198
C00011894	L-114-21-p1	21-Oct-10	14	Y	1	S	S	+	UK	SRR3334835
C00011803	L-7-93-p1	02-Nov-10	14	Y	1	S	S	+	UK	SRR3334870
C00010690	L-114-62-p1	03-Nov-10	14	Y	1	S	S	+	UK	SRR3335964
C00010698	L-115-48-p1	11-Nov-10	14	Y	1	S	S	+	UK	SRR3338497
C00011913	L-124-30-p1	17-Mar-11	14	Y	1	S	S	+	UK	SRR3335963
C00011858	L-11-49-p1	22-Mar-11	14	Y	1	S	S	+	UK	SRR3335831
C00010585	L-125-16-p1	01-Apr-11	14	Y	1	S	S	+	UK	SRR3335209

C00010583	L-125-6-p1	05-Apr-11	14	Y	1	S	S	+	UK	SRR3338224
C00010605	L-127-22-p1	20-May-11	14	Y	1	S	S	+	UK	SRR3339184
C00011085	L-128-27-p1	02-Jun-11	14	Y	1	S	S	+	UK	SRR3335851
C00011091	L-130-23-p1	28-Jun-11	14	Y	1	S	S	+	UK	SRR3339314
C00011536	L-L-12-7901438-p1	09-Jul-11	14	Y	1	S	S	+	UK	SRR3339315
C00011544	L-L-12-7901447-p1	26-Jul-11	14	Y	1	S	S	+	UK	SRR3338678
C00011125	L-134-80-p1	07-Sep-11	14	Y	1	S	S	+	UK	SRR3335221
C00012206	L-8-3-p1	09-Sep-11	14	Y	1	S	S	+	UK	SRR3338504
C00011672	L-L-12-7901485-p1	05-Oct-11	14	Y	1	S	S	+	UK	SRR3339005
C00011700	L-L-12-7901518-p1	16-Nov-11	14	Y	1	S	S	+	UK	SRR3335301
C00012031	L-146-53-p1	14-Dec-11	14	Y	1	S	S	+	UK	SRR3336039
C00011709	L-L-12-7901533-p1	18-Dec-11	14	Y	1	S	S	+	UK	SRR3335882
C00011738	L-L-12-7901572-p1	26-Feb-12	14	Y	1	S	S	+	UK	SRR3335536
C00012040	L-148-08-p1	25-Mar-12	14	Y	1	S	S	+	UK	SRR3338213
C00012659	L-L-12-7901612-p1	28-Apr-12	14	Y	1	S	S	+	UK	SRR3335193
C00012150	L-L-12-7901623-p1	14-May-12	14	Y	1	S	S	+	UK	SRR3335575
C00025181	I00019184	14-May-12	14	Y	1	S	S	+	UK	SRR3335292
C00012159	L-L-12-7901633-p1	23-May-12	14	Y	1	S	S	+	UK	SRR3335210
C00012160	L-L-12-7901634-p1	23-May-12	14	Y	1	S	S	+	UK	SRR3338948
C00025176	I00019180	23-May-12	14	Y	1	S	S	+	UK	SRR3338207
C00025206	I00019208	24-May-12	14	Y	1	S	S	+	UK	SRR3337777
C00028666	I00019155	03-Jun-12	14	Y	1	S	S	+	UK	SRR3338119
C00025191	I00019194	06-Jun-12	14	Y	1	S	S	+	UK	SRR3338787
C00025145	I00019149	19-Jun-12	14	Y	1	S	S	+	UK	SRR3338654
C00025233	I00019235	22-Jun-12	14	Y	1	S	S	+	UK	SRR3335484
C00025275	I00019275	16-Jul-12	14	Y	1	S	S	+	UK	SRR3338645
C00025283	I00019283	16-Jul-12	14	Y	1	S	S	+	UK	SRR3339346
C00025302	I00019302	16-Sep-12	14	Y	1	S	S	+	UK	SRR3335247
C00025124	I00019128	18-Sep-12	14	Y	1	S	S	+	UK	SRR3338742
C00025271	I00019271	26-Sep-12	14	Y	1	S	S	+	UK	SRR3335451
C00025344	I00019342	28-Sep-12	14	Y	1	S	S	+	UK	SRR3338753

C00025384	I00019382	05-Oct-12	14	Y	1	S	S	+	UK	SRR3335225
C00025392	I00019390	06-Oct-12	14	Y	1	S	S	+	UK	SRR3335563
C00025333	I00019331	20-Oct-12	14	Y	1	S	S	+	UK	SRR3335934
C00025397	I00019395	25-Oct-12	14	Y	1	S	S	+	UK	SRR3338183
C00025377	I00019375	06-Nov-12	14	Y	1	S	S	+	UK	SRR3339352
C00025457	I00019453	10-Dec-12	14	Y	1	S	S	+	UK	SRR3338775
C00025120	I00019124	01-May-13	14	Y	1	S	S	+	UK	SRR3334919
C00025196	I00019199	05-Oct-12	15	N	1	S	S	-	UK	SRR3335877
C00025445	I00019441	20-Feb-13	15	N	1	S	S	-	UK	SRR3338467
C00011888	L-109-14-p1	08-Aug-10	16	Y	1	S	S	+	UK	SRR3335913
C00012098	L-L-12-7901280-p1	06-Sep-10	16	Y	1	S	S	+	UK	SRR3336031
C00011901	L-115-53-p1	25-Oct-10	16	Y	1	S	S	+	UK	SRR3338980
C00011896	L-114-80-p1	02-Nov-10	16	Y	1	S	S	+	UK	SRR3338480
C00011093	L-130-24-p1	02-Jul-11	16	Y	1	S	S	+	UK	SRR3338992
C00011547	L-L-12-7901451-p1	31-Jul-11	16	Y	1	S	S	+	UK	SRR3335223
C00011679	L-L-12-7901494-p1	14-Oct-11	16	Y	1	S	R	+	UK	SRR3338212
C00011698	L-L-12-7901516-p1	16-Nov-11	16	Y	1	S	S	+	UK	SRR3338120
C00012170	L-145-72-p1	11-Feb-12	16	Y	1	S	S	+	UK	SRR3337788
C00012186	L-152-23-p1	24-May-12	16	Y	1	S	S	+	UK	SRR3338231
C00025321	I00019319	15-Oct-12	16	Y	1	S	S	+	UK	SRR3338256
C00012219	L-L-12-7901254-p1	04-Aug-10	17	Y	1	S	S	+	UK	SRR3335454
C00010672	L-112-65-p1	04-Oct-10	17	Y	1	S	S	+	UK	SRR3338719
C00010684	L-114-20-p1	21-Oct-10	17	Y	1	S	S	+	UK	SRR3335551
C00010744	L-120-29-p1	28-Jan-11	17	Y	1	S	S	+	UK	SRR3336090
C00011831	L-10-21-p1	29-Jan-11	17	Y	1	S	S	+	UK	SRR3334866
C00012116	L-L-12-7901468-p1	05-Sep-11	17	Y	1	S	S	+	UK	SRR3339329
C00012663	L-L-12-7901617-p1	11-May-12	17	Y	1	S	S	+	UK	SRR3335607
C00025149	I00019153	11-May-12	17	Y	1	S	S	+	UK	SRR3335444
C00025252	I00019254	04-Aug-12	17	Y	1	S	S	+	UK	SRR3335965
C00025246	I00019248	08-Sep-12	17	Y	1	S	S	+	UK	SRR3335958

C00025286	I00019286	15-Sep-12	17	Y	1	S	S	+	UK	SRR3335584
C00025140	I00019144	23-Sep-12	17	Y	1	S	S	+	UK	SRR3339372
C00025376	I00019374	04-Oct-12	17	Y	1	S	S	+	UK	SRR3335857
C00025380	I00019378	11-Oct-12	17	Y	1	S	S	+	UK	SRR3339342
C00025317	I00019316	17-Oct-12	17	Y	1	S	S	+	UK	SRR3338112
C00025341	I00019339	20-Oct-12	17	Y	1	S	S	+	UK	SRR3335824
C00028670	I00019341	10-Nov-12	17	Y	1	S	S	+	UK	SRR3338686
C00025416	I00019412	14-Nov-12	17	Y	1	S	S	+	UK	SRR3335121
C00025386	I00019384	10-Dec-12	17	Y	1	S	S	+	UK	SRR3338798
C00025473	I00019469	14-Dec-12	17	Y	1	S	S	+	UK	SRR3335585
C00025413	I00019410	19-Feb-13	17	Y	1	S	S	+	UK	SRR3336060
C00011162	L-151-24-p1	09-Oct-10	18	N	1	S	S	+	UK	SRR3338478
C00011520	L-L-12-7901420-p1	07-Jun-11	18	N	1	S	S	+	UK	SRR3339316
C00025192	I00019195	07-Jun-12	18	N	1	S	S	+	UK	SRR3335589
C00025864	I00019843	05-Jul-12	18	N	1	S	S	+	UK	SRR3338515
C00025285	I00019285	29-Aug-12	18	N	1	S	S	-	UK	SRR3338751
C00025115	I00019119	03-Aug-12	20	N	1	S	S	+	UK	SRR3338588
C00011895	L-114-24-p1	10-Aug-10	22	N	3	S	S	+	UK	SRR3338248
C00011132	L-135-58-p1	21-Aug-11	22	N	3	S	S	+	UK	SRR3338685
C00025117	I00019121	05-Dec-12	22	N	3	S	S	+	UK	SRR3335437
C00011153	L-151-15-p1	14-Sep-10	26	N	1	S	S	-	UK	SRR3337739
C00011928	L-132-09-p1	25-Jul-11	26	N	1	S	S	-	UK	SRR3338625
C00011720	L-L-12-7901547-p1	09-Jan-12	26	N	1	S	S	-	UK	SRR3339006
C00012643	L-L-12-7901594-p1	13-Apr-12	26	N	1	S	S	-	UK	SRR3335884
C00025467	I00019463	20-Jan-13	29	N	1	S	S	-	UK	SRR3338667
C00025483	I00019479	20-Jan-13	29	N	1	S	S	-	UK	SRR3337746
C00011935	L-137-33-p1	27-Sep-11	31	N	1	S	S	+	UK	SRR3338521
C00025249	I00019251	22-Jun-12	31	N	1	S	S	-	UK	SRR3338250
C00011885	L-155-36-p1	23-Sep-10	33	N	1	S	S	+	UK	SRR3335286
C00011480	L-L-12-7901323-p1	28-Oct-10	33	N	1	S	S	+	UK	SRR3338894

C00011898	L-115-02-p1	01-Nov-10	33	N	1	S	S	+	UK	SRR3335600
C00011871	L-12-04-p1	08-Apr-11	33	N	1	S	S	+	UK	SRR3335945
C00025348	I00019346	07-Oct-12	33	N	1	S	S	+	UK	SRR3336081
C00012191	L-151-33-p1	21-May-12	34	N	1	S	S	+	UK	SRR3338941
C00025166	I00019170	21-May-12	34	N	1	S	S	+	UK	SRR3339009
C00011800	L-7-64-p1	22-Oct-10	35	N	1	S	S	+	UK	SRR3338572
C00010720	L-117-49-p1	19-Dec-10	35	N	1	S	S	+	UK	SRR3338523
C00012025	L-K6-64-p1	18-Jul-11	35	N	1	S	S	+	UK	SRR3337782
C00011139	L-136-66-p1	29-Sep-11	35	N	1	S	S	+	UK	SRR3338739
C00011142	L-138-56-p1	15-Oct-11	35	N	1	S	S	+	UK	SRR3338726
C00012226	L-L-12-7901265-p1	22-Aug-10	36	Y	1	S	S	+	UK	SRR3337707
C00010745	L-120-79-p1	31-Jan-11	36	Y	1	S	S	+	UK	SRR3336052
C00011854	L-11-16-p1	11-Mar-11	36	Y	1	S	S	+	UK	SRR3335599
C00010597	L-126-15-p1	26-Apr-11	36	Y	1	S	S	+	UK	SRR3335240
C00011537	L-L-12-7901439-p1	13-Jul-11	36	Y	1	S	S	+	UK	SRR3335545
C00011929	L-132-41-p1	01-Aug-11	36	Y	1	S	S	+	UK	SRR3337593
C00011931	L-133-20-p1	12-Aug-11	36	Y	1	S	S	+	UK	SRR3334800
C00011957	L-142-36-p1	15-Dec-11	36	Y	1	S	S	+	UK	SRR3338650
C00011737	L-L-12-7901571-p1	22-Feb-12	36	Y	1	S	S	+	UK	SRR3335532
C00025202	I00019204	02-Aug-12	36	Y	1	S	S	+	UK	SRR3339015
C00025349	I00019347	21-Oct-12	36	Y	1	S	S	+	UK	SRR3335518
C00025334	I00019332	30-Oct-12	36	Y	1	S	S	+	UK	SRR3338676
C00025436	I00019432	22-Jan-13	36	Y	1	S	S	+	UK	SRR3339023
C00025332	I00019330	25-Feb-13	36	Y	1	S	S	+	UK	SRR3337730
C00025877	I00019856	07-Mar-13	36	Y	1	S	S	+	UK	SRR3338777
C00025884	I00019863	18-Mar-13	36	Y	1	S	S	+	UK	SRR3335258
C00010694	L-115-04-p1	24-Oct-10	37	Y	4	S	S	+	UK	SRR3339036
C00011859	L-11-51-p1	23-Mar-11	37	Y	4	S	S	+	UK	SRR3335971
C00011658	L-L-12-7901467-p1	01-Sep-11	37	Y	4	S	S	+	UK	SRR3337766
C00011129	L-135-42-p1	07-Sep-11	37	Y	4	S	S	+	UK	SRR3335283

C00011962	L-142-53-p1	04-Oct-11	37	Y	4	S	S	+	UK	SRR3338908
C00012660	L-L-12-7901613-p1	28-Apr-12	37	Y	4	S	S	+	UK	SRR3338076
C00025385	I00019383	11-Nov-12	37	Y	4	S	S	+	UK	SRR3337752
C00025444	I00019440	24-Jan-13	37	Y	4	S	S	+	UK	SRR3335813
C00025823	I00019803	02-Apr-13	37	Y	4	S	S	+	UK	SRR3336002
C00011747	L-L-12-7901583-p1	16-Mar-12	41	N	2	S	S	+	UK	SRR3337575
C00010664	L-111-28-p1	29-Aug-10	42	Y	1	R	S	+	UK	SRR3335179
C00010679	L-113-24-p1	02-Sep-10	42	Y	1	R	S	+	UK	SRR3338275
C00010673	L-112-74-p1	21-Sep-10	42	Y	1	R	S	+	UK	SRR3335820
C00011161	L-151-23-p1	04-Oct-10	42	Y	1	R	S	+	UK	SRR3338195
C00006867	L-116-33-p1	11-Nov-10	42	Y	1	R	S	+	UK	SRR3338964
C00010700	L-115-75-p1	16-Nov-10	42	Y	1	R	S	+	UK	SRR3338525
C00011485	L-L-12-7901332-p1	21-Nov-10	42	Y	1	R	S	+	UK	SRR3334843
C00006903	L-118-41-p1	17-Dec-10	42	Y	1	R	S	+	UK	SRR3335244
C00011992	L-K2-79-p1	11-Jan-11	42	Y	1	R	S	+	UK	SRR3335535
C00011993	L-K2-80-p1	12-Jan-11	42	Y	1	R	S	+	UK	SRR3335462
C00011839	L-10-48-p1	08-Feb-11	42	Y	1	R	S	+	UK	SRR3335576
C00011998	L-K4-18-p1	23-Feb-11	42	Y	1	R	S	+	UK	SRR3335993
C00010573	L-124-15-p1	02-Mar-11	42	Y	1	R	S	+	UK	SRR3335181
C00011121	L-134-05-p1	12-Aug-11	42	Y	1	R	S	+	UK	SRR3335218
C00011110	L-133-33-p1	15-Aug-11	42	Y	1	R	S	+	UK	SRR3338377
C00012204	L-7-71-p1	31-Aug-11	42	Y	1	R	S	+	UK	SRR3334804
C00011664	L-L-12-7901474-p1	13-Sep-11	42	Y	1	R	S	+	UK	SRR3334909
C00011712	L-L-12-7901537-p1	23-Dec-11	42	Y	1	R	S	+	UK	SRR3335977
C00012172	L-148-24-p1	30-Mar-12	42	Y	1	R	S	+	UK	SRR3334908
C00012192	L-151-34-p1	08-May-12	42	Y	1	R	S	+	UK	SRR3338661
C00025141	I00019145	08-May-12	42	Y	1	R	S	+	UK	SRR3339326
C00012156	L-L-12-7901630-p1	22-May-12	42	Y	1	R	S	+	UK	SRR3338506
C00025168	I00019172	22-May-12	42	Y	1	R	S	+	UK	SRR3337677
C00025113	I00019118	14-Jun-12	42	Y	1	R	S	+	UK	SRR3335151

C00025296	I00019296	17-Jun-12	42	Y	1	R	S	+	UK	SRR3335171
C00025186	I00019189	30-Jul-12	42	Y	1	R	S	+	UK	SRR3339020
C00025171	I00019175	15-Aug-12	42	Y	1	R	S	+	UK	SRR3339308
C00025352	I00019350	03-Oct-12	42	Y	1	R	S	+	UK	SRR3337662
C00025374	I00019372	04-Nov-12	42	Y	1	R	S	+	UK	SRR3335531
C00025355	I00019353	18-Jan-13	42	Y	1	R	S	+	UK	SRR3335463
C00025404	I00019402	21-Jan-13	42	Y	1	R	S	+	UK	SRR3335930
C00025395	I00019393	04-Feb-13	42	Y	1	R	S	+	UK	SRR3335449
C00025437	I00019433	20-Feb-13	42	Y	1	R	S	+	UK	SRR3335262
C00010658	L-110-17-p1	20-Aug-10	43	N	1	S	S	+	UK	SRR3338774
C00011159	L-151-21-p1	28-Sep-10	43	N	1	S	S	+	UK	SRR3338126
C00010692	L-114-81-p1	24-Oct-10	43	N	1	S	S	+	UK	SRR3338892
C00011927	L-131-48-p1	07-Jan-11	43	N	1	S	S	+	UK	SRR3335561
C00010736	L-120-6-p1	25-Jan-11	43	N	1	S	S	+	UK	SRR3338973
C00011836	L-10-41-p1	05-Feb-11	43	N	1	S	S	+	UK	SRR3338169
C00011999	L-K4-27-p1	28-Feb-11	43	N	1	S	S	+	UK	SRR3339307
C00012029	L-146-51-p1	18-Feb-12	43	N	1	S	S	+	UK	SRR3338460
C00012653	L-L-12-7901605-p1	22-Apr-12	43	N	1	S	S	+	UK	SRR3337749
C00010665	L-111-29-p1	17-Aug-10	44	Y	1	S	S	+	UK	SRR3335269
C00012227	L-L-12-7901266-p1	25-Aug-10	44	Y	1	S	S	+	UK	SRR3336091
C00011152	L-151-14-p1	04-Sep-10	44	Y	1	S	S	+	UK	SRR3335960
C00006914	L-112-73-p1	15-Sep-10	44	Y	1	S	S	+	UK	SRR3338226
C00011468	L-L-12-7901297-p1	22-Sep-10	44	Y	1	S	S	+	UK	SRR3335436
C00007734	L-113-57-p1	13-Oct-10	44	Y	1	S	S	+	UK	SRR3338118
C00011796	L-7-48-p1	13-Oct-10	44	Y	1	S	S	+	UK	SRR3336084
C00006843	L-114-63-p1	24-Oct-10	44	Y	1	S	S	+	UK	SRR3337764
C00006855	L-116-31-p1	20-Nov-10	44	Y	1	S	S	+	UK	ERS243753
C00011805	L-8-62-p1	23-Nov-10	44	Y	1	S	S	+	UK	SRR3335440
C00007993	L-117-26-p2	04-Dec-10	44	Y	1	S	S	+	UK	SRR3336065
C00011811	L-9-02-p1	13-Dec-10	44	Y	1	S	S	+	UK	SRR3334839

C00011498	L-L-12-7901355-p1	18-Jan-11	44	Y	1	S	S	+	UK	SRR3335565
C00011832	L-10-22-p1	30-Jan-11	44	Y	1	S	S	+	UK	SRR3337512
C00011846	L-10-76-p1	17-Feb-11	44	Y	1	S	S	+	UK	SRR3337519
C00010606	L-127-33-p1	28-Mar-11	44	Y	1	S	S	+	UK	SRR3338907
C00011862	L-11-69-p1	30-Mar-11	44	Y	1	S	S	+	UK	SRR3338675
C00011869	L-11-88-p1	04-Apr-11	44	Y	1	S	S	+	UK	SRR3338123
C00010599	L-126-17-p1	02-May-11	44	Y	1	S	S	+	UK	SRR3335886
C00011878	L-12-50-p1	03-May-11	44	Y	1	S	S	+	UK	SRR3335875
C00011079	L-127-62-p1	13-May-11	44	Y	1	S	S	+	UK	SRR3337798
C00011882	L-12-76-p1	16-May-11	44	Y	1	S	S	+	UK	SRR3335978
C00012007	L-K5-48-p1	29-May-11	44	Y	1	S	S	+	UK	SRR3335429
C00011090	L-129-44-p1	19-Jun-11	44	Y	1	S	S	+	UK	SRR3335911
C00012166	L-K6-18-p1	22-Jun-11	44	Y	1	S	S	+	UK	SRR3335461
C00011100	L-130-53-p1	23-Jun-11	44	Y	1	S	S	+	UK	SRR3338993
C00011531	L-L-12-7901433-p1	30-Jun-11	44	Y	1	S	S	+	UK	SRR3335847
C00011098	L-130-51-p1	03-Jul-11	44	Y	1	S	S	+	UK	SRR3338099
C00011104	L-130-79-p1	07-Jul-11	44	Y	1	S	S	+	UK	SRR3339320
C00011981	L-K6-69-p1	21-Jul-11	44	Y	1	S	S	+	UK	SRR3338507
C00011548	L-L-12-7901452-p1	04-Aug-11	44	Y	1	S	S	+	UK	SRR3335173
C00011124	L-134-44-p1	11-Aug-11	44	Y	1	S	S	+	UK	SRR3339343
C00011135	L-136-09-p1	15-Sep-11	44	Y	1	S	S	+	UK	SRR3338266
C00012209	L-8-15-p1	21-Sep-11	44	Y	1	S	S	+	UK	SRR3339004
C00012210	L-8-18-p1	23-Sep-11	44	Y	1	S	S	+	UK	SRR3335113
C00011674	L-L-12-7901487-p1	10-Oct-11	44	Y	1	S	S	+	UK	SRR3339380
C00011693	L-L-12-7901509-p1	01-Nov-11	44	Y	1	S	S	+	UK	SRR3335929
C00011696	L-L-12-7901514-p1	10-Nov-11	44	Y	1	S	S	+	UK	SRR3338141
C00012034	L-146-66-p1	29-Jan-12	44	Y	1	S	S	+	UK	SRR3334940
C00012027	L-146-10-p1	08-Feb-12	44	Y	1	S	S	+	UK	SRR3338756
C00011980	L-145-31-p1	14-Feb-12	44	Y	1	S	S	+	UK	SRR3338122
C00011751	L-L-12-7901588-p1	27-Mar-12	44	Y	1	S	S	+	UK	SRR3339377
C00011750	L-L-12-7901587-p1	28-Mar-12	44	Y	1	S	S	+	UK	SRR3337705

C00012640	L-L-12-7901590-p1	01-Apr-12	44	Y	1	S	S	+	UK	SRR3339014
C00012175	L-148-75-p1	05-Apr-12	44	Y	1	S	S	+	UK	SRR3335205
C00012178	L-149-50-p1	13-Apr-12	44	Y	1	S	S	+	UK	SRR3335189
C00012658	L-L-12-7901611-p1	26-Apr-12	44	Y	1	S	S	+	UK	SRR3338518
C00025142	I00019146	19-May-12	44	Y	1	S	S	+	UK	SRR3338114
C00012158	L-L-12-7901632-p1	22-May-12	44	Y	1	S	S	+	UK	SRR3335249
C00025182	I00019185	22-May-12	44	Y	1	S	S	+	UK	SRR3336066
C00025258	I00019260	05-Jul-12	44	Y	1	S	S	+	UK	SRR3335583
C00025227	I00019229	11-Jul-12	44	Y	1	S	S	+	UK	SRR3334863
C00025162	I00019166	25-Jul-12	44	Y	1	S	S	+	UK	SRR3339330
C00028668	I00019249	22-Sep-12	44	Y	1	S	S	+	UK	SRR3335241
C00025255	I00019257	26-Sep-12	44	Y	1	S	S	+	UK	SRR3338985
C00025337	I00019335	22-Oct-12	44	Y	1	S	S	+	UK	SRR3338131
C00025464	I00019460	25-Nov-12	44	Y	1	S	S	+	UK	SRR3335596
C00025481	I00019477	16-Dec-12	44	Y	1	S	S	+	UK	SRR3337716
C00025412	I00019409	21-Jan-13	44	Y	1	S	S	+	UK	SRR3338974
C00025853	I00019832	01-Mar-13	44	Y	1	S	S	+	UK	SRR3338794
C00025829	I00019808	27-Mar-13	44	Y	1	S	S	+	UK	SRR3339028
C00025855	I00019834	04-Apr-13	44	Y	1	S	S	+	UK	SRR3335448
C00011810	L-8-99-p1	12-Dec-10	45	N	1	S	S	+	UK	SRR3335195
C00011865	L-11-75-p1	01-Apr-11	45	N	1	S	S	+	UK	SRR3338573
C00011932	L-134-43-p1	30-Aug-11	45	N	1	S	S	+	UK	SRR3338534
C00011675	L-L-12-7901488-p1	10-Oct-11	45	N	1	S	S	+	UK	SRR3338611
C00011971	L-143-20-p1	31-Dec-11	45	N	1	S	S	+	UK	SRR3337666
C00012174	L-148-74-p1	05-Apr-12	45	N	1	S	S	+	UK	SRR3338375
C00025184	I00019187	06-Jun-12	45	N	1	S	S	+	UK	SRR3338568
C00025180	I00019183	28-Sep-12	45	N	1	S	S	+	UK	SRR3337584
C00025310	I00019309	27-Oct-12	45	N	1	S	S	+	UK	SRR3335164
C00028671	I00019357	13-Nov-12	45	N	1	S	S	+	UK	SRR3335206
C00025472	I00019468	26-Nov-12	45	N	1	S	S	+	UK	SRR3338902

C00025805	I00019785	20-Mar-13	45	N	1	S	S	+	UK	SRR3335172
C00025813	I00019793	26-Mar-13	45	N	1	S	S	+	UK	SRR3335533
C00011148	L-151-10-p1	16-Aug-10	46	N	1	S	S	+	UK	SRR3335614
C00014381	L-L-12-7901378-p1	01-Mar-11	46	N	1	S	S	+	UK	SRR3337580
C00011919	L-124-74-p1	19-Mar-11	46	N	1	S	S	+	UK	SRR3338260
C00011515	L-L-12-7901415-p1	25-May-11	46	N	1	S	S	+	UK	SRR3338140
C00011741	L-L-12-7901576-p1	03-Mar-12	46	N	1	S	S	+	UK	SRR3335160
C00012182	L-150-01-p1	22-Apr-12	46	N	1	S	S	+	UK	SRR3338694
C00012655	L-L-12-7901608-p1	24-Apr-12	46	N	1	S	S	+	UK	SRR3338220
C00012148	L-L-12-7901620-p1	11-May-12	46	N	1	S	S	+	UK	SRR3338130
C00025157	I00019161	11-May-12	46	N	1	S	S	+	UK	SRR3336062
C00028669	I00019264	26-Sep-12	46	N	1	S	S	+	UK	SRR3338539
C00025347	I00019345	14-Jan-13	46	N	1	S	S	+	UK	SRR3338482
C00025797	I00019777	20-Mar-13	46	N	1	S	S	+	UK	SRR3335829
C00011466	L-L-12-7901291-p1	20-Sep-10	48	N	1	S	S	+	UK	SRR3338565
C00011964	L-142-57-p1	18-Dec-11	48	N	1	S	S	+	UK	SRR3338100
C00025305	I00019304	06-Oct-12	48	N	1	S	S	-	UK	SRR3337738
C00012101	L-L-12-7901333-p1	21-Nov-10	49	Y	1	S	S	+	UK	SRR3338655
C00011081	L-128-18-p1	04-Jun-11	49	Y	1	S	S	+	UK	SRR3334943
C00012020	L-K6-33-p1	30-Jun-11	49	Y	1	S	S	+	UK	SRR3338273
C00011557	L-L-12-7901462-p1	24-Aug-11	49	Y	1	S	S	+	UK	SRR3338548
C00011713	L-L-12-7901538-p1	26-Dec-11	49	Y	1	S	S	+	UK	SRR3337715
C00012162	L-L-12-7901636-p1	25-May-12	49	Y	1	S	S	+	UK	SRR3338672
C00025119	I00019123	25-May-12	49	Y	1	S	S	+	UK	SRR3335261
C00025199	I00019202	06-Jun-12	49	Y	1	S	S	+	UK	SRR3338671
C00025248	I00019250	10-Jun-12	49	Y	1	S	S	+	UK	SRR3338110
C00025147	I00019151	12-Aug-12	49	Y	1	S	S	+	UK	SRR3335880
C00025187	I00019190	24-Aug-12	49	Y	1	S	S	+	UK	SRR3338551
C00025350	I00019348	01-Nov-12	49	Y	1	S	S	+	UK	SRR3335588
C00025410	I00019407	24-Dec-12	49	Y	1	S	S	+	UK	SRR3334890

C00025872	I00019851	31-Dec-12	49	Y	1	S	S	+	UK	SRR3338935
C00025798	I00019778	13-Mar-13	49	Y	1	S	S	+	UK	SRR3335146
C00011686	L-L-12-7901500-p1	20-Oct-11	51	N	1	S	S	+	UK	SRR3338068
C00011938	L-140-60-p1	13-Nov-11	51	N	1	S	S	+	UK	SRR3335375
C00011156	L-151-18-p1	20-Sep-10	53	N	1	S	S	+	UK	SRR3335277
C00011955	L-142-25-p1	06-Dec-11	53	N	1	S	S	+	UK	SRR3337778
C00012037	L-146-80-p1	06-Mar-12	53	N	1	S	S	+	UK	SRR3335297
C00010702	L-116-29-p1	22-Nov-10	54	Y	1	S	S	+	UK	SRR3336053
C00011814	L-9-07-p1	15-Dec-10	54	Y	1	S	S	+	UK	SRR3338263
C00011851	L-10-99-p1	02-Mar-11	54	Y	1	S	S	+	UK	SRR3338716
C00011084	L-128-26-p1	02-Jun-11	54	Y	1	S	S	+	UK	SRR3339296
C00011107	L-132-19-p1	24-Jun-11	54	Y	1	S	S	+	UK	SRR3335928
C00011546	L-L-12-7901450-p1	28-Jul-11	54	Y	1	S	S	+	UK	SRR3338072
C00012203	L-7-70-p1	03-Sep-11	54	Y	1	S	S	+	UK	SRR3334816
C00011952	L-142-22-p1	26-Sep-11	54	Y	1	S	S	+	UK	SRR3335162
C00011695	L-L-12-7901512-p1	09-Nov-11	54	Y	1	S	S	+	UK	SRR3338258
C00011711	L-L-12-7901536-p1	21-Dec-11	54	Y	1	S	S	+	UK	SRR3337674
C00012152	L-L-12-7901626-p1	15-May-12	54	Y	1	S	S	+	UK	SRR3335556
C00025189	I00019192	15-May-12	54	Y	1	S	S	+	UK	SRR3335473
C00025224	I00019226	07-Jun-12	54	Y	1	S	S	+	UK	SRR3335925
C00025256	I00019258	10-Jun-12	54	Y	1	S	S	+	UK	SRR3335916
C00025218	I00019220	29-Jun-12	54	Y	1	S	S	+	UK	SRR3334871
C00025226	I00019228	01-Jul-12	54	Y	1	S	S	+	UK	SRR3338077
C00025214	I00019216	06-Sep-12	54	Y	1	S	S	+	UK	SRR3339319
C00025382	I00019380	04-Nov-12	54	Y	1	S	S	+	UK	SRR3338714
C00025489	I00019484	19-Dec-12	54	Y	1	S	S	+	UK	SRR3337761
C00011471	L-L-12-7901303-p1	28-Sep-10	55	Y	1	S	S	+	UK	SRR3338956
C00011492	L-L-12-7901347-p1	05-Jan-11	55	Y	1	S	S	+	UK	SRR3335260
C00011874	L-12-21-p1	18-Apr-11	55	Y	1	S	S	+	UK	SRR3335968
C00011535	L-L-12-7901437-p1	08-Jul-11	55	Y	1	S	S	+	UK	SRR3338912

C00012202	L-7-60-p1	27-Aug-11	55	Y	1	S	S	+	UK	SRR3338904
C00011668	L-L-12-7901480-p1	29-Sep-11	55	Y	1	S	S	+	UK	SRR3338647
C00011940	L-140-77-p1	20-Oct-11	55	Y	1	S	S	+	UK	SRR3335148
C00025353	I00019351	26-Oct-12	55	Y	1	S	S	+	UK	SRR3335567
C00025338	I00019336	29-Nov-12	55	Y	1	S	S	+	UK	SRR3337685
C00011160	L-151-22-p1	01-Oct-10	56	N	1	S	S	+	UK	SRR3338662
C00025244	I00019246	03-Aug-12	56	N	1	S	S	+	UK	SRR3334854
C00025276	I00019276	07-Aug-12	56	N	1	S	S	+	UK	SRR3336004
C00025253	I00019255	23-Aug-12	56	N	1	S	S	+	UK	SRR3339361
C00025131	I00019135	08-Aug-12	57	N	1	S	S	+	UK	SRR3339340
C00011150	L-151-12-p1	02-Sep-10	58	Y	1	S	S	+	UK	SRR3335838
C00011818	L-9-29-p1	23-Dec-10	58	Y	1	S	S	+	UK	SRR3338897
C00011850	L-10-94-p1	27-Feb-11	58	Y	1	S	S	+	UK	SRR3335985
C00011877	L-12-47-p1	02-May-11	58	Y	1	S	S	+	UK	SRR3334806
C00011556	L-L-12-7901461-p1	24-Aug-11	58	Y	1	S	S	+	UK	SRR3338246
C00011558	L-L-12-7901463-p1	25-Aug-11	58	Y	1	S	S	+	UK	SRR3339040
C00011661	L-L-12-7901471-p1	05-Sep-11	58	Y	1	S	S	+	UK	SRR3337757
C00012033	L-146-65-p1	09-Jan-12	58	Y	1	S	S	+	UK	SRR3335601
C00012039	L-147-49-p1	16-Mar-12	58	Y	1	S	S	+	UK	SRR3338669
C00012646	L-L-12-7901598-p1	17-Apr-12	58	Y	1	S	S	+	UK	SRR3338784
C00025425	I00019421	06-Dec-12	58	Y	1	S	S	+	UK	SRR3337665
C00025443	I00019439	14-Jan-13	58	Y	1	S	S	+	UK	SRR3335367
C00025807	I00019787	31-Mar-13	58	Y	1	S	S	+	UK	SRR3338637
C00011717	L-L-12-7901543-p1	30-Dec-11	63	N	1	S	S	+	UK	SRR3338458
C00025273	I00019273	26-Jun-12	63	N	1	S	S	+	UK	SRR3335299
C00010569	L-123-64-p1	17-Mar-11	66	N	1	S	S	+	UK	SRR3336059
C00011799	L-7-61-p1	22-Oct-10	76	N	1	S	S	+	UK	SRR3336080
C00011524	L-L-12-7901424-p1	08-Jun-11	76	N	1	S	S	+	UK	SRR3335812
C00011893	L-113-27-p1	28-Sep-10	80	N	1	S	S	+	UK	SRR3338188

C00011900	L-115-44-p1	11-Nov-10	82	N	1	S	S	+	UK	SRR3338191
C00025172	I00019176	28-Sep-12	82	N	1	S	S	+	UK	SRR3335926
C00025458	I00019454	29-Dec-12	82	N	1	S	S	+	UK	SRR3335859
C00010674	L-112-75-p1	01-Sep-10	92	N	1	S	S	+	UK	SRR3338947
C00010677	L-113-19-p1	02-Oct-10	92	N	1	S	S	+	UK	SRR3339383
C00011976	L-144-08-p1	17-Jan-12	102	N	1	S	S	+	UK	SRR3335276
C00012157	L-L-12-7901631-p1	22-May-12	103	N	1	S	S	+	UK	SRR3338943
C00025174	I00019178	22-May-12	103	N	1	S	S	+	UK	SRR3335453
C00011899	L-115-42-p1	09-Nov-10	114	N	2	S	S	+	UK	SRR3335981
C00012652	L-L-12-7901603-p1	21-Apr-12	122	N	-	S	S	+	UK	SRR3334820
C00025152	I00019156	15-May-12	122	N	-	S	S	+	UK	SRR3335508
C00011903	L-116-13-p1	13-Nov-10	123	N	2	S	S	+	UK	SRR3337514
C00025346	I00019344	29-Nov-12	130	N	2	S	S	+	UK	SRR3335870
C00025354	I00019352	01-Dec-12	130	N	2	S	S	+	UK	SRR3335593
C00011978	L-144-80-p1	08-Jan-12	133	N	1	S	S	+	UK	SRR3338989
C00011948	L-141-73-p1	10-Dec-11	139	N	1	S	S	+	UK	SRR3334828
C00011733	L-L-12-7901566-p1	16-Feb-12	152	N	1	S	S	+	UK	ERS216174
C00025421	I00019417	19-Feb-13	234	N	1	S	S	+	UK	SRR3335275
C00011688	L-L-12-7901502-p1	22-Oct-11	245	N	1	S	S	+	UK	SRR3336082
C00011495	L-L-12-7901350-p1	10-Jan-11	263	N	1	S	S	+	UK	SRR3337724

(c) Isolate collection: Oxfordshire Human Clinical Isolates (from Enzyme Immunoassay Negative Samples)

Genome Identification Number	Isolate Name	Isolation Date	ST	Phylogeny	clade	*gyrA*	*gyrB*	PaLoc	Country	SRA Accession
C00011214	TN142-p1	13-Jun-11	1	N	2	R	S	+	UK	SRR3337744
C00011250	TN145-p1	15-Jun-11	1	N	2	S	S	+	UK	SRR3335170
C00009697	TN46-p1	01-Feb-11	2	Y	1	S	S	+	UK	SRR3339002
C00009784	TN78-p1	03-Mar-11	2	Y	1	S	S	+	UK	SRR3336055
C00011168	TN101-p1	29-Mar-11	2	Y	1	S	S	+	UK	SRR3335883
C00011222	TN112-p1	22-May-11	2	Y	1	S	S	+	UK	SRR3338477
C00011247	TN121-p1	24-May-11	2	Y	1	S	S	+	UK	SRR3338802
C00011259	TN122-p1	25-May-11	2	Y	1	S	S	+	UK	SRR3334878
C00009752	TN161-p1	27-Jun-11	2	Y	1	S	S	+	UK	SRR3335869
C00009706	TN172-p1	05-Jul-11	2	Y	1	S	S	+	UK	SRR3335215
C00009791	TN187-p1	20-Jul-11	2	Y	1	S	S	+	UK	SRR3339953
C00009780	TN194-p1	01-Aug-11	2	Y	1	S	S	+	UK	SRR3335450
C00009792	TN195-p1	03-Aug-11	2	Y	1	S	S	+	UK	SRR3338642
C00009793	TN196-p1	04-Aug-11	2	Y	1	S	S	+	UK	SRR3335374
C00011204	TN209-p1	22-Aug-11	2	Y	1	S	S	+	UK	SRR3334848
C00011228	TN211-p1	23-Aug-11	2	Y	1	S	S	+	UK	SRR3335243
C00009855	TN221-p1	04-Sep-11	2	Y	1	S	S	+	UK	SRR3339373
C00009857	TN237-p1	19-Sep-11	2	Y	1	S	S	+	UK	SRR3339017
C00012707	3316-p1	22-Apr-12	2	Y	1	S	S	+	UK	SRR3335246
C00012695	3292-p1	30-Apr-12	2	Y	1	S	S	+	UK	SRR3334823
C00012714	3324-p1	06-May-12	2	Y	1	S	S	+	UK	SRR3334812
C00012715	3326-p1	13-May-12	2	Y	1	S	S	+	UK	SRR3338154
C00012720	3331-p1	14-May-12	2	Y	1	S	S	+	UK	SRR3335153
C00014312	3477-p1	16-Jun-12	2	Y	1	S	S	+	UK	SRR3338754
C00014317	3486-p1	19-Jun-12	2	Y	1	S	S	+	UK	SRR3338514
C00014321	3492-p1	04-Jul-12	2	Y	1	S	S	+	UK	SRR3338968
C00015504	3653-p1	10-Oct-12	2	Y	1	S	S	+	UK	SRR3338600

C00015507	3657-p1	12-Oct-12	2	Y	1	S	S	+	UK	SRR3336046
C00020399	3822-p1	10-Feb-13	2	Y	1	S	S	+	UK	SRR3335122
C00020401	3824-p1	18-Feb-13	2	Y	1	S	S	+	UK	SRR3334948
C00009733	TN49-p1	06-Feb-11	3	N	1	S	S	-	UK	SRR3335147
C00009773	TN85-p1	10-Mar-11	3	N	1	S	S	+	UK	SRR3338101
C00009718	TN173-p1	05-Jul-11	3	N	1	S	S	+	UK	SRR3337576
C00009853	TN201-p1	13-Aug-11	3	N	1	S	S	-	UK	SRR3337663
C00013885	3454-p1	26-May-12	3	N	1	S	S	-	UK	SRR3335167
C00015452	3578-p1	06-Sep-12	3	N	1	S	S	+	UK	SRR3338917
C00020386	3803-p1	24-Jan-13	3	N	1	S	S	-	UK	SRR3335517
C00020418	3850-p1	05-Mar-13	3	N	1	S	S	+	UK	SRR3335433
C00009862	TN214-p1	29-Aug-11	4	N	1	S	S	+	UK	SRR3339341
C00020369	3782-p1	05-Jan-13	4	N	1	S	S	+	UK	SRR3335956
C00009921	TN14-p1	06-Jan-11	5	N	3	S	S	+	UK	SRR3336076
C00009781	TN53-p1	09-Feb-11	5	N	3	S	S	+	UK	SRR3335522
C00011258	TN114-p1	23-May-11	5	N	3	S	S	+	UK	SRR3337596
C00009810	TN241-p1	21-Sep-11	5	N	3	S	S	+	UK	SRR3335191
C00009945	TN16-p1	10-Jan-11	6	Y	1	S	S	+	UK	SRR3337726
C00009984	TN43-p1	30-Jan-11	6	Y	1	S	S	+	UK	SRR3338219
C00009698	TN54-p1	10-Feb-11	6	Y	1	S	S	+	UK	SRR3335915
C00009758	TN59-p1	14-Feb-11	6	Y	1	S	S	+	UK	SRR3338554
C00009782	TN61-p1	14-Feb-11	6	Y	1	S	S	+	UK	SRR3337590
C00009711	TN63-p1	15-Feb-11	6	Y	1	S	S	+	UK	SRR3335169
C00009702	TN87-p1	14-Mar-11	6	Y	1	S	S	+	UK	SRR3338938
C00011200	TN125-p1	31-May-11	6	Y	1	S	S	+	UK	SRR3337609
C00011192	TN156-p1	22-Jun-11	6	Y	1	S	S	+	UK	SRR3339021
C00009704	TN157-p1	26-Jun-11	6	Y	1	S	S	+	UK	SRR3338071
C00009789	TN171-p1	05-Jul-11	6	Y	1	S	S	+	UK	SRR3338909
C00013837	3400-p1	01-Apr-12	6	Y	1	S	S	+	UK	SRR3339339
C00012665	3255-p1	13-Apr-12	6	Y	1	S	S	+	UK	SRR3334886

C00013823	3382-p1	18-Apr-12	6	Y	1	S	S	+	UK	SRR3339358
C00012692	3287-p1	03-May-12	6	Y	1	S	S	+	UK	SRR3334813
C00012699	3299-p1	10-May-12	6	Y	1	S	S	+	UK	SRR3338727
C00013831	3391-p1	15-May-12	6	Y	1	S	S	+	UK	SRR3335665
C00014326	3497-p1	06-Jul-12	6	Y	1	S	S	+	UK	SRR3335245
C00014332	3507-p1	03-Aug-12	6	Y	1	S	S	+	UK	SRR3338163
C00015460	3589-p1	13-Sep-12	6	Y	1	S	S	+	UK	SRR3338975
C00015480	3610-p1	25-Sep-12	6	Y	1	S	S	+	UK	SRR3335879
C00015503	3652-p1	10-Oct-12	6	Y	1	S	S	+	UK	SRR3338579
C00016338	3674-p1	24-Oct-12	6	Y	1	S	S	+	UK	SRR3335255
C00020142	3770-p1	29-Dec-12	6	Y	1	S	S	+	UK	SRR3338729
C00020392	3811-p1	22-Feb-13	6	Y	1	S	S	+	UK	SRR3335896
C00021216	3881-p1	25-Mar-13	6	Y	1	S	S	+	UK	SRR3336073
C00009982	TN27-p1	19-Jan-11	7	Y	1	S	S	-	UK	ERS243723
C00009935	TN31-p1	24-Jan-11	7	Y	1	S	S	-	UK	ERS243724
C00009900	TN36-p1	26-Jan-11	7	Y	1	S	S	-	UK	ERS243725
C00009772	TN77-p1	03-Mar-11	7	Y	1	S	S	-	UK	ERS243726
C00009701	TN79-p1	03-Mar-11	7	Y	1	S	S	-	UK	ERS243727
C00009714	TN88-p1	14-Mar-11	7	Y	1	S	S	-	UK	ERS243728
C00011199	TN117-p1	23-May-11	7	Y	1	S	S	+	UK	SRR3339034
C00011201	TN133-p1	06-Jun-11	7	Y	1	S	S	-	UK	ERS243710
C00011226	TN143-p1	15-Jun-11	7	Y	1	S	S	-	UK	ERS243711
C00009716	TN158-p1	26-Jun-11	7	Y	1	S	S	-	UK	ERS243712
C00009705	TN164-p1	28-Jun-11	7	Y	1	S	S	-	UK	ERS243713
C00009730	TN174-p1	05-Jul-11	7	Y	1	S	S	-	UK	ERS243714
C00009754	TN176-p1	10-Jul-11	7	Y	1	S	S	-	UK	ERS243715
C00009790	TN179-p1	13-Jul-11	7	Y	1	S	S	-	UK	ERS243716
C00009755	TN184-p1	19-Jul-11	7	Y	1	S	S	-	UK	ERS243717
C00009743	TN183-p1	19-Jul-11	7	Y	1	S	S	+	UK	SRR3338370
C00009877	TN202-p1	15-Aug-11	7	Y	1	S	S	-	UK	ERS243718

C00011240	TN212-p1	23-Aug-11	7	Y	1	S	S	-	UK	ERS243719
C00009854	TN213-p1	24-Aug-11	7	Y	1	S	S	+	UK	ERS243720
C00011252	TN225-p1	07-Sep-11	7	Y	1	S	S	-	UK	ERS243721
C00009879	TN238-p1	19-Sep-11	7	Y	1	S	S	+	UK	ERS243722
C00012701	3306-p1	01-Apr-12	7	Y	1	R	S	-	UK	SRR3335279
C00013811	3359-p1	10-Apr-12	7	Y	1	S	S	-	UK	SRR3335590
C00013832	3392-p1	15-May-12	7	Y	1	S	S	-	UK	SRR3335507
C00012677	3271-p1	18-May-12	7	Y	1	S	S	-	UK	SRR3335554
C00013858	3423-p1	28-May-12	7	Y	1	S	S	+	UK	SRR3338995
C00014323	3494-p1	16-Jun-12	7	Y	1	S	S	-	UK	SRR3338628
C00014344	3525-p1	07-Aug-12	7	Y	1	S	S	-	UK	SRR3334925
C00015463	3593-p1	20-Sep-12	7	Y	1	S	S	-	UK	SRR3338782
C00015494	3633-p3	27-Sep-12	7	Y	1	S	S	-	UK	SRR3339376
C00015500	3646-p1	03-Oct-12	7	Y	1	S	S	+	UK	SRR3335226
C00015501	3648-p1	09-Oct-12	7	Y	1	S	S	-	UK	SRR3338526
C00015506	3655-p1	12-Oct-12	7	Y	1	S	S	-	UK	SRR3338779
C00016344	3683-p1	29-Oct-12	7	Y	1	S	S	-	UK	SRR3335907
C00016353	3695-p1	05-Nov-12	7	Y	1	S	S	-	UK	SRR3338791
C00020119	3746-p1	01-Dec-12	7	Y	1	S	S	-	UK	SRR3334841
C00020409	3838-p1	13-Feb-13	7	Y	1	S	S	-	UK	SRR3335845
C00009931	TN1-p1	03-Dec-10	8	Y	1	S	S	+	UK	SRR3335526
C00009968	TN10-p1	14-Dec-10	8	Y	1	S	S	+	UK	SRR3335546
C00009923	TN30-p1	23-Jan-11	8	Y	1	S	S	+	UK	SRR3338371
C00009721	TN48-p1	03-Feb-11	8	Y	1	S	S	+	UK	SRR3337600
C00009774	TN93-p1	21-Mar-11	8	Y	1	S	S	+	UK	SRR3338543
C00009727	TN98-p1	26-Mar-11	8	Y	1	S	S	+	UK	SRR3338558
C00011202	TN141-p1	12-Jun-11	8	Y	1	S	S	+	UK	SRR3335296
C00011179	TN147-p1	16-Jun-11	8	Y	1	S	S	+	UK	SRR3335527
C00011215	TN150-p1	18-Jun-11	8	Y	1	S	S	+	UK	SRR3339311
C00009717	TN165-p1	28-Jun-11	8	Y	1	S	S	+	UK	SRR3338687

C00009779	TN186-p1	19-Jul-11	8	Y	1	S	S	+	UK	SRR3335937
C00009845	TN236-p1	19-Sep-11	8	Y	1	S	S	+	UK	SRR3339025
C00009846	TN244-p1	27-Sep-11	8	Y	1	S	S	+	UK	SRR3335184
C00013820	3376-p1	03-Apr-12	8	Y	1	S	S	+	UK	SRR3338082
C00012684	3280-p1	14-May-12	8	Y	1	S	S	+	UK	SRR3338159
C00013871	3436-p1	05-Jun-12	8	Y	1	S	S	+	UK	SRR3335452
C00013865	3429-p1	10-Jun-12	8	Y	1	S	S	+	UK	SRR3338693
C00013882	3451-p1	13-Jun-12	8	Y	1	S	S	+	UK	SRR3335238
C00014322	3493-p1	16-Jun-12	8	Y	1	S	S	+	UK	SRR3335267
C00014364	3551-p1	24-Aug-12	8	Y	1	S	S	+	UK	SRR3338079
C00014372	3563-p1	03-Sep-12	8	Y	1	S	S	+	UK	SRR3338512
C00015469	3599-p1	23-Sep-12	8	Y	1	S	S	+	UK	SRR3339345
C00015518	3670-p1	20-Oct-12	8	Y	1	S	S	+	UK	SRR3338536
C00016363	3710-p1	09-Nov-12	8	Y	1	S	S	+	UK	SRR3338269
C00020415	3845-p1	03-Mar-13	8	Y	1	S	S	+	UK	SRR3338569
C00020428	3860-p1	07-Mar-13	8	Y	1	S	S	+	UK	SRR3338717
C00021214	3879-p1	21-Mar-13	8	Y	1	S	S	+	UK	SRR3335608
C00020429	3888-p1	03-Apr-13	8	Y	1	S	S	+	UK	SRR3335281
C00009715	TN97-p1	23-Mar-11	9	N	1	S	S	+	UK	SRR3338503
C00011172	TN107-p1	05-Apr-11	9	N	1	S	S	+	UK	SRR3334936
C00011216	TN210-p1	23-Aug-11	9	N	1	S	S	+	UK	SRR3335819
C00013808	3355-p1	03-Apr-12	9	N	1	S	S	+	UK	SRR3335666
C00020364	3777-p1	19-Dec-12	9	N	1	S	S	+	UK	SRR3338571
C00009967	TN3-p1	05-Dec-10	10	N	1	S	S	+	UK	SRR3335972
C00009958	TN25-p1	18-Jan-11	10	N	1	S	S	+	UK	SRR3335582
C00009924	TN38-p1	26-Jan-11	10	N	1	S	S	+	UK	SRR3334801
C00009723	TN65-p1	21-Feb-11	10	N	1	S	S	+	UK	SRR3338499
C00009739	TN99-p1	27-Mar-11	10	N	1	S	S	+	UK	SRR3338128
C00011176	TN123-p1	30-May-11	10	N	1	S	S	+	UK	SRR3334895
C00009741	TN167-p1	30-Jun-11	10	N	1	S	S	+	UK	SRR3338963

C00009766	TN177-p1	11-Jul-11	10	N	1	S	S	+	UK	SRR3335974
C00009732	TN190-p1	25-Jul-11	10	N	1	S	S	+	UK	SRR3338619
C00009809	TN233-p1	14-Sep-11	10	N	1	S	S	+	UK	SRR3338998
C00009798	TN240-p1	21-Sep-11	10	N	1	S	S	+	UK	SRR3338125
C00009834	TN243-p1	25-Sep-11	10	N	1	S	S	+	UK	SRR3335303
C00013848	3413-p1	01-Jun-12	10	N	1	S	S	+	UK	SRR3335549
C00016350	3690-p1	28-Oct-12	10	N	1	S	S	+	UK	SRR3334891
C00020382	3799-p1	18-Jan-13	10	N	1	S	S	+	UK	SRR3338934
C00020404	3828-p1	05-Feb-13	10	N	1	S	S	+	UK	SRR3335232
C00009909	TN13-p1	05-Jan-11	11	N	5	S	S	+	UK	SRR3339325
C00011249	TN137-p1	07-Jun-11	11	N	5	S	S	+	UK	SRR3338274
C00011190	TN140-p1	12-Jun-11	11	N	5	S	S	+	UK	SRR3335494
C00009821	TN234-p1	16-Sep-11	11	N	5	S	S	+	UK	SRR3339018
C00014299	3339-p1	07-Apr-12	11	N	5	S	S	+	UK	SRR3335266
C00013900	3475-p1	02-Jul-12	11	N	5	S	S	+	UK	SRR3338475
C00015508	3658-p1	15-Oct-12	11	N	5	R	S	+	UK	SRR3338135
C00020108	3730-p1	22-Nov-12	11	N	5	S	S	+	UK	SRR3338198
C00020414	3844-p1	17-Feb-13	11	N	5	S	S	+	UK	SRR3338252
C00009896	TN5-p1	06-Dec-10	12	N	1	S	S	+	UK	SRR3337750
C00011177	TN131-p1	06-Jun-11	12	N	1	S	S	+	UK	SRR3338481
C00009719	TN181-p1	13-Jul-11	12	N	1	S	S	+	UK	SRR3335574
C00009829	TN199-p1	10-Aug-11	12	N	1	S	S	+	UK	SRR3335931
C00009842	TN208-p1	18-Aug-11	12	N	1	S	S	+	UK	SRR3335976
C00015484	3616-p1	19-Sep-12	12	N	1	S	S	+	UK	SRR3335848
C00016346	3686-p1	29-Oct-12	12	N	1	S	S	+	UK	SRR3336099
C00020424	3856-p1	17-Feb-13	12	N	1	S	S	+	UK	SRR3334838
C00009980	TN11-p1	04-Jan-11	13	N	1	S	S	+	UK	SRR3338945
C00009972	TN42-p1	30-Jan-11	13	N	1	S	S	+	UK	SRR3335924
C00011166	TN64-p1	16-Feb-11	13	N	1	S	S	+	UK	SRR3337510
C00009761	TN84-p1	10-Mar-11	13	N	1	S	S	+	UK	SRR3335496

C00011186	TN110-p1	11-Apr-11	13	N	1	S	S	+	UK	SRR3338180
C00009808	TN226-p1	08-Sep-11	13	N	1	S	S	+	UK	SRR3334831
C00013821	3379-p1	11-Apr-12	13	N	1	S	S	+	UK	SRR3336040
C00014327	3500-p1	08-Jul-12	13	N	1	S	S	+	UK	SRR3335183
C00014360	3546-p1	14-Aug-12	13	N	1	S	S	+	UK	SRR3335460
C00009897	TN12-p1	04-Jan-11	14	N	1	S	S	+	UK	SRR3338781
C00009922	TN22-p1	13-Jan-11	14	N	1	S	S	+	UK	SRR3339305
C00009946	TN24-p1	17-Jan-11	14	N	1	S	S	+	UK	SRR3335479
C00011167	TN94-p1	21-Mar-11	14	N	1	S	S	+	UK	SRR3335560
C00011170	TN105-p1	04-Apr-11	14	N	1	S	S	+	UK	SRR3335962
C00011237	TN136-p1	07-Jun-11	14	N	1	S	S	+	UK	SRR3335152
C00011251	TN153-p1	20-Jun-11	14	N	1	S	S	+	UK	SRR3334926
C00009794	TN204-p1	16-Aug-11	14	N	1	S	S	+	UK	SRR3339011
C00009795	TN216-p1	30-Aug-11	14	N	1	S	S	+	UK	SRR3334931
C00009884	TN223-p1	05-Sep-11	14	N	1	S	S	+	UK	SRR3335231
C00009864	TN246-p1	28-Sep-11	14	N	1	S	S	+	UK	SRR3339027
C00012685	3281-p1	04-Apr-12	14	N	1	S	S	+	UK	SRR3338255
C00013826	3385-p1	25-Apr-12	14	N	1	S	S	+	UK	SRR3338773
C00013879	3447-p1	30-May-12	14	N	1	S	S	+	UK	SRR3335991
C00013857	3422-p1	07-Jun-12	14	N	1	S	S	+	UK	SRR3335033
C00016349	3689-p1	29-Oct-12	14	N	1	S	R	+	UK	SRR3337664
C00016362	3708-p1	08-Nov-12	14	N	1	S	S	+	UK	SRR3338682
C00021209	3873-p1	20-Mar-13	14	N	1	S	R	+	UK	SRR3338139
C00009957	TN17-p1	10-Jan-11	15	N	1	S	S	-	UK	SRR3337508
C00009969	TN18-p1	11-Jan-11	15	N	1	S	S	-	UK	SRR3339317
C00009934	TN23-p1	17-Jan-11	15	N	1	S	S	-	UK	SRR3338763
C00009911	TN29-p1	23-Jan-11	15	N	1	S	S	-	UK	SRR3337682
C00009971	TN34-p1	25-Jan-11	15	N	1	S	S	-	UK	SRR3338927
C00009912	TN37-p1	26-Jan-11	15	N	1	S	S	-	UK	SRR3338627
C00009757	TN51-p1	07-Feb-11	15	N	1	S	S	-	UK	SRR3334859

C00009722	TN56-p1	10-Feb-11	15	N	1	S	S	-	UK	SRR3334849
C00009699	TN62-p1	14-Feb-11	15	N	1	S	S	-	UK	SRR3338241
C00009724	TN73-p1	28-Feb-11	15	N	1	S	S	-	UK	SRR3338690
C00009726	TN89-p1	15-Mar-11	15	N	1	S	S	-	UK	SRR3334873
C00009762	TN92-p1	17-Mar-11	15	N	1	S	S	-	UK	SRR3337779
C00009787	TN103-p1	30-Mar-11	15	N	1	S	S	-	UK	SRR3339328
C00011169	TN104-p1	03-Apr-11	15	N	1	S	S	-	UK	SRR3335265
C00011187	TN116-p1	23-May-11	15	N	1	S	S	-	UK	SRR3334840
C00011211	TN118-p1	24-May-11	15	N	1	S	S	-	UK	SRR3334942
C00011213	TN134-p1	07-Jun-11	15	N	1	S	S	-	UK	SRR3338470
C00011191	TN148-p1	16-Jun-11	15	N	1	S	S	-	UK	SRR3334857
C00011239	TN152-p1	20-Jun-11	15	N	1	S	S	-	UK	SRR3338278
C00011180	TN155-p1	22-Jun-11	15	N	1	S	S	-	UK	SRR3338668
C00009753	TN168-p1	02-Jul-11	15	N	1	S	S	-	UK	SRR3338222
C00009742	TN175-p1	08-Jul-11	15	N	1	S	S	-	UK	SRR3338677
C00009796	TN224-p1	06-Sep-11	15	N	1	S	S	-	UK	SRR3335158
C00009820	TN227-p1	12-Sep-11	15	N	1	S	S	-	UK	SRR3335986
C00009869	TN231-p1	14-Sep-11	15	N	1	S	S	-	UK	SRR3337748
C00009460	3253-p1	15-Apr-12	15	N	1	S	S	-	UK	SRR3336063
C00013812	3361-p1	16-Apr-12	15	N	1	S	S	-	UK	SRR3338969
C00012680	3275-p1	18-Apr-12	15	N	1	S	S	-	UK	SRR3338673
C00012689	3285-p1	19-Apr-12	15	N	1	S	S	-	UK	SRR3335497
C00013854	3419-p1	30-May-12	15	N	1	S	S	-	UK	SRR3337717
C00013886	3455-p1	03-Jun-12	15	N	1	S	S	-	UK	SRR3339302
C00013889	3460-p1	14-Jun-12	15	N	1	S	S	-	UK	SRR3338257
C00013899	3474-p1	01-Jul-12	15	N	1	S	S	-	UK	SRR3335966
C00014345	3526-p1	17-Jul-12	15	N	1	S	S	-	UK	SRR3337714
C00014350	3532-p1	25-Jul-12	15	N	1	S	S	-	UK	SRR3334946
C00014336	3514-p1	01-Aug-12	15	N	1	S	S	-	UK	SRR3334815
C00014361	3547-p1	16-Aug-12	15	N	1	S	S	-	UK	SRR3338087
C00015467	3597b-p1	14-Sep-12	15	N	1	S	S	-	UK	SRR3338711

C00015496	3636-p1	27-Sep-12	15	N	1	S	S	-	UK	SRR3338957
C00016357	3700-p1	06-Nov-12	15	N	1	S	S	-	UK	SRR3337762
C00020139	3767-p1	24-Dec-12	15	N	1	S	S	-	UK	SRR3338885
C00020137	3765-p1	03-Jan-13	15	N	1	S	S	-	UK	SRR3334867
C00020132	3760-p1	04-Jan-13	15	N	1	S	S	-	UK	SRR3337686
C00020383	3800-p1	19-Jan-13	15	N	1	S	S	-	UK	SRR3338280
C00020384	3801-p1	22-Jan-13	15	N	1	S	S	-	UK	SRR3338749
C00020241	3892-p1	05-Apr-13	15	N	1	S	S	-	UK	SRR3337658
C00009775	TN102-p1	30-Mar-11	16	N	1	S	S	+	UK	SRR3338630
C00009883	TN203-p1	15-Aug-11	16	N	1	S	S	+	UK	SRR3335207
C00009712	TN72-p1	28-Feb-11	17	N	1	S	S	+	UK	SRR3339300
C00011248	TN129-p1	03-Jun-11	17	N	1	S	S	+	UK	SRR3335180
C00009843	TN220-p1	04-Sep-11	17	N	1	S	S	+	UK	SRR3338730
C00020394	3814-p1	31-Jan-13	17	N	1	S	S	+	UK	SRR3338583
C00011210	TN111-p1	22-May-11	18	N	1	S	S	+	UK	SRR3335447
C00013819	3375-p1	08-May-12	22	N	3	S	S	+	UK	SRR3338728
C00021207	3869-p1	14-Mar-13	24	N	1	S	S	+	UK	SRR3336068
C00009944	TN8-p1	12-Dec-10	26	N	1	S	S	-	UK	SRR3339019
C00009956	TN9-p1	14-Dec-10	26	N	1	S	S	-	UK	SRR3334903
C00009745	TN50-p1	07-Feb-11	26	N	1	S	S	-	UK	SRR3338474
C00009700	TN71-p1	27-Feb-11	26	N	1	S	S	-	UK	SRR3338106
C00009783	TN70-p1	27-Feb-11	26	N	1	S	S	-	UK	SRR3335202
C00009748	TN75-p1	01-Mar-11	26	N	1	S	S	-	UK	SRR3335310
C00009713	TN80-p1	07-Mar-11	26	N	1	S	S	-	UK	SRR3335904
C00011174	TN109-p1	08-Apr-11	26	N	1	S	S	-	UK	SRR3335486
C00011236	TN128-p1	01-Jun-11	26	N	1	S	S	-	UK	SRR3338184
C00011238	TN144-p1	15-Jun-11	26	N	1	S	S	-	UK	SRR3339035
C00011227	TN151-p1	19-Jun-11	26	N	1	S	S	-	UK	SRR3335592
C00009728	TN159-p1	26-Jun-11	26	N	1	S	S	-	UK	SRR3338580
C00009707	TN180-p1	13-Jul-11	26	N	1	S	S	-	UK	SRR3339007

C00009805	TN197-p1	08-Aug-11	26	N	1	S	S	-	UK	SRR3339337
C00012686	3282-p1	11-Apr-12	26	N	1	S	S	-	UK	SRR3338651
C00012666	3258-p1	13-Apr-12	26	N	1	S	S	-	UK	SRR3338723
C00013825	3384-p1	24-Apr-12	26	N	1	S	S	-	UK	SRR3338190
C00012722	3333-p1	14-May-12	26	N	1	S	S	-	UK	SRR3338949
C00013847	3412-p1	01-Jun-12	26	N	1	S	S	-	UK	SRR3335594
C00014320	3491-p1	27-Jun-12	26	N	1	S	S	-	UK	SRR3335562
C00013891	3464-p1	29-Jun-12	26	N	1	S	S	-	UK	SRR3338563
C00014315	3484-p1	03-Jul-12	26	N	1	S	S	-	UK	SRR3338789
C00015454	3582-p1	14-Sep-12	26	N	1	S	S	-	UK	SRR3338971
C00015481	3611-p1	25-Sep-12	26	N	1	S	R	-	UK	SRR3337704
C00015516	3668-p1	19-Oct-12	26	N	1	S	S	-	UK	SRR3334837
C00016361	3706-p1	08-Nov-12	26	N	1	S	S	-	UK	SRR3338734
C00020106	3728-p1	20-Nov-12	26	N	1	S	S	-	UK	SRR3334808
C00020366	3779-p1	02-Jan-13	26	N	1	S	S	-	UK	SRR3338540
C00020373	3788-p1	09-Jan-13	26	N	1	S	S	-	UK	SRR3338793
C00020110	3732-p1	24-Nov-12	28	N	1	S	S	-	UK	SRR3335263
C00020406	3832-p1	12-Feb-13	28	N	1	S	S	-	UK	SRR3335529
C00009955	TN2-p1	04-Dec-10	29	N	1	S	S	-	UK	SRR3335863
C00009818	TN206-p1	16-Aug-11	29	N	1	S	S	-	UK	SRR3334864
C00009749	TN83-p1	10-Mar-11	33	N	1	S	S	+	UK	SRR3339041
C00009751	TN100-p1	28-Mar-11	33	N	1	S	S	+	UK	SRR3338709
C00009841	TN200-p1	11-Aug-11	33	N	1	S	S	+	UK	SRR3338901
C00015459	3588-p1	13-Sep-12	33	N	1	S	S	+	UK	SRR3338735
C00021205	3867-p1	13-Mar-13	34	N	1	R	S	+	UK	SRR3335208
C00011171	TN106-p1	05-Apr-11	35	N	1	S	S	+	UK	SRR3338893
C00009744	TN191-p1	25-Jul-11	35	N	1	S	S	+	UK	SRR3334898
C00009768	TN193-p1	31-Jul-11	35	N	1	S	S	+	UK	SRR3336058
C00009868	TN215-p1	30-Aug-11	35	N	1	S	S	+	UK	SRR3334912
C00013817	3372-p1	08-May-12	35	N	1	S	S	+	UK	SRR3335939

C00015462	3592-p1	20-Sep-12	35	N	1	R	S	+	UK	SRR3337769
C00020362	3775-p1	25-Dec-12	35	N	1	S	S	+	UK	SRR3336032
C00021208	3872-p1	19-Mar-13	35	N	1	S	S	+	UK	SRR3335871
C00009863	TN230-p1	14-Sep-11	36	N	1	S	S	+	UK	SRR3338084
C00011175	TN115-p1	23-May-11	37	Y	4	S	S	+	UK	SRR3337771
C00016355	3697-p1	05-Nov-12	37	Y	4	S	S	+	UK	SRR3338720
C00009735	TN66-p1	21-Feb-11	39	N	4	S	S	-	UK	ERS243555
C00009725	TN81-p1	09-Mar-11	39	N	4	S	S	-	UK	ERS243554
C00016367	3714-p1	13-Nov-12	39	N	4	S	S	-	UK	SRR3335888
C00009737	TN82-p1	09-Mar-11	42	N	1	R	S	+	UK	SRR3338986
C00013852	3417-p1	31-May-12	42	N	1	R	S	+	UK	SRR3339395
C00015488	3624-p1	23-Sep-12	42	N	1	S	S	+	UK	SRR3335298
C00009885	TN239-p1	20-Sep-11	43	N	1	S	S	+	UK	SRR3335903
C00015502	3649-p1	09-Oct-12	43	N	1	S	S	+	UK	SRR3338933
C00020388	3805-p1	28-Jan-13	43	N	1	S	S	+	UK	SRR3335467
C00009746	TN58-p1	13-Feb-11	44	N	1	S	S	+	UK	SRR3336097
C00011188	TN124-p1	30-May-11	44	N	1	S	S	+	UK	SRR3335472
C00011224	TN127-p1	01-Jun-11	44	N	1	S	S	+	UK	SRR3335604
C00011263	TN154-p1	21-Jun-11	44	N	1	S	S	+	UK	SRR3335538
C00009740	TN160-p1	27-Jun-11	44	N	1	S	S	+	UK	SRR3339016
C00009777	TN170-p1	03-Jul-11	44	N	1	S	S	+	UK	SRR3335492
C00009767	TN185-p1	19-Jul-11	44	N	1	S	S	+	UK	SRR3335443
C00009817	TN198-p1	10-Aug-11	44	N	1	S	S	+	UK	SRR3335573
C00009807	TN217-p1	30-Aug-11	44	N	1	S	S	+	UK	SRR3338144
C00009832	TN228-p1	12-Sep-11	44	N	1	S	S	+	UK	SRR3335477
C00009822	TN242-p1	22-Sep-11	44	N	1	S	S	+	UK	SRR3335516
C00009459	3252-p1	11-Apr-12	44	N	1	S	S	+	UK	SRR3334888
C00013813	3364-p1	23-Apr-12	44	N	1	S	S	+	UK	SRR3335540
C00014333	3508-p1	17-Jul-12	44	N	1	S	S	+	UK	SRR3335967
C00015457	3585-p1	07-Sep-12	44	N	1	S	S	+	UK	SRR3338199

C00015479	3609-p1	25-Sep-12	44	N	1	S	S	+	UK	SRR3335906
C00016364	3711-p1	11-Nov-12	44	N	1	S	S	+	UK	SRR3335564
C00020120	3748-p1	05-Dec-12	44	N	1	S	S	+	UK	SRR3335499
C00011261	TN138-p1	07-Jun-11	45	N	1	S	S	+	UK	SRR3336047
C00016360	3703-p1	07-Nov-12	45	N	1	R	S	+	UK	SRR3334824
C00020131	3759-p1	01-Jan-13	45	N	1	S	S	+	UK	SRR3335895
C00009709	TN47-p1	03-Feb-11	46	N	1	S	S	+	UK	SRR3335874
C00014359	3545-p1	14-Aug-12	46	N	1	S	S	+	UK	SRR3335994
C00009908	TN6-p1	07-Dec-10	48	N	1	R	S	-	UK	SRR3339353
C00011189	TN132-p1	06-Jun-11	48	N	1	S	S	-	UK	SRR3335610
C00009756	TN192-p1	27-Jul-11	48	N	1	S	R	+	UK	SRR3335316
C00009831	TN219-p1	03-Sep-11	48	N	1	S	S	-	UK	SRR3335123
C00014348	3529-p1	23-Jul-12	48	N	1	S	S	-	UK	SRR3338636
C00009979	TN4-p1	06-Dec-10	49	N	1	S	S	+	UK	SRR3338103
C00009898	TN20-p1	13-Jan-11	49	N	1	S	S	+	UK	SRR3337667
C00009910	TN21-p1	13-Jan-11	49	N	1	S	S	+	UK	SRR3338972
C00009948	TN40-p1	27-Jan-11	49	N	1	S	S	+	UK	SRR3338990
C00009769	TN52-p1	07-Feb-11	49	N	1	S	S	+	UK	SRR3335905
C00012732	3348-p1	22-May-12	49	N	1	S	S	+	UK	SRR3339327
C00013859	3424-p1	25-May-12	49	N	1	S	S	+	UK	SRR3335505
C00015451	3576-p1	06-Sep-12	49	N	1	S	S	+	UK	SRR3335149
C00015477	3606b-p1	17-Sep-12	49	N	1	S	S	+	UK	SRR3339012
C00015483	3614-p1	18-Sep-12	49	N	1	S	S	+	UK	SRR3338581
C00015455	3583-p1	20-Sep-12	49	N	1	S	S	+	UK	SRR3338624
C00015493	3632-p1	27-Sep-12	49	N	1	S	S	+	UK	SRR3335970
C00012672	3266-p1	10-May-12	51	N	1	S	S	+	UK	SRR3334861
C00013841	3405-p1	28-Apr-12	54	N	1	S	S	+	UK	SRR3338991
C00014329	3504-p1	20-Jul-12	54	N	1	S	S	+	UK	SRR3336035
C00014342	3522-p1	27-Jul-12	54	N	1	S	S	+	UK	SRR3338070
C00014337	3515-p1	02-Aug-12	54	N	1	S	S	+	UK	SRR3335491

C00015482	3612-p1	12-Sep-12	54	N	1	S	S	+	UK	SRR3335155
C00020408	3836-p1	13-Feb-13	54	N	1	S	S	+	UK	SRR3338582
C00009708	TN188-p1	20-Jul-11	55	N	1	S	S	+	UK	SRR3337790
C00009858	TN245-p1	27-Sep-11	55	N	1	S	S	+	UK	SRR3337775
C00014354	3538-p1	09-Aug-12	55	N	1	S	S	+	UK	SRR3334941
C00009738	TN90-p1	15-Mar-11	58	N	1	S	S	+	UK	SRR3336051
C00011260	TN130-p1	05-Jun-11	58	N	1	S	S	+	UK	SRR3338235
C00012669	3262-p1	04-May-12	58	N	1	S	S	+	UK	SRR3334910
C00020111	3733-p1	26-Nov-12	58	N	1	S	S	+	UK	SRR3338081
C00009959	TN33-p1	24-Jan-11	75	N	1	S	S	+	UK	SRR3334847
C00013840	3404-p1	21-Apr-12	75	N	1	S	S	+	UK	SRR3337594
C00009770	TN60-p1	14-Feb-11	92	N	1	S	S	+	UK	SRR3338516
C00016345	3684-p1	27-Oct-12	92	N	1	S	S	+	UK	SRR3335483
C00009960	TN41-p1	27-Jan-11	101	N	1	S	S	-	UK	SRR3338643
C00016356	3698-p1	06-Nov-12	103	N	1	S	S	+	UK	SRR3338920
C00009788	TN163-p1	27-Jun-11	107	N	1	S	S	-	UK	SRR3334809
C00014366	3553-p1	31-Aug-12	107	N	1	S	S	-	UK	SRR3336102
C00020359	3556-p1	17-Aug-12	109	Y	4	S	S	-	UK	SRR3338610
C00009920	TN7-p1	07-Dec-10	124	N	4	S	S	-	UK	SRR3338374
C00009899	TN28-p1	19-Jan-11	125	N	1	S	S	-	UK	SRR3335791
C00011223	TN119-p1	24-May-11	125	N	1	S	S	-	UK	SRR3337792
C00013877	3443-p1	08-Jun-12	125	N	1	S	S	-	UK	SRR3338663
C00012713	3323-p1	06-May-12	139	N	1	S	S	+	UK	SRR3335434
C00009778	TN178-p1	11-Jul-11	205	N	1	S	S	-	UK	SRR3337773
C00013839	3402-p1	22-Apr-12	240	N	1	S	S	-	UK	SRR3338232
C00016340	3678-p1	26-Oct-12	240	N	1	S	S	-	UK	SRR3338633
C00014313	3478-p1	22-Jun-12	241	N	4	S	S	-	UK	SRR3336036
C00015471	3601-p1	22-Sep-12	242	N	1	S	S	-	UK	SRR3338151

C00011235	TN120-p1	24-May-11	243	N	4	S	S	-	UK	SRR3337718
C00009833	TN235-p1	19-Sep-11	250	N	1	S	S	-	UK	SRR3338157
C00009806	TN205-p1	16-Aug-11	251	N	1	S	S	+	UK	SRR3338094
C00013876	3441-p1	12-Jun-12	256	N	1	S	S	+	UK	SRR3335203
C00013845	3410-p1	01-Jun-12	266	N	4	S	S	-	UK	SRR3335192
C00014338	3517-p1	14-Jul-12	267	N	1	S	S	-	UK	SRR3335572
C00013860	3425-p1	27-May-12	270	N	1	S	S	+	UK	SRR3337703
C00009729	TN166-p1	29-Jun-11	272	N	1	S	S	-	UK	SRR3338939
C00009786	TN95-p1	22-Mar-11	273	N	4	S	S	-	UK	SRR3335248

(d) Optimer Fidaxomicin Trial Human Clinical Isolates

Oxfordshire clinical trial isolates (n=27) were excluded to avoid duplication.

Genome Identification Number	Isolate Name	Isolation Date	ST	Phylogeny	clade	*gyrA*	*gyrB*	PaLoc	Country	City	SRA Accession
C00010913	OPT_2250	01-May-09	1	N	2	R	S	+	Belgium	Columbia	ERS352265
C00020804	OPT_2568	11-Sep-09	1	N	2	R	S	+	Belgium	Gent	SRR3335439
C00020925	OPT_2687	14-Oct-08	1	N	2	S	S	+	Belgium	Kortrijk	SRR3334897
C00020499	OPT_2074	20-Dec-07	1	N	2	R	S	+	Belgium	LiÞge	SRR3338083
C00020549	OPT_2345	30-Jan-08	1	N	2	R	S	+	Canada	Ajax	SRR3335278
C00021112	OPT_2861	09-Apr-08	1	N	2	R	S	+	Canada	Ajax	SRR3335568
C00010975	OPT_2241	12-Apr-09	1	N	2	R	R	+	Canada	Calgary	ERS352278
C00010983	OPT_2279	06-Nov-06	1	N	2	S	S	+	Canada	Calgary	ERS352260
C00020244	OPT_1683	07-Jun-07	1	N	2	S	S	+	Canada	Calgary	SRR3338479
C00020247	OPT_1687	20-Aug-07	1	N	2	S	S	+	Canada	Calgary	SRR3338121
C00020527	OPT_2324	30-Jan-08	1	N	2	S	S	+	Canada	Calgary	SRR3337741
C00020733	OPT_2499	21-Apr-09	1	N	2	R	S	+	Canada	Calgary	SRR3334880
C00020756	OPT_2522	22-Sep-09	1	N	2	S	S	+	Canada	Calgary	SRR3338764
C00020968	OPT_2730	07-Nov-06	1	N	2	S	S	+	Canada	Calgary	SRR3336049
C00021017	OPT_2778	15-Mar-07	1	N	2	S	S	+	Canada	Calgary	SRR3336074
C00021084	OPT_2833	11-Aug-07	1	N	2	S	S	+	Canada	Calgary	SRR3339397
C00028649	OPT_2754	31-Jan-07	1	N	2	S	S	+	Canada	Calgary	SRR3335284
C00028656	OPT_2832	10-Aug-07	1	N	2	S	S	+	Canada	Calgary	SRR3335481
C00010923	OPT_1953	26-Sep-08	1	N	2	R	S	+	Canada	Chicoutimi	ERS352262
C00010925	OPT_1954	03-Oct-08	1	N	2	R	S	+	Canada	Chicoutimi	ERS352269
C00010929	OPT_2238	02-Mar-09	1	N	2	S	S	+	Canada	Chicoutimi	ERS352253
C00020711	OPT_2477	24-Mar-09	1	N	2	R	S	+	Canada	Chicoutimi	SRR3335938
C00020816	OPT_2580	06-Nov-08	1	N	2	R	S	+	Canada	Chicoutimi	SRR3338999
C00020753	OPT_2519	29-Sep-09	1	N	2	R	S	+	Canada	Edmonton	SRR3338983
C00010915	OPT_2044	11-Sep-08	1	N	2	R	S	+	Canada	Hamilton	ERS352280

C00010918	OPT_2234	03-Mar-09	1	N	2	R	S	+	Canada	Hamilton	ERS352273
C00010920	OPT_2235	09-Mar-09	1	N	2	R	S	+	Canada	Hamilton	ERS352285
C00011072	OPT_1846	14-Jul-08	1	N	2	R	S	+	Canada	Hamilton	ERS352271
C00020310	OPT_1790	15-Jul-08	1	N	2	R	S	+	Canada	Hamilton	SRR3335537
C00020699	OPT_2465	10-Mar-09	1	N	2	R	R	+	Canada	Hamilton	SRR3336095
C00020710	OPT_2476	08-Apr-09	1	N	2	R	S	+	Canada	Hamilton	SRR3335228
C00020900	OPT_2662	24-Sep-08	1	N	2	R	S	+	Canada	Hamilton	SRR3338078
C00020928	OPT_2690	24-Nov-08	1	N	2	R	S	+	Canada	Hamilton	SRR3338898
C00020929	OPT_2691	30-Nov-08	1	N	2	R	S	+	Canada	Hamilton	SRR3335951
C00010935	OPT_2060	30-Oct-08	1	Y	2	R	S	+	Canada	Montreal	ERS352270
C00010936	OPT_2076	20-Jan-09	1	Y	2	R	S	+	Canada	Montreal	ERS352276
C00010953	OPT_2082	20-Jan-09	1	Y	2	R	S	+	Canada	Montreal	ERS352283
C00016335	OPT-1877	27-May-08	1	Y	2	R	S	+	Canada	Montreal	ERS352288
C00020277	OPT_1721	19-Sep-07	1	Y	2	R	S	+	Canada	Montreal	SRR3335598
C00020289	OPT_1737	28-Sep-07	1	Y	2	R	S	+	Canada	Montreal	SRR3338511
C00020291	OPT_1739	01-Oct-07	1	Y	2	R	S	+	Canada	Montreal	SRR3334851
C00020292	OPT_1740	15-Apr-08	1	Y	2	R	S	+	Canada	Montreal	SRR3335902
C00020305	OPT_1783	03-Dec-07	1	Y	2	R	S	+	Canada	Montreal	SRR3335885
C00020309	OPT_1788	17-Jun-08	1	Y	2	R	S	+	Canada	Montreal	SRR3338638
C00020312	OPT_1793	07-Feb-08	1	Y	2	R	S	+	Canada	Montreal	SRR3334833
C00020315	OPT_1802	21-Nov-07	1	Y	2	R	S	+	Canada	Montreal	SRR3335876
C00020318	OPT_1805	12-Dec-07	1	Y	2	R	S	+	Canada	Montreal	SRR3338545
C00020319	OPT_1806	04-Jan-08	1	Y	2	R	S	+	Canada	Montreal	SRR3338965
C00020322	OPT_1809	23-Apr-08	1	Y	2	R	S	+	Canada	Montreal	SRR3335901
C00020323	OPT_1810	08-Jun-08	1	Y	2	R	S	+	Canada	Montreal	SRR3338691
C00020327	OPT_1815	10-Oct-07	1	Y	2	R	S	+	Canada	Montreal	SRR3337733
C00020328	OPT_1816	22-Oct-07	1	Y	2	R	S	+	Canada	Montreal	SRR3335455
C00020329	OPT_1817	20-Feb-08	1	Y	2	R	S	+	Canada	Montreal	SRR3339332
C00020330	OPT_1818	01-Apr-08	1	Y	2	R	S	+	Canada	Montreal	SRR3339037
C00020331	OPT_1819	10-Jun-08	1	Y	2	R	S	+	Canada	Montreal	SRR3338755
C00020332	OPT_1820	15-Jul-08	1	Y	2	R	S	+	Canada	Montreal	SRR3338462

C00020437	OPT_1839	31-Jan-08	1	Y	2	R	S	+	Canada	Montreal	SRR3338722
C00020439	OPT_1840	23-Oct-07	1	Y	2	R	S	+	Canada	Montreal	SRR3335237
C00020441	OPT_1855	25-Feb-08	1	Y	2	R	S	+	Canada	Montreal	SRR3337786
C00020482	OPT_1939	27-May-09	1	Y	2	R	S	+	Canada	Montreal	SRR3338476
C00020494	OPT_2048	26-Jan-09	1	Y	2	R	S	+	Canada	Montreal	SRR3337759
C00020496	OPT_2054	20-Aug-08	1	Y	2	R	S	+	Canada	Montreal	SRR3338132
C00020500	OPT_2078	09-Jan-09	1	Y	2	R	S	+	Canada	Montreal	SRR3338528
C00020508	OPT_2268	28-Jul-09	1	Y	2	R	S	+	Canada	Montreal	SRR3337668
C00020535	OPT_2331	14-Jan-08	1	Y	2	R	S	+	Canada	Montreal	SRR3334887
C00020546	OPT_2342	09-Jan-08	1	Y	2	R	S	+	Canada	Montreal	SRR3335983
C00020554	OPT_2349	12-Feb-08	1	Y	2	R	S	+	Canada	Montreal	SRR3338236
C00020573	OPT_2368	19-Nov-07	1	Y	2	R	S	+	Canada	Montreal	SRR3334911
C00020596	OPT_2391	23-Jun-08	1	Y	2	R	S	+	Canada	Montreal	SRR3336037
C00020605	OPT_2400	14-Jun-08	1	Y	2	R	S	+	Canada	Montreal	SRR3336000
C00020611	OPT_2406	14-Jul-08	1	Y	2	R	S	+	Canada	Montreal	SRR3338202
C00020705	OPT_2471	29-Jan-09	1	Y	2	R	S	+	Canada	Montreal	SRR3338465
C00020708	OPT_2474	23-Feb-09	1	Y	2	R	S	+	Canada	Montreal	SRR3335844
C00020709	OPT_2475	20-Feb-09	1	Y	2	R	S	+	Canada	Montreal	SRR3334817
C00020713	OPT_2479	24-Feb-09	1	Y	2	R	S	+	Canada	Montreal	SRR3335815
C00020714	OPT_2480	25-Mar-09	1	Y	2	R	S	+	Canada	Montreal	SRR3338223
C00020715	OPT_2481	03-Apr-09	1	Y	2	R	S	+	Canada	Montreal	SRR3337684
C00020716	OPT_2482	06-Apr-09	1	Y	2	R	S	+	Canada	Montreal	SRR3335544
C00020729	OPT_2495	02-Mar-09	1	Y	2	R	S	+	Canada	Montreal	SRR3334922
C00020762	OPT_2528	13-Nov-08	1	Y	2	R	S	+	Canada	Montreal	SRR3336093
C00020764	OPT_2530	08-Apr-09	1	Y	2	R	S	+	Canada	Montreal	SRR3338635
C00020776	OPT_2541	24-Apr-09	1	Y	2	R	S	+	Canada	Montreal	SRR3338170
C00020819	OPT_2583	25-Nov-08	1	Y	2	R	S	+	Canada	Montreal	SRR3338171
C00020820	OPT_2584	04-Dec-08	1	Y	2	R	S	+	Canada	Montreal	SRR3339371
C00020833	OPT_2597	03-Dec-08	1	Y	2	R	S	+	Canada	Montreal	SRR3338590
C00020834	OPT_2598	06-Dec-08	1	Y	2	R	S	+	Canada	Montreal	SRR3334904
C00020854	OPT_2617	16-Jan-09	1	Y	2	R	S	+	Canada	Montreal	SRR3335144

C00020855	OPT_2618	02-Feb-09	1	Y	2	R	S	+	Canada	Montreal	SRR3338979
C00020861	OPT_2624	23-Jan-09	1	Y	2	R	S	+	Canada	Montreal	SRR3335291
C00020901	OPT_2663	09-Sep-08	1	Y	2	R	S	+	Canada	Montreal	SRR3338538
C00020905	OPT_2667	30-Sep-08	1	Y	2	R	S	+	Canada	Montreal	SRR3335523
C00020911	OPT_2673	25-Aug-08	1	Y	2	R	S	+	Canada	Montreal	SRR3335431
C00020912	OPT_2674	29-Aug-08	1	Y	2	R	S	+	Canada	Montreal	SRR3338940
C00020914	OPT_2676	14-Oct-08	1	Y	2	R	S	+	Canada	Montreal	SRR3338108
C00020915	OPT_2677	29-Oct-08	1	Y	2	R	S	+	Canada	Montreal	SRR3334826
C00020916	OPT_2678	04-Nov-08	1	Y	2	R	S	+	Canada	Montreal	SRR3339368
C00020917	OPT_2679	11-Nov-08	1	Y	2	R	S	+	Canada	Montreal	SRR3334893
C00020948	OPT_2711	30-Aug-06	1	Y	2	R	S	+	Canada	Montreal	SRR3335224
C00021053	OPT_2803	13-Apr-07	1	Y	2	R	S	+	Canada	Montreal	SRR3334868
C00021054	OPT_2804	19-Apr-07	1	Y	2	R	S	+	Canada	Montreal	SRR3338752
C00021055	OPT_2805	23-Apr-07	1	Y	2	R	S	+	Canada	Montreal	SRR3339038
C00021056	OPT_2806	01-May-07	1	Y	2	R	S	+	Canada	Montreal	SRR3338724
C00021108	OPT_2857	01-Apr-08	1	Y	2	R	S	+	Canada	Montreal	SRR3337791
C00021109	OPT_2858	25-Jun-08	1	Y	2	R	S	+	Canada	Montreal	SRR3337689
C00011077	OPT_1847	04-Jul-08	1	N	2	S	S	+	Canada	Saskatoon	ERS352389
C00020430	OPT_1824	11-Jul-08	1	N	2	S	S	+	Canada	Saskatoon	SRR3335867
C00020892	OPT_2654	03-Jul-08	1	N	2	R	S	+	Canada	St-jerome	SRR3337760
C00010939	OPT_1950	09-Sep-08	1	N	2	R	R	+	Canada	Toronto	ERS352256
C00011066	OPT_2066	17-Jan-08	1	N	2	R	R	+	Canada	Toronto	ERS352254
C00011068	OPT_1864	26-Mar-08	1	N	2	R	S	+	Canada	Toronto	ERS352259
C00011070	OPT_1787	17-Jun-08	1	N	2	R	R	+	Canada	Toronto	ERS352266
C00020259	OPT_1698	07-Aug-07	1	N	2	R	S	+	Canada	Toronto	SRR3338987
C00020304	OPT_1782	02-Oct-07	1	N	2	R	S	+	Canada	Toronto	SRR3335856
C00020306	OPT_1784	26-Jan-08	1	N	2	R	S	+	Canada	Toronto	SRR3337799
C00020325	OPT_1813	29-Mar-08	1	N	2	R	S	+	Canada	Toronto	SRR3334852
C00020326	OPT_1814	27-Mar-08	1	N	2	R	S	+	Canada	Toronto	SRR3337577
C00020540	OPT_2336	09-Dec-07	1	N	2	R	S	+	Canada	Toronto	SRR3338242
C00020588	OPT_2383	25-Mar-08	1	N	2	R	S	+	Canada	Toronto	SRR3337728

C00020599	OPT_2394	15-Jul-08	1	N	2	R	S	+	Canada	Toronto	SRR3338156
C00020652	OPT_2420	09-Feb-08	1	N	2	R	S	+	Canada	Toronto	SRR3338486
C00020702	OPT_2468	05-Mar-09	1	N	2	R	S	+	Canada	Toronto	SRR3225358
C00020822	OPT_2586	26-Nov-08	1	N	2	R	S	+	Canada	Toronto	SRR3225362
C00020856	OPT_2619	26-Jan-09	1	N	2	R	R	+	Canada	Toronto	SRR3225365
C00020897	OPT_2659	22-Aug-08	1	N	2	S	S	+	Canada	Toronto	SRR3225360
C00020907	OPT_2669	24-Sep-08	1	N	2	R	S	+	Canada	Toronto	SRR3225357
C00020919	OPT_2681	07-Oct-08	1	N	2	R	R	+	Canada	Toronto	SRR3225361
C00021094	OPT_2842	17-Jul-07	1	N	2	R	S	+	Canada	Toronto	SRR3225359
C00010959	OPT_2232	19-Feb-09	1	N	2	S	S	+	Canada	Trois-Rivieres	ERS352287
C00010961	OPT_1963	11-Sep-09	1	N	2	R	S	+	Canada	Trois-Rivieres	ERS352286
C00020717	OPT_2483	03-Apr-09	1	N	2	R	S	+	Canada	Trois-Rivieres	SRR3225356
C00020718	OPT_2484	08-Apr-09	1	N	2	R	S	+	Canada	Trois-Rivieres	SRR3225363
C00020769	OPT_2535	11-Jun-09	1	N	2	R	S	+	Canada	Trois-Rivieres	SRR3225364
C00020807	OPT_2571	16-Sep-09	1	N	2	S	S	+	Canada	Trois-Rivieres	SRR3334379
C00020447	OPT_1881	02-Jun-08	1	N	2	R	S	+	Canada	Vancouver	SRR3334002
C00020562	OPT_2357	05-Mar-08	1	N	2	R	S	+	Canada	Vancouver	SRR3334030
C00020299	OPT_1747	28-May-08	1	N	2	R	S	+	Canada	Windsor	SRR3333629
C00020470	OPT_1924	11-Aug-07	1	N	2	R	S	+	Canada	Windsor	SRR3340034
C00020545	OPT_2341	12-Feb-08	1	N	2	R	S	+	Canada	Windsor	SRR3340190
C00020664	OPT_2432	16-Oct-07	1	N	2	R	S	+	UK	Brighton	SRR3334045
C00020487	OPT_2034	13-May-08	1	N	2	R	S	+	UK	Nottingham	SRR3340197
C00020617	OPT_2412	04-Dec-07	1	N	2	R	S	+	UK	Nottingham	SRR3333146
C00010969	OPT_2259	03-Aug-09	1	N	2	R	S	+	USA	Akron	ERS352264
C00011033	OPT_1914	26-Feb-07	1	N	2	R	S	+	USA	Akron	ERS352281
C00020593	OPT_2388	04-Jun-08	1	N	2	S	S	+	USA	Akron	SRR3333114
C00020959	OPT_2721	11-Aug-06	1	N	2	R	S	+	USA	Akron	SRR3333596
C00021024	OPT_2785	30-Mar-07	1	N	2	R	S	+	USA	Akron	SRR3334091
C00020530	OPT_2326	07-Dec-07	1	N	2	R	S	+	USA	Albany	SRR3333067
C00020586	OPT_2381	24-Mar-08	1	N	2	R	S	+	USA	Albany	SRR3333093
C00020556	OPT_2351	19-Mar-08	1	N	2	R	S	+	USA	Allentown	SRR3340226

C00028639	OPT_1960	30-Jul-09	1	N	2	R	S	+	USA	Baltimore	SRR3340059
C00020456	OPT_1900	07-Sep-06	1	N	2	R	S	+	USA	Boston	SRR3340090
C00020457	OPT_1901	29-Sep-06	1	N	2	R	S	+	USA	Boston	SRR3340262
C00021022	OPT_2783	22-Mar-07	1	N	2	R	S	+	USA	Boston	SRR3333798
C00020477	OPT_1931	01-May-09	1	N	2	R	S	+	USA	Bronx	SRR3340018
C00020803	OPT_2567	09-Jul-09	1	N	2	R	S	+	USA	Bronx	SRR3334112
C00011012	OPT_1906	06-Dec-06	1	N	2	R	S	+	USA	Buffalo	ERS352261
C00021030	OPT_2792	26-Apr-07	1	N	2	R	S	+	USA	Buffalo	SRR3333045
C00021078	OPT_2827	11-May-07	1	N	2	R	S	+	USA	Buffalo	SRR3339995
C00020525	OPT_2322	12-Dec-07	1	N	2	R	S	+	USA	Camden	SRR3340030
C00020313	OPT_1795	01-May-08	1	N	2	R	S	+	USA	Charlottesville	SRR3333095
C00020529	OPT_2325	10-Jan-08	1	N	2	R	S	+	USA	Charlottesville	SRR3333292
C00010942	OPT_2046	16-Sep-08	1	N	2	R	S	+	USA	Chicago	ERS352268
C00020317	OPT_1804	30-Jan-08	1	N	2	R	S	+	USA	Chicago	SRR3333550
C00020465	OPT_1917	15-May-07	1	N	2	R	S	+	USA	Chicago	SRR3332998
C00020466	OPT_1918	17-May-07	1	N	2	R	S	+	USA	Chicago	SRR3333723
C00020478	OPT_1932	15-May-09	1	N	2	R	S	+	USA	Chicago	SRR3334051
C00020726	OPT_2492	22-Apr-09	1	N	2	R	R	+	USA	Chicago	SRR3340029
C00020909	OPT_2671	29-Sep-08	1	N	2	R	S	+	USA	Chicago	SRR3340188
C00020952	OPT_2714	23-Aug-06	1	N	2	R	R	+	USA	Chicago	SRR3334226
C00020997	OPT_2758	17-Jan-07	1	N	2	R	S	+	USA	Chicago	SRR3333046
C00021009	OPT_2770	09-Feb-07	1	N	2	R	S	+	USA	Chicago	SRR3333000
C00021031	OPT_2793	20-Apr-07	1	N	2	R	S	+	USA	Chicago	SRR3333751
C00021086	OPT_2834	30-Jul-07	1	N	2	R	S	+	USA	Chicago	SRR3334443
C00021087	OPT_2835	30-Jul-07	1	N	2	R	S	+	USA	Chicago	SRR3332962
C00020308	OPT_1786	04-Mar-08	1	N	2	R	R	+	USA	Clearwater	SRR3333364
C00020932	OPT_2694	04-Feb-08	1	N	2	R	S	+	USA	Clearwater	SRR3334177
C00020461	OPT_1908	05-Jan-07	1	N	2	R	S	+	USA	Cleveland	SRR3332946
C00020949	OPT_2712	24-Aug-06	1	N	2	R	R	+	USA	Cleveland	SRR3334161
C00020993	OPT_2755	10-Jan-07	1	N	2	R	S	+	USA	Cleveland	SRR3333580
C00011038	OPT_2296	07-Feb-07	1	N	2	R	S	+	USA	Columbus	ERS352257

C00020459	OPT_1905	10-Nov-06	1	N	2	R	S	+	USA	Columbus	SRR3333659
C00020823	OPT_2587	02-Dec-08	1	N	2	R	S	+	USA	Columbus	SRR3333048
C00020874	OPT_2637	20-Jan-09	1	N	2	R	S	+	USA	Columbus	SRR3340056
C00020908	OPT_2670	02-Oct-08	1	N	2	R	S	+	USA	Columbus	SRR3333331
C00020982	OPT_2744	20-Dec-06	1	N	2	R	S	+	USA	Columbus	SRR3334199
C00021013	OPT_2774	05-Feb-07	1	N	2	R	S	+	USA	Columbus	SRR3334179
C00021066	OPT_2816	04-May-07	1	N	2	R	S	+	USA	Columbus	SRR3334034
C00020293	OPT_1741	14-May-08	1	N	2	R	S	+	USA	Dayton	SRR3334140
C00020667	OPT_2435	18-Dec-07	1	N	2	R	S	+	USA	Dayton	SRR3333248
C00020850	OPT_2613	18-Feb-09	1	N	2	R	S	+	USA	Dayton	SRR3333227
C00020334	OPT_1822	17-Mar-08	1	N	2	R	S	+	USA	Decatur	SRR3334190
C00020443	OPT_1862	08-Feb-08	1	N	2	R	S	+	USA	Decatur	SRR3340070
C00020589	OPT_2384	18-Apr-08	1	N	2	R	S	+	USA	Decatur	SRR3333409
C00011050	OPT_1794	23-Apr-08	1	N	2	R	S	+	USA	Detroit	ERS352282
C00020521	OPT_2318	30-Nov-07	1	N	2	R	S	+	USA	Detroit	SRR3333226
C00021026	OPT_2787	21-Mar-07	1	N	2	R	S	+	USA	Detroit	SRR3334125
C00020274	OPT_1718	02-Oct-07	1	N	2	R	S	+	USA	Houston	SRR3334160
C00020324	OPT_1811	15-Feb-08	1	N	2	R	S	+	USA	Houston	SRR3340005
C00020531	OPT_2327	13-Dec-07	1	N	2	R	S	+	USA	Jena	SRR3340055
C00020868	OPT_2631	12-Feb-09	1	N	2	R	S	+	USA	Jena	SRR3334407
C00021093	OPT_2841	25-Jul-07	1	N	2	R	S	+	USA	Jena	SRR3333325
C00020303	OPT_1781	27-Nov-07	1	N	2	R	S	+	USA	Keego Harbor	SRR3332950
C00020797	OPT_2561	03-Apr-09	1	N	2	R	S	+	USA	Keego Harbor	SRR3333815
C00020858	OPT_2621	14-Dec-08	1	N	2	R	S	+	USA	Keego Harbor	SRR3333812
C00020458	OPT_1902	31-Aug-06	1	N	2	R	R	+	USA	Lakewood	SRR3339989
C00020460	OPT_1907	18-Dec-06	1	N	2	R	S	+	USA	Lakewood	SRR3334431
C00020467	OPT_1919	09-May-07	1	N	2	R	S	+	USA	Lakewood	SRR3333436
C00020515	OPT_2312	28-Feb-07	1	N	2	R	S	+	USA	Lakewood	SRR3340031
C00020972	OPT_2734	08-Dec-06	1	N	2	R	S	+	USA	Lakewood	SRR3333556
C00021064	OPT_2814	10-May-07	1	N	2	R	S	+	USA	Lakewood	SRR3334174
C00021065	OPT_2815	01-Jun-07	1	N	2	R	S	+	USA	Lakewood	SRR3340255

C00021079	OPT_2828	18-Jun-07	1	N	2	R	S	+	USA	Lakewood	SRR3334065
C00020272	OPT_1715	17-Sep-07	1	N	2	R	S	+	USA	Long Beach	SRR3333435
C00021044	OPT_2794	20-Apr-07	1	N	2	R	S	+	USA	Long Beach	SRR3333552
C00010955	OPT_1958	14-Oct-08	1	N	2	R	S	+	USA	Marietta	ERS352274
C00011021	OPT_1897	13-Jun-06	1	N	2	R	S	+	USA	Marietta	ERS352258
C00011025	OPT_1848	13-Apr-08	1	N	2	R	S	+	USA	Marietta	ERS352388
C00011026	OPT_1796	01-May-08	1	N	2	R	S	+	USA	Marietta	ERS352275
C00020242	OPT_1678	23-Nov-06	1	N	2	R	S	+	USA	Marietta	SRR3333663
C00020314	OPT_1801	16-Jul-07	1	N	2	R	S	+	USA	Marietta	SRR3333309
C00020316	OPT_1803	16-Nov-07	1	N	2	R	S	+	USA	Marietta	SRR3334180
C00020510	OPT_2307	12-Feb-08	1	N	2	R	S	+	USA	Marietta	SRR3339957
C00020559	OPT_2354	19-Nov-07	1	N	2	R	S	+	USA	Marietta	SRR3333255
C00020569	OPT_2364	11-Jan-08	1	N	2	R	S	+	USA	Marietta	SRR3333808
C00020863	OPT_2626	17-Dec-08	1	N	2	R	S	+	USA	Marietta	SRR3333381
C00020942	OPT_2705	31-Jul-06	1	N	2	R	S	+	USA	Marietta	SRR3333990
C00020954	OPT_2716	31-Aug-06	1	N	2	R	S	+	USA	Marietta	SRR3334193
C00020958	OPT_2720	30-Aug-06	1	N	2	R	S	+	USA	Marietta	SRR3340060
C00020981	OPT_2743	02-Jan-07	1	N	2	R	S	+	USA	Marietta	SRR3333407
C00021010	OPT_2771	15-Feb-07	1	N	2	R	S	+	USA	Marietta	SRR3339979
C00021103	OPT_2851	06-Dec-07	1	N	2	R	S	+	USA	Marietta	SRR3334040
C00010892	OPT_2058	18-Dec-07	1	N	2	R	S	+	USA	Maywood	ERS352284
C00010894	OPT_1951	15-Oct-08	1	N	2	S	S	+	USA	Maywood	ERS352279
C00020792	OPT_2557	21-Aug-09	1	N	2	R	S	+	USA	Maywood	SRR3339976
C00020848	OPT_2611	17-Feb-09	1	N	2	R	S	+	USA	Maywood	SRR3332959
C00020462	OPT_1910	19-Dec-06	1	N	2	R	S	+	USA	Minneapolis	SRR3333329
C00020502	OPT_2233	16-Jan-09	1	N	2	R	S	+	USA	Modesto	SRR3334146
C00020662	OPT_2430	12-Oct-07	1	N	2	R	S	+	USA	New Orleans	SRR3340248
C00020736	OPT_2502	14-May-09	1	N	2	S	S	+	USA	New Orleans	SRR3333106
C00020333	OPT_1821	14-Mar-08	1	N	2	R	S	+	USA	New York	SRR3339968
C00020576	OPT_2371	12-Nov-07	1	N	2	R	S	+	USA	New York	SRR3340037
C00020275	OPT_1719	21-Sep-07	1	N	2	R	S	+	USA	Newark	SRR3340027

C00020671	OPT_2439	23-Apr-08	1	N	2	R	S	+	USA	Palm Springs and Rancho Mirage	SRR3340072
C00020681	OPT_2448	22-Jun-08	1	N	2	R	S	+	USA	Palm Springs and Rancho Mirage	SRR3333007
C00020791	OPT_2556	24-Jul-09	1	N	2	R	S	+	USA	Palm Springs and Rancho Mirage	SRR3340048
C00020828	OPT_2592	08-Jan-09	1	N	2	R	S	+	USA	Palm Springs and Rancho Mirage	SRR3333383
C00020829	OPT_2593	15-Jan-09	1	N	2	R	S	+	USA	Palm Springs and Rancho Mirage	SRR3334238
C00020877	OPT_2640	17-Sep-08	1	N	2	R	S	+	USA	Palm Springs and Rancho Mirage	SRR3334444
C00020891	OPT_2653	09-Jul-08	1	N	2	R	S	+	USA	Philadelphia	SRR3333708
C00020507	OPT_2266	02-Sep-09	1	N	2	S	S	+	USA	Rapid City	SRR3333032
C00028645	OPT_2564	02-Sep-09	1	N	2	S	S	+	USA	Rapid City	SRR3333289
C00020616	OPT_2411	19-Apr-07	1	N	2	S	S	+	USA	San Jose	SRR3340071
C00020619	OPT_2414	22-Aug-07	1	N	2	R	S	+	USA	San Jose	SRR3333418
C00020669	OPT_2437	26-Dec-07	1	N	2	R	S	+	USA	San Jose	SRR3333471
C00020878	OPT_2641	23-Oct-08	1	N	2	R	S	+	USA	San Jose	SRR3334022
C00020913	OPT_2675	06-Nov-08	1	N	2	R	S	+	USA	San Jose	SRR3333437
C00020539	OPT_2335	15-Jan-08	1	N	2	R	S	+	USA	Smyrna Beach	SRR3333307
C00021020	OPT_2781	23-Mar-07	1	N	2	R	S	+	USA	Springfield	SRR3334024
C00011028	OPT_1797	05-Feb-08	1	N	2	R	S	+	USA	St. Louis	ERS352255
C00020486	OPT_2033	10-Jul-06	1	N	2	R	S	+	USA	St. Louis	SRR3334049
C00020941	OPT_2704	13-Jun-06	1	N	2	R	S	+	USA	St. Louis	SRR3333568
C00020943	OPT_2706	19-Jul-06	1	N	2	R	S	+	USA	St. Louis	SRR3333454
C00020960	OPT_2722	21-Sep-06	1	N	2	R	S	+	USA	St. Louis	SRR3340274
C00020307	OPT_1785	06-Feb-08	1	N	2	R	S	+	USA	Stamford	SRR3332955
C00021100	OPT_2848	14-Nov-07	1	N	2	R	S	+	USA	Stamford	SRR3340269
C00011052	OPT_1903	13-Sep-06	1	N	2	R	S	+	USA	Summers Point	ERS352267
C00011053	OPT_2299	08-Nov-06	1	N	2	R	S	+	USA	Summers Point	ERS352272
C00020965	OPT_2727	31-Oct-06	1	N	2	R	S	+	USA	Summers Point	SRR3333058
C00011059	OPT_2303	01-Jul-07	1	N	2	R	S	+	USA	Toledo	ERS352263
C00020311	OPT_1792	28-Nov-07	1	N	2	R	S	+	USA	Toledo	SRR3340244
C00020537	OPT_2333	03-Jan-08	1	N	2	R	S	+	USA	Toledo	SRR3334067

C00020655	OPT_2423	20-Feb-08	1	N	2	R	S	+	USA	Tupelo	SRR3340259
C00020498	OPT_2067	24-Sep-08	1	N	2	R	S	+	USA	Winchester	SRR3332968
C00020670	OPT_2438	07-Apr-08	1	N	2	R	S	+	USA	Winchester	SRR3334044
C00021068	OPT_2818	22-May-07	1	N	2	R	S	+	USA	Winchester	SRR3340243
C00021069	OPT_2819	22-May-07	1	N	2	R	S	+	USA	Winchester	SRR3333396
C00020468	OPT_1921	20-Mar-07	1	N	2	R	S	+	USA	Winston-Salem	SRR3333345
C00020517	OPT_2314	27-Jul-07	1	N	2	R	S	+	USA	Winston-Salem	SRR3333575
C00020876	OPT_2639	02-Apr-09	1	N	2	R	R	+	USA	Winston-Salem	SRR3339971
C00021000	OPT_2761	11-Jan-07	1	N	2	R	S	+	USA	Winston-Salem	SRR3340242
C00020676	OPT_2443	08-Apr-08	2	N	1	S	S	+	Belgium	Aalst	SRR3340044
C00010906	OPT_2059	23-Oct-07	2	N	1	S	S	+	Belgium	Brussels	ERS352291
C00020519	OPT_2316	29-Oct-07	2	N	1	S	S	+	Belgium	Brussels	SRR3334110
C00020720	OPT_2486	20-Mar-09	2	N	1	S	S	+	Belgium	Brussels	SRR3333379
C00020844	OPT_2607	12-Dec-08	2	N	1	S	S	+	Belgium	Brussels	SRR3333667
C00020723	OPT_2489	09-Apr-09	2	N	1	S	S	+	Belgium	Gent	SRR3333572
C00020927	OPT_2689	30-Oct-08	2	N	1	S	S	+	Belgium	Montigny-le-Tilleul	SRR3333584
C00010981	OPT_2255	15-Jun-09	2	Y	1	S	S	+	Canada	Calgary	ERS352289
C00010982	OPT_1962	01-Aug-09	2	Y	1	S	S	+	Canada	Calgary	ERS352290
C00011006	OPT_1843	19-Jun-08	2	Y	1	S	S	+	Canada	Calgary	ERS352293
C00020522	OPT_2319	15-Dec-07	2	Y	1	S	S	+	Canada	Calgary	SRR3333757
C00020812	OPT_2576	02-Oct-09	2	Y	1	S	S	+	Canada	Calgary	SRR3333743
C00020975	OPT_2737	11-Dec-06	2	Y	1	S	S	+	Canada	Calgary	SRR3333245
C00020991	OPT_2753	30-Jan-07	2	Y	1	S	S	+	Canada	Calgary	SRR3333817
C00021072	OPT_2822	12-Jun-07	2	Y	1	S	S	+	Canada	Calgary	SRR3340053
C00020560	OPT_2355	18-Feb-08	2	N	1	S	S	+	Canada	Chicoutimi	SRR3333105
C00020700	OPT_2466	10-Feb-09	2	N	1	S	S	+	Canada	Chicoutimi	SRR3333473
C00020738	OPT_2504	17-Apr-09	2	N	1	S	S	+	Canada	Hamilton	SRR3333627
C00011064	OPT_1827	28-Nov-07	2	N	1	S	S	+	Canada	Levis	ERS352294
C00010949	OPT_2065	05-Oct-08	2	Y	1	S	S	+	Canada	Montreal	ERS352292
C00020278	OPT_1722	25-Sep-07	2	Y	1	S	S	+	Canada	Montreal	SRR3333785

C00020433	OPT_1830	12-Feb-08	2	Y	1	S	S	+	Canada	Montreal	SRR3334228
C00020472	OPT_1926	04-May-09	2	Y	1	S	S	+	Canada	Montreal	SRR3333472
C00020480	OPT_1936	12-May-09	2	Y	1	S	S	+	Canada	Montreal	SRR3333601
C00020552	OPT_2348	31-Jan-08	2	Y	1	S	S	+	Canada	Montreal	SRR3333633
C00020706	OPT_2472	16-Feb-09	2	Y	1	S	S	+	Canada	Montreal	SRR3333693
C00021007	OPT_2768	09-Feb-07	2	Y	1	S	S	+	Canada	Montreal	SRR3340063
C00021075	OPT_2825	26-Jun-07	2	Y	1	S	S	+	Canada	Montreal	SRR3334006
C00011076	OPT_1750	30-May-08	2	N	1	S	S	+	Canada	Saskatoon	ERS352296
C00020719	OPT_2485	15-Apr-09	2	N	1	S	S	+	Canada	Trois-Rivieres	SRR3332954
C00020740	OPT_2506	02-May-09	2	N	1	S	S	+	Canada	Trois-Rivieres	SRR3333643
C00020836	OPT_2600	19-Dec-08	2	N	1	S	S	+	Canada	Trois-Rivieres	SRR3334227
C00020805	OPT_2569	03-Feb-09	2	N	1	S	S	+	France	Amiens Cedex	SRR3334462
C00020492	OPT_2043	09-Sep-08	2	N	1	S	S	+	France	Garches	SRR3333741
C00020673	OPT_2441	26-Mar-08	2	N	1	S	S	+	France	Garches	SRR3339972
C00020759	OPT_2525	14-Oct-09	2	N	1	S	S	+	France	Garches	SRR3333422
C00020678	OPT_2445	06-May-08	2	N	1	S	R	+	Germany	Köln	SRR3333998
C00020721	OPT_2487	25-Mar-09	2	N	1	S	S	+	Germany	Regensburg	SRR3333803
C00020765	OPT_2531	08-May-09	2	N	1	S	S	+	Italy	Modena	SRR3340042
C00020971	OPT_2733	08-Dec-06	2	N	1	S	S	+	USA	Chicago	SRR3333116
C00021061	OPT_2811	06-Jun-07	2	N	1	R	S	+	USA	Chicago	SRR3334114
C00021088	OPT_2836	01-Aug-07	2	N	1	S	S	+	USA	Chicago	SRR3333378
C00020867	OPT_2630	09-Jun-08	2	N	1	S	S	+	USA	Fargo	SRR3340022
C00020296	OPT_1744	09-Jan-08	2	N	1	S	S	+	USA	Idaho Falls	SRR3333261
C00020262	OPT_1701	25-Jun-07	2	N	1	S	S	+	USA	Keego Harbor	SRR3334061
C00020795	OPT_2559	04-Apr-09	2	N	1	S	S	+	USA	Keego Harbor	SRR3334027
C00011023	OPT_2291	06-Dec-06	2	N	1	S	S	+	USA	Marietta	ERS352409
C00011024	OPT_1850	26-Mar-08	2	N	1	S	S	+	USA	Marietta	ERS352295
C00020955	OPT_2717	16-Aug-06	2	N	1	S	S	+	USA	Marietta	SRR3333088
C00021023	OPT_2784	13-Mar-07	2	N	1	S	S	+	USA	Marietta	SRR3334230
C00011061	OPT_1856	24-Jun-08	2	N	1	S	S	+	USA	Smyrna Beach	ERS352416

C00010986	OPT_1911	07-Mar-07	3	Y	1	R	S	+	Canada	Calgary	ERS352305
C00010987	OPT_2281	13-Mar-07	3	Y	1	S	S	+	Canada	Calgary	ERS352302
C00010995	OPT_1711	14-Sep-07	3	Y	1	S	S	+	Canada	Calgary	ERS352304
C00011000	OPT_1889	19-Jan-08	3	Y	1	R	S	+	Canada	Calgary	ERS352297
C00020246	OPT_1686	16-Aug-07	3	Y	1	R	S	+	Canada	Calgary	SRR3339970
C00020294	OPT_1742	21-May-08	3	Y	1	R	S	+	Canada	Calgary	SRR3333739
C00020551	OPT_2347	24-Feb-08	3	Y	1	R	S	+	Canada	Calgary	SRR3333680
C00020561	OPT_2356	08-Dec-07	3	Y	1	R	S	+	Canada	Calgary	SRR3333639
C00020938	OPT_2701	03-Oct-06	3	Y	1	S	S	+	Canada	Calgary	SRR3340016
C00020987	OPT_2749	12-Jan-07	3	Y	1	R	S	+	Canada	Calgary	SRR3334222
C00020988	OPT_2750	13-Jan-07	3	Y	1	R	S	+	Canada	Calgary	SRR3333326
C00020990	OPT_2752	23-Jan-07	3	Y	1	R	S	+	Canada	Calgary	SRR3333008
C00010930	OPT_2240	24-Mar-09	3	N	1	S	S	+	Canada	Chicoutimi	ERS352299
C00028643	OPT_2470	15-Jan-09	3	N	1	S	S	+	Canada	Chicoutimi	SRR3333347
C00010971	OPT_2258	14-May-09	3	N	1	R	S	+	Canada	Edmonton	ERS352300
C00010974	OPT_2264	16-Jul-09	3	N	1	S	S	+	Canada	Edmonton	ERS352303
C00020493	OPT_2045	18-Sep-08	3	N	1	R	S	+	Canada	Montreal	SRR3333243
C00020567	OPT_2362	20-Mar-08	3	N	1	S	S	+	Canada	Montreal	SRR3340079
C00020731	OPT_2497	05-Mar-09	3	N	1	S	S	+	Canada	Montreal	SRR3333091
C00020902	OPT_2664	23-Sep-08	3	N	1	S	S	-	Canada	Montreal	SRR3340091
C00020977	OPT_2739	22-Nov-06	3	N	1	S	S	+	Canada	Montreal	SRR3340067
C00020774	OPT_2539	16-Jun-09	3	N	1	S	S	+	Canada	Trois-Rivieres	SRR3333358
C00020837	OPT_2601	29-Dec-08	3	N	1	S	S	+	Canada	Trois-Rivieres	SRR3333698
C00020685	OPT_2452	27-Jun-08	3	N	1	S	S	+	France	Garches	SRR3333062
C00020479	OPT_1933	06-Jan-09	3	N	1	R	S	+	Germany	Köln	SRR3333566
C00020689	OPT_2456	01-Jul-08	3	N	1	R	S	+	Germany	Köln	SRR3333448
C00020694	OPT_2460	05-Aug-08	3	N	1	R	S	+	Germany	Köln	SRR3340183
C00020698	OPT_2464	25-Jul-08	3	N	1	R	S	+	Germany	Köln	SRR3334166
C00020744	OPT_2510	07-Sep-09	3	N	1	R	S	+	Germany	Köln	SRR3333397
C00020775	OPT_2540	18-Jun-09	3	N	1	R	S	+	Germany	Köln	SRR3333925
C00020788	OPT_2553	30-Jul-09	3	N	1	R	S	+	Germany	Köln	SRR3334070

C00020840	OPT_2604	14-Jan-09	3	N	1	R	S	+	Germany	Köln	SRR3334471
C00020802	OPT_2566	12-Mar-09	3	N	1	R	S	+	Germany	Lübeck	SRR3340195
C00020659	OPT_2427	16-Jan-08	3	N	1	S	S	+	Sweden	Orebro	SRR3333443
C00020490	OPT_2039	13-Jun-08	3	N	1	R	S	+	UK	Brighton	SRR3334209
C00020615	OPT_2410	30-Nov-07	3	N	1	R	S	+	UK	York	SRR3334097
C00020516	OPT_2313	10-Jul-07	3	N	1	R	S	+	USA	Butte	SRR3334026
C00020922	OPT_2684	10-Nov-08	3	N	1	R	S	+	USA	Chicago	SRR3333094
C00021058	OPT_2808	21-May-07	3	N	1	R	S	+	USA	Chicago	SRR3340065
C00020512	OPT_2309	13-Oct-06	3	N	1	R	S	+	USA	Cleveland	SRR3333296
C00020898	OPT_2660	18-Sep-08	3	N	1	S	S	+	USA	Fargo	SRR3334019
C00020794	OPT_2558	21-Jan-09	3	N	1	R	S	+	USA	Keego Harbor	SRR3339960
C00011047	OPT_1866	29-Aug-07	3	N	1	S	S	+	USA	Madison	ERS352298
C00020888	OPT_2651	15-Jul-08	3	N	1	S	S	-	USA	Maywood	SRR3334440
C00011044	OPT_1845	10-Nov-07	3	N	1	S	S	+	USA	Minneapolis	ERS352301
C00020957	OPT_2719	24-Aug-06	3	N	1	S	S	+	USA	St. Louis	SRR3333425
C00020964	OPT_2726	06-Oct-06	3	N	1	R	S	+	USA	Summers Point	SRR3332977
C00020649	OPT_2417	17-Jan-08	3	N	1	S	S	+	USA	Tupelo	SRR3334178
C00020989	OPT_2751	22-Jan-07	4	N	1	S	S	+	Canada	Calgary	SRR3333695
C00020290	OPT_1738	29-Sep-07	4	N	1	S	R	+	Canada	Montreal	SRR3340003
C00020604	OPT_2399	02-Jun-08	4	N	1	S	S	+	Canada	Montreal	SRR3334198
C00020606	OPT_2401	16-Jun-08	4	N	1	S	S	+	Canada	Montreal	SRR3334184
C00020680	OPT_2447	16-May-08	4	N	1	S	S	+	Sweden	Orebro	SRR3333006
C00020970	OPT_2732	01-Dec-06	4	N	1	S	S	+	USA	Buffalo	SRR3333562
C00021091	OPT_2839	14-Jun-07	4	N	1	S	S	+	USA	Idaho Falls	SRR3334423
C00020956	OPT_2718	23-Aug-06	4	N	1	S	S	+	USA	Marietta	SRR3333237
C00020779	OPT_2544	14-Jul-09	5	N	3	S	S	+	Canada	Calgary	SRR3333469
C00020504	OPT_2244	03-Apr-09	5	N	3	S	S	+	Spain	Madrid	SRR3333228
C00020722	OPT_2488	19-Nov-08	6	N	1	S	S	+	Belgium	Brussels	SRR3333315
C00020618	OPT_2413	18-Oct-07	6	N	1	S	S	+	Belgium	Kortrijk	SRR3333346
C00020550	OPT_2346	23-Feb-08	6	N	1	S	S	+	Canada	Calgary	SRR3334057

C00020809	OPT_2573	11-Sep-09	6	N	1	S	S	+	Canada	Calgary	SRR3333021
C00010946	OPT_2080	16-Oct-08	6	N	1	S	S	+	Canada	Montreal	ERS352421
C00020923	OPT_2685	24-Oct-08	6	N	1	S	S	+	France	Garches	SRR3333583
C00020666	OPT_2434	16-Nov-07	6	N	1	S	S	+	Germany	Köln	SRR3333647
C00028637	OPT_1743	28-May-08	6	N	1	S	S	+	USA	Allentown	SRR3334427
C00011014	OPT_2288	20-Mar-07	6	N	1	S	S	+	USA	Chicago	ERS352307
C00020435	OPT_1832	25-Jun-08	6	N	1	S	S	+	USA	Chicago	SRR3333252
C00020825	OPT_2589	18-Nov-08	6	N	1	S	S	+	USA	Chicago	SRR3334456
C00020921	OPT_2683	29-Oct-08	6	N	1	S	R	+	USA	Chicago	SRR3340231
C00020996	OPT_2757	10-Jan-07	6	N	1	S	S	+	USA	Chicago	SRR3340227
C00020790	OPT_2555	30-Jul-09	6	N	1	S	S	+	USA	Fargo	SRR3333027
C00020505	OPT_2252	03-Apr-09	6	N	1	S	S	+	USA	Idaho Falls	SRR3333624
C00020963	OPT_2725	16-Oct-06	6	N	1	S	S	+	USA	Indianapolis	SRR3339975
C00020839	OPT_2603	21-Jan-09	7	N	1	S	S	-	UK	Brighton	SRR3333997
C00020657	OPT_2425	22-Feb-08	8	N	1	S	S	+	Belgium	Kortrijk	SRR3334158
C00020782	OPT_2547	24-Mar-09	8	N	1	S	R	+	Belgium	Mons	SRR3334135
C00010977	OPT_1934	28-May-09	8	Y	1	S	S	+	Canada	Calgary	ERS352311
C00020287	OPT_1734	01-Nov-07	8	Y	1	S	S	+	Canada	Calgary	SRR3340280
C00020584	OPT_2379	25-Nov-07	8	Y	1	S	S	+	Canada	Calgary	SRR3333336
C00020796	OPT_2560	23-Apr-09	8	Y	1	S	S	+	Canada	Calgary	SRR3333367
C00020808	OPT_2572	09-Sep-09	8	Y	1	S	S	+	Canada	Calgary	SRR3333405
C00020934	OPT_2697	08-Sep-06	8	Y	1	S	S	+	Canada	Calgary	SRR3340009
C00020966	OPT_2728	23-Sep-06	8	Y	1	S	S	+	Canada	Calgary	SRR3333234
C00021005	OPT_2766	09-Feb-07	8	Y	1	S	S	+	Canada	Calgary	SRR3333750
C00021052	OPT_2802	23-May-07	8	Y	1	S	S	+	Canada	Calgary	SRR3333712
C00021082	OPT_2831	03-Aug-07	8	Y	1	S	S	+	Canada	Calgary	SRR3334013
C00020548	OPT_2344	20-Nov-07	8	N	1	S	S	+	Canada	Chicoutimi	SRR3333760
C00020701	OPT_2467	19-Feb-09	8	N	1	S	S	+	Canada	Chicoutimi	SRR3333274
C00020931	OPT_2693	04-Nov-08	8	N	1	S	S	+	Canada	Chicoutimi	SRR3334012
C00020587	OPT_2382	28-Mar-08	8	N	1	S	R	+	Canada	Hamilton	SRR3333573

C00020610	OPT_2405	16-Jul-08	8	N	1	S	S	+	Canada	Hamilton	SRR3333413
C00020859	OPT_2622	26-Jan-09	8	N	1	S	S	+	Canada	Hamilton	SRR3333632
C00020446	OPT_1880	18-Feb-08	8	Y	1	S	S	+	Canada	Montreal	SRR3340260
C00020497	OPT_2057	26-Aug-08	8	Y	1	S	S	+	Canada	Montreal	SRR3333423
C00020532	OPT_2328	12-Dec-07	8	Y	1	S	S	+	Canada	Montreal	SRR3333096
C00020590	OPT_2385	17-Mar-08	8	Y	1	S	S	+	Canada	Montreal	SRR3334109
C00020712	OPT_2478	17-Feb-09	8	Y	1	S	S	+	Canada	Montreal	SRR3333147
C00020728	OPT_2494	01-Mar-09	8	Y	1	S	S	+	Canada	Montreal	SRR3332961
C00020862	OPT_2625	23-Jan-09	8	Y	1	S	S	+	Canada	Montreal	SRR3333076
C00021008	OPT_2769	21-Feb-07	8	Y	1	S	S	+	Canada	Montreal	SRR3333014
C00021106	OPT_2855	14-Feb-08	8	Y	1	S	S	+	Canada	Montreal	SRR3333051
C00028638	OPT_1922	26-Jun-07	8	Y	1	S	S	+	Canada	Montreal	SRR3334092
C00011075	OPT_1876	01-Mar-08	8	N	1	S	S	+	Canada	Saskatoon	ERS352309
C00021105	OPT_2854	13-Mar-08	8	N	1	S	S	+	Canada	Toronto	SRR3333037
C00010967	OPT_2275	09-Nov-09	8	N	1	S	S	+	Canada	Trois-Rivieres	ERS352312
C00020741	OPT_2507	07-May-09	8	N	1	S	S	+	Canada	Trois-Rivieres	SRR3334028
C00020748	OPT_2514	14-Oct-09	8	N	1	S	S	+	Canada	Trois-Rivieres	SRR3333440
C00020872	OPT_2635	18-Feb-09	8	N	1	S	S	+	France	Lille	SRR3333285
C00020787	OPT_2552	18-Jul-09	8	N	1	S	S	+	Italy	Modena	SRR3333637
C00010910	OPT_1947	05-Apr-08	8	N	1	S	S	+	Sweden	Orebro	ERS352310
C00021071	OPT_2821	13-Jun-07	8	N	1	S	S	+	UK	York	SRR3334004
C00011035	OPT_2295	07-Jun-07	8	N	1	S	S	+	USA	Akron	ERS352419
C00020243	OPT_1682	26-Jul-07	8	N	1	S	S	+	USA	Akron	SRR3333779
C00020614	OPT_2409	10-Nov-07	8	N	1	S	S	+	USA	Akron	SRR3333348
C00021113	OPT_2862	03-Jan-08	8	N	1	S	S	+	USA	Butte	SRR3340257
C00020501	OPT_2079	22-Jan-09	8	N	1	S	R	+	USA	Chicago	SRR3333727
C00020953	OPT_2715	28-Aug-06	8	N	1	S	S	+	USA	Chicago	SRR3334124
C00021057	OPT_2807	14-May-07	8	N	1	S	S	+	USA	Chicago	SRR3333392
C00020999	OPT_2760	03-Jan-07	8	N	1	R	S	+	USA	Columbus	SRR3333686
C00021111	OPT_2860	12-Jul-08	8	N	1	S	S	+	USA	Idaho Falls	SRR3333017
C00020258	OPT_1697	21-Jun-07	8	N	1	S	S	+	USA	Jena	SRR3333340

C00020979	OPT_2741	11-Dec-06	8	N	1	S	S	+	USA	Marietta	SRR3340220
C00020851	OPT_2614	28-Jan-09	8	N	1	S	S	+	USA	Palm Springs and Rancho Mirage	SRR3333238
C00020785	OPT_2550	18-Feb-09	8	N	1	S	S	+	USA	Winston-Salem	SRR3334331
C00021090	OPT_2838	02-Aug-07	8	N	1	S	S	+	USA	Winston-Salem	SRR3332952
C00020679	OPT_2446	09-May-08	10	N	1	S	S	+	Belgium	Brussels	SRR3332973
C00010998	OPT_1828	29-Dec-07	10	N	1	S	S	+	Canada	Calgary	ERS352314
C00020301	OPT_1749	11-Jul-07	10	N	1	S	S	+	Canada	Chicoutimi	SRR3333791
C00020449	OPT_1883	15-Nov-07	10	N	1	S	S	+	Canada	Chicoutimi	SRR3334037
C00020703	OPT_2469	11-Mar-09	10	N	1	S	S	+	Canada	Hamilton	SRR3334090
C00020852	OPT_2615	03-Feb-09	10	N	1	S	S	+	Canada	Hamilton	SRR3332997
C00010950	OPT_2072	07-Dec-08	10	N	1	S	S	+	Canada	Montreal	ERS352313
C00020835	OPT_2599	16-Dec-08	10	N	1	S	S	+	Canada	Montreal	SRR3333403
C00020597	OPT_2392	13-Jun-08	10	N	1	S	S	+	Canada	Saskatoon	SRR3340033
C00020260	OPT_1699	16-Aug-07	10	N	1	S	S	+	Canada	Toronto	SRR3334191
C00020751	OPT_2517	16-Dec-08	10	N	1	S	S	+	France	Garches	SRR3333330
C00020254	OPT_1693	15-Aug-07	10	N	1	S	S	+	USA	Akron	SRR3333621
C00020950	OPT_2713	22-Aug-06	10	N	1	S	S	+	USA	Buffalo	SRR3333066
C00021027	OPT_2789	04-Apr-07	10	N	1	S	S	+	USA	Butte	SRR3334421
C00020608	OPT_2403	27-Jun-08	10	N	1	S	S	+	USA	Chicago	SRR3340224
C00021025	OPT_2786	19-Mar-07	10	N	1	S	S	+	USA	Columbus	SRR3334410
C00020511	OPT_2308	30-Aug-06	10	N	1	S	S	+	USA	Marietta	SRR3333993
C00021062	OPT_2812	23-May-07	10	N	1	S	S	+	USA	Marietta	SRR3333841
C00011046	OPT_1852	22-Apr-08	10	N	1	S	S	+	USA	Minneapolis	ERS352415
C00020264	OPT_1703	09-Oct-07	11	N	5	R	S	+	Belgium	Brussels	SRR3340054
C00020613	OPT_2408	26-Oct-07	11	N	5	S	S	+	Belgium	Brussels	SRR3333749
C00020815	OPT_2579	24-Jun-09	11	N	5	S	S	+	Belgium	Columbia	SRR3334450
C00020758	OPT_2524	22-Jan-09	11	N	5	S	S	+	Belgium	Mons	SRR3333923
C00020335	OPT_1823	02-Jan-08	11	N	5	S	S	+	Canada	Calgary	SRR3340089
C00020276	OPT_1720	11-Sep-07	11	N	5	S	S	+	Canada	Montreal	SRR3333359
C00020651	OPT_2419	31-Jan-08	11	N	5	S	S	+	Canada	Toronto	SRR3333452

C00020906	OPT_2668	22-Sep-08	11	N	5	R	S	+	Canada	Toronto	SRR3333604
C00020843	OPT_2606	24-Oct-08	11	N	5	S	S	+	France	Amiens Cedex	SRR3333688
C00020847	OPT_2610	26-Jun-08	11	N	5	S	S	+	France	Caen Cedex	SRR3333074
C00020780	OPT_2545	30-Jun-09	11	N	5	S	S	+	Germany	Köln	SRR3333225
C00020826	OPT_2590	08-Jan-09	11	N	5	R	S	+	Italy	Arsizio	SRR3333586
C00020684	OPT_2451	23-Jun-08	11	N	5	R	S	+	Italy	Modena	SRR3334098
C00020870	OPT_2633	26-Feb-09	11	N	5	R	S	+	Italy	Modena	SRR3333317
C00020871	OPT_2634	11-Mar-09	11	N	5	S	S	+	Italy	Modena	SRR3333111
C00020885	OPT_2648	30-Sep-08	11	N	5	S	S	+	Italy	Modena	SRR3334464
C00016337	OPT-2075	01-Apr-08	11	N	5	R	S	+	Sweden	Orebro	ERS352315
C00020273	OPT_1717	02-Oct-07	11	N	5	S	S	+	USA	Butte	SRR3333394
C00020431	OPT_1825	12-Jul-08	11	N	5	S	S	+	USA	Butte	SRR3334418
C00020432	OPT_1826	23-Apr-08	11	N	5	S	S	+	USA	Butte	SRR3340208
C00021004	OPT_2765	21-Jan-07	11	N	5	S	S	+	USA	Butte	SRR3333052
C00020543	OPT_2339	03-Feb-08	11	N	5	S	S	+	USA	Dothan	SRR3340200
C00021049	OPT_2799	09-May-07	11	N	5	S	S	+	USA	Dothan	SRR3340232
C00020475	OPT_1929	04-Mar-09	11	N	5	S	S	+	USA	Idaho Falls	SRR3333242
C00020491	OPT_2040	25-Nov-08	11	N	5	S	S	+	USA	Idaho Falls	SRR3340268
C00020577	OPT_2372	20-Jul-07	11	N	5	S	S	+	USA	Idaho Falls	SRR3340045
C00020445	OPT_1879	06-Nov-07	11	N	5	S	S	+	USA	Keego Harbor	SRR3334206
C00020253	OPT_1692	30-Aug-07	11	N	5	S	S	+	USA	Maywood	SRR3333731
C00020620	OPT_2415	16-Jan-08	11	N	5	S	S	+	USA	Maywood	SRR3333327
C00021002	OPT_2763	29-Nov-06	11	N	5	S	S	+	USA	Minneapolis	SRR3333813
C00020621	OPT_2416	07-Jan-08	11	N	5	S	S	+	USA	New Orleans	SRR3334084
C00020280	OPT_1724	21-Sep-07	11	N	5	S	S	+	USA	Winston-Salem	SRR3333063
C00020283	OPT_1727	11-Oct-07	12	N	1	S	S	+	Germany	Köln	SRR3333406
C00020263	OPT_1702	17-Sep-07	12	N	1	S	S	+	UK	Brighton	SRR3333769
C00020827	OPT_2591	21-Nov-08	13	N	1	S	S	+	Belgium	Kortrijk	SRR3333300
C00020814	OPT_2578	24-Sep-09	13	N	1	S	S	+	France	Garches	SRR3333034
C00021114	OPT_2863	12-Feb-08	13	N	1	S	S	+	USA	Butte	SRR3334197

C00020284	OPT_1731	16-Oct-07	14	N	1	S	S	+	Canada	Calgary	SRR3340097
C00020534	OPT_2330	17-Jan-08	14	N	1	S	S	+	Canada	Calgary	SRR3333288
C00020947	OPT_2710	03-Sep-06	14	N	1	S	S	+	Canada	Calgary	SRR3332957
C00021006	OPT_2767	05-Feb-07	14	N	1	S	S	+	Canada	Montreal	SRR3334003
C00020612	OPT_2407	08-Jul-08	14	N	1	S	S	+	Canada	Saskatoon	SRR3340228
C00020742	OPT_2508	12-May-09	14	N	1	S	S	+	Canada	Trois-Rivieres	SRR3333730
C00020747	OPT_2513	09-Oct-09	14	N	1	S	S	+	Canada	Trois-Rivieres	SRR3333282
C00020770	OPT_2536	19-Jun-09	14	N	1	S	S	+	Canada	Trois-Rivieres	SRR3340015
C00020806	OPT_2570	03-Sep-09	14	N	1	S	S	+	Canada	Trois-Rivieres	SRR3334467
C00020572	OPT_2367	12-Nov-07	14	N	1	S	S	+	USA	Chicago	SRR3334116
C00021059	OPT_2809	31-May-07	14	N	1	R	S	+	USA	Chicago	SRR3334149
C00020739	OPT_2505	10-Apr-09	14	N	1	S	R	+	USA	Idaho Falls	SRR3334062
C00020857	OPT_2620	05-Dec-08	14	N	1	S	S	+	USA	Idaho Falls	SRR3334122
C00011022	OPT_2290	03-Nov-06	14	N	1	S	S	+	USA	Marietta	ERS352316
C00021095	OPT_2843	10-Jul-07	14	N	1	S	S	+	USA	Winchester	SRR3333086
C00020454	OPT_1892	02-Aug-07	15	N	1	R	S	-	USA	Chicago	SRR3334441
C00020513	OPT_2310	13-Oct-06	15	N	1	S	S	-	USA	Marietta	SRR3334144
C00020831	OPT_2595	19-Jan-09	16	N	1	S	S	+	Canada	Hamilton	SRR3334405
C00020463	OPT_1912	08-Feb-07	16	N	1	S	S	+	Canada	Montreal	SRR3333304
C00020750	OPT_2516	08-Oct-09	16	N	1	S	S	+	France	Lille	SRR3333102
C00020656	OPT_2424	19-Mar-08	16	N	1	S	S	+	Germany	Köln	SRR3334211
C00020695	OPT_2461	13-Aug-08	16	N	1	S	S	+	Germany	Köln	SRR3340214
C00020555	OPT_2350	19-Feb-08	16	N	1	S	S	+	USA	Allentown	SRR3333612
C00020746	OPT_2512	11-Sep-09	17	Y	1	R	S	+	Italy	Arsizio	SRR3340202
C00020766	OPT_2532	15-May-09	17	Y	1	R	S	+	Italy	Arsizio	SRR3334104
C00020873	OPT_2636	26-Feb-09	17	Y	1	R	S	+	Italy	Arsizio	SRR3340240
C00020506	OPT_2254	10-Jun-09	17	Y	1	R	S	+	Italy	Modena	SRR3334231
C00020677	OPT_2444	12-Jun-08	17	Y	1	R	S	+	Italy	Modena	SRR3333780
C00020734	OPT_2500	23-Apr-09	17	Y	1	R	S	+	Italy	Modena	SRR3333787
C00020735	OPT_2501	06-May-09	17	Y	1	R	S	+	Italy	Modena	SRR3334023

C00020773	OPT_2538	24-Jun-09	17	Y	1	R	S	+	Italy	Modena	SRR3340193
C00020801	OPT_2565	10-Aug-09	17	Y	1	R	S	+	Italy	Modena	SRR3340215
C00020813	OPT_2577	23-Sep-09	17	Y	1	R	S	+	Italy	Modena	SRR3333266
C00028644	OPT_2549	04-Aug-09	17	Y	1	R	S	+	Italy	Modena	SRR3334118
C00020692	OPT_2458	18-Jul-08	17	Y	1	R	S	+	Italy	Torino	SRR3333676
C00020881	OPT_2644	23-Jul-08	17	Y	1	R	S	+	Italy	Torino	SRR3333337
C00020452	OPT_1887	16-Apr-08	17	N	1	R	S	+	USA	Butte	SRR3334005
C00011040	OPT_2297	07-May-07	17	N	1	S	S	+	USA	Winston-Salem	ERS352317
C00011010	OPT_2287	29-Jun-07	18	N	1	S	R	+	Canada	Montreal	ERS352318
C00020585	OPT_2380	11-Oct-07	18	N	1	S	S	+	Canada	Montreal	SRR3333794
C00020886	OPT_2649	08-Oct-08	18	N	1	S	S	+	Germany	Köln	SRR3340267
C00010933	OPT_2248	08-Apr-09	21	N	1	S	S	+	Canada	Chicoutimi	ERS352319
C00020580	OPT_2375	19-Nov-07	21	N	1	R	S	+	USA	Chicago	SRR3332966
C00020727	OPT_2493	27-Apr-09	21	N	1	R	S	+	USA	Chicago	SRR3340272
C00020660	OPT_2428	09-Jan-08	22	N	3	S	S	+	Sweden	Göteborg	SRR3334154
C00020271	OPT_1714	02-Oct-07	32	N	2	S	S	+	Canada	Calgary	SRR3334137
C00021092	OPT_2840	10-Jul-07	32	N	2	S	S	+	USA	Idaho Falls	SRR3333028
C00020962	OPT_2724	11-Oct-06	32	N	2	S	S	+	USA	Marietta	SRR3333356
C00010993	OPT_1689	04-Sep-07	34	N	1	S	S	+	Canada	Calgary	ERS352321
C00020565	OPT_2360	23-Apr-08	34	N	1	S	S	+	Canada	Calgary	SRR3334155
C00028648	OPT_2738	13-Dec-06	34	N	1	S	S	+	Canada	Calgary	SRR3340203
C00020832	OPT_2596	08-Dec-08	34	N	1	S	S	+	Canada	Chicoutimi	SRR3333656
C00020288	OPT_1736	26-Sep-07	34	N	1	S	S	+	Canada	Montreal	SRR3333119
C00020811	OPT_2575	06-Oct-09	34	N	1	S	S	+	Canada	Trois-Rivieres	SRR3333431
C00021045	OPT_2795	24-Apr-07	34	N	1	S	S	+	USA	Detroit	SRR3332994
C00011045	OPT_1851	29-Mar-08	34	N	1	S	S	+	USA	Minneapolis	ERS352322
C00010902	OPT_2305	07-Jun-07	34	N	1	S	R	+	USA	Winchester	ERS352320
C00020658	OPT_2426	03-Jan-08	35	N	1	R	S	+	Germany	Köln	SRR3333457
C00028636	OPT_1723	02-Oct-07	36	N	1	S	S	+	Canada	Montreal	SRR3333316

C00020763	OPT_2529	23-Oct-08	36	N	1	S	S	+	UK	Surrey	SRR3333097
C00020574	OPT_2369	29-Dec-07	37	N	4	S	S	+	Canada	Calgary	SRR3339984
C00020696	OPT_2462	02-Sep-08	37	N	4	S	S	+	Germany	Köln	SRR3333035
C00020526	OPT_2323	12-Dec-07	37	N	4	R	S	+	USA	Detroit	SRR3334007
C00021048	OPT_2798	12-Apr-07	37	N	4	S	S	+	USA	Dothan	SRR3334200
C00020920	OPT_2682	16-Oct-08	37	N	4	R	S	+	USA	Fargo	SRR3332989
C00020476	OPT_1930	17-Mar-09	37	N	4	S	S	+	USA	Idaho Falls	SRR3339961
C00020536	OPT_2332	28-Jan-08	37	N	4	R	S	+	USA	Johnson City	SRR3333388
C00020743	OPT_2509	06-May-09	37	N	4	S	S	+	USA	New Orleans	SRR3333995
C00020889	OPT_2652	11-Jul-08	37	N	4	S	S	+	USA	New Orleans	SRR3333561
C00020974	OPT_2736	02-Nov-06	37	N	4	S	S	+	USA	Salt Lake City	SRR3332953
C00011062	OPT_1859	09-Jun-08	37	N	4	R	S	+	USA	Smyrna Beach	ERS352323
C00010895	OPT_1945	15-Nov-07	37	N	4	R	S	+	USA	Tupelo	ERS352324
C00020286	OPT_1733	31-Oct-07	41	N	2	S	S	+	Canada	Calgary	SRR3333804
C00021081	OPT_2830	18-Jul-07	41	N	2	S	S	+	Canada	Calgary	SRR3334172
C00020752	OPT_2518	28-Sep-09	41	N	2	S	S	+	Canada	Edmonton	SRR3334074
C00010963	OPT_2271	26-Oct-09	41	N	2	S	S	+	Canada	Trois-Rivieres	ERS352325
C00011034	OPT_2294	22-May-07	41	N	2	S	S	+	USA	Akron	ERS352326
C00020969	OPT_2731	06-Nov-06	41	N	2	S	S	+	USA	Cleveland	SRR3334422
C00020524	OPT_2321	30-Nov-07	41	N	2	S	S	+	USA	Columbus	SRR3340264
C00020594	OPT_2389	30-May-08	41	N	2	S	S	+	USA	Detroit	SRR3333466
C00020320	OPT_1807	19-Apr-08	41	N	2	S	S	+	USA	Dothan	SRR3334143
C00020849	OPT_2612	23-Jan-09	41	N	2	S	S	+	USA	Fargo	SRR3333679
C00021046	OPT_2796	03-Apr-07	41	N	2	S	S	+	USA	Idaho Falls	SRR3334457
C00021014	OPT_2775	12-Feb-07	41	N	2	S	S	+	USA	Minneapolis	SRR3333260
C00010979	OPT_1961	23-Jun-09	42	N	1	S	S	+	Canada	Calgary	ERS352404
C00010990	OPT_2284	04-Jun-07	42	N	1	S	S	+	Canada	Calgary	ERS352436
C00020853	OPT_2616	12-Feb-09	42	N	1	S	S	+	Canada	Hamilton	SRR3334081
C00020252	OPT_1691	09-Aug-07	42	N	1	S	S	+	Canada	Montreal	SRR3333278
C00020453	OPT_1891	31-Mar-08	42	N	1	S	S	+	Canada	Montreal	SRR3340075

C00020603	OPT_2398	02-May-08	42	N	1	S	S	+	Canada	Montreal	SRR3333334
C00020798	OPT_2562	24-Feb-09	42	N	1	S	S	+	Spain	Madrid	SRR3340235
C00021070	OPT_2820	20-Jun-07	42	N	1	R	S	+	UK	Brighton	SRR3333078
C00020281	OPT_1725	29-Aug-07	42	N	1	R	S	+	UK	Cambridge	SRR3333389
C00020937	OPT_2700	09-May-06	42	N	1	S	S	+	USA	Akron	SRR3333324
C00020434	OPT_1831	22-Feb-07	42	N	1	S	S	+	USA	Chicago	SRR3333748
C00020571	OPT_2366	31-Oct-07	42	N	1	S	S	+	USA	Chicago	SRR3333001
C00020824	OPT_2588	03-Dec-08	42	N	1	S	S	+	USA	Columbus	SRR3334077
C00020904	OPT_2666	01-Aug-08	42	N	1	S	S	+	USA	Palm Springs and Rancho Mirage	SRR3339959
C00020789	OPT_2554	10-Aug-09	42	N	1	S	S	+	USA	Port Orange	SRR3340238
C00020777	OPT_2542	16-Jul-09	42	N	1	R	S	+	USA	Winston-Salem	SRR3333010
C00020767	OPT_2533	21-May-09	43	N	1	S	S	+	Canada	Calgary	SRR3333707
C00020730	OPT_2496	03-Mar-09	43	N	1	S	S	+	Canada	Montreal	SRR3334465
C00020579	OPT_2374	11-Oct-07	43	N	1	S	S	+	Canada	Toronto	SRR3333118
C00020754	OPT_2520	22-Oct-09	43	N	1	S	S	+	Canada	Trois-Rivieres	SRR3340035
C00020653	OPT_2421	24-Aug-07	43	N	1	S	S	+	UK	Barnsley	SRR3339956
C00020875	OPT_2638	03-Apr-09	43	N	1	S	S	+	USA	Cedar Knolls	SRR3334001
C00020609	OPT_2404	11-Jul-08	43	N	1	S	S	+	USA	Chicago	SRR3333012
C00020910	OPT_2672	07-Oct-08	43	N	1	S	S	+	USA	Chicago	SRR3333456
C00020520	OPT_2317	17-Nov-07	43	N	1	S	S	+	USA	Detroit	SRR3333060
C00020895	OPT_2657	04-Sep-08	43	N	1	S	S	+	USA	Fargo	SRR3333115
C00020768	OPT_2534	26-Feb-09	43	N	1	S	S	+	USA	Marietta	SRR3334046
C00020903	OPT_2665	08-Oct-08	43	N	1	S	S	+	USA	New Orleans	SRR3334409
C00021016	OPT_2777	09-Mar-07	44	N	1	S	S	+	Canada	Calgary	SRR3333366
C00020918	OPT_2680	03-Oct-08	44	N	1	S	S	+	Canada	Toronto	SRR3333570
C00020841	OPT_2605	24-Sep-08	44	N	1	S	S	+	France	Amiens Cedex	SRR3334123
C00020690	OPT_2457	05-Aug-08	44	N	1	S	S	+	Germany	Regensburg	SRR3333742
C00010960	OPT_2239	25-Mar-09	45	N	1	S	S	+	Canada	Trois-Rivieres	ERS352380
C00020509	OPT_2269	24-Jul-09	45	N	1	S	S	+	France	Lille	SRR3333599
C00028646	OPT_2643	01-Aug-08	45	N	1	S	S	+	Germany	Frankfurt	SRR3334142

C00020887	OPT_2650	22-Sep-08	46	N	1	S	S	+	Belgium	Aalst	SRR3334117
C00020725	OPT_2491	07-Apr-09	46	N	1	S	S	+	Canada	Chicoutimi	SRR3334459
C00010947	OPT_2064	25-Sep-08	46	N	1	S	S	+	Canada	Montreal	ERS352328
C00020821	OPT_2585	10-Dec-08	46	N	1	S	S	+	Canada	Montreal	SRR3333042
C00028657	OPT_2847	15-Nov-07	46	N	1	S	S	+	Canada	Montreal	SRR3333408
C00010958	OPT_1894	01-Apr-09	46	N	1	S	S	+	Canada	Sherbrook	ERS352327
C00020600	OPT_2395	08-Jul-08	46	N	1	S	S	+	Canada	Sherbrooke	SRR3333772
C00020707	OPT_2473	24-Feb-09	46	N	1	S	S	+	Canada	Trois-Rivieres	SRR3339978
C00020257	OPT_1696	26-Jun-07	46	N	1	S	S	+	USA	Smyrna Beach	SRR3332951
C00020893	OPT_2655	22-Aug-08	47	N	2	S	S	+	USA	Maywood	SRR3333402
C00021073	OPT_2823	15-Jun-07	48	N	1	S	S	+	Canada	Calgary	SRR3333072
C00020672	OPT_2440	07-May-08	49	N	1	S	S	+	Belgium	Brussels	SRR3333302
C00020838	OPT_2602	18-Apr-08	49	N	1	S	S	+	Belgium	Brussels	SRR3340199
C00020882	OPT_2645	06-Jun-08	49	N	1	S	S	+	Belgium	Brussels	SRR3333622
C00020755	OPT_2521	05-Sep-09	49	N	1	S	S	+	Canada	Calgary	SRR3332945
C00011017	OPT_1874	18-Oct-07	49	N	1	S	S	+	USA	Chicago	ERS352329
C00020538	OPT_2334	03-Jan-08	49	N	1	S	S	+	USA	Miami	SRR3334052
C00021018	OPT_2779	17-Mar-07	53	N	1	S	S	+	Canada	Calgary	SRR3333385
C00020444	OPT_1873	09-Apr-08	53	N	1	S	S	+	Canada	Montreal	SRR3333018
C00021104	OPT_2853	06-Mar-08	53	N	1	S	S	+	Canada	Quebec City	SRR3334352
C00021107	OPT_2856	19-Mar-08	53	N	1	S	S	+	Canada	Sherbrooke	SRR3333273
C00020261	OPT_1700	16-Aug-07	53	N	1	S	S	+	Canada	Toronto	SRR3334076
C00020300	OPT_1748	13-May-08	53	N	1	S	S	+	Canada	Toronto	SRR3333029
C00020442	OPT_1858	06-Nov-07	53	N	1	S	S	+	USA	Chicago	SRR3340185
C00021060	OPT_2810	31-May-07	53	N	1	S	S	+	USA	Chicago	SRR3333725
C00020533	OPT_2329	19-Dec-07	53	N	1	S	S	+	USA	Decatur	SRR3339988
C00020894	OPT_2656	21-Aug-08	53	N	1	S	S	+	USA	Fargo	SRR3333694
C00020944	OPT_2707	20-Jul-06	53	N	1	S	S	+	USA	Lakewood	SRR3340064
C00021012	OPT_2773	12-Feb-07	53	N	1	S	S	+	USA	Lakewood	SRR3339985
C00020578	OPT_2373	18-Oct-07	53	N	1	S	S	+	USA	Newark	SRR3333786

C00020601	OPT_2396	26-Nov-07	53	N	1	S	S	+	USA	Newark	SRR3333747
C00020448	OPT_1882	11-Mar-08	54	N	1	S	S	+	Canada	Hamilton	SRR3340077
C00020544	OPT_2340	12-Feb-08	54	N	1	S	S	+	Canada	Windsor	SRR3334432
C00020926	OPT_2688	29-Oct-08	54	N	1	R	S	+	Germany	Magdeburg	SRR3340186
C00020650	OPT_2418	11-Feb-08	54	N	1	R	S	+	Sweden	Orebro	SRR3333697
C00010997	OPT_1729	13-Oct-07	55	N	1	S	S	+	Canada	Calgary	ERS352330
C00020245	OPT_1684	12-Jun-07	55	N	1	S	S	+	Canada	Calgary	SRR3334439
C00020268	OPT_1710	15-Sep-07	55	N	1	S	S	+	Canada	Calgary	SRR3334025
C00020285	OPT_1732	16-Oct-07	55	N	1	S	S	+	Canada	Calgary	SRR3334170
C00020298	OPT_1746	19-May-08	55	N	1	S	S	+	Canada	Calgary	SRR3333677
C00010926	OPT_2070	15-Dec-08	55	N	1	S	S	+	Canada	Chicoutimi	ERS352332
C00020930	OPT_2692	31-Oct-08	55	N	1	S	S	+	Canada	Chicoutimi	SRR3334120
C00011063	OPT_1836	21-Dec-07	55	N	1	S	S	+	Canada	Levis	ERS352387
C00020440	OPT_1853	31-Mar-08	55	N	1	R	S	+	Canada	Montreal	SRR3340081
C00010966	OPT_2272	05-Nov-09	55	N	1	S	S	+	Canada	Trois-Rivieres	ERS352331
C00028642	OPT_2455	14-Jul-08	55	N	1	S	S	+	Italy	Modena	SRR3333788
C00020760	OPT_2526	17-Jun-09	55	N	1	S	S	+	UK	Brighton	SRR3340036
C00020481	OPT_1938	29-May-09	55	N	1	S	S	+	USA	Fargo	SRR3333451
C00021050	OPT_2800	18-Apr-07	55	N	1	S	S	+	USA	Fort Worth	SRR3333026
C00011037	OPT_1920	02-Jun-07	55	N	1	S	S	+	USA	Lakewood	ERS352333
C00020933	OPT_2696	11-May-06	55	N	1	S	S	+	USA	Marietta	SRR3334087
C00020980	OPT_2742	21-Dec-06	55	N	1	S	S	+	USA	Marietta	SRR3340250
C00020724	OPT_2490	01-May-09	55	N	1	S	S	+	USA	Maywood	SRR3333059
C00020297	OPT_1745	14-May-08	55	N	1	S	S	+	USA	Minneapolis	SRR3333002
C00020899	OPT_2661	02-Oct-08	55	N	1	S	R	+	USA	Palm Springs and Rancho Mirage	SRR3334094
C00020761	OPT_2527	04-Mar-09	58	N	1	S	S	+	Belgium	Gent	SRR3333434
C00011003	OPT_1838	12-Feb-08	58	N	1	S	S	+	Canada	Calgary	ERS352334
C00020541	OPT_2337	10-Dec-07	58	N	1	S	S	+	Canada	Toronto	SRR3340011
C00020302	OPT_1752	29-Apr-08	58	N	1	S	S	+	Canada	Vancouver	SRR3334096
C00020488	OPT_2035	30-May-08	58	N	1	S	S	+	UK	Nottingham	SRR3333264

C00021067	OPT_2817	18-May-07	58	N	1	S	S	+	USA	Dothan	SRR3340046
C00020936	OPT_2699	22-May-06	58	N	1	S	S	+	USA	Marietta	SRR3340221
C00020564	OPT_2359	30-Mar-08	63	N	1	R	S	+	Canada	Calgary	SRR3334035
C00011008	OPT_2285	08-Sep-06	63	N	1	S	S	+	Canada	Montreal	ERS352336
C00021110	OPT_2859	22-May-08	63	N	1	R	S	+	Canada	Montreal	SRR3334108
C00020563	OPT_2358	12-Mar-08	63	N	1	R	S	+	Canada	Toronto	SRR3333560
C00011030	OPT_2292	26-Jun-06	63	N	1	R	S	+	USA	Akron	ERS352338
C00020450	OPT_1884	18-Oct-07	63	N	1	S	R	+	USA	Baltimore	SRR3333360
C00020451	OPT_1885	18-Nov-07	63	N	1	R	S	+	USA	Baltimore	SRR3333543
C00021019	OPT_2780	02-Mar-07	63	N	1	R	S	+	USA	Buffalo	SRR3340222
C00011055	OPT_2301	13-Feb-07	63	N	1	R	S	+	USA	Butte	ERS352337
C00020256	OPT_1695	23-Aug-07	63	N	1	R	S	+	USA	Butte	SRR3340181
C00020602	OPT_2397	03-Jan-08	63	N	1	R	S	+	USA	Butte	SRR3333380
C00020973	OPT_2735	01-Dec-06	63	N	1	R	R	+	USA	Camden	SRR3340051
C00020255	OPT_1694	07-Jun-07	63	N	1	R	S	+	USA	Charlottesville	SRR3333277
C00011019	OPT_1854	31-Jan-08	63	N	1	R	S	+	USA	Chicago	ERS352335
C00021015	OPT_2776	01-Mar-07	63	N	1	R	S	+	USA	Fort Worth	SRR3333287
C00020998	OPT_2759	02-Feb-07	63	N	1	R	S	+	USA	Lakewood	SRR3333691
C00020464	OPT_1913	19-Feb-07	63	N	1	R	S	+	USA	Marietta	SRR3333724
C00020939	OPT_2702	28-Jun-06	63	N	1	R	S	+	USA	Marietta	SRR3334175
C00020940	OPT_2703	05-Jul-06	63	N	1	R	S	+	USA	Marietta	SRR3339992
C00021063	OPT_2813	30-May-07	63	N	1	R	S	+	USA	Marietta	SRR3340198
C00020436	OPT_1833	16-Oct-06	63	N	1	R	R	+	USA	Minneapolis	SRR3333398
C00020983	OPT_2745	27-Dec-06	63	N	1	R	S	+	USA	Naperville	SRR3334302
C00021011	OPT_2772	23-Feb-07	63	N	1	R	S	+	USA	St. Louis	SRR3334031
C00021047	OPT_2797	20-Apr-07	63	N	1	R	R	+	USA	Summers Point	SRR3333015
C00020674	OPT_2442	09-Jan-08	67	N	2	S	S	+	Belgium	Aalst	SRR3340002
C00020455	OPT_1893	09-Jan-08	67	N	2	S	S	+	Canada	Calgary	SRR3333593
C00020474	OPT_1928	18-May-09	67	N	2	S	S	+	Canada	Calgary	SRR3340275
C00020583	OPT_2378	23-Nov-07	67	N	2	S	S	+	Canada	Calgary	SRR3334413

C00020945	OPT_2708	09-Aug-06	67	N	2	S	S	+	Canada	Calgary	SRR3334210
C00020946	OPT_2709	18-Aug-06	67	N	2	S	S	+	Canada	Calgary	SRR3333631
C00021029	OPT_2791	05-May-07	67	N	2	S	S	+	Canada	Calgary	SRR3333590
C00021101	OPT_2849	01-Feb-08	67	N	2	S	S	+	Canada	Calgary	SRR3332971
C00020817	OPT_2581	18-Nov-08	67	N	2	S	S	+	Canada	Chicoutimi	SRR3334162
C00020321	OPT_1808	29-Feb-08	67	N	2	S	S	+	Canada	Montreal	SRR3333080
C00020598	OPT_2393	13-Jul-08	67	N	2	S	S	+	Canada	Montreal	SRR3334445
C00021077	OPT_2826	28-Jun-07	67	N	2	S	S	+	Canada	Montreal	SRR3340276
C00010890	OPT_1869	13-Apr-08	67	N	2	S	S	+	Canada	Vancouver	SRR3334437
C00020884	OPT_2647	16-Sep-08	67	N	2	S	S	+	Sweden	Orebro	SRR3333564
C00011058	OPT_1834	19-Apr-08	67	N	2	S	S	+	USA	Butte	ERS352341
C00010945	OPT_2051	03-Oct-08	67	N	2	S	S	+	USA	Chicago	ERS352340
C00020568	OPT_2363	11-Apr-08	67	N	2	S	S	+	USA	Chicago	SRR3340040
C00020994	OPT_2756	25-Jan-07	67	N	2	S	S	+	USA	Cleveland	SRR3333272
C00020860	OPT_2623	30-Oct-08	67	N	2	S	S	+	USA	Idaho Falls	SRR3334194
C00020582	OPT_2377	11-Oct-07	67	N	2	S	S	+	USA	Keego Harbor	SRR3333416
C00020686	OPT_2453	26-Jun-08	69	N	1	S	R	+	France	Caen Cedex	SRR3340246
C00020778	OPT_2543	25-Jun-09	80	N	1	S	S	+	Canada	Calgary	SRR3340000
C00021021	OPT_2782	14-Mar-07	83	N	1	S	S	-	USA	Chicago	SRR3333699
C00020489	OPT_2038	17-Feb-07	95	N	2	S	S	+	Canada	Calgary	SRR3333597
C00020595	OPT_2390	17-Jun-08	95	N	2	S	S	+	Canada	Hamilton	SRR3333056
C00010912	OPT_1937	05-Feb-09	95	N	2	S	S	+	Sweden	Göteborg	SRR3340052
C00020845	OPT_2608	09-Dec-08	95	N	2	S	S	+	Sweden	Göteborg	SRR3333642
C00020749	OPT_2515	16-Oct-09	95	N	2	S	S	+	USA	Maywood	SRR3334454
C00020503	OPT_2237	27-Mar-09	95	N	2	S	S	+	USA	New Orleans	SRR3334164
C00020781	OPT_2546	11-Dec-07	95	N	2	S	S	+	USA	Savannah	SRR3334079
C00021003	OPT_2764	25-Jan-07	97	N	2	S	S	+	USA	Minneapolis	SRR3333449
C00020864	OPT_2627	31-Jan-09	102	N	1	S	S	+	Canada	Trois-Rivieres	SRR3334011
C00011004	OPT_1841	29-Mar-08	103	N	1	S	S	+	Canada	Calgary	ERS352342
C00020270	OPT_1713	22-Sep-07	103	N	1	S	S	+	Canada	Calgary	SRR3333103

C00020514	OPT_2311	02-Feb-07	103	N	1	S	S	+	Canada	Calgary	SRR3333328
C00020473	OPT_1927	07-May-09	103	N	1	S	S	+	Canada	Montreal	SRR3333344
C00020783	OPT_2548	21-Jul-09	107	N	1	S	S	-	Canada	Calgary	SRR3333036
C00020984	OPT_2746	04-Jan-07	109	N	4	S	S	-	Canada	Calgary	SRR3333545
C00020542	OPT_2338	04-Feb-08	110	N	1	S	S	+	USA	Chicago	SRR3334138
C00020558	OPT_2353	21-Nov-07	110	N	1	S	S	+	USA	Marietta	SRR3333715
C00010940	OPT_2249	16-Apr-09	122	N	-	S	S	+	Canada	Toronto	ERS216173
C00021028	OPT_2790	30-Apr-07	123	N	2	S	S	+	Canada	Calgary	SRR3340076
C00021074	OPT_2824	04-Jul-07	123	N	2	S	S	+	Canada	Calgary	SRR3340028
C00020732	OPT_2498	16-Mar-09	123	N	2	S	S	+	Canada	Montreal	SRR3334458
C00010941	OPT_2236	19-Mar-09	123	N	2	S	S	+	Canada	Toronto	ERS352345
C00020523	OPT_2320	14-Dec-07	123	N	2	S	S	+	USA	Chicago	SRR3333283
C00020607	OPT_2402	26-Jun-08	123	N	2	S	S	+	USA	Chicago	SRR3340020
C00020484	OPT_1940	05-Mar-09	123	N	2	S	S	+	USA	Idaho Falls	SRR3334032
C00010962	OPT_1964	15-Oct-09	140	N	2	S	S	+	Canada	Trois-Rivieres	ERS352379
C00020978	OPT_2740	05-Dec-06	149	N	1	S	S	+	Canada	Montreal	SRR3333994
C00021102	OPT_2850	04-Dec-07	156	N	2	S	S	+	USA	Marietta	SRR3333669
C00020591	OPT_2386	23-Apr-08	188	N	2	S	S	+	Canada	Montreal	SRR3340023
C00020865	OPT_2628	06-Feb-09	188	N	2	S	S	+	Canada	Trois-Rivieres	SRR3333617
C00020924	OPT_2686	10-Nov-08	188	N	2	S	S	+	Sweden	Orebro	SRR3332964
C00028647	OPT_2658	31-Jul-08	188	N	2	S	S	+	USA	Winchester	SRR3333428
C00020967	OPT_2729	07-Nov-06	192	N	2	R	S	+	Canada	Calgary	SRR3334151
C00020592	OPT_2387	16-Jun-08	192	N	2	S	S	+	USA	Chicago	SRR3333404
C00020566	OPT_2361	26-Apr-08	198	N	4	S	S	+	Canada	Calgary	SRR3334088
C00010904	OPT_2055	30-Nov-07	207	N	5	S	S	+	Spain	Barcelona	ERS352344
C00021080	OPT_2829	14-Jul-07	221	N	3	S	S	+	Canada	Calgary	SRR3334072
C00020547	OPT_2343	15-Feb-08	222	N	2	S	S	+	Canada	Calgary	SRR3333775
C00020986	OPT_2748	08-Jan-07	222	N	2	S	S	+	Canada	Calgary	SRR3333375
C00020786	OPT_2551	20-May-09	222	N	2	S	S	+	Canada	Montreal	SRR3333459

C00020663	OPT_2431	07-Nov-07	222	N	2	S	S	+	USA	Akron	SRR3333762
C00021089	OPT_2837	28-Jul-07	222	N	2	S	S	+	USA	Marietta	SRR3334216
C00020737	OPT_2503	15-May-09	222	N	2	S	S	+	USA	New Orleans	SRR3333620
C00010989	OPT_2283	17-May-07	223	N	2	S	S	+	Canada	Calgary	ERS352343
C00020575	OPT_2370	18-Jan-08	223	N	2	S	S	+	USA	Gainesville	SRR3333081
C00010968	OPT_2276	27-Nov-09	224	N	2	S	S	+	Canada	Trois-Rivieres	ERS352405
C00020557	OPT_2352	21-Feb-08	225	N	1	S	S	+	USA	Chicago	SRR3333609
C00020745	OPT_2511	08-Oct-09	226	N	2	S	S	+	Germany	Ulm	SRR3333411
C00020471	OPT_1925	15-Apr-09	227	N	2	S	S	+	Canada	Montreal	SRR3333608
C00020961	OPT_2723	27-Sep-06	228	N	2	S	S	+	USA	Akron	SRR3333702
C00020799	OPT_2563	22-Jul-09	229	N	2	S	S	+	Canada	Edmonton	SRR3333700
C00021001	OPT_2762	07-Feb-07	230	N	2	S	S	+	USA	Massapequa	SRR3340210
C00020810	OPT_2574	22-Jul-09	231	N	2	S	S	+	Canada	Calgary	SRR3333077
C00020251	OPT_1690	06-Sep-07	232	N	2	S	S	+	Canada	Calgary	SRR3333429
C00020570	OPT_2365	03-May-08	232	N	2	S	S	+	Canada	Calgary	SRR3334451
C00021051	OPT_2801	16-May-07	233	N	1	S	S	+	Canada	Calgary	SRR3333576
C00028641	OPT_2376	02-Oct-07	234	N	1	S	S	+	Canada	Chicoutimi	SRR3333546
C00020269	OPT_1712	18-Sep-07	235	N	1	S	S	+	Canada	Calgary	SRR3333229
C00020866	OPT_2629	17-Feb-09	236	N	1	S	S	+	Canada	Trois-Rivieres	SRR3334453
C00020248	OPT_1688	22-Aug-07	237	N	1	S	S	+	Canada	Calgary	SRR3333649
C00020757	OPT_2523	20-Oct-09	264	N	2	S	S	+	Germany	Köln	SRR3333602
C00020830	OPT_2594	05-Jan-09	265	N	1	S	R	+	Canada	Hamilton	SRR3340057

(e) Oxfordshire Healthy Human Infant Isolates (Non-clinical)

Genome Identification Number	Isolate Name	Isolation Date	ST	Phylogeny	clade	*gyrA*	*gyrB*	PaLoc	Country	SRA Accession
bd94e430-7ce6-4bc6-a045-1eb832735fcf	69f15a_36	10-Jul-13	2	Y	1	S	S	+	UK	SRR3317194
c3969c49-3c75-4941-8a7f-215cd2cebdf6	69f15a_55	31-Dec-11	2	Y	1	S	S	+	UK	SRR3317195
2c33a959-210a-4322-814d-844c2d686f56	69f15a_37	05-May-13	2	Y	1	S	S	+	UK	SRR3317172
C00007139	OVG007H-p1	03-Sep-11	2	Y	1	S	S	+	UK	SRR3339987
C00007184	BF083I-p1	01-Sep-11	2	Y	1	S	S	+	UK	SRR3333820
C00007583	BF085G-p1	26-Jul-11	2	Y	1	S	S	+	UK	ERS351881
C00007587	OVG010E-p1	20-Jul-11	2	Y	1	S	S	+	UK	ERS351883
C00007603	BF063I-p1	24-Apr-11	2	Y	1	S	S	+	UK	SRR3333759
C00007611	OVG014-p1	17-Mar-11	2	Y	1	S	S	+	UK	SRR3332976
C00007622	OVG005E-p1	05-Jun-11	2	Y	1	S	S	+	UK	ERS351898
C00007648	WDC021-p1	21-Apr-11	2	Y	1	S	S	+	UK	SRR3333341
C00007651	BF069A-p1	03-Feb-11	2	Y	1	S	S	+	UK	ERS351911
C00011280	BF091F-p1	13-Oct-11	2	Y	1	S	S	+	UK	SRR3334029
C00011295	BF308A-p1	17-Feb-12	2	Y	1	S	S	+	UK	SRR3334148
C00011303	OVG012H-p1	14-Oct-11	2	Y	1	S	S	+	UK	SRR3333722
C00011315	WBC259G-p1	03-Mar-12	2	Y	1	S	S	+	UK	SRR3333651
C00011331	WBC770C-p1	13-Oct-11	2	Y	1	S	S	+	UK	SRR3340237
C00011349	WBC794-p1	24-Nov-11	2	Y	1	S	S	+	UK	SRR3333613
C00011352	WBC819-p1	09-Mar-12	2	Y	1	S	S	+	UK	SRR3333795
C00011358	WDC774-p1	15-Sep-11	2	Y	1	S	S	+	UK	SRR3334059
C00015872	BF839E-p1	07-Aug-12	2	Y	1	S	S	+	UK	SRR3340245
C00017264	BF534D-p1	22-Feb-13	2	Y	1	S	S	+	UK	SRR3333684
C00020069	WBC871A-p1	09-May-12	2	Y	1	S	S	+	UK	SRR3333455
C00020082	WBC887F-p1	13-Oct-12	2	Y	1	S	S	+	UK	SRR3339991
C00020097	WDC867-p3	16-Apr-12	2	Y	1	S	S	+	UK	SRR3333463
C00020160	WBC507E-p1	29-Sep-12	2	Y	1	S	S	+	UK	SRR3340013

C00020163	WBC509G-p1	10-Dec-12	2	Y	1	S	S	+	UK	SRR3339996
C00020194	WBC779H-p1	05-May-12	2	Y	1	S	S	+	UK	SRR3333591
C00020198	WBC786I-p1	12-Jul-12	2	Y	1	S	S	+	UK	SRR3333461
C00020230	WBC852E-p1	03-Sep-12	2	Y	1	S	S	+	UK	SRR3333393
C00020236	WBC854F-p1	20-Sep-12	2	Y	1	S	S	+	UK	SRR3333465
406c231d-42c6-47c8-b430-7557a3e3318e	69f15a_38	08-Jun-13	2	Y	1	S	S	+	UK	SRR3317174
b6688e1e-3f8d-4b4c-9b61-41f924f2b621	69f15a_39	12-Jul-13	2	Y	1	S	S	+	UK	SRR3317192
c4811e20-2701-40c5-81b6-e04283211e48	69f15a_34	22-Feb-13	2	Y	1	S	S	+	UK	SRR3317196
fc6942b5-1f57-42a2-958d-dd86997d55df	69f15a_35	24-Apr-13	2	Y	1	S	S	+	UK	SRR3317205
C00007638	BF063D-p1	21-Oct-10	3	N	1	S	S	-	UK	ERS351904
C00007641	BF075A-p1	23-Dec-10	3	N	1	S	S	-	UK	SRR3333476
C00011297	BF831-p1	09-Mar-12	3	N	1	S	S	-	UK	SRR3333614
C00011340	WBC781-p1	13-Oct-11	3	N	1	S	S	-	UK	SRR3334050
C00020089	WBC889D-p3	08-Sep-12	3	N	1	S	S	-	UK	SRR3333480
C00020099	WDC869-p1	20-Apr-12	3	N	1	S	S	+	UK	SRR3333668
C00011329	WBC766D-p1	09-Nov-11	5	N	3	S	S	+	UK	SRR3333231
C00015895	BF859F-p1	11-Oct-12	5	N	3	S	S	+	UK	SRR3332947
94caeada-443d-4ad9-b946-995ea6e51893	69f15a_52	24-Mar-13	6	Y	1	S	S	+	UK	SRR3317185
0a7dacf6-e42e-457c-a3a8-51a965a0fc0b	69f15a_47	21-May-13	6	Y	1	S	S	+	UK	SRR3317166
4598ec0f-e178-4107-b5bf-c2f5f9db34f7	69f15a_54	03-May-13	6	Y	1	S	S	+	UK	SRR3317177
4361ba61-5f8f-4995-ad63-516fc0a31f10	69f15a_53	18-Apr-13	6	Y	1	S	S	+	UK	SRR3317175
C00007610	OVG005D-p1	09-May-11	6	Y	1	S	S	+	UK	ERS243672
C00011309	OVG016E-p1	19-Aug-11	6	Y	1	S	S	+	UK	SRR3340189
C00011346	WBC788C-p1	17-Feb-12	6	Y	1	S	S	+	UK	SRR3333800
C00015852	BF808H-p1	15-Aug-12	6	Y	1	S	S	+	UK	SRR3334111
C00015879	BF842E-p1	09-Jul-12	6	Y	1	S	S	+	UK	SRR3340078
C00020078	WBC876-p1	06-May-12	6	Y	1	S	S	+	UK	SRR3339969
C00020150	OVG002E-R-p1	22-May-11	6	Y	1	S	S	+	UK	SRR3333992
C00020173	WBC520-p1	21-Aug-12	6	Y	1	S	S	+	UK	SRR3333640
C00020188	WBC528D-p1	23-Dec-12	6	Y	1	S	S	+	UK	SRR3333061

C00020200	WBC789E-p1	22-Mar-12	6	Y	1	S	S	+	UK	SRR3333615
C00020213	WBC812E-p1	07-Jun-12	6	Y	1	S	S	+	UK	SRR3333696
C00004601	IBP30-p1	16-Dec-08	7	N	1	S	S	-	UK	ERS243708
C00011320	WBC271B-p1	11-Nov-11	7	N	1	S	S	+	UK	ERS243709
C00007577	BF007-p1	08-Mar-10	8	Y	1	S	S	+	UK	SRR3340251
C00007601	BF013I-p1	02-Jan-11	8	Y	1	S	S	+	UK	SRR3333100
C00007636	WDC020-p1	20-Apr-11	8	Y	1	S	S	+	UK	SRR3334402
C00011299	OVG004I-p1	21-Sep-11	8	Y	1	S	S	+	UK	SRR3334406
C00011310	OVG016F-p1	30-Aug-11	8	Y	1	S	S	+	UK	SRR3334128
C00011361	WDC849-p1	28-Mar-12	8	Y	1	S	S	+	UK	SRR3334047
C00020066	WBC856-p1	02-Apr-12	8	Y	1	S	S	+	UK	SRR3334301
C00020235	WBC853G-p1	28-Sep-12	8	Y	1	S	S	+	UK	SRR3340014
C00020238	WBC854H-p1	16-Nov-12	8	Y	1	S	S	+	UK	SRR3334165
C00020096	WDC539-p1	22-Nov-12	9	N	1	S	S	+	UK	SRR3334058
C00015841	BF515D-p1	13-Sep-12	11	N	5	S	S	+	UK	SRR3340271
C00015856	BF835H-p1	22-Oct-12	11	N	5	S	S	+	UK	SRR3334419
C00015891	BF848G-p1	13-Oct-12	11	N	5	S	S	+	UK	SRR3333039
C00020060	WBC854S1-p1	11-Apr-12	11	N	5	S	S	+	UK	SRR3333246
C00020193	WBC766I-p1	04-Apr-12	11	N	5	S	S	+	UK	SRR3334219
C00020196	WBC786G-p1	03-May-12	11	N	5	S	S	+	UK	SRR3333233
C00007668	NUR007-p1	09-Jun-11	12	N	1	S	S	+	UK	SRR3334415
C00020215	WBC813G-p1	27-Jul-12	13	N	1	S	S	+	UK	SRR3340216
C00011304	OVG012I-p1	28-Oct-11	14	N	1	S	S	+	UK	SRR3340194
C00015898	BF862C-p1	21-Jun-12	14	N	1	S	S	+	UK	SRR3333450
C00005936	KBP65-p1	28-Dec-08	15	N	1	S	S	-	UK	SRR3340211
C00005937	KBP89a-p1	13-Jan-09	15	N	1	S	S	-	UK	SRR3333004
C00005946	BF32-p1	23-Nov-08	15	N	1	S	S	-	UK	SRR3334433
C00005947	BF33-p1	30-Nov-08	15	N	1	S	S	-	UK	SRR3332963
C00005948	KBP99-p1	19-Jan-09	15	N	1	S	S	-	UK	SRR3334218

C00007595	BF087B-p1	18-Mar-11	15	N	1	S	S	-	UK	ERS351887
C00007599	OVG013E-p1	28-Jul-11	15	N	1	S	S	-	UK	SRR3333262
C00007602	BF063A-p1	21-Jun-10	15	N	1	S	S	-	UK	ERS351888
C00011284	BF092H-p1	13-Oct-11	15	N	1	S	S	-	UK	SRR3334181
C00011298	OVG001I-p1	07-Sep-11	15	N	1	S	S	-	UK	SRR3333433
C00011323	WBC301-p1	11-Aug-11	15	N	1	S	S	-	UK	SRR3333419
C00011324	WBC304G-p1	26-Jan-12	15	N	1	S	S	-	UK	SRR3333996
C00011325	WBC761-p1	24-Aug-11	15	N	1	S	S	-	UK	SRR3333003
C00011336	WBC779B-p1	16-Nov-11	15	N	1	S	S	-	UK	SRR3334176
C00011342	WBC783-p1	27-Oct-11	15	N	1	S	S	-	UK	SRR3333610
C00011360	WDC817-p1	17-Feb-12	15	N	1	S	S	-	UK	SRR3339977
C00015836	BF514E-p1	24-Oct-12	15	N	1	S	S	-	UK	SRR3332996
C00015911	BF893G-p1	02-Dec-12	15	N	1	S	S	-	UK	SRR3333308
C00020162	WBC509F-p1	15-Oct-12	15	N	1	S	S	-	UK	SRR3334411
C00020169	WBC516G-p1	02-Jan-13	15	N	1	S	S	-	UK	SRR3333107
C00020172	WBC519A-p1	21-Aug-12	15	N	1	S	S	-	UK	SRR3333581
C00020214	WBC812I-p1	21-Sep-12	15	N	1	S	S	-	UK	SRR3333351
C00028632	WBC526D-p1	24-Nov-12	15	N	1	S	S	-	UK	SRR3333023
C00020174	WBC524AA-p1	21-Aug-12	18	N	1	S	S	+	UK	SRR3333799
C00020207	WBC803E-p1	17-May-12	18	N	1	S	S	+	UK	SRR3334168
252e0278-78ca-4c63-ac0e-5ddb55e9a5ef	69f15a_48	21-Aug-12	18	N	1	S	S	+	UK	SRR3317170
C00011330	WBC769-p1	18-Aug-11	21	N	1	S	S	+	UK	SRR3333726
ea32ac4c-0296-47a4-8392-c405bb707de4	69f15a_40	11-Jan-11	26	N	1	S	S	-	UK	SRR3317203
C00005939	HBP24a-p1	07-Nov-08	26	N	1	S	S	-	UK	SRR3340266
C00005941	IBP32-p1	16-Dec-08	26	N	1	S	S	-	UK	SRR3333361
C00005942	KBP64-p1	21-Dec-08	26	N	1	S	S	-	UK	SRR3333439
C00005943	KBP73a-p1	05-Jan-09	26	N	1	S	S	-	UK	SRR3332956
C00005944	KBP75-p1	06-Jan-09	26	N	1	S	S	-	UK	SRR3334420
C00006337	HB43-p1	05-Dec-08	26	N	1	S	S	-	UK	SRR3333240
C00006338	IBP25-p1	15-Dec-08	26	N	1	S	S	-	UK	SRR3334374

C00007162	OVG015F-p1	22-Aug-11	26	N	1	S	S	-	UK	SRR3334353
C00007582	BF076A-p1	06-Jan-11	26	N	1	S	S	-	UK	ERS351880
C00007605	BF073G-p1	12-Jul-11	26	N	1	S	S	-	UK	ERS351890
C00007612	WDC014-p1	14-Feb-11	26	N	1	S	S	-	UK	SRR3334430
C00007649	BF042F-p1	12-Feb-11	26	N	1	S	S	-	UK	ERS351909
C00007656	NUR004-p1	02-Mar-11	26	N	1	S	S	-	UK	SRR3333044
C00007660	WDC023-p1	23-May-11	26	N	1	S	S	-	UK	SRR3334403
C00007667	BF091B-p1	21-May-11	26	N	1	S	S	-	UK	ERS351921
C00011335	WBC778-p1	06-Oct-11	26	N	1	S	S	-	UK	SRR3334099
C00011341	WBC782-p1	27-Oct-11	26	N	1	S	S	-	UK	SRR3333310
C00011354	WBC823-p1	09-Mar-12	26	N	1	S	S	-	UK	SRR3334173
C00011357	WDC760-p1	28-Jul-11	26	N	1	S	S	-	UK	SRR3334215
C00015819	BF065I-R-p1	26-Jul-11	26	N	1	S	S	-	UK	SRR3339962
C00015853	BF828H-p1	05-Oct-12	26	N	1	S	S	-	UK	SRR3340277
C00015912	BF895C-p1	31-Jul-12	26	N	1	S	S	-	UK	SRR3332981
C00020061	WBC855EA-p1	07-Sep-12	26	N	1	S	S	-	UK	SRR3333143
C00020155	WBC271I-p1	12-Jul-12	26	N	1	S	S	-	UK	SRR3340080
C00020212	WBC811I-p1	29-Sep-12	26	N	1	S	S	-	UK	SRR3333305
C00020226	WBC839C-p1	22-Jun-12	26	N	1	S	S	-	UK	SRR3333279
C00020187	WBC527-p1	20-Sep-12	27	N	1	S	S	-	UK	SRR3333706
C00005945	KBP102-p1	25-Jan-09	29	N	1	S	S	-	UK	ERS216162
C00006339	HB40-p1	04-Dec-08	29	N	1	S	S	-	UK	SRR3333319
C00011327	WBC765-p1	01-Sep-11	29	N	1	S	S	-	UK	SRR3333718
C00011359	WDC809-p1	27-Jan-12	29	N	1	S	S	-	UK	SRR3333286
C00006340	HB21-p1	12-Nov-08	30	N	4	S	S	-	UK	ERS216163
C00006341	HBP8a-p1	30-Oct-08	30	N	4	S	S	-	UK	ERS243548
315f1baf-704a-4754-82e8-4ba4b0f2f153	69f15a_43	03-Apr-13	35	N	1	S	S	+	UK	SRR3317173
bc558b98-ed89-4551-955d-8dd276d936c9	69f15a_42	02-Mar-13	35	N	1	S	S	+	UK	SRR3317193
C00011326	WBC762-p1	11-Aug-11	35	N	1	S	S	+	UK	SRR3333069
C00011343	WBC785-p1	10-Nov-11	35	N	1	S	S	+	UK	SRR3333814

C00017267	WBC519H-p1	01-Mar-13	35	N	1	S	S	+	UK	SRR3339994
C00020159	WBC505F-p1	06-Jan-13	35	N	1	S	S	+	UK	SRR3340050
dbe34ad8-2134-45ac-9ad5-04216f80ca2b	69f15a_46	22-Mar-13	35	N	1	S	S	+	UK	SRR3317198
65b70aea-217a-4aa9-ba50-b6978e1fd4e9	69f15a_45	01-Mar-13	35	N	1	S	S	+	UK	SRR3317180
C00007624	WDC019-p1	14-Apr-11	36	N	1	S	S	+	UK	SRR3333771
C00011344	WBC788A-p1	24-Nov-11	36	N	1	S	S	+	UK	SRR3334217
4581f748-e643-4e26-abd1-b2d6648d2282	69f15a_31	26-Aug-11	37	Y	4	S	S	+	UK	SRR3317176
50a30e88-5bd9-46c6-a962-6d85b9691cc6	69f15a_49	23-Feb-13	37	Y	4	S	S	+	UK	SRR3317178
d52274e0-7bcb-4268-a2ca-9948be0b4ec5	69f15a_32	01-Jan-12	37	Y	4	S	S	+	UK	SRR3317197
C00007593	BF072H-p1	20-Jul-11	37	Y	4	S	S	+	UK	SRR3332948
C00007596	BF252B-p1	30-Jul-11	37	Y	4	S	S	+	UK	SRR3334106
C00007597	NUR256-p1	07-Jul-11	37	Y	4	S	S	+	UK	SRR3334234
C00007598	OVG005B-p1	05-Mar-11	37	Y	4	S	S	+	UK	SRR3340073
C00007658	OVG007F-p1	20-Jul-11	37	Y	4	S	S	+	UK	ERS351914
C00011277	BF084I-p1	15-Sep-11	37	Y	4	S	S	+	UK	SRR3333089
C00011279	BF089I-p1	21-Oct-11	37	Y	4	S	S	+	UK	SRR3334470
C00011307	OVG013H-p1	14-Oct-11	37	Y	4	S	S	+	UK	SRR3333376
C00011350	WBC816A-p1	24-Feb-12	37	Y	4	S	S	+	UK	SRR3339993
C00011363	WDH806-p1	19-Jan-12	37	Y	4	S	S	+	UK	SRR3333427
C00015849	BF808E-p1	31-May-12	37	Y	4	S	S	+	UK	SRR3340004
C00015885	BF844H-p1	01-Oct-12	37	Y	4	S	S	+	UK	SRR3333321
C00015901	BF862F-p1	17-Sep-12	37	Y	4	S	S	+	UK	SRR3333430
C00015905	BF880B-p1	20-Jun-12	37	Y	4	S	S	+	UK	SRR3334121
C00017268	WBC524S2-p1	23-Feb-13	37	Y	4	S	S	+	UK	SRR3333672
C00020079	WBC877-p1	12-May-12	37	Y	4	S	S	+	UK	SRR3333412
C00020149	NUR009-R-p1	15-Apr-11	37	Y	4	S	S	+	UK	SRR3340281
C00020185	WBC526F-p1	21-Jan-13	37	Y	4	S	S	+	UK	SRR3333756
C00007671	WBC201I-p1	02-May-11	39	N	4	R	S	-	UK	ERS216165
C00011301	OVG010H-p1	13-Oct-11	39	N	4	S	S	-	UK	ERS243552
C00011348	WBC793-p1	24-Nov-11	39	N	4	R	S	-	UK	ERS243553

e30e4b51-bd8d-48f1-9b63-2b94dc346d54	69f15a_33	10-Nov-12	42	N	1	S	S	+	UK	SRR3317200
C00015833	BF503H-p1	01-Dec-12	42	N	1	S	S	+	UK	SRR3334048
C00015844	BF515H-p1	05-Jan-13	42	N	1	S	S	+	UK	SRR3334066
C00011292	BF254G-p1	23-Dec-11	45	N	1	S	S	+	UK	SRR3333657
C00020229	WBC852D-p1	11-Aug-12	46	N	1	S	S	+	UK	SRR3333565
C00006344	HB59-p1	21-Dec-08	48	N	1	S	S	-	UK	SRR3333251
C00015873	BF841E-p1	15-Jul-12	49	N	1	S	S	+	UK	SRR3334187
C00015886	BF845H-p1	10-Nov-12	49	N	1	S	S	+	UK	SRR3333818
C00011362	WDH257-p1	20-Jul-11	53	N	1	S	S	+	UK	SRR3339998
C00020192	WBC536-p1	23-Nov-12	53	N	1	S	S	+	UK	SRR3333281
1a951f3c-b2e0-47b6-8580-37814b6172c7	69f15a_41	18-Jan-11	54	N	1	S	S	+	UK	SRR3317168
C00007627	BF068F-p1	28-May-11	54	N	1	S	S	+	UK	ERS243570
C00007640	BF071E-p1	12-Apr-11	54	N	1	S	S	+	UK	ERS243571
C00007647	WBC201F-p1	18-Jan-11	54	N	1	S	S	+	UK	ERS243572
C00011316	WBC260D-p1	07-Jan-12	54	N	1	S	S	+	UK	SRR3334434
C00011353	WBC822-p1	09-Mar-12	54	N	1	S	S	+	UK	SRR3334429
C00015830	BF502B-p1	22-Jun-12	58	N	1	S	S	+	UK	SRR3334073
C00020088	WBC889B-p1	28-Jun-12	58	N	1	S	S	+	UK	SRR3332988
C00011314	OVG261A-p1	06-Sep-11	107	N	1	S	S	-	UK	ERS216171
C00020191	WBC533-p1	20-Dec-12	107	N	1	S	S	-	UK	SRR3333259
C00020156	WBC504-p1	01-Jun-12	125	N	1	S	S	-	UK	SRR3334000
C00015862	BF837B-p1	22-Apr-12	150	N	1	S	S	+	UK	SRR3334223
C00011356	WDC757-p1	01-Jul-11	236	N	1	S	S	+	UK	SRR3333387
C00011286	BF095I-p1	23-Dec-11	238	N	4	S	S	-	UK	ERS216172
C00007609	OVG001C-p1	15-Mar-11	239	N	1	S	S	+	UK	ERS351892

Table S2. Cross-correlation of antibiotic class with CDI incidence in England, Oxfordshire and Leeds

National data is based on years 2005-2012, where data were complete for England.
Community antibiotic use was not available for Oxfordshire or Leeds. CDI cases from each of these regions form ~1% of English cases and hence have lower power to identify correlations.
Highest correlation value per antibiotic class shown, with optimum lag (where lag 1 means antibiotic use in previous year is best predictor of CDI in current year).
For the more limited Leeds data, only a restricted number of antibiotic groups and only a time lag of 0 was available.
Antibiotics are ordered by association between English hospital+community use and English CDI incidence.
Approximate 95% CI are based on simulation, see supplementary methods.

**Antibiotic class**	**Source of prescribing (cross-correlation, lag, approximate 95% CI)**				
	**England (2005-2012, 318337 CDI cases)**			**Oxfordshire (2004-2013, 5301 CDI cases)**	**Leeds (2008-2014, 4230 CDI cases)**
	**Hospital and community**	**Community only**	**Hospital only**	**Hospital only**	**Hospital only**
Fluoroquinolones	0.998, 0 (0.837,0.998)	0.881, 0 (0.478,0.947)	0.932, 0 (0.662,0.967)	0.621, 1 (-0.086,0.810)	0.709, 0 (-0.107,0.896)
All cephalosporins	0.975, 0 (0.821,0.983)	0.937, 0 (0.678,0.966)	0.972, 0 (0.807,0.986)	0.730, 1 (0.150,0.860)	0.966, 0 (0.762,0.982)
1st generation cephalosporins	0.958, 0 (0.742,0.977)	0.906, 0 (0.477,0.956)	0.969, 0 (0.809,0.990)	0.827, 1 (0.226,0.909)	0.988, 0 (0.716,0.988)
2nd generation cephalosporins	0.948, 0 (0.718,0.973)	0.921, 0 (0.633,0.964)	0.993, 0 (0.854,0.993)	0.727, 0 (0.072,0.867)	0.938, 0 (0.725,0.973)
3rd generation cephalosporins	0.890, 1 (0.539,0.913)	0.950, 0 (0.794,0.982)	0.889, 1 (0.500,0.909)	-0.579, -1 (-0.691,-0.244)	0.584, 0 (0.000,0.835)
All antibiotics	-0.569, 1 (-0.674,-0.415)	-0.588, 1 (-0.682,-0.439)	0.294, 1 (-0.185,0.601)	0.128, 1 (-0.383,0.525)	0.776, 0 (0.033,0.908)
Extended spectrum penicillins	-0.585, -1 (-0.710,-0.361)	-0.600, 1 (-0.729,-0.403)	0.776, 0 (0.313,0.903)	0.839, 0 (0.245,0.901)	Not available
Beta-lactamase resistant penicillins	-0.645, 1 (-0.758,-0.461)	-0.622, 1 (-0.715,-0.473)	0.885, 0 (0.618,0.947)	0.669, 1 (-0.037,0.808)	Not available
Piperacillin/Tazobactam	-0.656, 1 (-0.784,-0.452)	-0.668, -1 (-0.659,0.367)	-0.656, 1 (-0.787,-0.445)	-0.488, 1 (-0.732,0.242)	-0.949, 0 (-0.972,-0.677)
Carbapenems	-0.667, 1 (-0.763,-0.511)	-0.575, 1 (-0.766,-0.090)	-0.668, 1 (-0.766,-0.511)	0.412, 1 (-0.198,0.709)	-0.703, 0 (-0.891,0.027)
Co-amoxiclav	-0.669, 1 (-0.753,-0.528)	-0.656, 1 (-0.746,-0.509)	-0.682, 1 (-0.769,-0.530)	-0.645, -1 (-0.725,-0.352)	-0.918, 0 (-0.964,-0.673)
All penicillin/beta-lactamase inhibitor combinations	-0.678, 1 (-0.760,-0.535)	-0.668, -1 (-0.732,-0.533)	-0.681, 1 (-0.777,-0.525)	-0.656, -1 (-0.735,-0.362)	Not available

Note: English antibiotic data only available as an aggregate across Trusts; data not available for Trust by Trust comparison.
